# Revision of the Agathidinae (Hymenoptera, Braconidae) of Vietnam, with the description of forty-two new species and three new genera

**DOI:** 10.3897/zookeys.54.475

**Published:** 2010-09-09

**Authors:** Cornelis van Achterberg, Khuat Dang Long

**Affiliations:** 1 Department of Terrestrial Zoology, NCB Naturalis, Postbus 9517, 2300 RA Leiden, The Netherlands; 2 Institute of Ecology & Biological Resources, Vietnam Academy of Science & Technology, 18 Hoang Quoc Viet Road, Cau Giay, Ha Noi, Vietnam

**Keywords:** Braconidae, Agathidinae, Agathis, Bassus, Biroia, Braunsia, Camptothlipsis, Coccygidium, Coronagathis, Cremnops, Disophrys, Earinus, Euagathis, Gyragathis, Gyrochus, Lytopylus, Therophilus, Troticus, Zelodia, new species, new genus, key, Vietnam

## Abstract

The species of seventeen genera of Agathidinae (Braconidae) from Vietnam are revised: Agathis Latreille, 1804, Bassus Fabricius, 1804; Biroia Szépligeti, 1900; Braunsia Kriechbaumer, 1894; Camptothlipsis Enderlein, 1920; Coccygidium de Saussure, 1892; Coronagathis **gen. n.** (type species: Coronagathis cornifera **sp. n.**); Cremnops Foerster, 1862; Disophrys Foerster, 1862; Earinus Wesmael, 1837; Euagathis Szépligeti, 1900; Gyragathis **gen. n.** (type species: Gyragathis quyi **sp. n.**), Gyrochus Enderlein, 1920; Lytopylus Foerster, 1862; Therophilus Wesmael, 1837; Troticus Brullé, 1846, and Zelodia **gen. n.** (type species: Zelomorpha varipes van Achterberg & Maetô, 1990). Keys to the Vietnamese species are given.

Sixty-five species are recognised, of which twelve species are newly recorded for Vietnam: Bassus albifasciatus (Watanabe, 1934), Coccygidium angostura (Bhat & Gupta, 1977), Cremnops atricornis (Smith, 1874), stat. n., Disophrys erythrocephala Cameron, 1900, Gyrochus yunnanensis Wang, 1984, Lytopylus romani (Shestakov, 1940), **comb. n.**, Therophilus festivus (Muesebeck, 1953), **comb. n.**, Therophilus javanus (Bhat & Gupta, 1977), **comb. n.**, Therophilus lienhuachihensis (Chou & Sharkey, 1989), **comb. n.**, Therophilus marshi (Bhat & Gupta, 1977), **comb. n.**, Zelodia absoluta (Chen & Yang, 1998), **comb. n.** and Zelodia longidorsata (Bhat & Gupta, 1977), **comb. n.**

Forty-two species are new to science: Agathis citrinisoma **sp. n.**, Bassus albobasalis **sp. n.**, Bassus albozonatus **sp. n.**, Biroia soror **sp. n.**, Braunsia bicolorata **sp. n.**, Braunsia devriesi **sp. n.**, Braunsia maculifera **sp. n.**, Braunsia nigrapiculata **sp. n.**, Braunsia pumatica **sp. n.**, Camptothlipsis hanoiensis **sp. n.**, Coronagathis cornifera **sp. n.**, Earinus aurantius **sp. n.**, Earinus brevistigmus **sp. n.**, Euagathis flavosoma **sp. n.**, Disophrys maculifera **sp. n.**, Disophrys quymanhi **sp. n.**, Disophrys rhinoides **sp. n.**, Gyragathis quyi **sp. n.**, Therophilus annuliferus **sp. n.**, Therophilus cattienensis **sp. n.**, Therophilus contrastus **sp. n.**, Therophilus crenulisulcatus **sp. n.**, Therophilus depressiferus **sp. n.**, Therophilus elongator **sp. n.**, Therophilus levisoma **sp. n.**, Therophilus marucae **sp. n.**, Therophilus mellisoma **sp. n.**, Therophilus nigrolineatus **sp. n.**, Therophilus nuichuaensis **sp. n.**, Therophilus parasper **sp. n.**, Therophilus planifrons **sp. n.**, Therophilus punctiscutum **sp. n.**, Therophilus robustus **sp. n.**, Therophilus rugosiferus **sp. n.**, Therophilus scutellatus **sp. n.**, Troticus alloflavus **sp. n.**, Troticus giganteus **sp. n.**, Zelodia albobasalis **sp. n.**, Zelodia anginota **sp. n.**, Zelodia bicoloristigma **sp. n.**, Zelodia brevifemoralis **sp. n.** and Zelodia flavistigma **sp. n.**

The following new synonyms are proposed: Euagathis nigrithorax Bhat & Gupta, 1977, Euagathis variabilis Enderlein, 1920, Euagathis variabilis var. tibialis Enderlein, 1920, Euagathis variabilis var. melanopleura Enderlein, 1920 and Euagathis variabilis var. sucarandana Enderlein, 1920 with Euagathis abbotti (Ashmead, 1900); Euagathis jinshanensis Chen & Yang, 2006 and Euagathis sharkeyi Chen & Yang, 2006, with Euagathis forticarinata (Cameron, 1899). The genus Amputostypos Sharkey, 2009, is synonymised with Coccygidium de Saussure, 1892, syn. n.

The following new combinations are given: Bassus subrasa (Enderlein, 1920), **comb. n.**, Gyragathis angulosa (Bhat & Gupta, 1977), **comb. n.**, Lytopylus romani (Shestakov, 1940), **comb. n.**, Therophilus annulus (Chou & Sharkey, 1989), **comb. n.**, Therophilus asper (Chou & Sharkey, 1989), **comb. n.**, Therophilus cingulipes (Nees, 1812), **comb. n.**, Therophilus daanyuanensis (Chen & Yang, 2006), **comb. n.**, Therophilus fujianicus (Chen & Yang, 2006), **comb. n.**, Therophilus javanus (Bhat & Gupta, 1977), **comb. n.**, Therophilus lanyuensis (Chou & Sharkey, 1989), **comb. n.**, Therophilus luzonicus (Bhat & Gupta, 1977), **comb. n.**, Therophilus muesebecki (Bhat & Gupta, 1977), **comb. n.**, Therophilus rudimentarius (Enderlein, 1920), **comb. n.**, Therophilus similis (Bhat & Gupta, 1977), **comb. n.**, Therophilus sungkangensis (Chou & Sharkey, 1989), **comb. n.**, Therophilus tanycoleosus (Chen & Yang, 2006), **comb. n.**, Therophilus tonghuaensis (Chen & Yang, 2006), **comb. n.**, Therophilus tongmuensis (Chen & Yang, 2006), **comb. n.**, Therophilus transcasperatus (Chen & Yang, 2006), **comb. n.**, Troticus latiabdominalis (Bhat, 1978),**comb. n.**, Zelodia absoluta (Chen & Yang, 1998), **comb. n.**, Zelodia achterbergi (Chen & Yang, 2006), **comb. n.**, Zelodia albopilosella (Cameron, 1908), **comb. n.**, Zelodia chromoptera (Roman, 1913), **comb. n.**, Zelodia nihonensis (Sharkey, 1996), **comb. n.**, Zelodia cordata (Bhat & Gupta, 1977), **comb. n.**, Zelodia diluta (Turner, 1918), **comb. n.**, Zelodia dravida (Bhat & Gupta, 1977), **comb. n.**, Zelodia exornata (Turner, 1918), **comb. n.**, Zelodia longidorsata (Bhat & Gupta, 1977), **comb. n.**, Zelodia longiptera (Yang & Chen, 2006), **comb. n.**, Zelodia maculipes (Cameron, 1911), **comb. n.**, Zelodia nigra (Bhat & Gupta, 1977), **comb. n.**, Zelodia philippinensis (Bhat & Gupta, 1977), **comb. n.**, Zelodia reticulosa (Yang & Chen, 2006), **comb. n.**, Zelodia quadrifossulata (Enderlein, 1920), **comb. n.**, Zelodia ruida (Sharkey, 1996), **comb. n.**, Zelodia similis (Bhat & Gupta, 1977), **comb. n.**, Zelodia penetrans (Smith, 1860), **comb. n.** and Zelodia varipes (van Achterberg & Maetô, 1990), **comb. n.**

## Introduction

Members of the moderately large subfamily Agathidinae Nees, 1814 (Hymenoptera: Ichneumonoidea: Braconidae), from Vietnam have only recently been extensively studied; consequently, no reliable keys to species are available. The few keys available for the Oriental Region are outdated (e.g., Bhat and Gupta 1977) or are limited to an island (Chou and Sharkey 1989). Those limited to a country (Sharkey 1996, Chen and Yang 2006) deal with a partly or completely East Palaearctic fauna. Only the genus Euagathis Szépligeti, 1900, has received more attention (van Achterberg and Chen 2002). This paper is an attempt to give an overview of the diversity of Agathidinae of Vietnam and to supply keys to the taxa. For the first time all available Vietnamese species are illustrated by colour photographs. The study of variation in most species is severely hampered by the lack of specimens, so the keys presented here are just a start to a better understanding of the diversity of the subfamily in Vietnam. The paper by Sharkey et al. (2009) on the Oriental genera of Agathidinae (with special attention to the fauna of Thailand) appeared just before the present paper was finished and deals with a similar fauna. It is an important attempt to summarize the latest results on the phylogeny of the subfamily and deals with some recent finds in Thailand. However, the number of genera found in Vietnam is higher than in Thailand; e.g. the genus Bassus Fabricius s.s. is not recorded for Thailand, and we found two new genera in Vietnam. The different interpretation of some diagnostic characters, the use of additional characters, the correct interpretation of a type species and the new genera are sufficient reason to include a new key to the genera in our review. We propose another generic name (Zelodia van Achterberg gen. n.) for one genus following the correct interpretation of a type species. In addition, keys to all known species from Vietnam are presented and the 42 species new to science are described and illustrated. The new species represent two-thirds of the total number of species of Agathidinae known from Vietnam.

The biology of most species is unknown, but in general agathidines are koinobiont endoparasitoids of larvae of Lepidoptera. The species with a short ovipositor select exposed larvae and those with a long ovipositor use larvae with a concealed way of life.

## Material and methods

Two recent (and probably largest) collections of agathidines from Vietnam are used for this revision: the Braconidae collection in the Institute of Ecology & Biological Resources (IEBR) at Hanoi (assembled by the second author) and the NCB Naturalis collection (RMNH) at Leiden (assembled during five RMNH-IEBR expeditions in Vietnam). For identification of the subfamily Agathidinae, see van Achterberg (1990, 1993, 1997), for references to the genera, see Yu et al. (2007) and Sharkey et al. (2009; but genus Bassus omitted in the description part) and for the terminology used in this paper (except for the stigmal spot), see van Achterberg (1988, 1993). The stigmal spot is a well defined and more or less circular dark brown patch below the parastigma present in many species (Figs 57, 159; Fig. 113 in Bhat and Gupta 1977; Figs 19–21, 26–28 in Simbolotti & van Achterberg, 1995). The ramellus is the short vein externally connected to the second submarginal cell of the fore wing (Figs 47, 57, 117). Measurements are taken as indicated by van Achterberg (1988). Additional non-exclusive characters in the key are between brackets. In the keys to species sometimes notes to similar species unknown from Vietnam are included because they may occur in Vietnam. Putative apomorphies were determined through outgroup comparison making assumptions about the placement of certain taxa in Agathidinae according to the cladistic analysis by Sharkey et al. (2006). No taxonomic history is presented in this paper; for information is referred to the Taxapad interactive catalogue (Yu et al. 2007 and later updates).

## Key to genera of the subfamily Agathidinae Haliday known from Vietnam

**Table:** 

1.	Fore tarsal claws bifurcate, the inner tooth usually large and with no lobe (a); outer face of middle tibia without submedial pegs (b), but with one peg in Coronagathis; area between antennal sockets with a pair of crests or carinae (c), less frequently (about 30 %) with a trough (cc) or tubercle	2
	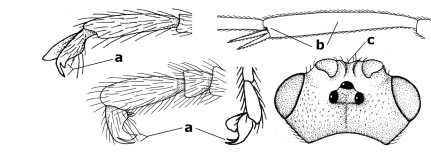	
–	Fore tarsal claws simple or with a more or less lamelliform, basal lobe (aa); outer face of middle tibia with pegs submedially or pegs in a subapical cluster (bb); area between antennal sockets usually with a simple median elevation or with a trough (cc)	13
	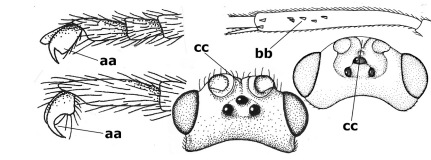	
2.	Notauli completely absent and area smooth (a); gena more or less protruding postero-ventrally (b); inner tooth of bifurcate fore claws larger than its outer tooth (female) or teeth equal (male) (c)	3
	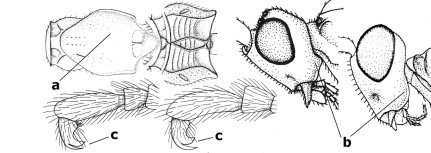	
–	Notauli at least in anterior half of mesoscutum impressed (aa), if shallowly impressed then area distinctly sculptured; gena variable postero-ventrally (bb); inner tooth of bifurcate fore claws as large as its outer tooth or smaller (both sexes; cc)	4
	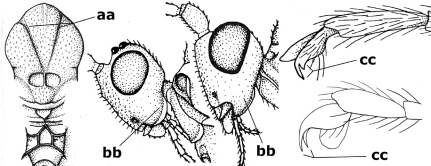	
3.	Outer ventral margin of hind trochantellus with a carina (a) and basally angulate; ovipositor sheath short, about as long as apical height of metasoma; vein 2r-m of hind wing absent (b); [scutellum with subposterior elevation (but sometimes reduced); epomial carinae double or single; semi-circular crest present behind antennal sockets; vein SR1 of fore wing rather curved]	Gyrochus Enderlein, 1920
	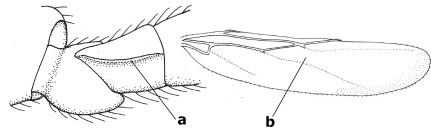	
–	Outer ventral margin of hind trochantellus without carina (aa) and not or only slightly angulate basally; ovipositor sheath long, about as long as fore wing or somewhat shorter; vein 2r-m of hind wing indicated (bb)	Biroia Szépligeti, 1900
	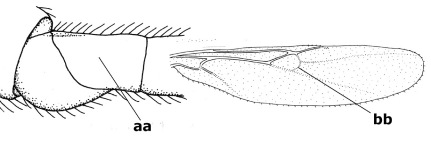	
4.	Inner spur of middle tibia 0.8–1.1 times as long as middle basitarsus (a); apex of antenna with short to medium-sized spine (b), sometimes minute; ovipositor sheath short, about as long as apical height of metasoma, hardly or not protruding; hind trochantellus usually with a distinct ventral carina on its outer edge (c) or edge distinctly angulate; [apex of the ovipositor sheath blunt apically and with numerous ampulliform papillae]; Coccygidium complex	5
	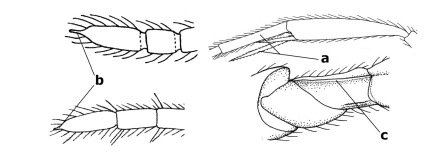	
–	Inner spur of middle tibia 0.4–0.7 times as long as middle basitarsus (aa); apex of antenna without spine (bb); relative length of ovipositor sheath vari able; ventral carina of hind trochantellus often (about two-thirds) absent or obsolescent (cc)	7
	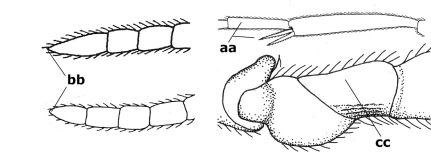	
5.	Malar suture long and deep (a); outer and inner side of antennal socket strongly protruding (b); eye with distinct subocular groove posteriorly (c); [hind coxa much longer than first tergite; not yet found but may occur in South Vietnam]	Hypsostypos Baltazar, 1963
	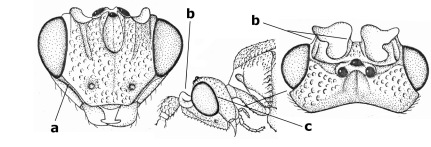	
–	Malar suture absent (aa); outer side of antennal socket weakly protruding (bb), eye without subocular groove posteriorly (cc)	6
	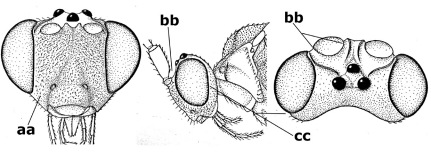	
6.	Fore tibial spur with a long curved and glabrous apical spine (a); malar space rugulose (b); notauli comparatively wide (c); hind coxa with longitudinal carina or rugae (d); frons with lateral crests or carinae more or less developed (e)	Coccygidium de Saussure, 1892
	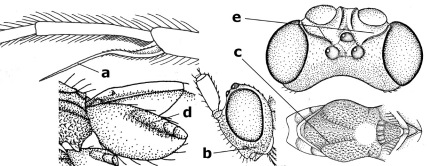	
–	Fore tibial spur with a short, straight and setose apex (aa); malar space smooth or punctate (bb); notauli comparatively narrow (cc); hind coxa without longitudinal carina or rugae (dd); frons without lateral crests or carinae (ee); [area between antennal sockets without a pair of carinae; temples medium-sized; ventral surface of hind femur densely rugose]	Zelodia gen. n.
	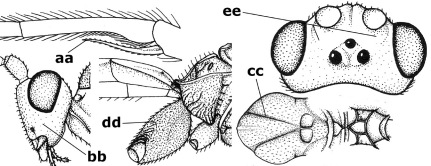	
7.	Inner or outer hind claw with a distinct wide and acute lobe submedially or a wide dark obtuse submedial lamella (a); if rather narrow then about as long as apical tooth; outer aspect of scapus partly smooth and somewhat concave (b); ovipositor sheath slender and much longer than apical height of metasoma (c); [frons without a pair of lamellae between antennal sockets]	8
	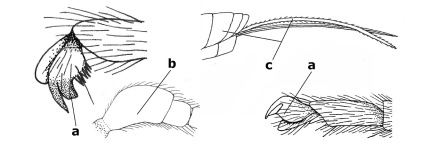	
–	Inner and outer hind claw bifurcate, with a small subapical inner tooth or (nearly) simple (aa); if with a rather flattened inner tooth then this distinctly shorter than apical tooth; outer aspect of scapus punctate or punctulate and usually less concave (bb); ovipositor sheath more or less widened and short, about as long as apical height of metasoma or less (cc)	9
	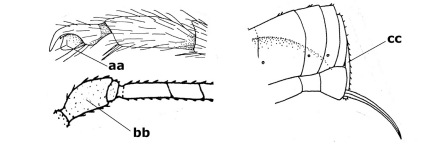	
8.	Outer hind tarsal claw with a distinct lobe similar to lobe of inner hind tarsal claw and no pecten (a); area near inner side of antennal sockets shallowly depressed (b); mesosternal sulcus deep and coarsely crenulate; hind trochantellus more or less carinate ventrally (c; but may be rounded or nearly so); at least posterior half of precoxal sulcus distinctly crenulate (d); [not yet found but likely to occur in North Vietnam]	Cremnoptoides van Achterberg & Chen, 2004
	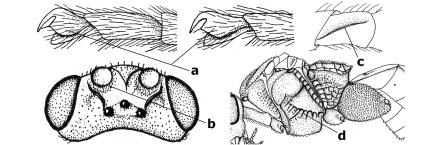	
–	Outer hind tarsal claw with a dentiform or squarish black lamella and more or less with a black pecten basally and different from lobed inner claw (aa); area near inner side of antennal sockets deeply depressed (bb); mesosternal sulcus shallow and smooth or nearly so; hind trochantellus rounded ventrally (cc); precoxal sulcus absent or nearly so (dd)	Cremnops Foerster, 1862
	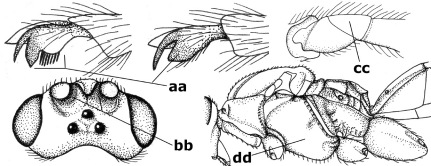	
9.	Scutellum with a pair of large horns and axillae protruding (a); subbasal cell of hind wing narrower than plical lobe (b); hind coxa long, exceeding length of first tergite and coarsely sculptured (c)	Coronagathis gen. n.
	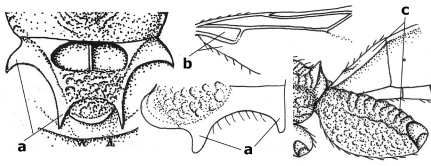	
–	Scutellum without a pair of tubercles (aa); subbasal cell of hind wing about as wide as plical lobe (bb); hind coxa shorter, at most as long as first tergite (cc), if rarely somewhat longer than coxa largely smooth	10
	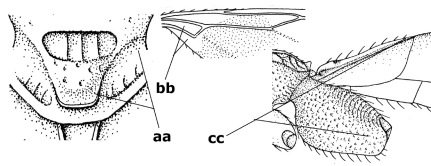	
10.	Frons without lateral carinae (a); vein M+CU of hind wing at most 0.8 times vein 1-M (b); labio-maxillary complex only slightly protruding (c)	Euagathis Szépligeti, 1900
	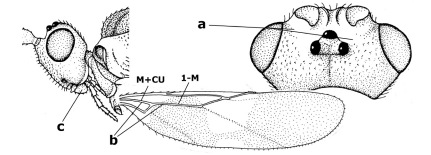	
–	Frons with lateral carinae (aa); vein M+CU of hind wing longer than vein 1-M or subequal (up to 0.9 times; bb); labio-maxillary complex usually rather protruding (cc, but not in Oreba)	11
	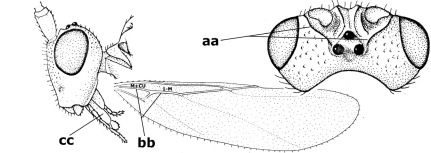	
11.	First subdiscal cell of fore wing distinctly higher than first discal cell (a); body metallic; marginal cell of hind wing ends in front of wing apex (b); vein m-cu of fore wing subparallel to vein 1-M (c); area between antennal sockets with an obtuse tubercle (d); [outer ventral margin of hind trochantellus cariniform; only the North Oriental type species (Oreba purpurea Cameron, 1900) is known; not yet found in Vietnam but may occur in the northern mountains]	Oreba Cameron, 1900
	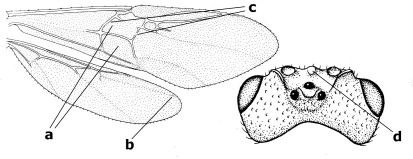	
–	First subdiscal cell of fore wing lower than first discal cell (aa; Disophrys) or subequal (aaa; Troticus); body non-metallic; marginal cell of hind wing close to wing apex (bb); vein m-cu of fore wing diverging from vein 1-M (c); area between antennal sockets with a pair of carinae or crests (dd)	12
	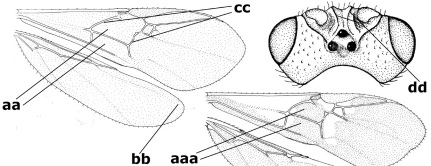	
12.	Prepectal carina distinctly angulate subdorsally (a); lateral carina of frons pointing to anterior ocellus or between anterior and posterior ocelli (b); scapus robust compared to third antennal segment and straight (c); [hind trochantellus rounded ventrally]	Troticus Brullé, 1846
	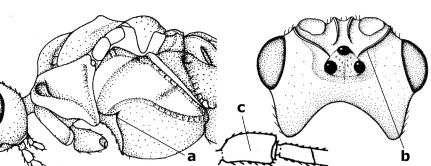	
–	Prepectal carina evenly curved subdorsally (aa); lateral carina of frons pointing to posterior ocellus (bb); scapus less robust compared to third antennal segment and more or less bent (cc)	Disophrys Foerster, 1862
	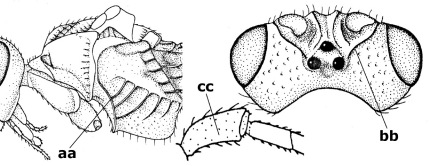	
13.	Veins of fore wing largely absent (a); hind trochantellus much longer than trochanter (b); hind tibia with blackish bristles (c); pedicellus enlarged (d); [not yet found in Vietnam, but expected to occur]	Aneurobracon Brues, 1930
	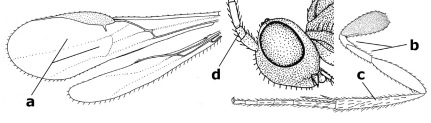	
–	Veins of fore wing largely present (aa); hind trochantellus about as long as trochanter or shorter (bb); hind tibia without black bristles (cc); pedicellus short (dd)	14
	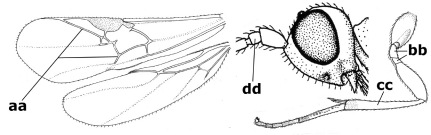	
14.	Vertical axis [v] of malar triangle 1.7–6.0 times its horizontal axis [h] and the part of head below eyes only gradually narrowed ventrally (a) to parallel-sided; clypeus strongly convex (b); mouth-parts more or less lengthened in form of a beak, galea nearly always distinctly longer than 1.3 times its width, longer than labial palp (c); [mainly Holarctic, but some species reach the northern Oriental region]	Agathis Latreille, 1804
	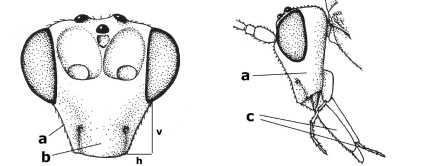	
–	Vertical axis [v] of malar triangle 1.0–1.5 times its horizontal axis [h] and the part of head below eyes directly narrowed ventrally (aa); clypeus usually at least partly flattened (bb), only in Therophilus mediator-group distinctly convex (bbb); mouth-parts normal, galea not longer than wide, shorter than labial palp and usually hardly or not visible in lateral view (cc)	15
	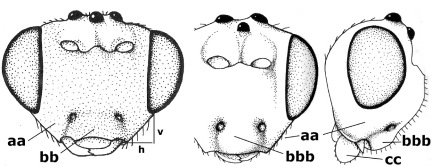	
15.	Fore and middle tarsal claws simple and comparatively robust (a); area behind antennal sockets deeply impressed (b); [vein 1-M of hind wing 1.1–1.6 times as long as vein M+CU]	Bassus Fabricius, 1804 s.s.
	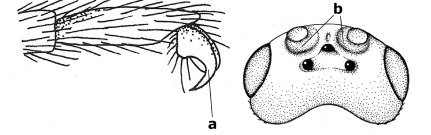	
–	Fore and middle tarsal claws nearly always with a distinct basal lobe and comparatively slender (aa); area behind antennal sockets nearly always moderately to shallowly impressed (bb); [vein 1-M of hind wing 0.6–1.4 times as long as vein M+CU]	16
	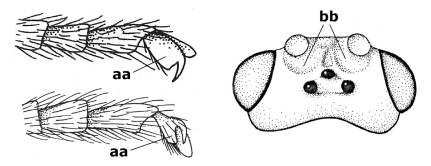	
16.	Vein 1-SR+M of fore wing more or less developed (a), usually sclerotised medially, but sometimes only as a brownish pigmented stripe and notauli absent (but mesoscutum medio-posteriorly with a deep to superficial depression; b)	Earinus Wesmael, 1837
	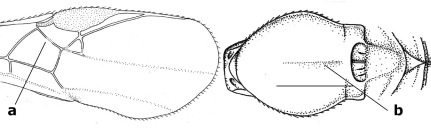	
–	Vein 1-SR+M of fore wing medially absent, not sclerotised and usually without pigmentation (aa), if pigmented then notauli complete (bb)	17
	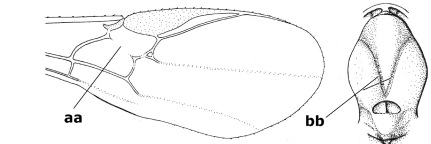	
17.	Antennal sockets with a circular carina reaching dorsally as high as level of anterior ocellus (a); temples with a lateral tubercle (b); genal protuberance present and protruding ventrally (c); scutellum with a distinct subposterior crest (d) and medio-posterior depression transverse (e); [transverse metasternal carina non-lamelliform and beyond upper level of hind coxal cavities]	Gyragathis gen. n.
	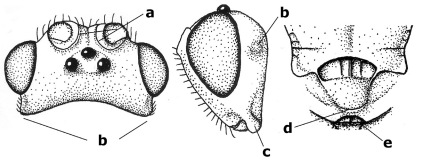	
–	Antennal sockets without a circular carina (aa); temples without a lateral tubercle (bb); protuberance absent (cc), if present then protruding posteriorly; scutellum without a distinct subposterior crest (dd) and transverse medio-posterior depression absent, obsolescent or semicircular (ee)	18
	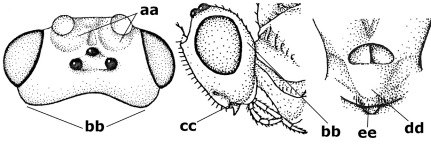	
18.	Outer side of antennal sockets distinctly lamelliform protruding in dorsal view (a); subpronope deep, wide and epomia strongly developed (b); basal half of third metasomal tergite usually with sharp lateral margin (c); dorsal carinae of first tergite usually long and costate (d); propodeal spiracle comparatively large and elliptical (e); ramellus of fore wing usually present (f); [transverse metasternal carina lamelliform; no subposterior crest of scutellum]	Braunsia Kriechbaumer, 1894
	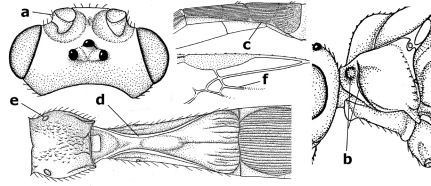	
–	Outer side of antennal sockets not or only slightly protruding in dorsal view (aa), sometimes as a narrow lamella; subpronope comparatively shallow and epomia medium-sized to weakly developed (bb); basal half of third tergite often without sharp lateral margin or weakly developed (cc), except in Lytopylus; dorsal carinae of first tergite usually weakly developed (dd) or absent; propodeal spiracle medium-sized to small, round or sub-elliptical (ee); ramellus of fore wing absent (ff)	19
	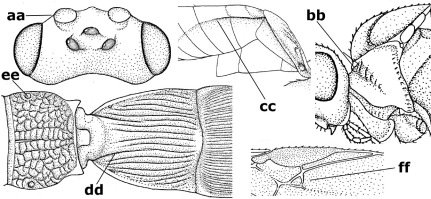	
19.	Vein r-m of fore wing absent (a); first metasomal tergite and often also metapleuron granulate and dull and tergite distinctly convex medially and dorsal carinae absent (b); precoxal sulcus about 0.6 times as long as mesopleuron and sculptured (c) or superficially impressed and smooth	Camptothlipsis Enderlein, 1920
	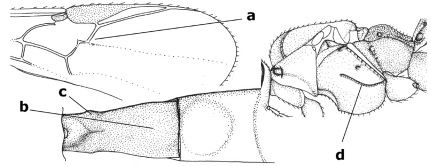	
–	Vein r-m of fore wing present (aa), rarely obsolescent; sculpture of first tergite and metapleuron variable (bb), if granulate then first tergite less convex medially and with dorsal carinae (bbb); precoxal sulcus longer than 0.6 times length of mesopleuron and sculptured (cc) or absent	20
	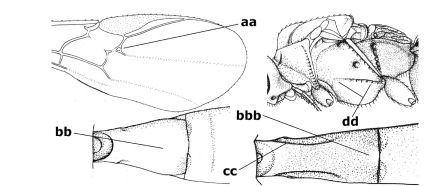	
20.	First metasomal tergite 5–6 times as long as wide apically (a) and 1.6–1.7 times as long as hind coxa (b); frons flattened anteriorly except for a short median depression (c); hind femur short compared to hind coxa (d); hind basitarsus with numerous spiny setae ventrally (e); [not yet found in Vietnam but expected to occur; synonymised with Lytopylus Foerster by Sharkey et al. (2009), but it runs in their key to Therophilus Wesmael. Provisionally retained as separate genus because of the synapomorphous character states (a-d) listed above]	Facilagathis van Achterberg & Chen, 2004
	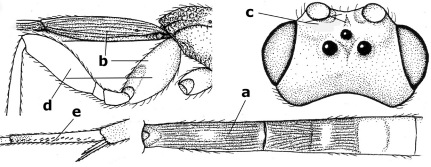	
–	First tergite 1–3 times as long as wide apically (aa) and about as long as hind coxa (bb); frons partly convex anteriorly and behind antennal sockets concave (cc); hind femur longer compared to hind coxa (dd); hind basitarsus only bristly setae ventrally (ee)	21
	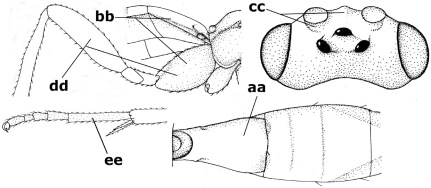	
21.	Metasomal cavity [mc] at most reaching upper level of hind coxal cavities [hcc] (a); transverse metasternal carina [tmc] (= carina behind metasomal cavity) straight or nearly so and coarsely developed (b); anterior half of third metasomal tergite often more or less coarsely striate (c), but sometimes smooth	Lytopylus Foerster, 1862
	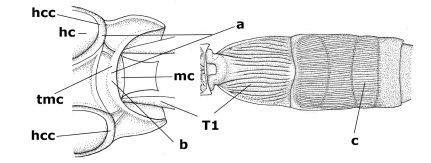	
–	Metasomal cavity [mc] protruding beyond upper level of hind coxal cavities [hcc] (aa); transverse metasternal carina [tmc] curved and less developed (bb); sculpture of anterior half of third tergite variable, usually smooth or finely striate (cc)	Therophilus Wesmael, 1837
	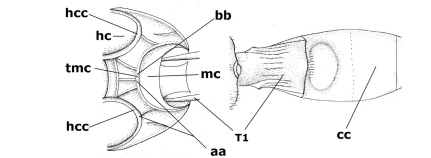	

## Systematics

### Genus Agathis

Latreille, 1804

#### Note.

In Vietnam only one species has been found, Agathis citrinisoma sp. n., which is rather aberrant because of its colouration (metasoma entirely yellow and strongly contrasting with black metasoma) and the partly developed vein 1-SR+M of the fore wing (Fig. 5). It has the first metasomal tergite very robust (as long as wide apically), superficially finely striate and partly smooth (Fig. 9).

#### Agathis citrinisoma sp. n.

urn:lsid:zoobank.org:act:6F759EE8-DC7C-49AA-ADB0-BAFA83EED7E9

Figs 1 –10 

##### Type material.

Holotype, ♀ (RMNH), “S. Vietnam: Dak Lak, Chu Yang Sin N.P., n[ea]r dam, 800–940 m, 2–10.vi.2007, Mal traps, C. v. Achterberg & R. de Vries, RMNH’07”.

##### Diagnosis.

Agathis citrinisoma runs in the key by Sharkey (1996) to Agathis asternaulica Sharkey, 1996, from Japan; however, this species has the metasoma largely black (citrinisoma: yellow), no trace of vein 1-SR+M of the fore wing (citrinisoma: partly developed) and the notauli are smooth posteriorly (citrinisoma: crenulate).

##### Description.

Holotype, ♀, length of body 4.0 mm, of fore wing 3.7 mm, of ovipositor sheath 2.0 mm.

###### Head.

Antennal segments 34, length of third segment 1.5 times fourth segment, length of third, fourth and penultimate segments 3.7, 2.8 and 1.7 times their width, respectively; length of apical antennal segment 1.6 times as long as penultimate segment; maxillary palp 0.7 times height of head; malar space 2.8 times as long as basal width of mandible; in dorsal view temple short, length of eye 5.3 times temple; temple directly narrowed posteriorly (Fig. 7); POL:OD:OOL= 8:4:10; face shiny with no carinae and with short medial groove between antennal sockets, sparsely and finely punctate and setose; frons, vertex and temple shiny and smooth (Fig. 7).

###### Mesosoma.

Length of mesosoma 1.5 times its height; pronotum reticulate-rugose ventrally, setose and finely punctate dorsally; area near lateral carina of mesoscutum crenulate; mesoscutum dull, with irregular punctures and setae; notauli completely crenulate, united posteriorly forming a groove near scutellar sulcus; scutellar sulcus with 4 carinae (Fig. 4); scutellum distinctly convex with sparse fine punctures; precoxal sulcus short, similar to a wide groove; mesopleuron largely smooth with sparse fine punctures anteriorly; propodeum rugose.

**Figure 1.**
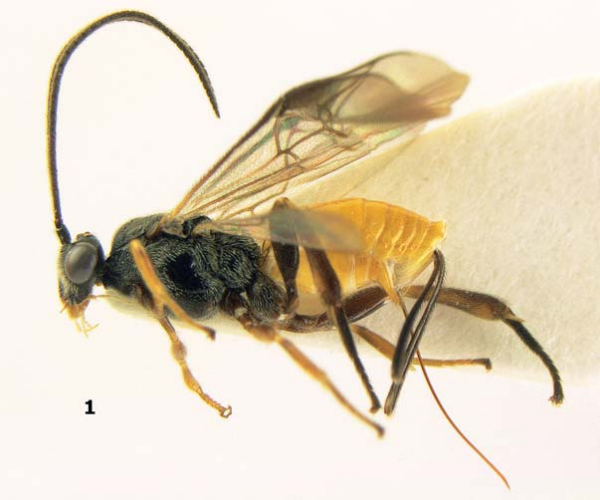
Agathis citrinisoma sp. n., female, holotype. Habitus lateral.

**Figures 2–10.**
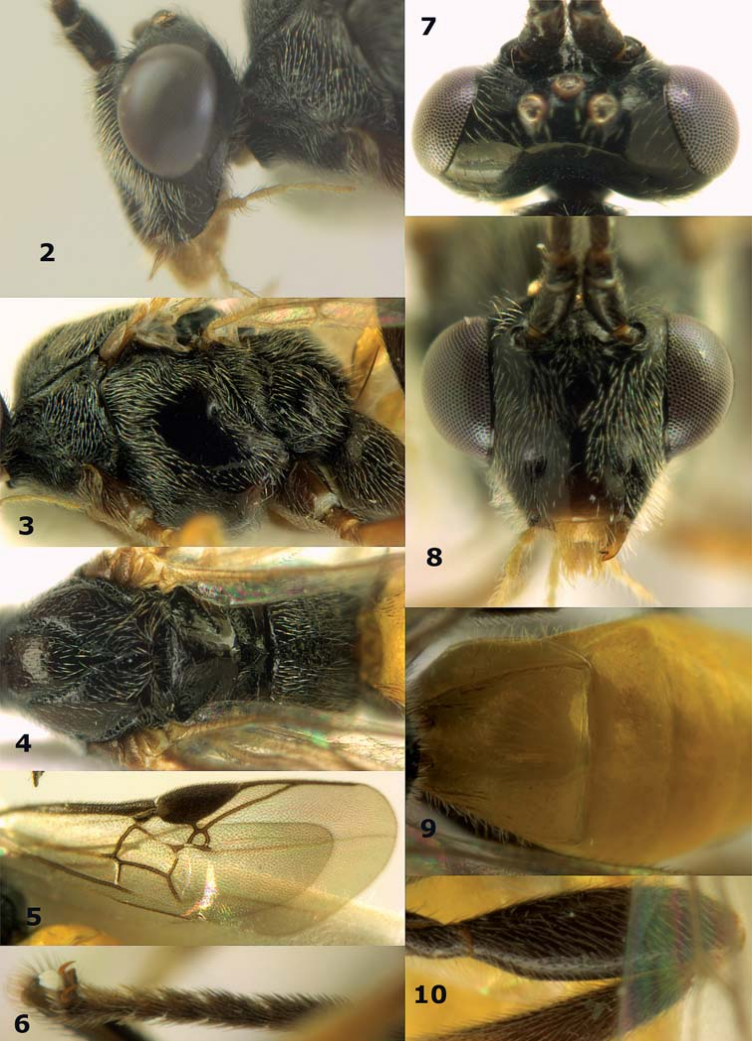
Agathis citrinisoma sp. n., female, holotype. **2** head lateral **3** mesosoma lateral **4** mesosoma dorsal **5** fore wing **6** hind tarsal claw **7** head dorsal **8** head anterior **9** first-third metasomal tergites dorsal **10** hind femur lateral.

###### Wings.

Fore wing: second submarginal cell rectangular (Fig. 5); vein 1-SR+M partly developed (Fig. 5); vein SR1 straight; r:3-SR+SR1 = 3:49; vein cu-a distinctly postfurcal. Hind wing: vein M+CU 1.3 times as long as vein 1-M.

###### Legs.

Length of hind femur, tibia and basitarsus 2.8, 4.7 and 6.5 times their width, respectively; hind femur (as remainder of legs) with short setae; length of outer and inner spur of middle tibia 0.3 and 0.4 times middle basitarsus, respectively; outer side of middle tibia with 2 pegs, apex with 2 pegs; length of outer and inner spur of hind tibia 0.3 and 0.5 times hind basitarsus; tarsal claws with lobe (Fig. 6).

###### Metasoma.

First tergite short, widened apically, as long as wide apically, superficially striate and partly smooth (Fig. 9), its dorsal carinae short, only basally distinct; second metasomal suture weakly impressed (Fig. 9); ovipositor sheath 0.5 times as long as fore wing, somewhat widened (Fig. 1).

###### Colour.

Black; palpi pale yellow; clypeus, galea, fore leg, middle leg (but coxa brown and femur yellowish-brown) and metasoma yellow; pterostigma and veins dark brown; wing membrane rather infuscate.

##### Distribution.

S Vietnam: Dak Lak.

##### Biology.

Unknown.

##### Etymology.

From “citrinus” (Latin for “of citron”) and “soma” (Greek for “body”), because of the yellow metasoma.

### Genus Bassus

Fabricius, 1804 s.s.

Hemiogaster Enderlein 1920, **syn. n.** The type species, Hemiogaster subrasa Enderlein, 1920, from Sumatra is a new combination in Bassus Fabricius.

#### Notes.

**Figures 11–15.**
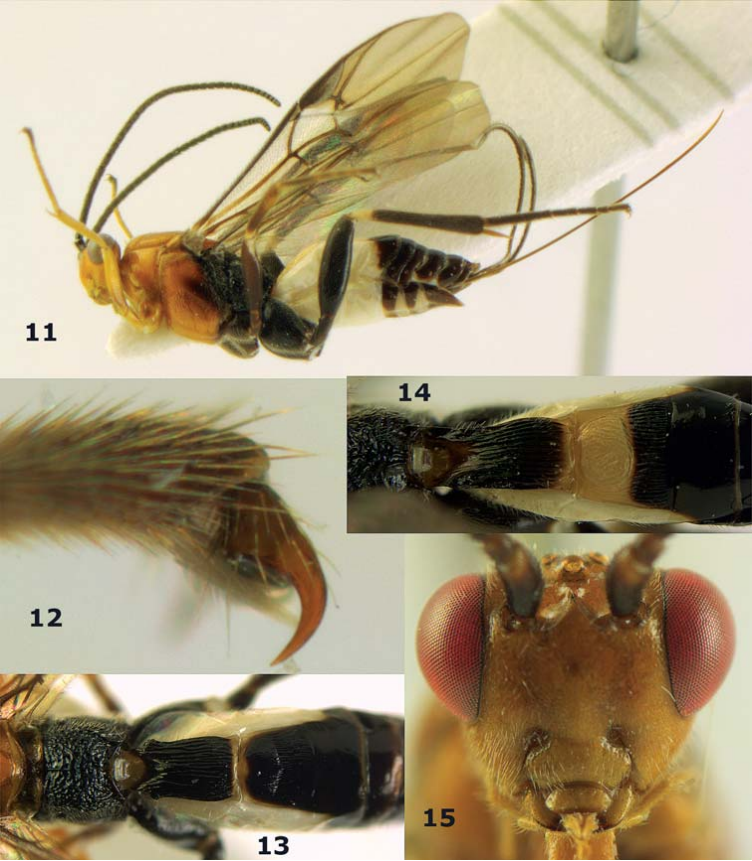
Bassus albifasciatus (Watanabe), female, Cuc Phuong National Park, but 13 of dark female from Cat Tien National Park. **11** habitus lateral **12** hind tarsal claw **13**, **14** first-third metasomal tergites dorsal **15** head anterior.

**Figure 16.**
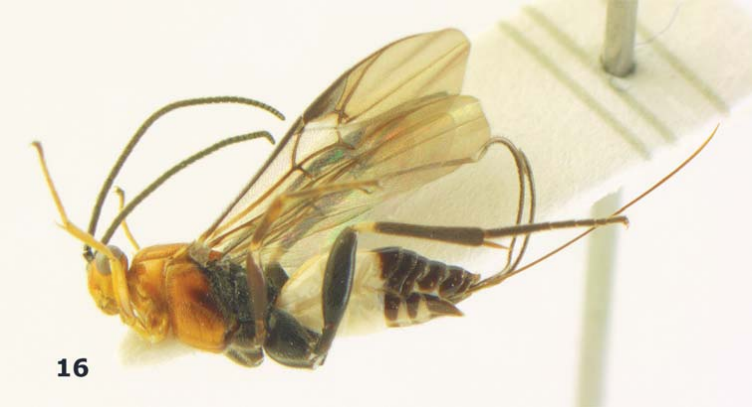
Bassus albobasalis sp. n., female, holotype. Habitus lateral.

Used in the strict sense for the species with simple tarsal claws, as was proposed by Sharkey et al. (2009), excluding the species now in Camptothlipsis Enderlein, Lytopylus Foerster and Therophilus Wesmael. The Vietnamese species have the basal half of the second metasomal tergite ivory and the hind tibia black with a pale basal ring (Figs 11, 16, 26). The type species of the genus Hemiogaster Enderlein, 1920, has been examined and proved to belong to Bassus Fabricius s.s. as currently used (Sharkey et al. 2009).

#### Key to Vietnamese species of the genus Bassus Fabricius

**Table:** 

1.	First tergite and basal half of second tergite ivory (Fig. 19); length of first metasomal tergite about 1.4 times as long as wide apically	Bassus albobasalis sp. n.
–	First tergite largely black or dark brown (Figs 13, 14, 29); length of first tergite 1.5–2.0 times as long as wide apically	2
2.	Dorsal carinae of first metasomal tergite largely obsolescent medially and posteriorly (Figs 13, 14); propodeum black; notauli narrowly crenulate; mesoscutum uneven medio-posteriorly; hind tibial spurs brown and weakly contrasting with blackish hind basitarsus	Bassus albifasciatus (Watanabe, 1934)
–	Dorsal carinae of first tergite costate medially and posteriorly nearly complete (Fig. 29); propodeum (except more or less posteriorly) brownish-yellow; notauli moderately widely crenulate (Fig. 28); mesoscutum flattened medio-posteriorly (Fig. 28); hind tibial spurs pale yellowish and distinctly contrasting with blackish hind basitarsus	Bassus albozonatus sp. n.

#### Bassus albifasciatus

(Watanabe, 1934)

Figs 11–15 

##### Distribution.

NE Vietnam: Ha Giang, Ninh Binh and S Vietnam: Dong Nai. New record. Outside Vietnam known from China (Hubei; Ningxia; Taiwan), Japan (Okinawa) and Korea.

#### Bassus albobasalis sp. n.

urn:lsid:zoobank.org:act:F36AD4C6-FB86-4581-B52B-2DE9BDB1DF45

Figs 16 –25 

##### Type material.

Holotype, ♀ (RMNH) “S. Vietnam: Dak Lak, Chu Yang Sin N.P., nr dam, 800–1000 m, 2–10.vi.2007, Mal. traps 9–11, C. v. Achterberg & R. de Vries, RMNH’07”. Paratypes (7 ♀): 1 ♀ (RMNH), id., but 800–940 m; 5 ♀ (RMNH, IEBR) “S. Vietnam: Dak Lak, Chu Yang Sin N.P., Krong K’Mar, Mal. traps, 840–940 m or 740–900 m, 2–10.vi.2007, C. van Achterberg & R. de Vries, RMNH’07”; 1 ♀ (RMNH) “N. Vietnam: Thua Thien-Hue, Phong Dien N.R. n[ea]r base-camp, 15 km W Phong My, c 60 m, 22.iii-6.iv.2001, Mal. traps 1–3, C. v. Achterberg & R. de Vries, RMNH’01”.

##### Diagnosis.

The new species is similar to Bassus lineaticollis (Cameron, 1910) from Sri Lanka, but has the scutellum sparsely finely punctate (in Bassus lineaticollis densely rugulose-punctate) and the first tergite 1.4 times (twice) as long as its apical width. The new species is also similar to Bassus cancellatus (Enderlein, 1920) from China (Taiwan), but differs by having the first and second tergites ivory (black), the body smaller (3–5 mm versus 6–10 mm), fewer antennal segments (29–31 versus 42–46), the first tergite (1.4 times versus 1.7–1.8 times) and the ovipositor sheath shorter (0.7 times versus 1.1 times as long as fore wing). Bassus subrasa (Enderlein, 1920) comb. n. from Indonesia (Sumatra) is similar but has the head dorsally, propodeum, hind coxa and femur brownish-yellow, first and second tergites coarsely striate and the eye about 3 times longer than the temple. Bassus canaliculatus Yang & Chen, 2006, from China (Hubei) has the ovipositor sheath about 1.3 times as long as the fore wing, the dorsal carinae of the first tergite lamelliform and nearly complete, the first tergite with a medio-longitudinal depression and both basal tergites of the metasoma black.

##### Description.

Holotype, ♀, length of body 5.2 mm, of fore wing 4.6 mm, of ovipositor sheath 3.3 mm.

###### Head.

Antennal segments 31, length of third segment 1.3 times fourth segment; third, fourth and penultimate segments 3.6, 3.3 and 1.4 times their width, respectively; length of apical segment 1.2 times as long as penultimate segment; length of maxillary palp 0.7 times height of head; malar space 2.7 times as long as basal width of mandible; temple short (Fig. 24), in dorsal view length of eye 4.6 times temple; ocelli in low triangle, POL:OD:OOL = 9:7:18 (Fig. 24); face with distinct fine punctures; frons shiny, smooth; vertex and temple shiny with sparse minute punctures (Fig. 24).

###### Mesosoma.

**Figures 17–25.**
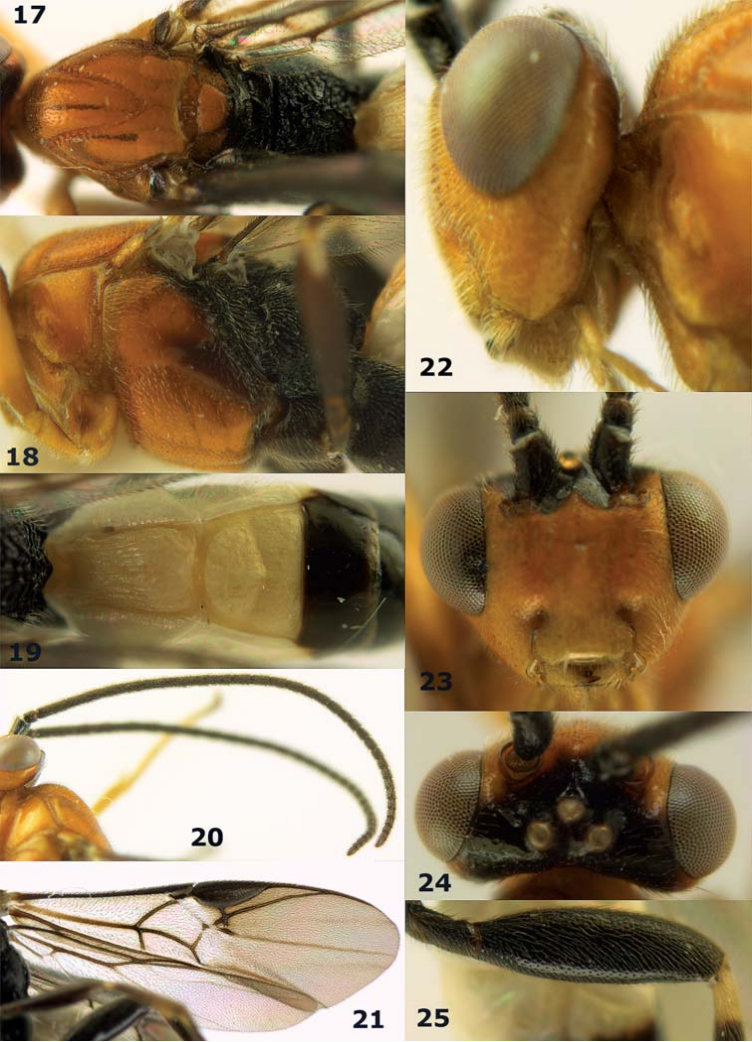
Bassus albobasalis sp. n., female, holotype. **17** mesosoma dorsal **18** mesosoma lateral **19** first-third metasomal tergites dorsal **20** antenna **21** wings **22** head lateral **23** head anterior **24** head dorsal **25** hind femur lateral.

Length of mesosoma 1.5 times its height; subpronope shallow; pronotum largely smooth laterally, with sparse fine punctures dorsally; area near lateral carina of mesoscutumsparsely crenulate; lateral and middle lobes of mesoscutum sparsely and distinctly punctate, flat and smooth posteriorly; notauli complete, moderately crenulate anteriorly and narrowly crenulate posteriorly; scutellar sulcus 0.4 times as long as dorsal face of scutellum and with 4 carinae; scutellum slightly convex and distinctly narrowed with lateral carina, shiny with sparse fine punctures, subposterior crest curved (Fig. 17); precoxal sulcus rather deep, moderately crenulate (Fig. 18); mesopleuron below precoxal sulcus with sparse fine punctures; mesopleuron above precoxal sulcus largely smooth; metapleuron with large sparse punctures; propodeum closely reticulate-rugose; propodeal spiracle small, as long as wide.

###### Wings.

Fore wing: second submarginal cell triangular (Fig. 21); vein SR1 straight; r:3-SR+SR1 = 6:63. Hind wing: vein M+CU 0.6 times as long as vein 1-M (Fig. 21).

###### Legs.

Length of hind femur, tibia and basitarsus 3.2, 6.8 and 10.0 times their width, respectively; hind femur (as remainder of legs) with bristly setae; length of outer and inner spur of middle tibia 0.3 and 0.5 times middle basitarsus, respectively; apex of outer side of hind tibia with a cluster of 8 pegs; length of outer and inner spur of hind tibia 0.3 and 0.5 times hind basitarsus, respectively; tarsal claws without lobe (cf. Fig. 12).

###### Metasoma.

Length of first tergite 1.4 times its apical width (Fig. 19); first tergite sparsely and rather coarsely longitudinally striate, dorsal carinae nearly complete; second tergite largely smooth, but transverse groove and area behind it striate; remainder of metasoma smooth (Fig. 19); ovipositor sheath 0.7 times as long as fore wing, slender (Fig. 16).

###### Colour.

Orange brown; mouthparts yellow; antenna, frons and vertex dark brown; propodeum, third-eighth metasomal segments, hind coxa, trochantellus and femur black; hind tibial spurs yellow; first and second tergites ivory dorsally and white ventrally; hind tibia and tarsus dark brown, except yellow basal ring; veins and pterostigma dark brown, but pterostigma basally narrowly pale brownish; apical 0.4 of fore wing distinctly infuscate and remainder of wings slightly infuscate or subhyaline.

###### Variation.

Antennal segments 29–31; second submarginal cell of fore wing with vein r-m absent or present; vein M+CU of hind wing 0.6–0.8 times as long as vein 1-M; second tergite with smooth or striate transverse groove; apical segment of antenna 1.0–1.2 times as long as penultimate segment; length of body 3.2–5.2 mm.

##### Distribution.

C Vietnam: Thua Thien-Hue and S Vietnam: Dak Lak.

##### Biology.

Unknown.

##### Etymology.

From “albus” (Latin for “white”) and “basis” (Latin for “foundation, base”), because of the white base of the metasoma.

#### Bassus albozonatus sp. n.

urn:lsid:zoobank.org:act:7E9F1682-3C25-40AA-8CEE-50C371E2D051

Figs 26 –35 

##### Type material.

Holotype, ♂ (RMNH), Aga. 282, “NE Vietnam: Ninh Binh, Cuc Phuong N.P., MT, 20°23'N; 105°34'E, 5–10.v.2002, K.D. Long” . Paratypes: 1 ♂ (IEBR), Aga. 283, same data as holotype; 1 ♂ (RMNH), Aga. 234, “S. Vietnam: Dac Lak, Cu N’Ga forest, 10.vi.2005. K.D. Long”.

##### Diagnosis.

The new species is similar to Bassus albifasciatus (Watanabe, 1934), but differs by having the malar space twice (2.6–2.8 times in Bassus albifasciatus) as long as basal width of mandible, the precoxal sulcus short and shallow (distinct and 0.8 times as long as mesopleuron), the propodeum with three complete apical carinae (evenly reticulate-rugose) and (except more or less posteriorly) brownish-yellow (brown to black), the notauli more widely crenulate (rather narrowly crenulate), the mesoscutum flattened medio-posteriorly (rather convex) and the hind tibial spurs pale yellowish and distinctly contrasting with the blackish hind basitarsus (brownish and less contrasting). Because of the colour of the head and of the first tergite the new species is similar to Bassus subrasa (Enderlein, 1920) comb. n. from Indonesia. However, the latter has the eye about 3 times as long as the temple (2.3 times in Bassus albozonatus), the second tergite distinctly costate (only partly striate), the notauli smooth (finely crenulate) and vein cu-a of the fore wing distinctly postfurcal (interstitial).

##### Description.

Holotype, ♂, length of body 6.5 mm, of fore wing 6.0 mm.

###### Head.

Antennal segments 36, length of third segment 1.1 times fourth segment, length of third, fourth and penultimate segments 4.4, 4.0 and 2.3 times their width, respectively; length of apical antennal segment 1.6 times as long as penultimate segment; maxillary palp 0.7 times height of head; malar space twice as long as basal width of mandible; in dorsal view length of eye 2.3 times temple; ocelli in low triangle, POL:OD:OOL = 8:7:21 (Fig. 34); face shiny, with distinct punctures; frons with a medial ridge, smooth (Fig. 34); vertex and temple shiny, with sparse minute punctures.

###### Mesosoma.

Length of mesosoma 1.4 times its height; subpronope shallow; pronotum largely smooth with sparse fine punctures dorsally; area near lateral carina of mesoscutum sparsely crenulate; lateral lobes of mesoscutum shiny with fine punctures anteriorly, flat and smooth posteriorly; middle lobe of mesoscutum with sparse punctures and largely smooth posteriorly (Fig. 28); notauli complete and finely crenulate; scutellar sulcus 0.4 times as long as dorsal face of scutellum and with 3 carinae; scutellum convex, rather long and narrowed posteriorly, without subposterior crest (Fig. 28); precoxal sulcus short, shallow and rugose (Fig. 27); mesopleuron below precoxal sulcus with sparse distinct punctures; mesopleuron above precoxal sulcus largely smooth, but with sparse fine punctures posteriorly; metapleuron largely punctate anteriorly with irregular rugosity posteriorly; propodeum with three complete apical carinae and enclosing a large reticulate-rugose area (Fig. 28); propodeal spiracle rather small elliptical, 1.5 times as long as wide.

###### Wings.

Fore wing: second submarginal cell small trapezoid (Fig. 30); vein SR1 sinuate; r:3-SR+SR1 = 4:61. Hind wing: vein M+CU 0.7 times as long as vein 1-M (Fig. 30).

###### Legs.

Length of hind femur, tibia and basitarsus 3.9, 7.5 and 10.4 times their width, respectively; hind femur (as remainder of legs) with short setae; length of outer and inner spur of middle tibia 0.4 and 0.5 times middle basitarsus, respectively; outer apex of middle tibia with a cluster of 10 pegs; outer apex of hind tibia with a cluster of 12 pegs; length of outer and inner spur of hind tibia 0.4 and 0.5 times hind basitarsus, respectively; outer side of hind coxa with sparse punctures, of hind femur with distinct punctures; tarsal claws without lobe (Fig. 35).

###### Metasoma.

Length of first tergite 1.5 times its apical width, with dorsal and dorso-lateral carinae coarsely developed, dorsal carinae convergent, costate medially and posteriorly nearly up to apex of tergite (Fig. 29); first tergite sparsely but coarsely striate; second tergite 1.4 times as long as third tergite, large basal area on two thirds of tergite partly smooth, rugose-punctate and remainder densely striate (Fig. 29); remainder of metasoma smooth.

###### Colour.

Brownish-yellow; antenna (but scapus yellow) brown; hind leg (but tibia with pale yellow basal ring) and metasoma dark brown or black (but basal area of second tergite ivory and first and second tergites white ventrally); pterostigma (except small pale brownish patch basally) and veins dark brown; wing membrane slightly infuscate or subhyaline, but apical 0.4 of fore wing rather infuscate.

###### Variation.

Length of body 6.5–7.0 mm, of fore wing 6.0–6.1 mm; penultimate antennal segment subequal to apical segment; vein M+CU of hind wing 0.7–0.8 times as long as 1-M; POL:OD:OOL = 8:6–7:21; outer apex of hind tibia with 9–12 pegs.

##### Distribution.

NE Vietnam: Ninh Binh and S Vietnam: Dak Lak.

**Figure 26.**
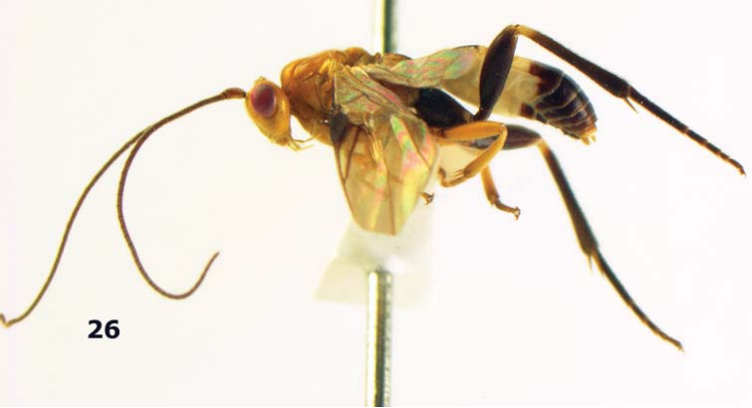
Bassus albozonatus sp. n., male, holotype. Habitus lateral.

**Figures 27–35.**
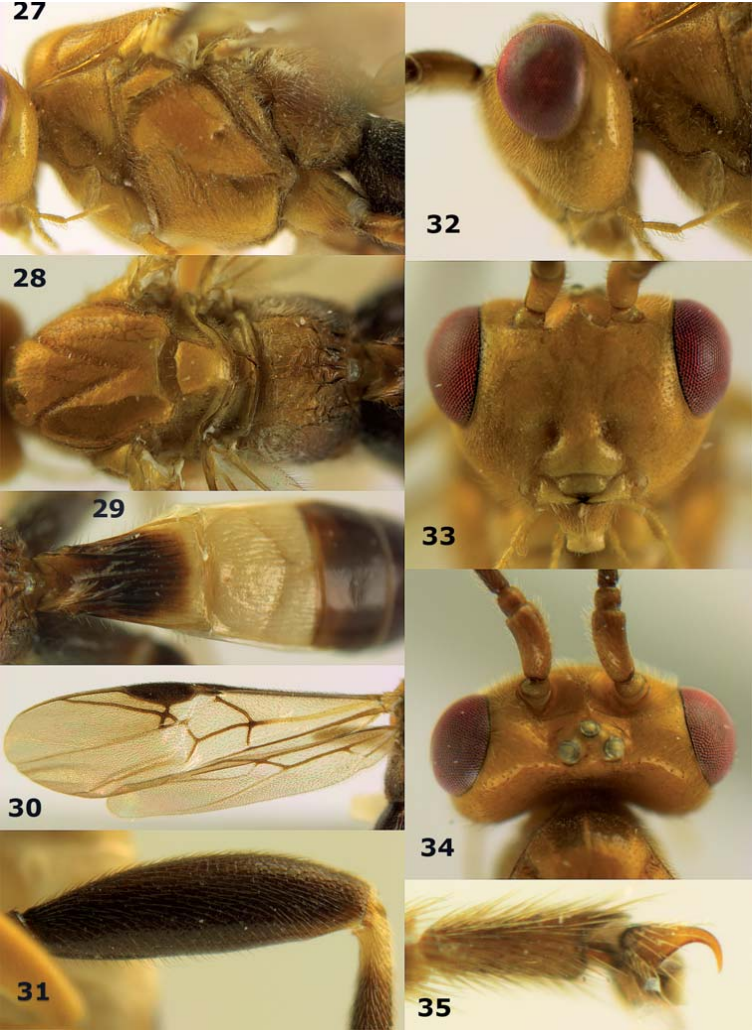
Bassus albozonatus sp. n., male, holotype. **27** mesosoma lateral **28** mesosoma dorsal **29** first-third metasomal tergites dorsal **30** wings **31** hind femur lateral **32** head lateral **33** head anterior **34** head dorsal **35** hind tarsal claw.

##### Biology.

Unknown.

##### Etymology.

From “albus” (Latin for “white”) and “zona” (Latin for “girdle”), because of the white part of the second metasomal tergite.

### Genus Biroia

Szépligeti, 1900

#### Biroia soror sp. n.

urn:lsid:zoobank.org:act:C7FE6AB0-4C62-4E25-85A1-8809847BC928

Figs 36 –43 

##### Type material.

Holotype, ♀ (RMNH), “S. Vietnam: Dong Nai, Cat Tien N. P., Dong trail, Mal. traps 13–16, c 100 m, 1–9.x.2005, C. v. Achterberg & R. de Vries, RMNH’05”. Paratypes (6 ♀): 2 ♀ (RMNH, IEBR), id. but Ficus trail, Mal. traps 1–8; 1 ♀ (RMNH), id. but bird trail, Mal. traps 9–12; 3 ♀ (RMNH, IEBR), id. but Ficus trail, 9–10.iv.2007, M.P. Quy & N.T. Manh.

##### Diagnosis.

**Figure 36.**
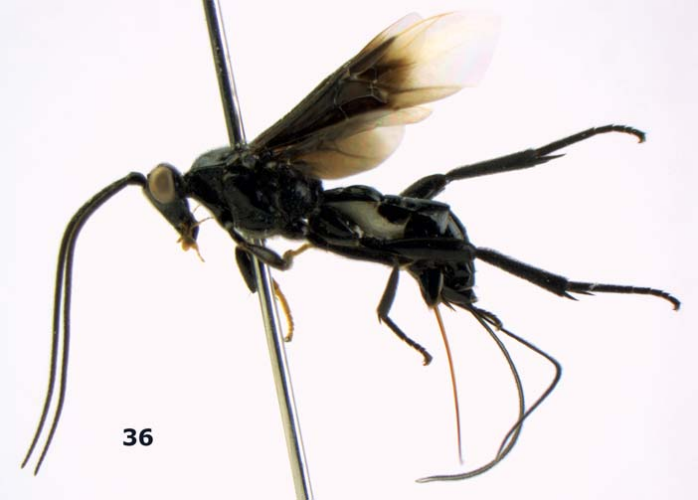
Biroia soror sp. n., female, holotype. Habitus lateral.

Morphologically similar to Bassus abdominalis (Enderlein, 1920) from Sundaland; the new species differs by the medium-sized prepectal carina (distinctly lamelliform in Bassus abdominalis, rarely intermediate), the less concave head medio-posteriorly (Fig. 41; more concave in Bassus abdominalis), the shorter setae of the fore tarsus (with longer setae), the somewhat shorter postero-ventral protuberance of the pronotal side (above the fore coxa; somewhat longer in Bassus abdominalis) and the completely black body (Fig. 36; the latter occurs rarely also in Bassus abdominalis, but often the head and the anterior part of the mesosoma are orange-brown).

##### Description.

Holotype, ♀, length of body 8.8 mm, of fore wing 7.2 mm, ovipositor sheath 6.3 mm.

###### Head.

Antennal segments 45, length of third segment 1.5 times fourth segment, length of third, fourth and penultimate segments 1.7, 1.7 and 1.7 times their width, respectively; apical antennal segment 1.6 times as long as penultimate segment; length of maxillary palp 0.5 times height of head; in dorsal view length of eye 4.7 times temple; temple gradually narrowed posteriorly (Fig. 41); POL:OD:OOL = 8:5:15; face elongate (Fig. 38), with dense and fine punctation laterally, shiny and sparsely punctate medially; frons smooth, two strong lateral carinae extending up near rim of antennal sockets (Fig. 41); vertex and temple with sparse fine punctures.

###### Mesosoma.

**Figures 37–43.**
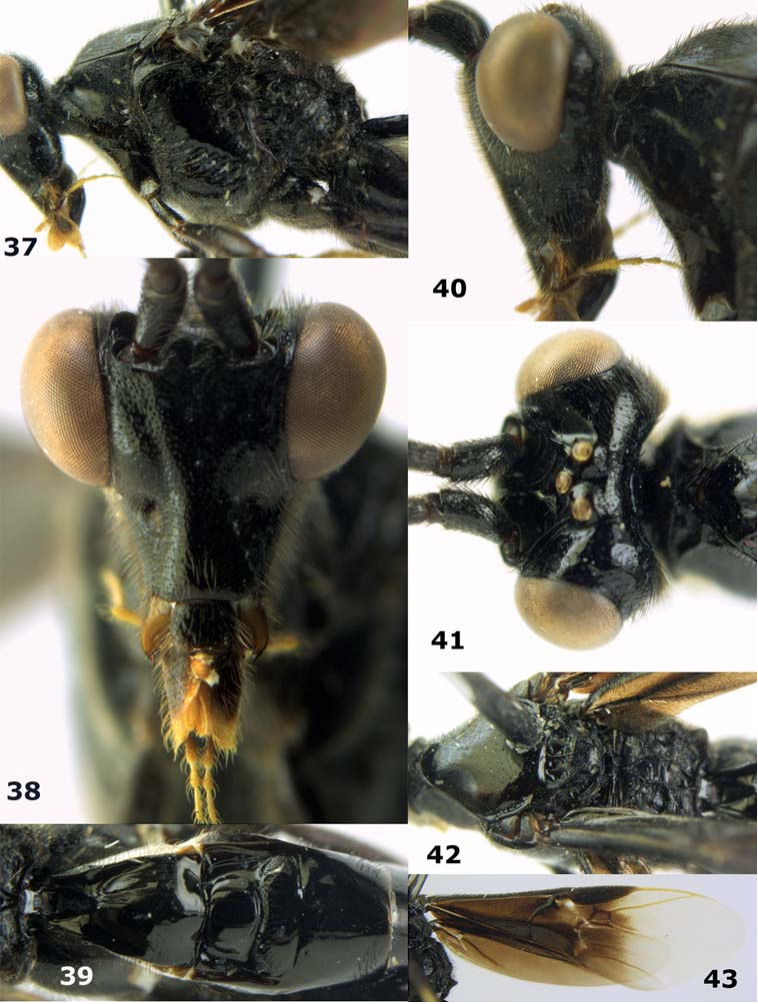
Biroia soror sp. n., female, holotype. **37** mesosoma lateral **38** head anterior **39** first-third metasomal tergites dorsal **40** head lateral **41** head dorsal **42** mesosoma dorsal **43** wings.

Length of mesosoma 1.6 times its height; pronotal trough smooth medially, with sparse fine punctures dorsally and crenulate posteriorly; area near lateral carina of mesoscutum smooth anteriorly, crenulate posteriorly; mesoscutum shiny with very sparse minute punctures; notauli completely absent; scutellar sulcus 0.5 times as long as dorsal face of scutellum and with 3 strong carinae (Fig. 42); precoxal sulcus wide, largely crenulate, three long carinae connected with prepectal carina anteriorly (Fig. 37); mesopleuron above precoxal sulcus shiny and nearly smooth with very sparse fine punctures; mesopleuron below precoxal sulcus with sparse distinct punctures; metapleuron setose with sparse distinct punctures dorsally, largely areolate-rugose ventrally; propodeum with a large areola and costulae developed, area of areola with 3 transverse carinae; propodeal spiracle large medium-sided, 1.75 times as long as wide.

###### Wings.

Fore wing: second submarginal cell wide rectangular (Fig. 43); vein SR1 straight; r:3-SR:SR1 = 4:10:64; r:2-SR:3-SR:r-m = 4:11:10:8. Hind wing: vein M+CU 0.6 times as long as vein 1-M.

###### Legs.

Length of hind femur, tibia and basitarsus 4.0, 6.0 and 9.3 times their width, respectively; hind femur (as remainder of legs) with strong setae; length of outer and inner spur of middle tibia 0.4 and 0.7 times middle basitarsus, respectively; length of outer and inner spur of hind tibia 0.3 and 0.5 times hind basitarsus; fore and middle tarsi slender (Fig. 36).

###### Metasoma.

Shiny smooth; first tergite distinctly widened subposteriorly and then narrowed apically; length of first tergite 1.4 times as long as its apical width (Fig. 39); dorsal carinae absent; first tergite with long erect setae laterally (Fig. 39), apical third of first tergite with transverse a row of sparse setae; second tergite with large basal round area bordered by a groove (Fig. 39), area near groove with two rows of sparse setae; latero-posterior corners of third tergite with a dense cluster of setae; ovipositor sheath 0.9 times as long as fore wing.

###### Colour.

Black; galea, palpi, mandible, fore legs and tarsus yellow; wing membrane black but hyaline on apical third of fore wing and on one fourth of hind wing.

###### Variation.

Length of body 8.5–10.0 mm, and of fore wing 6.8–8.0 mm; ratio of vein r:3-SR:SR1 = 3–4:11–12:71–82; vein M+CU of hind wing 0.5–0.7 times as long as vein 1-M; first tergite 1.4–1.7 times as long as its apical width.

##### Distribution.

S Vietnam: Dong Nai.

##### Biology.

Unknown.

##### Etymology.

From “soror” (Latin for “sister”), because of its close similarity to Bassus abdominalis (Enderlein).

### Genus Braunsia

Kriechbaumer, 1894

#### Key to Vietnamese species of the genus Braunsia Kriechbaumer

**Table:** 

1.	Fore wing with isolated stigmal spot or nearly so (Figs 54, 58, 67), vein cu-a of fore wing postfurcal or interstitial (Figs 58, 67); area near vein cu-a of hind wing setose; propodeum with an anterior transverse carina and usually with an areola; first tergite moderately robust (Figs 57, 66, 76) and longitudinally striate medially; ovipositor sheath slightly or not widened	2
–	Fore wing without a stigmal spot (Figs 44, 48, 84), vein cu-a of fore wing antefurcal (Fig. 84), but sometimes interstitial; area near vein cu-a of hind wing glabrous; propodeum without an anterior transverse carina or an areola; first tergite very slender (Figs 47, 85) and smooth medially; ovipositor sheath ribbon-shaped widened (Fig. 44)	4
2.	Vein 2-SR+M of hind wing distinctly transverse (or longitudinal); propodeum with a rather weakly developed transverse carina anteriorly (Fig. 65); lateral ocelli small (Fig. 71), POL 0.7 times as long as OOL; vein cu-a of fore wing interstitial (Fig. 67)	Braunsia maculifera sp. n. Notes. If POL 0.5 times OOL and vein cu-a of fore wing distinctly postfurcal, cf. Braunsia pappi Chen & Yang, 2006, from China. If the areola of the propodeum is wider, the apical quarter of the hind tibia infuscate and the hind tarsus blackish, the anterior transverse carina is incomplete, the antenna is yellowish, the pterostigma is dark brown except for the yellow basal third, the frons deeply concave near the antennal sockets, no isolated stigmal spot of the fore wing, the ovipositor sheath about as long as body and the first tergite is more robust, cf. Braunsia margaroniae Nixon, 1950, from India.
–	Vein 2-SR+M of hind wing vertical or near so (Fig. 75); propodeum with a coarse transverse carina basally (Figs 56, 74); lateral ocelli larger (Figs 62, 80), POL 0.4–0.5 times as long as OOL; vein cu-a of fore wing postfurcal (Fig. 58)	3
3.	Propodeum without a closed areola (Fig. 74); basal half of first tergite smooth; area behind transverse groove of third tergite and second tergite (except smooth apical small area) equally longitudinally striate; vein 2-SR+M of hind wing distinctly vertical (Fig. 75); apical half of wings brown and basal half yellow (Fig. 75); metasoma dark brown (but first tergite yellow basally and three basal metasomal segments ivory ventrally)	Braunsia nigrapiculata sp. n.
–	Propodeum with areola (Fig. 56); basal half of first tergite striate; area behind transverse groove on third tergite smooth, contrasting with striate second tergite; vein 2-SR+M of hind wing short and thick, slightly vertical (Fig. 58); wings and metasoma entirely yellow (Figs 54, 58)	Braunsia devriesi sp. n. Notes. Braunsia pappi Chen & Yang, 2006, from China differs from Braunsia devriesi by having the fore wing darkened apically with a distinct dark brown band below the stigmal spot and by the smooth basal half of the first metasomal tergite.
4.	Lateral ocelli small (Fig. 52), POL 0.8 times as long as OOL; metasoma yellowish-brown (Fig. 44); outer side of apical third of middle tibia with row of 5 pegs; eye medium-sized, in dorsal view length of eye 2.1 times as long as temple (Fig. 52); malar space 1.8 times as long as basal width of mandible	Braunsia bicolorata sp. n.
–	Lateral ocelli larger (Fig. 88), POL 0.5–0.6 times as long as OOL; metasoma mainly blackish-brown (Fig. 81); outer side of apical third of middle tibia with row of 4–6 pegs; eye rather large, in dorsal view length of eye 2.7 times as long as temple (Fig. 88); malar space 1.4 times as long as basal width of mandible	Braunsia pumatica sp. n.

#### Braunsia bicolorata sp. n.

urn:lsid:zoobank.org:act:4951D986-C862-46D2-B8C2-1D1E24C4A657

Figs 44 –53 

##### Type material.

Holotype, ♀ (RMNH), Aga. 119, “NE Vietnam: Hoa Binh, Mai Chau, Pa Co N.R, 1200 m, 24.iv.2002, K.D. Long”. Paratypes: 1 ♂ (IEBR), Aga. 120 and 1 ♂ (RMNH), Aga. 121, same data as holotype; 1 ♀ (IEBR), Aga. 276, id., but 21.iv.2002, K.D. Long; 2 ♂ (IEBR), Aga. 216, Aga. 219 and 1 ♂ (RMNH), Aga. 220, “N.W Vietnam: Lao Cai, Sa Pa, bushes, 8.x.2004, K.D. Long”.

##### Diagnosis.

The new species is morphologically similar to Braunsia latisocreata Bhat & Gupta, 1977, from India, but differs by having the first tergite about 3.7 times as long as its apical width (in Braunsia latisocreata 1.5 times); the second tergite about twice as long as wide apically (1.5 times); the second submarginal cell of the fore wing with a rather long (i.e. distinctly longer than vein r of fore wing) ramellus (short) and the metasoma entirely reddish yellow (black).

##### Description.

Holotype, ♀, length of body 9.0 mm, of fore wing 7.8 mm, ovipositor 8.0 mm.

###### Head.

Antennal segments 47, length of third segment 1.2 times fourth segment, length of third, fourth and penultimate segments 3.7, 3.3 and 2.0 times their width, respectively; length of maxillary palp 0.7 times height of head; in dorsal view head transverse and 1.3 times as wide as mesonotum; length of eye 2.1 times temple (Fig. 52); POL:OD:OOL = 7:4:11; antennal sockets not tubular; occipital flange sharp; malar space 1.8 times as long as basal width of mandible; face shiny with sparse fine punctures, frons and vertex smooth.

###### Mesosoma.

Length of mesosoma 1.5 times its height; subpronope large and deep; side of pronotum smooth; area near lateral carina of mesoscutum crenulate; lateral lobes of mesoscutum smooth; middle lobe with sparse fine punctures; notauli deep, crenulate (Fig. 46); scutellar sulcus 0.5 times as long as dorsal face of scutellum and with 3 carinae; scutellum convex anteriorly, smooth and with long setae; mesopleuron above precoxal sulcus largely smooth; mesopleuron below precoxal sulcus setose, with sparse fine punctures; precoxal sulcus wide, shallow and slightly crenulate (Fig. 45); metapleuron mainly smooth with long setae; propodeum setose, with a strong transverse carina subbasally, rugose posteriorly; spiracle medium-sized, round, 1.8 times as long as wide.

###### Wings.

Fore wing: second submarginal cell pentagonal, narrow anteriorly, with rather long ramellus, 1.1 times as long as vein 2-SR (17:15) (Fig. 48); r:3-SR:SR1 = 4:3:65; 2-SR:3-SR:r-m = 11:3:11; vein cu-a slightly antefurcal. Hind wing: vein 2-SR+M transverse; vein M+CU 0.6 times as long as 1-M (28:50); surroundings of cu-a glabrous.

###### Legs.

Length of hind femur, tibia and basitarsus 5.7, 10.0 and 10.6 times their width, respectively; hind coxa smooth; hind femur with short and sparse setosity (Fig. 49); outer side of apical third of middle tibia with a row of 5 pegs; outer side of apex of hind tibia with a cluster of 8 pegs; length of outer and inner spurs of middle tibia 0.4 and 0.5 times middle basitarsus, respectively; length of outer and inner spurs of hind tibia 0.3 and 0.4 times hind basitarsus.

###### Metasoma.

First tergite slender shiny, rugulose near apex, slightly and roundly widened apically (Fig. 47); length of first tergite 3.7 times its apical width; dorsal carinae of first tergite divergent and on three fourth of the tergite; second tergite 2.1 times as long as wide apically and with posteriorly diverging striae, on apical third of second tergite with transverse furrow; dorsal half of third tergite with striae, apical half finely granulate; remainder of metasoma smooth (Fig. 47), ovipositor sheath wide and ribbon-shaped (Fig. 50), 1.1 times as long as fore wing.

###### Colour.

Black; malar space ivory, clypeus and palpi pale yellow; antenna, legs and metasoma yellowish-brown, but tarsi paler than tibiae; wing membrane rather dark brown (Fig. 48).

###### Variation.

Female: second submarginal cell of fore wing triangular or pentagonal; vein M+CU of hind wing 0.5–0.6 times as long as 1-M; outer side of hind tibial apex with cluster of 8–10 pegs. Male: antenna with 45 or 48 segments; vein cu-a of fore wing interstitial; outer side of hind tibial apex with 7 pegs; hind coxa and first tergite apically dark brown.

##### Distribution.

NE Vietnam: Hoa Binh, NW Vietnam: Lao Cai.

**Figure 44.**
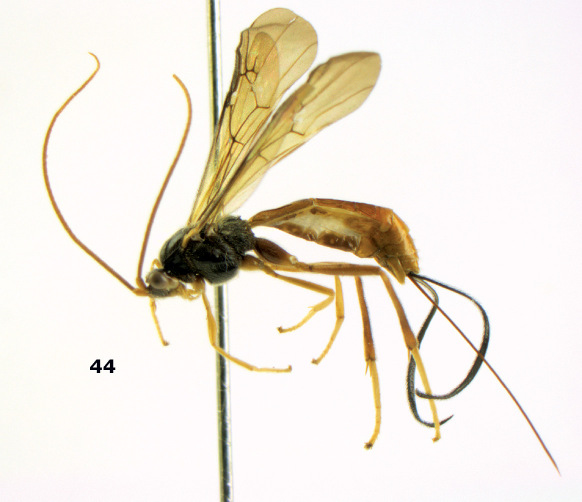
Braunsia bicolorata sp. n., female, holotype. Habitus lateral.

**Figures 45–53.**
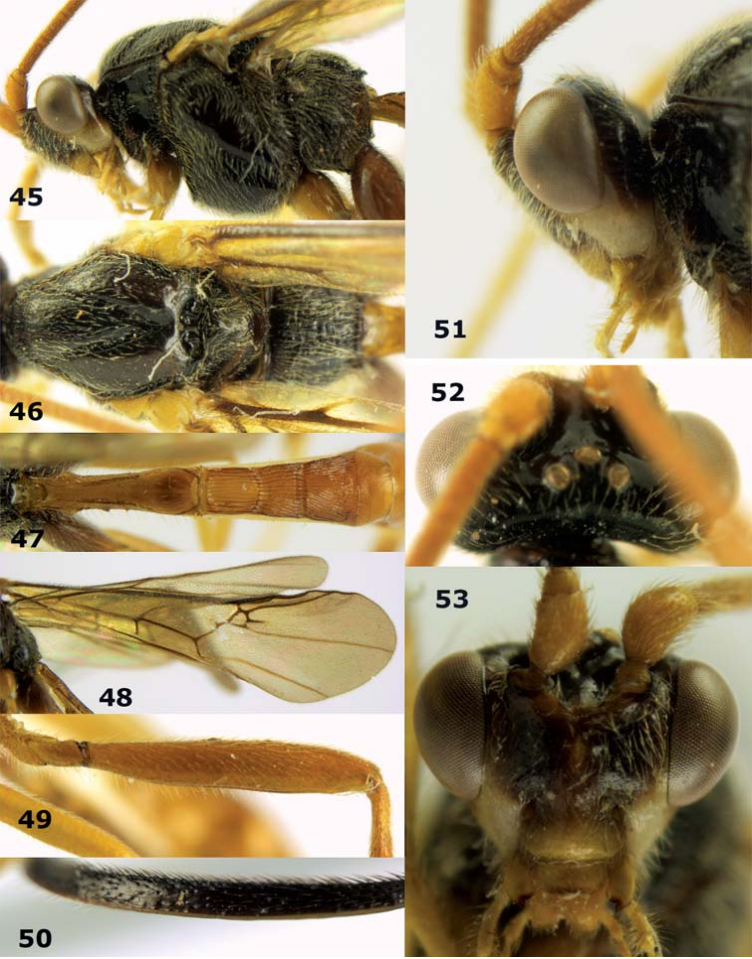
Braunsia bicolorata sp. n., female, holotype. **45** mesosoma lateral **46** mesosoma dorsal **47** first-third metasomal tergites dorsal **48** wings **49** hind femur lateral **50** ovipositor sheath lateral **51** head lateral **52** head dorsal **53** head anterior.

##### Biology.

Unknown.

##### Etymology.

From “bi” (Latin for “two”), and “coloris” (Latin for “hue, tint”), because of the bicoloured body.

#### Braunsia devriesi sp. n.

urn:lsid:zoobank.org:act:585C9AC9-B17A-4CE4-B250-A84DE8BF7E32

Figs 54 –62 

##### Type material.

Holotype, ♀ (RMNH), “N. Vietnam: Viet Tri, n[ea]r Thanh Son, Thuong Cuu, 20°59'N; 105°8'E, 350–400 m, 11–16.x.1999, Malaise traps, R. de Vries, RMNH’99”.

##### Diagnosis.

The new species is morphologically similar to Braunsia bipunctata Enderlein, 1906, from Indonesia, but differs by having the propodeum with a complete and regular basal transverse carina (Braunsia bipunctata: transverse carina partly weakly developed and irregular); and the anterior half of the first tergite coarsely striate medially (bipunctata: smooth except for a median carina).

##### Description.

Holotype, ♀, length of body 10.5 mm, of fore wing 9.3 mm, ovipositor 7.3 mm.

###### Head.

Antennal segments 45; length of third segment times fourth segment, length of third, fourth and penultimate segments 3.3, 2.3 and 1.3 times their width, respectively; length of maxillary palp 0.7 times height of head; in dorsal view length of eye twice temple (Fig. 62); POL:OD:OOL = 7:5:12; antennal sockets rather tubular (Fig. 62); occipital flange large, its ventral margin convex bellow (Fig. 60); face shiny smooth with sparse punctures; frons and vertex shiny and smooth.

###### Mesosoma.

Length of mesosoma 1.5 times its height; subpronope large and deep; side of pronotum smooth; area near lateral carina of mesoscutum smooth; side of mesoscutum largely smooth with sparse setae and fine punctures; notauli deep and smooth (Fig. 56); scutellar sulcus short, 0.4 times as long as dorsal face of scutellum and with 4 carinae; scutellum convex, smooth, sparsely setose; mesopleuron above and below precoxal sulcus shiny and smooth; precoxal sulcus narrow and shallow similar to a smooth groove (Fig. 55); metapleuron smooth; propodeum with basal and transverse carinae, two longitudinal carinae forming a large areola; spiracle medium-sized, subelliptical and 2.3 times as long as wide.

###### Wings.

Fore wing: second submarginal cell pentagonal, narrow anteriorly, with rather long ramellus, 0.8 times as long as vein 2-SR (Fig. 58); r:3-SR:SR1 = 6:2:55; 2-SR:3-SR:r-m = 9:2:9; vein cu-a distinctly postfurcal. Hind wing: vein 2-SR+M slightly vertical; vein M+CU 0.8 times as long as 1-M; surroundings of cu-a sparsely setose.

###### Legs.

Length of hind femur, tibia and basitarsus 5.1, 9.7 and 11.6 times their width, respectively; hind femur (as remainder of legs) with short and dense setosity; outer side of apical third of middle tibia with a row of 5 pegs and a cluster of 3 pegs at apex; outer side of apex of hind tibia with a cluster of 9 pegs; length of outer and inner spurs of middle tibia 0.4 and 0.6 times middle basitarsus, respectively; length of outer and inner spurs of hind tibia 0.3 and 0.4 times hind basitarsus, respectively.

###### Metasoma.

**Figure 54.**
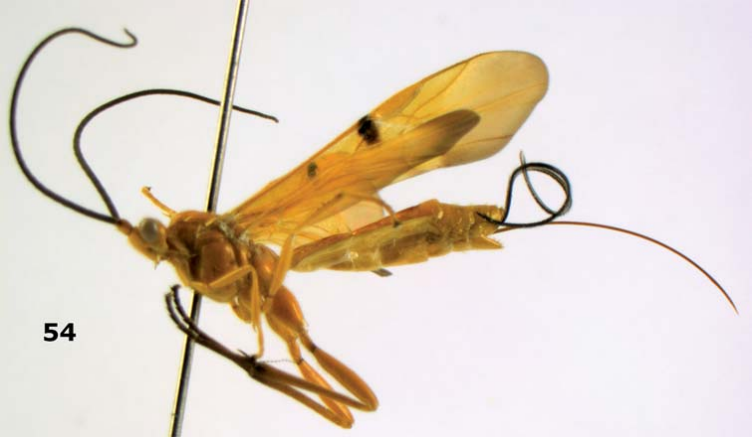
Braunsia devriesi sp. n., female, holotype. Habitus lateral.

First tergite rather long, widened apically, 2.3 times as long as its apical width (Fig. 57); first tergite longitudinally striate; dorsal carinae of first tergite strong, diverging apically and fused with striae apically (Fig. 57); second tergite as long as third tergite, longitudinally striate, with a deep striate transverse groove on apical third; third tergite with parallel striae on basal two thirds and with a wide striate transverse groove on apical third; apical third of third tergite and remainder of metasoma smooth, with sparse setae apically; ovipositor sheath long, rather narrow (Fig. 54) and 0.8 times as long as fore wing.

###### Colour.

Bright brownish-yellow; malar space pale yellow; antenna (except yellow scapus) and hind tarsus dark brown; fore wing with dark brown stigmal spot (Fig. 58); wing membrane yellowish, apically faintly infuscate (Fig. 58).

##### Distribution.

NE Vietnam: Viet Tri.

**Figures 55–62.**
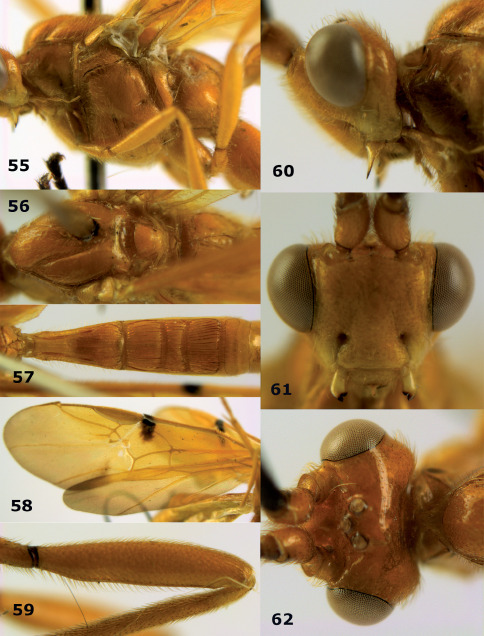
Braunsia devriesi sp. n., female, holotype. **55** mesosoma lateral **56** mesosoma dorsal **57** first-third metasomal tergites dorsal **58** wings **59** hind femur lateral **60** head lateral **61** head anterior **62** head dorsal.

##### Biology.

Unknown.

##### Etymology.

It is a pleasure to name this species after Mr Rob de Vries, who participated in all Vietnam expeditions and prepared the specimens. He plays an important role in the success of the expeditions.

#### Braunsia maculifera sp. n.

urn:lsid:zoobank.org:act:8F7EA4FE-E092-4AC3-973B-64110615F11A

Figs 63 –71 

##### Type material.

Holotype, ♀ (RMNH), Aga. 162, “NE Vietnam: Phu Tho, Xuan Son N.P., 6.vii.2003, Tr.X. Lam”.

##### Diagnosis.

The new species is morphologically similar to Braunsia margaroniae Nixon, 1950, from India, but differs by having the sides of the propodeal areola slightly curved (distinctly curved in Braunsia margaroniae), the pterostigma completely yellow (apical two thirds brown), the hind tibia yellowish apically (brown) and the hind tarsus infuscate (brown), frons shallowly depressed near antennal sockets (distinctly concave), the antenna (except scapus and pedicellus) dark brown (brown), the first tergite 1.4 times (1.6 times) as long as its apical width, the fore wing without an isolated dark brown stigmal spot (present) and the ovipositor sheath about 0.6 times (1.0–1.1 times) as long as body.

##### Description.

Holotype, ♀, length of body 8.7 mm, of fore wing 7.8 mm, ovipositor 4.8 mm.

###### Head.

Antennal segments 42; length of third segment 1.1 times fourth segment, length of third, fourth and penultimate segments 2.9, 2.6 and 1.4 times their width, respectively; apical antennal segment 1.7 times as long as penultimate segment; length of maxillary palp 0.7 times height of head; in dorsal view length of eye 2.2 times temple (Fig. 71); POL:OD:OOL = 8:5:14; antennal sockets distinctly protruding (Fig. 71); occipital flange large, its ventral margin convex (Fig. 70); length of malar space 1.9 times basal width of mandible; face setose and punctulate; frons smooth, moderately concave near antennal sockets; vertex slightly punctulate and sparsely setose.

###### Mesosoma.

Length of mesosoma 1.4 times its height; subpronope large and deep; side of pronotum smooth; area near lateral carina of mesoscutum smooth; side of mesoscutum largely smooth, moderately setose and punctulate; notauli deep and nearly completely smooth (Fig. 65); scutellar sulcus deep, 0.4 times as long as dorsal face of scutellum and with two short crenulae; scutellum smooth, distinctly convex and rather steep posteriorly; mesopleuron shiny and smooth; precoxal sulcus narrow, deep (but absent anteriorly) and with few short crenulae (Fig. 64); metapleuron largely smooth, punctulate and with some rugulae ventrally; propodeum with a rather weak complete transverse carina and a subparallel-sided areola (Fig. 65); spiracle rather large, elliptical, close to lateral carina and twice as long as wide; lateral carina of propodeum comparatively weak medially.

###### Wings.

Fore wing: second submarginal cell pentagonal, narrow anteriorly, with medium-sized ramellus slightly shorter than vein 2-SR (Fig. 67); r:3-SR:SR1 = 6:2:54; SR1 distinctly sinuate; 2-SR:3-SR:r-m = 5:1:5; vein cu-a interstitial. Hind wing: vein 2-SR+M longitudinal; vein M+CU 0.9 times as long as vein 1-M; surroundings of vein cu-a moderately setose.

###### Legs.

Hind coxa largely smooth, punctulate; length of hind femur, tibia and basitarsus 4.2, 8.2 and 8.2 times their width, respectively; hind femur (as remainder of legs) with medium-sized dense setosity; fore tarsal segments rather short, fourth segment about as long as wide; outer side of apical two thirds of middle tibia with a row of 3 pegs and a cluster of 2 pegs at apex; outer side of apex of hind tibia with a cluster of 7 pegs; length of outer and inner spurs of middle tibia 0.4 and 0.5 times middle basitarsus, respectively; length of outer and inner spurs of hind tibia 0.3 and 0.5 times hind basitarsus, respectively.

###### Metasoma.

**Figure 63.**
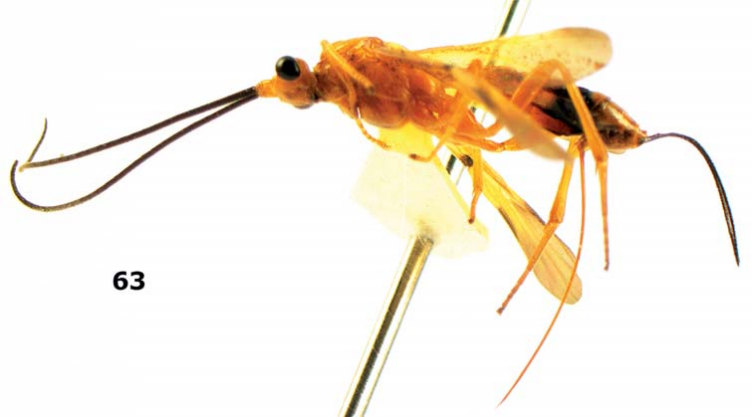
Braunsia maculifera sp. n., female, holotype. Habitus lateral.

First tergite distinctly widened apically, 1.4 times as long as its apical width (Fig. 66); basal third of first tergite smooth, remainder longitudinally costate as second tergite; dorsal carinae of first tergite strong lamelliform, slightly diverging posteriorly and nearly complete (Fig. 66); second tergite 0.8 times as long as wide apically and about as long as third tergite, longitudinally costate, with transverse groove submedially; third tergite with parallel costae and after striate transverse groove smooth; remainder of metasoma smooth and with sparse setae apically; ovipositor sheath narrow (Fig. 63) and 0.6 times as long as body or fore wing.

###### Colour.

Yellowish-brown; antenna dark brown, but scapus and pedicellus yellowish-brown; malar space slightly paler yellow than surroundings; fore wing with dark brown stigmal spot (Fig. 67); apical third of wings brownish, basal two thirds and veins yellow (but R1 dark brown); pterostigma entirely yellow; parastigma dark brown; legs brownish-yellow; hind tarsus slightly more brownish; first tergite yellow basally; ovipositor sheath black.

##### Distribution.

NE Vietnam: Phu Tho.

**Figures 64–71.**
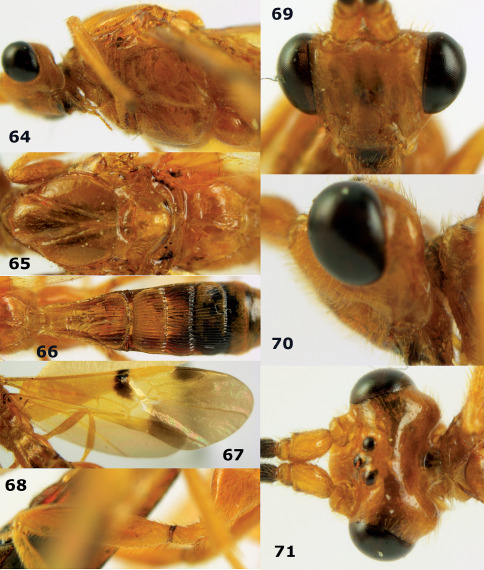
Braunsia maculifera sp. n., female, holotype. **64** mesosoma lateral **65** mesosoma dorsal **66** first-third metasomal tergites dorsal **67** wings **68** hind femur lateral **69** head anterior **70** head lateral **71** head dorsal.

##### Biology.

Unknown.

##### Etymology.

From “macula” (Latin for “spot”), and “fero” (Latin for “carry”), because of the dark brown stigmal spot.

#### Braunsia nigrapiculata sp. n.

urn:lsid:zoobank.org:act:3D8DA4B9-2EC1-4181-B2E1-4F874B2E9025

Figs 72 –80 

##### Type material.

Holotype, ♀ (RMNH), Aga. 265, “C. Vietnam: Thua Thien-Hue, Nam Dong, MT, 2–6.v.2005, N.Q. Truong”. Paratypes: 1 ♀ (IEBR), Aga. 190, “C. Vietnam: Quang Nam, Phuoc Son, forest, 28.vii.2004, N.Th. Huong”.

##### Diagnosis.

The new species similar to Braunsia bipunctata Enderlein, 1906, from Indonesia, but differs by having the transverse subbasal carina of the propodeum complete and moderately developed (Braunsia bipunctata has transverse carina partly weakly developed and irregular); the vein 1-R1 of the fore wing dark brown (bipunctata: yellow), the apical half of the third tergite longitudinally striate, with only apical fourth smooth (bipunctata: completely smooth or nearly so) and the metasoma dorsally (except basally), the inner side of the hind femur and the hind tibia largely dark brown (bipunctata: yellowish-brown).

##### Description.

Holotype, ♀, length of body 11.5 mm, of fore wing 10.3 mm, ovipositor 10.4 mm.

###### Head.

Antennal segments 50; length of third segment 1.3 times fourth segment, length of third, fourth and penultimate segments 2.5, 2.0 and 1.7 times their width, respectively; apical antennal segment 1.8 times as long as penultimate segment; length of maxillary palp 0.9 times height of head; in dorsal view length of eye twice temple (Fig. 80); POL:OD:OOL = 7:5:14; antennal sockets slightly tubular; occipital flange large, its ventral margin round (Fig. 79); face setose and with fine punctures; frons smooth; vertex smooth, sparsely setose.

###### Mesosoma.

Length of mesosoma 1.5 times its height; subpronope large and deep; side of pronotum smooth; area near lateral carina of mesoscutum smooth; side of mesoscutum largely smooth with sparse setae and fine punctures; notauli deep and smooth (Fig. 74); scutellar sulcus short, 0.4 times as long as dorsal face of scutellum and without carina; scutellum smooth, distinctly convex anteriorly and sloping posteriorly; mesopleuron above precoxal sulcus shiny and smooth, below precoxal sulcus shiny with minute punctures; precoxal sulcus narrow, similar to a smooth groove (Fig. 73); metapleuron smooth; propodeum with a subbasal transverse carina, without areola, spiracle large, elliptical, close to lateral carina and 2.8 times as long as wide; lateral carina of propodeum interrupted medially.

###### Wings.

Fore wing: second submarginal cell pentagonal, narrow anteriorly, with rather long ramellus, distinctly angled at ramellus and 1.2 times as long as vein 2-SR (24:20) (Fig. 75); r:3-SR:SR1 = 10:5:88; 2-SR:3-SR:r-m = 20:5:20; vein cu-a postfurcal. Hind wing: vein 2-SR+M vertical; vein M+CU 0.9 times as long as vein 1-M (24:27); surroundings of cu-a sparsely setose.

###### Legs.

Length of hind femur, tibia and basitarsus 5.0, 8.7 and 11.0 times their width, respectively; hind femur (as remainder of legs) with short and dense setosity (Fig. 77); outer side of apical third of middle tibia with a row of 5 pegs and a cluster of 3 pegs at apex; outer side of apex of hind tibia with a cluster of 7 pegs; length of outer and inner spurs of middle tibia 0.3 and 0.4 times middle basitarsus, respectively; length of outer and inner spurs of hind tibia 0.4 and 0.5 times hind basitarsus, respectively.

###### Metasoma.

First tergite moderately long, widened apically, 1.9 times as long as its apical width (Fig. 76); basal half of first tergite smooth, apical half longitudinally striate as second tergite; dorsal carinae of first tergite strong, diverging apically (Fig. 76); second tergite as long as third tergite, longitudinally striate, deep striate transverse groove on apical third; third tergite with parallel striae but smooth on extreme apex; striate transverse groove on apical third wide; remainder of metasoma smooth with sparse setae apically; ovipositor sheath broken; ovipositor about as long as fore wing.

###### Colour.

Brownish-yellow; antenna brown; malar space pale yellow; fore wing with a vague brown stigmal spot (Fig. 75); apical third of wings dark brown, basal two thirds yellow; parastigma dark brown; hind tarsus yellowish-brown; hind tibia and tarsus dark brown; metasoma black, but basal two thirds of first tergite yellow; first-third metasomal segments ivory ventrally.

##### Distribution.

C Vietnam: Thua Thien-Hue, Quang Nam.

**Figure 72.**
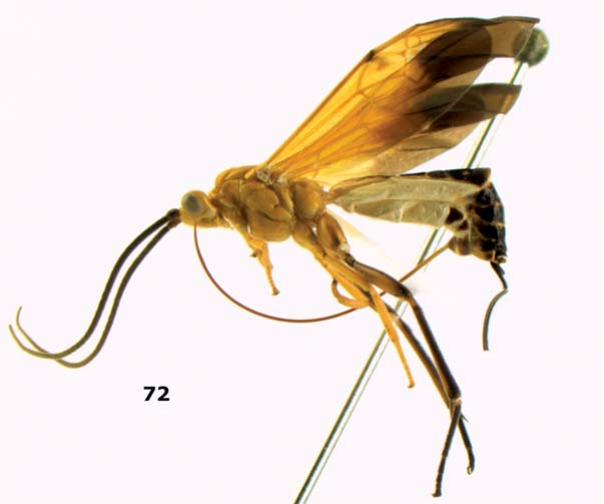
Braunsia nigrapiculata sp. n., female, holotype. Habitus lateral.

**Figures 73–80.**
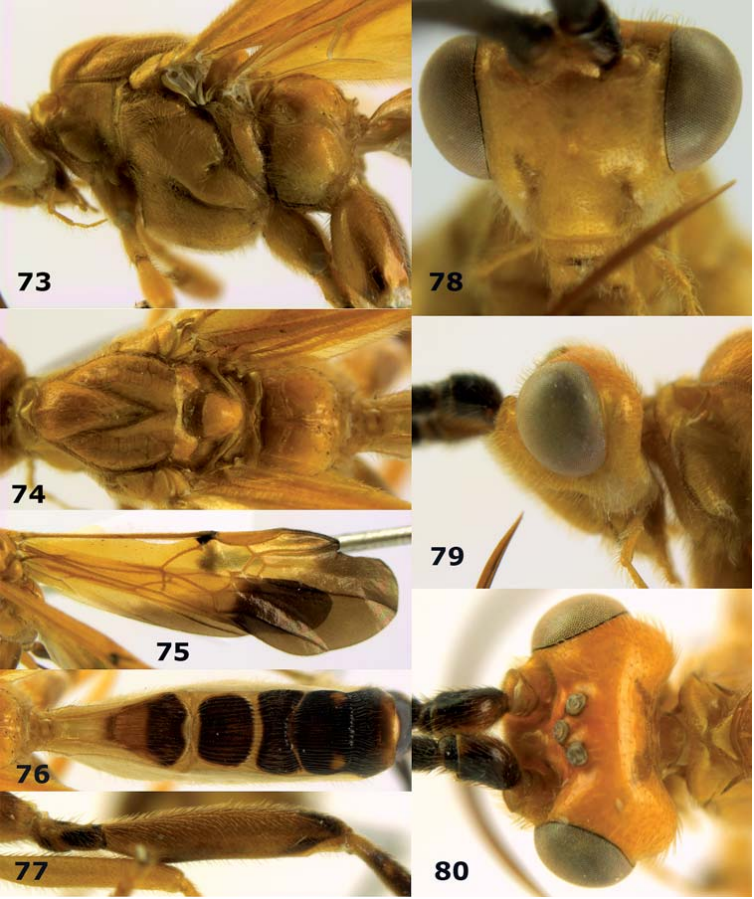
Braunsia nigrapiculata sp. n., female, holotype. **73** mesosoma lateral **74** mesosoma dorsal **75** wings **76** first-third metasomal tergites dorsal **77** hind femur lateral **78** head anterior **79** head lateral **80** head dorsal.

##### Biology.

Unknown.

##### Etymology.

From “nigra” (Latin for “black”), and “apiculatus” (Latin for “small-pointed”), because of the black apical part of the body.

#### Braunsia pumatica sp. n.

urn:lsid:zoobank.org:act:006E3326-5CFA-40D4-BA55-88C89DE65171

Figs 81 –90 

##### Type material.

Holotype, ♀ (RMNH), “S. Vietnnam: Dak Lak, Chu Yang Sin N.P. Krong K’Mar Mal. traps 740–900 m, 2–10.vii.2007, C. v. Achterberg & R. de Vries, RMNH’07”. Paratypes (7 ♀): 1 ♀ (IEBR), Aga. 179, “NE Vietnam: Phu Tho, Xuan Son N.P., 6–9.xii.2003, Tr.X. Lam”; 1 ♀ (IEBR), Aga. 196, ”Central North Vietnam: Nghe An, Pu Mat N.P., 6–9.viii.2005, N.Th. Huong”; 2 ♀ (IEBR), Aga. 295 and Aga. 180, id., but 13.iv.2006, P.Th. Nhi and 22–25.vii.2004, Tr.X. Lam; 1 ♀ (RMNH), Aga. 243, “Central North Vietnam: Nghe An, Pu Mat N.P., 12.ix.2005, P.Th. Nhi”; 2 ♀ (RMNH), Aga. 301 and Aga. 302, id., but 17.iv.2006.

##### Diagnosis.

The new species is close to Braunsia sumatrana Enderlein, 1906, from Indonesia and East Malaysia, but differs by having the first tergite 3.3 times (twice in Braunsia sumatrana) as long as its apical width, basal two thirds of the third tergite longitudinally striate (only basal half) and the apical third of the third tergite micro-sculptured (smooth) and the hind coxa smooth (sparsely finely punctate).

##### Description.

Holotype, ♀, length of body 9.2 mm, of fore wing 7.5 mm, ovipositor 8.0 mm.

###### Head.

Antennal segments 47; length of third segment 1.3 times fourth segment, length of third, fourth and penultimate segments 2.5, 2.2 and 1.3 times their width, respectively; length of apical antennal segment 1.6 times as long as penultimate segment; maxillary palp 0.8 times height of head; in dorsal view length of eye 2.9 times temple (Fig. 88); POL:OD:OOL = 7:5:13; antennal sockets weakly tubular (Fig. 88); face shiny, with sparse punctures; frons and vertex shiny and smooth; occipital flange rounded, area hardly protruding (Fig. 89).

###### Mesosoma.

Length of mesosoma 1.4 times its height; subpronope large and deep; side of pronotum smooth; area near lateral carina of mesoscutum with crenulae; side of mesoscutum nearly smooth, with sparse fine punctures and setae; notauli wide and crenulate anteriorly, narrower and smooth posteriorly (Fig. 83); scutellar sulcus deep and with 3 carinae; scutellum slightly convex with sparse fine punctures; mesopleuron above precoxal sulcus largely smooth; mesopleuron below precoxal sulcus with sparse fine punctures; precoxal sulcus wide and shallow, slightly crenulate (Fig. 80); metapleuron with sparse fine punctures; propodeum with a strong subbasal carina (Fig. 83), without areola, largely rugose posteriorly; spiracle medium-sized, round and 1.7 times as long as wide.

###### Wings.

Fore wing: second submarginal cell triangular, with rather long ramellus, 0.7 times as long as vein r (11:15) (Fig. 84); r:3-SR:SR1 = 5:1:61; 2-SR:3-SR:r-m = 15:1:15; vein cu-a of fore wing distinctly antefurcal. Hind wing: vein M+CU 0.4 times as long as 1-M (24:57); surroundings of cu-a glabrous.

###### Legs.

Length of hind femur, tibia and basitarsus 5.4, 7.6 and 9.0 times their width, respectively; hind coxa shiny smooth; hind femur with sparse fine punctures and medium-sized setosity (Fig. 86); outer side of apical third of middle tibia with a row of 4 pegs; length of outer and inner spurs of middle tibia 0.5, and 0.6 times their basitarsus, respectively; length of outer and inner spurs of hind tibia 0.4, and 0.5 times their basitarsus.

###### Metasoma.

First tergite long and slightly widened apically (Fig. 85), shiny and largely smooth; length of first tergite 3.3 times its apical width; dorsal carinae of first tergite diverging and fused apically; second tergite long and nearly parallel-sided; second tergite 2.6 times longer than wide apically; second tergite and base of third tergite largely striate; third tergite micro-sculptured apically; remainder of metasoma smooth; ovipositor sheath wide, ribbon-shaped (Fig. 90), 1.3 times as long as fore wing.

###### Colour.

Black; malar space ivory; clypeus, palpi, mandible, fore and middle legs yellow; mesosoma reddish-yellow (but below precoxal sulcus black); first tergite basally, first and second tergite ventrally white; wing membrane somewhat infuscate (Fig. 84).

###### Variation.

Male with 44–45 antennal segments; length of apical antennal segment 1.4 times as long as penultimate segment; vein cu-a of fore wing slightly antefurcal; vein 3-SR vertical; vein M+CU of hind wing 0.6 times as long as 1-M; outer side of middle tibia with row of 3 or 6 pegs and cluster of 3 and 5 pegs at apex; first and second metasomal segments whitish-yellow basally.

##### Distribution.

NE Vietnam: Phu Tho, CN Vietnam: Nghe An and S Vietnam: Dak Lak.

**Figure 81.**
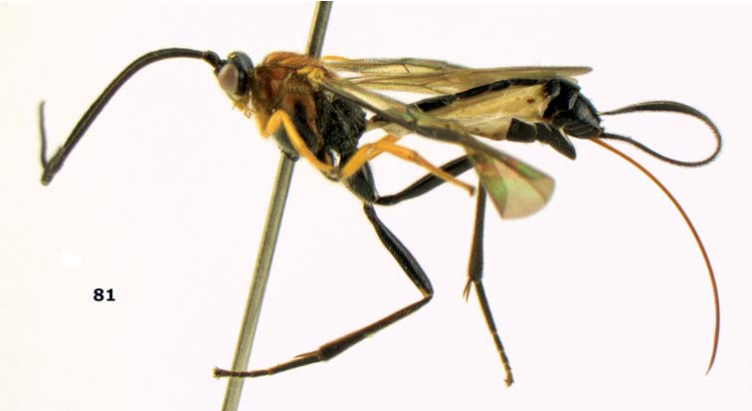
Braunsia pumatica sp. n., female, holotype. Habitus lateral.

**Figures 82–90.**
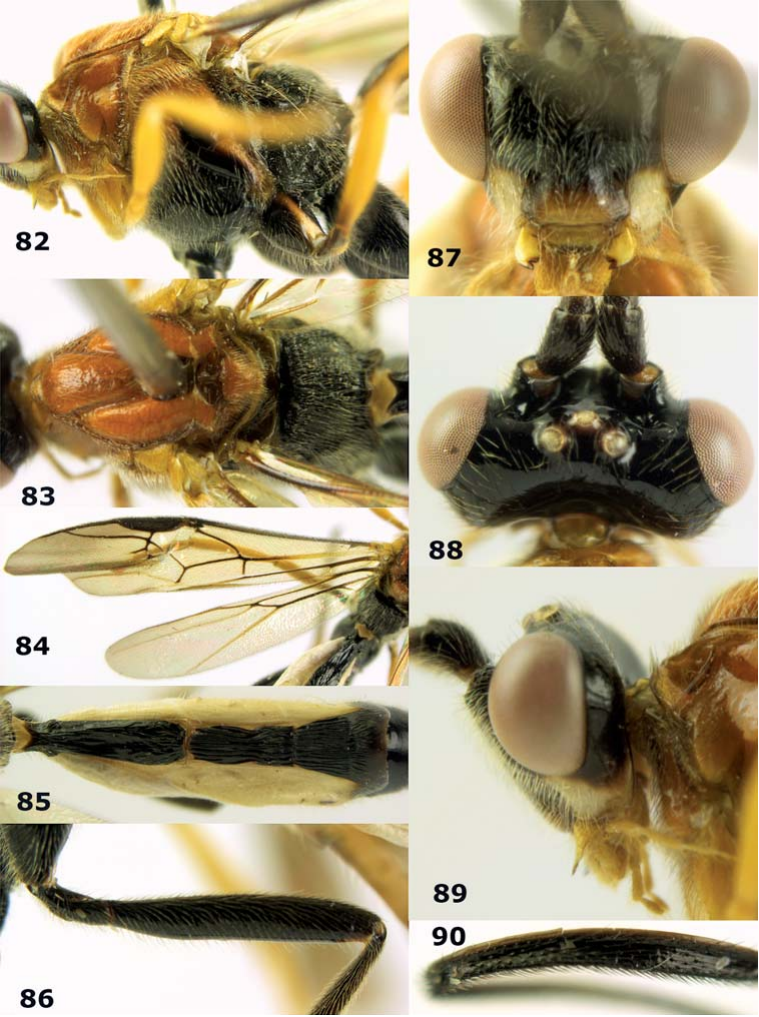
Braunsia pumatica sp. n., female, holotype. **82** mesosoma lateral **83** mesosoma dorsal **84** wings **85** first-third metasomal tergites dorsal **86** hind femur lateral **87** head anterior **88** head dorsal **89** head lateral **90** ovipositor sheath.

##### Biology.

Unknown.

##### Etymology.

Phantasy name.

### Genus Camptothlipsis

Enderlein, 1920

#### Notes.

In Vietnam only one species was found. Camptothlipsis hanoiensis sp. n. from North Vietnam. Therophilus tonghuaensis (Chen & Yang, 2006) comb. n. from Jilin (China) was originally assigned to the genus Camptothlipsis, but does not belong there because it has a shortvein r-m of the fore wing, resulting in a minute second submarginal cell and the basal metasomal tergites are coarsely sculptured (not finely granulate). Recently, Stevens et al. (2010) synonymised the senior genus Baeognatha Kokujev, 1903 with Camptothlipsis, resulting in a change of name of the genus. The synonymy is not accepted because the type species (Baeognatha turanica Kokujev, 1903) does not run in the key by Stevens et al. to Camptothlipsis (renamed in the paper as Baeognatha) because the vein r-m of the fore wing (1-RS in Stevens et al.) is present (Figs 131–133 in Simbolotti & van Achterberg, 1992). Baeognatha turanica is close to the genus Agathis Latreille considering the shape of the head, but differs by the elongate and nearly straight tarsal claws (Figs 138, 140, 141, l.c.) and, therefore, is retained as a separate genus.

#### Camptothlipsis hanoiensis sp. n.

urn:lsid:zoobank.org:act:79DB7F73-768E-494E-8D83-60230DCF2E2D

Figs 91 –99 

##### Type material.

Holotype, ♀ (RMNH), Aga. 040, “N. Vietnam: Ha Noi, Tu Liem, rice field, 13.vii.1996, K.D. Long”. Paratypes (1 ♂ + 1 ♀): 1 ♀ (IEBR), Aga. 348, “N. Vietnam: Ha Noi, Gia Lam, orchard, MT, 25.x-5.xi.2001, K.D. Long”. 1 ♂ (RMNH), Aga. 246, same data as holotype, but 18.x.2003.

##### Diagnosis.

Differs from Camptothlipsis taichungensis Chou & Sharkey, 1989, from Taiwan (China) by having the ovipositor sheath 0.9 times as long as fore wing (1.0–1.1 times in Camptothlipsis taichungensis), propodeum without rugosity (coarsely reticulate-rugose medially), pterostigma with a small pale basal area (absent), length of the malar space 3.3 times basal width of the mandible (2.1–2.4 times), posterior groove of pronotal side largely without crenulae (completely crenulate) and medio-posterior groove of mesoscutum shorter (longer, Fig. 163 in Chou & Sharkey, 1989). Camptothlipsis flavidus Gupta & Bhat, 1974, from India has the first tergite about as long as wide apically and the precoxal sulcus absent anteriorly. Camptothlipsis gossypiella Gupta & Bhat, 1974, from India is similar but has the propodeum finely rugose basally and the second tergite with a distinctly impressed transverse groove. Camptothlipsis dravida Gupta & Bhat, 1974, from India is very similar because it has the propodeum only granulate and first tergite 1.3–1.4 times as long as wide apically, but the length of the malar space is 2.5 times basal width of mandible, the transverse groove of the second tergite is distinctly developed, the frons very finely punctate and the metapleuron weakly granulate.

##### Description.

Holotype, ♀, length of body 3.4 mm, of fore wing 2.9 mm.

###### Head.

Antennal segments 29, length of third segment 1.1 times fourth segment, length of third, fourth and penultimate segments 3.4, 3.0 and 1.7 times their width, respectively; length of apical antennal segment 1.2 times as long as penultimate segment; maxillary palp 0.6 times height of head; malar space 3.3 times as long as basal width of mandible; in dorsal view length of eye 4.0 times temple; temple roundly narrowed posteriorly; ocelli in rather high triangle, POL:OD:OOL = 6:4:8 (Fig. 99); face sparsely finely punctate and its width 1.3 times height of face and clypeus combined; frons with rather dense distinct punctation laterally; vertex and temple near smooth.

###### Mesosoma.

Length of mesosoma 1.5 times its height; pronotal trough largely granulate, rugose anteriorly, posterior groove only ventrally crenulate; area near lateral carina of mesoscutum weakly crenulate; lateral lobes of mesoscutum flat posteriorly, sparsely finely punctate, middle lobe of mesoscutum with dense punctures anteriorly and sparsely punctate posteriorly; notauli complete and finely crenulate, united far in front of scutellar sulcus and medio-posterior groove medium-sized (Fig. 93); scutellar sulcus 0.5 times as long as scutellum with 5 carinae; scutellum sparsely finely punctate; precoxal sulcus absent anteriorly and remainder finely crenulate; mesopleuron below precoxal sulcus with sparse fine punctures; mesopleuron above precoxal sulcus shiny, granulate; metapleuron and propodeum distinctly granulate, without distinct rugosity medially.

###### Wings.

Fore wing: marginal cell narrow (Fig. 96); vein SR1 straight; r:3-SR+SR1= 3:48; subbasal cell evenly setose. Hind wing: vein M+CU 0.8 times as long as vein 1-M.

###### Legs.

Length of hind femur, tibia and basitarsus 3.3, 6.4 and 9.7 times their width, respectively; hind femur (as remainder of legs) with short setae (Fig. 95); length of outer and inner spur of middle tibia 0.5 and 0.7 times middle basitarsus, respectively; outer side of middle tibia with a cluster of 9 pegs of which 2 at apex; length of outer and inner spur of hind tibia 0.3 and 0.5 times hind basitarsus, respectively; tarsal claw with lobe.

###### Metasoma.

Length of first tergite 1.3 times its apical width (Fig. 94); first-second tergites granulate; second tergite with basal area bordered by a superficial transverse groove (Fig. 94); remainder of metasoma smooth; ovipositor sheath 0.9 times as long as fore wing.

###### Colour.

Yellowish-brown; antenna, apex of hind tibia, hind tarsus, metapleuron ventrally, propodeum, first tergite and ovipositor sheath dark brown; face, mouthparts, second tergite and remainder of legs brownish-yellow; wing venation pale brown, but pterostigma brown (except small pale brown patch basally); wing membrane subhyaline.

###### Variation.

Length of body 2.9–3.4 mm, and of fore wing 2.4–3.2 mm; antenna of both sexes 28–29; POL:OD:OOL = 5–6:4:8–10 (female); venation (including pterostigma) pale to dark brown; first tergite yellowish-brown to largely dark brown.

##### Distribution.

N Vietnam: Ha Noi.

**Figure 91.**
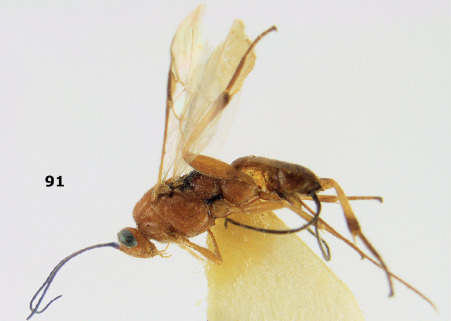
Camptothlipsis hanoiensis sp. n., female, holotype. Habitus lateral.

**Figures 92–99.**
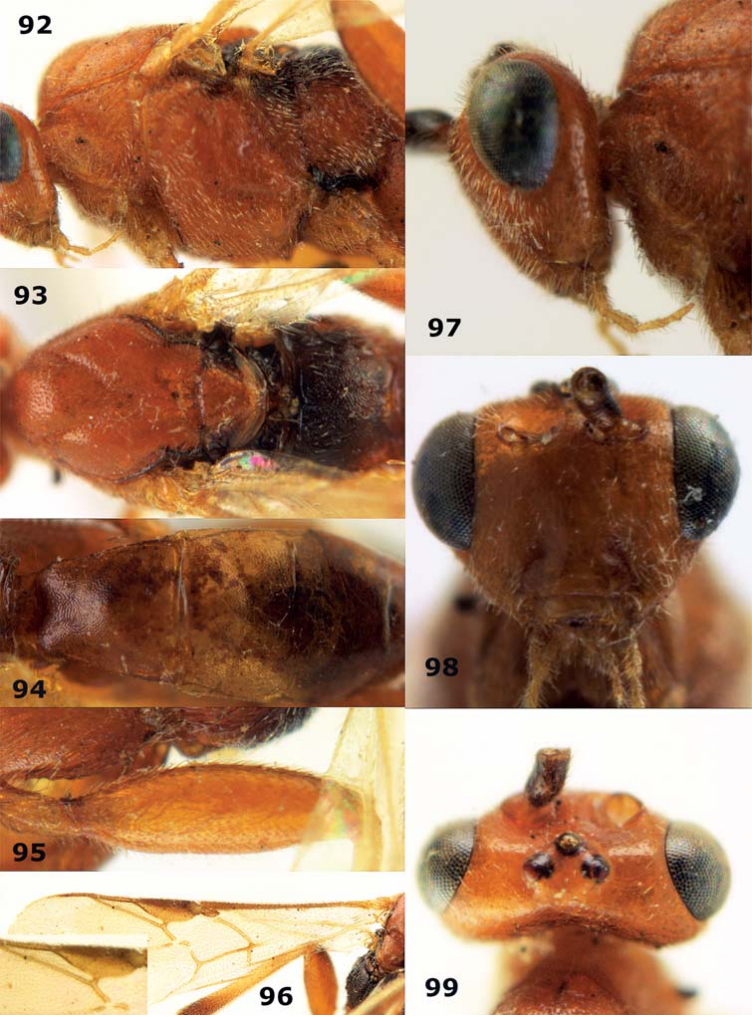
Camptothlipsis hanoiensis sp. n., female, holotype. **92** mesosoma lateral **93** mesosoma dorsal **94** first-third metasomal tergites dorsal **95** hind femur lateral **96** wings **97** head lateral **98** head anterior **99** head dorsal.

##### Biology.

Unknown.

##### Etymology.

Named after the type locality.

### Genus Coccygidium

de Saussure, 1892

#### Notes.

Prior to Sharkey et al (2009) the Old World species were included in the New World genus Zelomorpha Ashmead, 1900 or the Old World genus Coccygidium de Saussure, 1892. Most Oriental species belong to the Zelodia **gen. n.**, but some Oriental species and most species in the Afrotropical and Australian regions belong here. Including Disophrys concolor Szépligeti, 1908 from Sundaland and possibly Sri Lanka; the type species of the genus Amputostypos Sharkey, 2009. The lectotype of Disophrys concolor (designated by van Achterberg 1974) has been examined and it has the fore spur with a long curved and glabrous apical spine as figured in the key (a) for Coccygidium. Therefore, Amputostypos Sharkey is synonymised with Coccygidium de Saussure (**syn. n.**). From the north-eastern Oriental region only two species of Coccygidium de Saussure are known: Coccygidium sissoo (Wilkinson, 1929) comb. n. [a senior synonym of Zelomorpha amplarga Gupta & Bhat, 1972, synonymy according to Bhat (1978)] and Coccygidium angostura (Bhat & Gupta, 1977). In Vietnam only the last species was found.

#### Coccygidium angostura

(Bhat & Gupta, 1977)

Figs 100–102 

##### Distribution.

NE Vietnam: Hoa Binh. New to Vietnam. New record. Outside Vietnam known from China (Anhui; Fujian; Guangdong; Hainan Island; Henan; Hubei; Jiangxi; Sichuan; Yunnan; Zhejiang).

**Figures 100–102.**
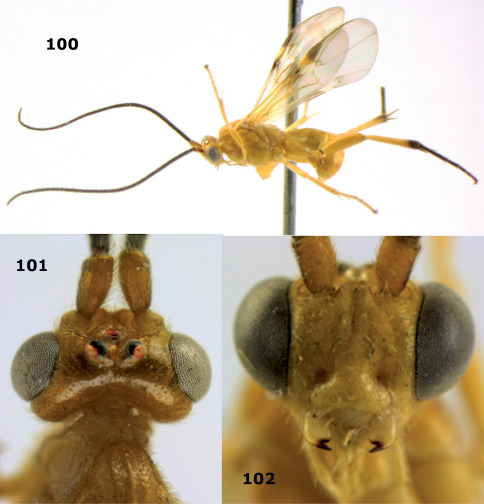
Coccygidium angostura (Bhat & Gupta), male, Yen Thuy. **100** habitus lateral **101** head dorsal **102** head anterior.

##### Note.

Length of inner spur of middle tibia varies from 0.8–1.1 times length of middle basitarsus and vein cu-a of fore wing is distinctly antefurcal or subinterstitial.

#### Genus Coronagathis gen. n.

urn:lsid:zoobank.org:act:F1039ED0-91FA-48AA-8C20-5A2309C20D6B

##### Type species.

Coronagathis cornifera sp. n.

##### Etymology.

Combination of “corona” (Latin for “crown”) and the generic name Agathis Latreille because of the protuberances of the scutellum and the axillae. Gender: feminine.

##### Diagnosis.

Apex of antenna without spine; malar space somewhat protruding posteriorly (Fig. 109); area between antennal sockets with a pair of lamelliform crests (Figs 104, 105); frons with lateral carinae running to posterior ocelli; area behind antennal sockets rather deep and outer rim of antennal socket protruding; outer aspect of scapus sparsely punctulate and convex; malar suture absent; notauli complete and crenulate (Fig. 110); scutellum with pair of large horns and axillae protruding, wing-like (Figs 106, 110); second submarginal cell of fore wing rectangular and with an obsolete ramellus (Fig. 109); vein M+CU of hind wing about half as long as vein 1-M; hind wing with 2 + 4 hamuli; subbasal cell of hind wing narrower than plical lobe (Fig. 109); fore tarsal claws bifurcate, the inner tooth nearly as large as the outer tooth; outer face of middle tibia with one lateral and one apical peg; inner spur of middle tibia about 0.6 times as long as middle basitarsus; ventral carina of hind trochantellus weakly developed; inner and outer hind claw bifurcate, with a medium-sized subapical inner tooth; hind coxa enlarged and coarsely sculptured; first metasomal tergite smooth and depressed behind spiracles (Fig. 107); length of ovipositor sheath unknown, but probably about as long as apical height of metasoma or less.

##### Phylogenetic position.

Putative synapomorphous character states of the new genus Coronagathis are the horns of the scutellum, the wing-like axillae and the coarsely sculptured and elongate hind coxa, surpassing the apex of the first tergite. Its position is uncertain, but seems to be betweenthe genus Disophrys Foerster and the Coccygidium complex. Similar to Disophrys because of the carinae of the frons (present and running to the lateral ocelli) and the elongate hind leg. However, the derived character state of the short vein M+CU of the hind wing indicates that it belongs to the more derived group consisting of the Coccygidium complex and the genus Euagathis Szépligeti. The new genus lacks the synapomorphies of the Coccygidium complex as the apical antennal spine and the long inner spur of the middle tibia. It cannot be included in Euagathis, because Euagathis is characterised by the derived character state of the absent lateral carinae of the frons. The genus Hypsostypos Baltazar from Sulawesi and Sundaland is similar, but Hypsostypos has several autapomorphies. For example, the long impressed malar suture, the reduced notauli and the enlarged lamelliform antennal sockets.

##### Distribution.

Vietnam.

##### Biology.

Unknown.

#### Coronagathis cornifera sp. n.

urn:lsid:zoobank.org:act:D600224D-4DD1-4C8F-860A-6F4FD57F02BB

Figs 103 –110 

##### Type material.

Holotype, ♂ (RMNH), “N.W. Vietnam: Tonkin, Hoang Lien N.P., 10 km, SW Sa Pa, c. 1550 m, 22–29.x.1999, Malaise traps, C. van Achterberg & R. de Vries, RMNH’99”.

##### Diagnosis.

Except for the aberrant scutellum and axillae it may be confused with aberrant species of the genus Disophrys Foerster because of the carinae of the frons (present and running to the lateral ocelli), the more or less angulate hind trochantellus and the elongate hind leg. However, it is separated from Disophrys by the size of the subbasal cell of the hind wing (narrower than plical lobe (Fig. 109; about as wide as plical lobe in Disophrys) and the short vein M+CU of the hind wing (distinctly shorter than vein 1-M versus at least subequal in Disophrys). Superficially similar to the genus Euagathis Szépligeti, but the scutellum bears a pair of large horns (Fig. 110; absent in Euagathis), the frons has lateral carinae (Fig. 105; absent in Euagathis), the hind trochantellus is more or less angulate ventrally (rounded ventrally in Euagathis) and the subbasal cell of the hind wing is narrower than the plical lobe (Fig. 109; subequal in Euagathis).

##### Description.

Holotype, ♂, length of body 6.3 mm, of fore wing 6.2 mm.

###### Head.

Antennal segments 47, length of third segment 1.5 times fourth segment, length of third, fourth and penultimate segments 3.2, 2.2 and 1.4 times their width, respectively; length of maxillary palp 0.7 times height of head; head in dorsal view 2.9 times as wide as its median length; in dorsal view length of eye 1.5 times temple; temple nearly straight behind eyes (Fig. 105) and spaced finely punctate; POL:OD:OOL = 10:5:8; clypeus, face and vertex coarsely areolate-punctate; face with long setae; clypeus strongly convex; frons largely smooth, rather deep near antennal sockets, lateral carina running from near antennal socket to posterior ocellus (Fig. 105); pair of crests between antennal sockets lamelliform, strongly protruding, parallel-sided, slightly higher than protruding outer rim of antennal sockets.

###### Mesosoma.

Length of mesosoma 1.5 times its height without spine; subpronope large and deep, epomial carina strong; side of pronotum shiny smooth anteriorly, upper side largely punctate, crenulate posteriorly; area near lateral carina of mesoscutum wide, strongly crenulate; side of mesoscutum long rugose-punctate; medio-posteriorly impressed, largely crenulate, its middle lobe long, almost parallel-sided laterally, without a pair of shallow grooves or a median carina anteriorly; notauli wide and strongly crenulate (Fig. 110); scutellar sulcus wide and deep with one carina; scutellum areolate-rugose with long setae; subposterior carina evenly curved, wide and raised, forming a pair of strong lateral long horns (Fig. 110); prepectal carina lamelliform; mesopleuron below sulcus largely densely coarsely punctate and above sulcus more spaced punctate; precoxal sulcus wide, rather shallow and strongly crenulate; metapleuron largely coarsely vermiculate-rugose with dense long setae; mesosternal sulcus shallow and narrowly crenulate; propodeum shiny, largely smooth medially, with coarse carinae but without areola, costulae partly present; propodeum with large postero-dorsal tooth-like protuberance (Fig. 108); spiracle rather large, elliptical, close to latero-basal corner of propodeum and twice as long as wide.

###### Wings.

Fore wing: second submarginal cell quadrate, with an indistinct ramellus (Fig. 109); r:3-SR:SR1 = 4:7:80; 2-SR:3-SR:r-m = 13:7:11; apical half of subbasal cell sparsely setose. Hind wing: M+CU 0.6 times as long as 1-M; no 2-M; surroundings of cu-a glabrous.

###### Legs.

Hind femur 0.9 times as long as hind tibia; hind tibia distinctly compressed basally and widened apically; length of hind femur, tibia and basitarsus 5.0, 6.0 and 7.0 times their width, respectively; hind femur and tibia largely punctate, but partly pimply, with short setosity; middle tibia with two small pegs; length of outer and inner spurs of middle tibia 0.4 and 0,5 times middle basitarsus, respectively; length of outer and inner spurs of hind tibia 0.4 and 0.6 times hind basitarsus respectively; fore and middle tarsi slender.

###### Metasoma.

First tergite 2.2 times as long as its apical width, smooth and subparallel-sided apically (Fig. 107); second tergite without a transverse depression and second metasomal suture almost absent.

###### Colour.

**Figure 103.**
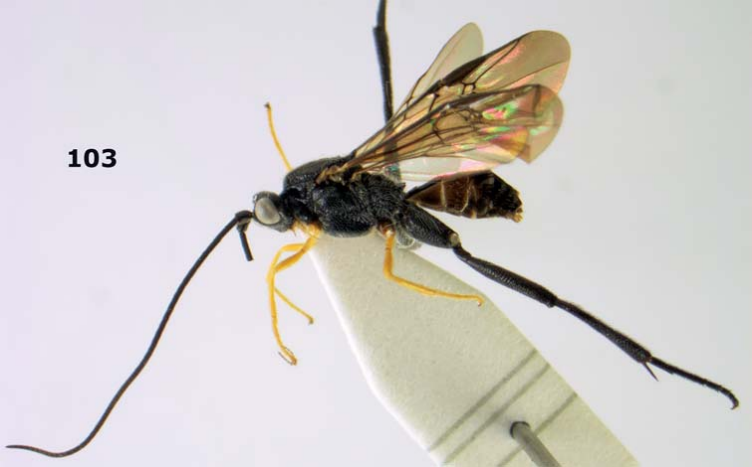
Coronagathis cornifera gen. n. sp. n., male, holotype. Habitus lateral.

Body black; wing membrane rather dark brown and without a stigmal spot (Fig. 103); mandible, palpi, fore and middle legs (but coxae rather brownish) pale yellow; pterostigma, veins and metasoma largely dark brown.

##### Distribution.

NW Vietnam: Lao Cai.

**Figures 104–110.**
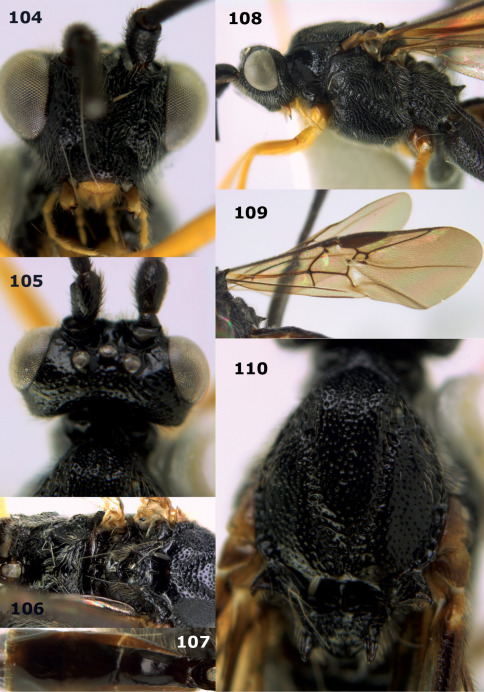
Coronagathis cornifera gen. n. sp. n., male, holotype. **104** head anterior **105** head dorsal **106** mesosoma dorsal **107** first-third metasomal tergites dorsal **108** mesosoma lateral **109** wings **110** mesoscutum and scutellum dorsal.

##### Etymology.

From “cornu” (Latin for “horn”), and “fero” (Latin for “carry”), because of the lateral horns of the scutellum.

### Genus Cremnops

Foerster, 1862

#### Cremnops atricornis

(Smith, 1874) stat. n.

Figs 111–113 

##### Distribution.

NE Vietnam: Phu Tho, Ha Giang, CN Vietnam: Nghe An and S Vietnam: Kien Giang, Ninh Thuan, Dong Nai. New record. Outside Vietnam known from China (Hunan; Liaoning; Shanxi; Taiwan), India, Indonesia (Java), Japan and Korea.

**Figures 111–113.**
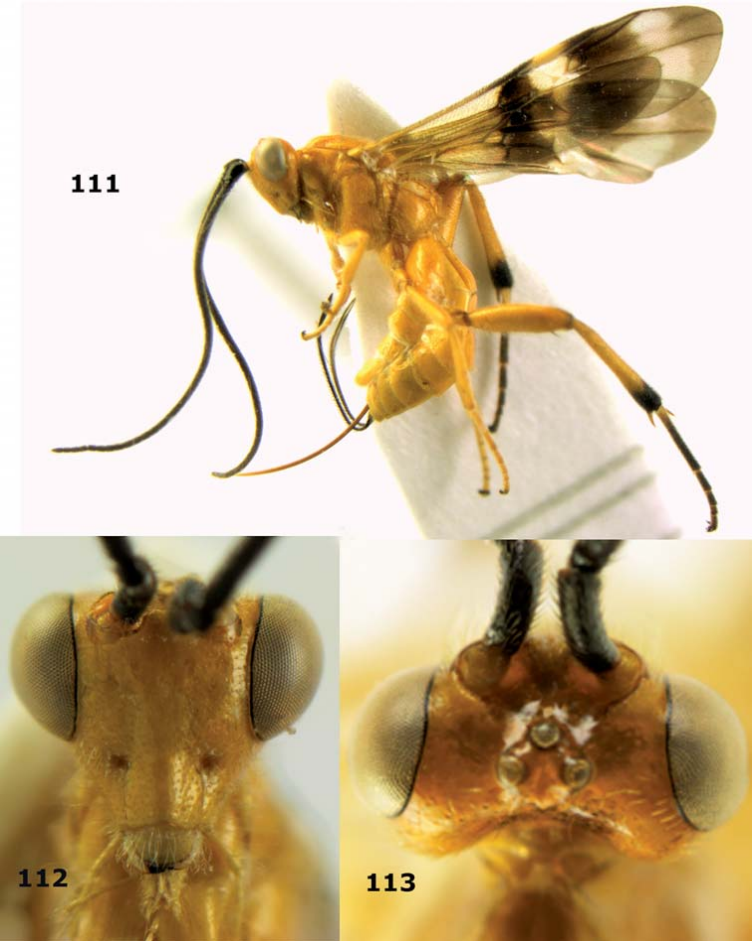
Cremnops atricornis (Smith), female, Cat Tien National Park. **111** habitus lateral **112** head anterior **113** head dorsal.

##### Diagnosis.

Very close to Cremnops desertor (Linnaeus, 1758). The traditionally used differences for separation are not sufficient and, consequently, it was synonymised by Sharkey (1996). Both species are very variable in colour, especially the extension of the dark parts of the wing membrane is variable and thus useless for diagnosis. Nevertheless, it is possible to separate the West Palaearctic typical specimens of Cremnops desertor from the East Palaearctic and Oriental specimens (including Cremnops atricornis) by the relative length of the fore tarsus of the females. In Cremnops desertor the fore tarsus is distinctly longer than the fore tibia and (including claws) about as long as the tibia in Cremnops atricornis. The Vietnamese specimens have the first tergite 1.5–1.8 times as long as its apical width (up to 2.2 times in East Palaearctic specimens according to Sharkey (1996)) and the vertex is yellowish-brown (Fig. 113).

### Genus Disophrys

Foerster, 1862

#### Note.

Pseudocremnops Szépligeti, 1902 (correctly synonymised with Disophrys by Sharkey et al., 2006) belongs here despite having more slender and completely dark brown wings (Fig. 118), a complete ventral carina of the hind trochantellus and vein 1r-m of the hind wing 1.0–1.4 times longer than 1-M (usually shorter than 1-M in other species). Recently, also Platyagathis Turner, 1918, from Western Australia has been correctly synonymised with Disophrys by Stevens et al. (2010).

#### Key to Vietnamese species of the genus Disophrys Foerster

**Table:** 

1.	Temples bulging behind eyes (Fig. 122); hind tibia, hind tarsus and metasoma (except first tergite) black; propodeum yellowish-brown (Fig. 121)	Disophrys maculifera sp. n.
–	Temples narrowed behind eyes (Figs 115, 128, 135); colour of hind tibia, hind tarsus and metasoma variable, if black then propodeum also black	2
2.	Between antennal sockets with a pair of horn-like protuberances (Fig. 135); second submarginal cell of fore wing without ramellus (Fig. 141); vein 1r-m of hind wing about 0.7 times vein 1-M	Disophrys rhinoides sp. n.
–	Between antennal sockets with a pair of lamelliform protuberances or carinae (Figs 115, 128); second submarginal cell of fore wing with a ramellus (Fig. 118) or this vein largely absent (Fig. 134); vein 1r-m of hind wing 0.8–1.4 times vein 1-M	3
3.	Basal half of wings nearly completely dark brown (Fig. 118); notauli widely crenulate medially and posteriorly (Fig. 117); hind trochantellus with distinct ventral carina; hind leg and metasoma black (Fig. 114); vertex punctulate; medially metapleuron vermiculate-rugose	Disophrys erythrocephala Cameron, 1900
–	Basal half of wings yellowish (Fig. 134); notauli smooth medially and posteriorly (Fig. 131); hind trochantellus without a ventral carina, at most angulate or with an obsolete carina; hind leg and metasoma brownish-yellow (Fig. 127; vertex rather coarsely punctate; medially metapleuron rather densely punctate	Disophrys quymanhi sp. n.

#### Disophrys erythrocephala

Cameron, 1900

Figs 114 –120 

##### Description.

###### Variation.

Antennal segments 57–64; vein M+CU of hind wing 0.8–1.0 times as long as vein 1-M; areola of propodeum with 3–4 carinae.

##### Distribution.

NE Vietnam: Hoa Binh, CN Vietnam: Nghe An and S Vietnam: Southern Highlands, Dak Lak. Further known from China (including Taiwan), India, Sri Lanka, Thailand, W. Malaysia and Indonesia (Sumatra, Krakatau, Kangean Islands). Vietnam, Malaysia and Thailand are new records.

**Figure 114.**
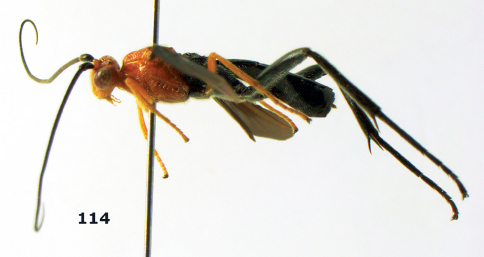
Disophrys erythrocephala Cameron, female, Thailand. Habitus lateral.

**Figures 115–120.**
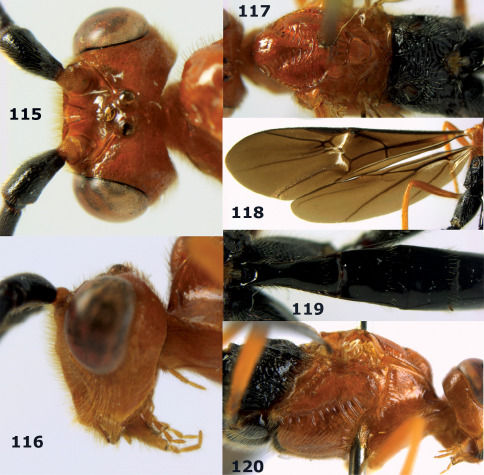
Disophrys erythrocephala Cameron, female, Thailand. **115** head dorsal **116** head lateral **117** mesosoma dorsal **118** wings **119** first-third metasomal tergites dorsal **120** mesosoma lateral.

#### Disophrys maculifera sp. n.

urn:lsid:zoobank.org:act:FA1700DB-1932-43B9-8F4B-6B57AB1613BF

Figs 121 –126 

##### Type material.

Holotype, ♀ (RMNH), “S. Vietnam, Dong Nai, Cat Tien N.P., Malaise trap, c. 100 m, 1–9.x.2005, C. v. Achterberg & R. de Vries, RMNH’05”. Paratypes: 1 ♀ (IEBR), Aga. 175, “Central North Vietnam: Ha Tinh, Huong Son, secondary forest, 5–10.v.2004, Tr.X. Lam”; 3 ♂ (RMNH, IEBR), Aga. 198, Aga. 199, Aga. 200, id., but 25.v.2004; 1 ♂ (IEBR), Aga. 172, id., but 27.v.2004.

##### Diagnosis.

Similar to Disophrys strigata Enderlein, 1920, from Indonesia (Sumatra) because of the completely yellow pterostigma, blackish labrum and presence of a ramellus. Disophrys strigata differs by having the hind leg completely black (Disophrys maculifera has at least hind coxa, trochanter and trochantellus yellowish ventrally), fore coxa with a black patch, middle of mesopleuron and dorsal third of metapleuron and propodeum medially black (brownish-yellow in Disophrys maculifera).

##### Description.

Holotype, ♀, length of body 9.8 mm, of fore wing 11.1 mm.

###### Head.

Antennal segments 55, length of third segment 1.3 times fourth segment, length of third, fourth and penultimate segments 1.9, 1.6 and 1.8 times their width, respectively; scapus robust; length of maxillary palp 0.6 times height of head; in dorsal view length of eye 1.5 times temple; temple rather bulging (Fig. 122); POL:OD:OOL = 7:6:20; face and clypeus shiny, densely setose with sparse fine punctures; frons and vertex smooth; pair of crests between antennal sockets sharp, lamelliform, extended to frons forming x-like shape; lateral carinae from anterior rim of lateral ocelli near to antennal sockets laterally (Fig. 122).

###### Mesosoma.

Length of mesosoma 1.7 times its height; subpronope small and shallow; side of pronotum smooth; area near lateral carina of mesoscutum crenulate; mesoscutum shiny and smooth; medio-posteriorly flat, middle lobe without a pair of distinct shallow grooves; lateral lobes flattened; notauli wide, deep and coarsely crenulate; scutellar sulcus with one carina and 0.8 times as long as dorsal face of scutellum; scutellum slightly convex, shiny and finely punctate, its subposterior crest long and transverse; precoxal sulcus wide and strongly crenulate; mesopleuron below precoxal sulcus narrowly and sparsely finely punctate and above precoxal sulcus shiny and smooth; metapleuron with strong rugae and long setae; propodeum with large areola, costulae, with short basal and long apical longitudinal carinae; propodeal spiracle round, 1.7 times as long as wide, distance between spiracle and lateral carina 1.5 times as long as width of spiracle.

###### Wings.

Fore wing: second submarginal cell narrowed anteriorly, with ramellus 0.8 times as long as vein r (Fig. 126); r:3-SR:SR1 = 8:9:75; 2-SR:3-SR:r-m = 13:9:13. Hind wing: M+CU 1.6 times as long as 1-M (30:19); surroundings of cu-a densely setose.

###### Legs.

Length of hind femur, tibia and basitarsus 3.4, 5.5 and 8.3 times their width, respectively; outer side of hind coxa smooth; hind femur (as remainder of legs) with long and dense setosity; outer side of apex of hind tibia with two equal long pegs; outer hind spur distinctly widened basally and curved apically; length of outer and inner spurs of middle tibia 0.4 and 0.5 times middle basitarsus, respectively; length of outer and inner spurs of hind tibia 0.3 and 0.5 times hind basitarsus, respectively.

###### Metasoma.

First tergite gradually widened apically (Fig. 125), shiny and smooth; length of first tergite 1.6 times its apical width, with three transverse rows of setae apically and slightly depressed laterally; second metasomal suture fine (Fig. 125); ovipositor sheath 0.3 times as long as inner hind tibial spur; ovipositor rather robust, widened basally and gradually curved.

###### Colour.

Brownish-yellow; antenna, apical third of fore wing, parastigma and vein C+SC+R dark brown; stemmaticum, lateral and middle lobes of mesoscutum anteriorly, mesosternum, metasoma and hind leg (but hind coxa, trochantellus and basal half of hind femur yellow); metasoma (but first tergite and first-third sternites yellow) black.

###### Variation.

Antennal segments 54–57; second submarginal cell of fore wing more or less narrowed anteriorly and with a short or rather long ramellus; vein M+CU of hind wing 1.6–1.8 times as long as 1-M; female and male may have hind leg and metasoma entirely black.

##### Distribution.

Central North Vietnam: Ha Tinh and S. Vietnam: Dong Nai.

**Figure 121.**
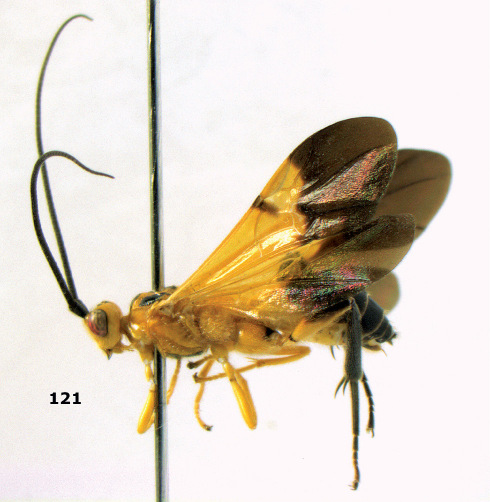
Disophrys maculiferasp. n., female, holotype. Habitus lateral.

**Figures 122–126.**
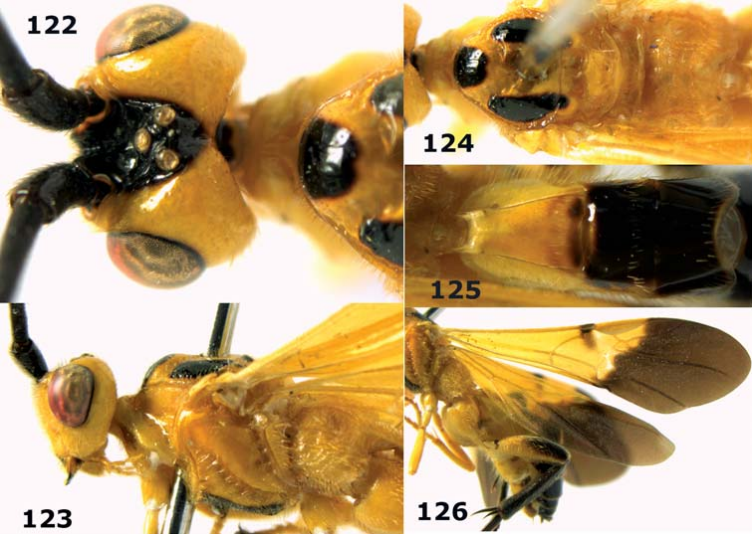
Disophrys maculiferasp. n., female, holotype. **122** head dorsal **123** head andmesosoma lateral **124** mesosoma dorsal **125** first-third metasomal tergites dorsal **126** wings.

##### Biology.

Unknown.

##### Etymology.

From “macula” (Latin for “spot”), and “fero” (Latin for “carry”), because of the black lobes of the mesoscutum.

#### Disophrys quymanhi sp. n.

urn:lsid:zoobank.org:act:256965B7-EC79-46C1-BE18-D1DA93EE2BC3

Figs 127 –134 

##### Type material.

Holotype, ♀ (RMNH), “S. Vietnam: Dông Nai, Cát Tien N.P., Mal. traps, c. 100 m, 1–8.iv.2007, Mai Phu Quy & Nguyen Thanh Manh, RMNH’07”. Paratype: 1 ♀ (IEBR), Aga. 293, “CN Vietnam: Nghe An, Con Cuong, Pu Mat N.P., 19.iv.2006, P.Th. Nhi”.

##### Diagnosis.

Similar to Disophrys subfasciata (Brullé, 1846) from India, but that species has the scapus partly yellowish (entirely black in Disophrys quymanhi), no ramellus (present) and the notauli are deeply impressed (shallow).

##### Description.

Holotype, ♀, length of body 11.5 mm, of fore wing 10.8 mm.

###### Head.

Antennal segments 61, length of third segment 1.8 times fourth segment, length of third, fourth and penultimate segments 2.7, 1.5 and 1.5 times their width, respectively; apical antennal segment 1.5 times as long as penultimate segment; length of maxillary palp 0.8 times height of head; in dorsal view length of eye 3.6 times temple (Fig. 128); POL:OD:OOL = 7:10:25; face coarsely punctate laterally and finely punctate medially; frons depressed near antennal sockets and laterally flat and punctate; pair of high lamelliform flanges between antennal sockets (Fig. 128); lateral carinae from anterior rim of lateral ocelli to antennal sockets laterally and its surroundings distinctly depressed; vertex with rather coarse punctures, but interspaces wider than interspaces; temple finely and spaced punctate.

###### Mesosoma.

Length of mesosoma 1.4 times its height; subpronope large and deep; side of pronotum moderately punctate dorsally, crenulate posteriorly and remainder largely smooth; lateral carina of mesoscutum lamelliform and surroundings smooth; mesoscutum shiny and largely spaced moderately punctate; medio-posteriorly hardly impressed, its middle lobe with a pair of faint shallow longitudinal grooves and lateral lobes weakly convex; notauli shallow and smooth, except for an anterior transverse crest (Fig. 131); scutellar sulcus slightly shorter than dorsal face of scutellum and with 3 carinae; scutellum with some coarse punctures; its subposterior crest strong and transverse, connected to a strong longitudinal carina; side of scutellum crenulate; precoxal sulcus wide, strongly crenulate, but separated from prepectal carina; mesopleuron below precoxal sulcus and dorso-anteriorly rather densely coarsely punctate, remainder of mesopleuron largely smooth; metapleuron with long setae and rather densely coarsely punctate, ventrally with some rugae; propodeum with lateral carina lamelliform, with wide and anteriorly triangular areola; propodeal spiracle elongate, elliptical, 2.5 times as long as wide; distance between spiracle and lateral carina twice as long as width of spiracle.

###### Wings.

Fore wing: second submarginal cell quadrate and with a medium-sized ramellus (Fig. 134); r:3-SR:SR1 = 5:5:32; 2-SR:3-SR:r-m = 9:10:12. Hind wing: M+CU 1.3 times as long as 1-M (Fig. 134); surroundings of cu-a densely setose; 1r-m 0.8 times as long as 1-M.

###### Legs.

Length of hind femur, tibia and basitarsus 3.1, 6.2 and 7.6 times their width, respectively; outer side of hind coxa spaced finely punctate; hind femur (as remainder of leg) with short and dense setosity (Fig. 133); length of outer and inner spurs of middle tibia 0.5 and 0.7 times middle basitarsus, respectively; outer side of apex of hind tibia with three equal pegs; outer hind spur distinctly widened basally, almost straight; length of outer and inner spurs of hind tibia 0.3 and 0.5 times hind basitarsus, respectively; fore and middle tarsi rather slender; hind basitarsus with a distinct carina-like row of setae ventrally.

###### Metasoma.

First tergite smooth, evenly convex (but concave basally) and gradually widened apically (Fig. 130); length of first tergite 2.4 times its apical width; laterope long; second metasomal suture absent; ovipositor sheath elliptical, with long setae and 0.02 times as long as fore wing and about as long as basal width of hind tibia; ovipositor narrow, slightly curved.

###### Colour.

Brownish-yellow; antenna black; apical half of wings, hind tarsus largely and apex of hind tibia narrowly dark brown; stigmal spot large; apical half of pterostigma largely dark brown and narrow area below pterostigma yellow as remainder of wings (Fig. 134).

###### Variation.

Paratype has length of body 9.2 mm and of fore wing 9.3 mm, antenna with 55 segments, apical third of pterostigma dark brown, ramellus obsolescent, length of first tergite 2.6 times its apical width, vein M+CU of hind wing 1.3 times as long as vein 1-M, vein 1r-m of hind wing 0.9 times as long as vein 1-M.

##### Distribution.

S Vietnam: Dông Nai and CN Vietnam: Nghe An.

**Figure 127.**
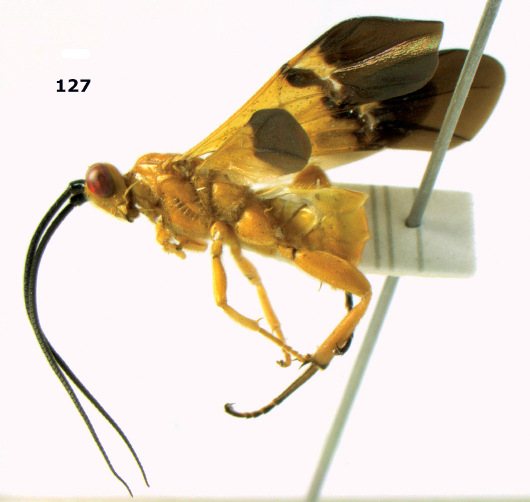
Disophrys quymanhisp. n., female, holotype. Habitus lateral.

**Figures 128–134.**
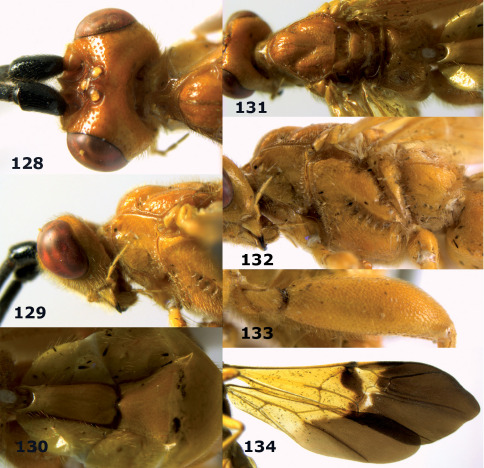
Disophrys quymanhisp. n., female, holotype. **128** head dorsal **129** head lateral **130** first-third metasomal tergites dorsal **131** mesosoma dorsal **132** mesosoma lateral **133** hind femur lateral **134** wings.

##### Biology.

Unknown.

##### Etymology.

Named after both collectors of the holotype: Mai Phu Quy and Nguyen Thanh Manh.

#### Disophrys rhinoides sp. n.

urn:lsid:zoobank.org:act:93E84863-25A0-452A-9A11-69367CED928A

Figs 135 –141 

##### Type material.

Holotype, ♀ (RMNH), Aga. 374, “NE Vietnam: Thai Nguyen, Dai Tu, MT, 360 m, 21°39'N; 105°32'E, 1–10.x.2007, K.D. Long”. Paratypes (8 ♀ + 2 ♂): 1 ♀ (IEBR), Aga. 339, id., but 21°43'N; 105°34'E, 15–20.vi.2007; 2 ♂ (RMNH, IEBR), Aga. 376, id., but 10–20.x.2007 and Aga. 375, id., but 20–30.x.2007; 1 ♀ (RMNH), Aga .067, id., but 19.viii.2000; 1 ♀ (IEBR), Aga. 066, “NE Vietnam: Hoa Binh, Yen Thuy, bushes, 15.vii.2000, Tr.X. Lam”; 2 ♀ (IEBR), Aga. 206 & 212, “Central North Vietnam: Nghe An, Pu Mat N.P., 8.ix.2005, P.Th. Nhi”; 2 ♀ (RMNH), Aga. 206 & 213, id.; 1 ♀ (IEBR), Aga. 182, id., but 22–25.vii.2006, Tr.X. Lam.

##### Diagnosis.

Similar to Disophrys subfasciata (Brullé, 1846) from India, but that species has no horn-like protuberances between the antennal sockets (present in Disophrys rhinoides), the face, mesoscutum and scutellum coarsely punctate (finely punctate), the scutellar sulcus with 3 carinae (with 1 carina), the area below the precoxal sulcus densely punctate (sparsely punctate) and the inner hind tibial spur 0.6 times (0.5 times) as long as the hind basitarsus.

##### Description.

Holotype, ♀, length of body 8.2 mm, of fore wing 7.5 mm.

###### Head.

Antennal segments 52, length of third segment 1.8 times fourth segment, length of third, fourth and penultimate segments 2.5, 1.4 and 2.0 times their width, respectively; apical antennal segment twice times as long as penultimate; length of maxillary palp 0.6 times height of head; in dorsal view length of eye 2.3 times temple (Fig. 136); POL:OD:OOL = 8:5:18; face finely punctate laterally, but rugose-punctate medially; crests between antennal sockets forming pair of large horn-like protuberances (Fig. 136); frons wide, shiny, with rugae; long carina emitted from each base of horn-like protuberances and diverging posteriorly; lateral carinae from anterior rim of lateral ocelli to antennal sockets laterally; vertex with sparse fine punctures.

###### Mesosoma.

Length of mesosoma 1.4 times its height; subpronope large and deep; side of pronotum with sparse fine punctures; area near sharp lateral carina of mesoscutum smooth; mesoscutum shiny and smooth with sparse fine punctures; medio-posteriorly impressed, its middle lobe with a pair of faint shallow longitudinal grooves; notauli narrow, deep and finely crenulate (Fig. 138); scutellar sulcus as long as dorsal face of scutellum; scutellum laterally smooth, slightly narrowed posteriorly, convex anteriorly and depressed posteriorly; subposterior crest transverse, lateral carina incomplete; precoxal sulcus wide and strongly crenulate; mesopleuron below and above precoxal sulcus shiny, with sparse fine punctures, almost smooth; metapleuron short setose and with sparse strong rugae; propodeum with strong lateral carinae, large areola with one transverse carina; propodeal spiracle elongate, elliptical, 2.8 times as long as wide; distance between spiracle and lateral carina twice as long as width of spiracle.

###### Wings.

Fore wing: second submarginal cell quadrate and without ramellus (Fig. 141); r:3-SR:SR1 = 8:10:78; 2-SR:3-SR:r-m = 12:10:13. Hind wing: M+CU 1.3 times as long as 1-M (25: 20); surroundings of cu-a densely setose.

###### Legs.

Length of hind femur, tibia and basitarsus 3.3, 6.1 and 7.8 times their width, respectively; outer side of hind coxa smooth; hind femur (as remainder of leg) with short and dense setosity; length of outer and inner spurs of middle tibia 0.5 and 0.6 times middle basitarsus, respectively; outer side of apex of hind tibia with two equal pegs; outer hind spur distinctly widened basally, almost straight; length of outer and inner spurs of hind tibia 0.3 and 0.5 times hind basitarsus, respectively; fore and middle tarsi rather robust; hind basitarsus with a distinct carina-like row of short setae ventrally.

###### Metasoma.

First tergite smooth and gradually widened apically (Fig. 140); length of first tergite 1.7 times its apical width, without setae apically; laterope long; second metasomal suture absent, ovipositor sheath robust and as long as basal width of hind tibia; ovipositor slender, slightly curved.

###### Colour.

Brownish-yellow; antenna and apical 0.6 of both wings dark brown; hind tarsal segments brown; stigmal spot large and dark brown (Fig. 141); basal two thirds of pterostigma, narrow area below pterostigma and basal 0.4 of wings yellow.

###### Variation.

Antennal segments 52–54; length of body 7.5–9.0 mm and of fore wing 7.5–8.6 mm; vein M+CU of hind wing 1.2–1.5 times as long as 1-M; one female has the body dusty dark brown.

##### Distribution.

NE Vietnam: Thai Nguyen, Hoa Binh and Central North Vietnam: Nghe An.

**Figure 135.**
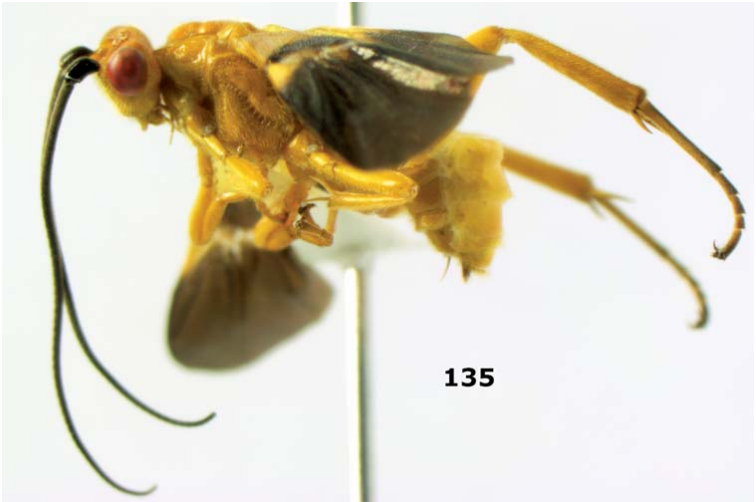
Disophrys rhinoidessp. n., female, holotype. Habitus lateral.

**Figures 136–141.**
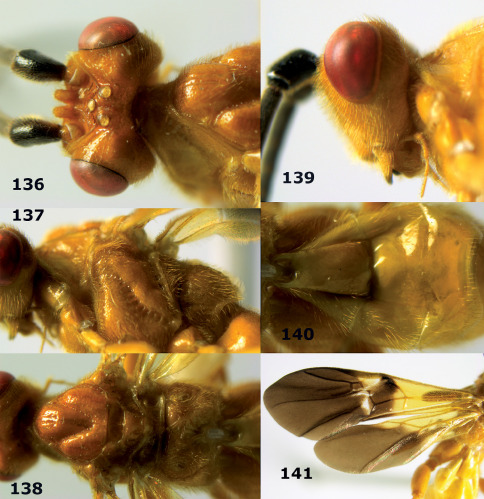
Disophrys rhinoidessp. n., female, holotype. **136** head dorsal **137** mesosoma lateral **138** mesosoma dorsal **139** head lateral **140** first-third metasomal tergites dorsal **141** wings.

##### Biology.

Unknown.

##### Etymology.

From “rhinos” (Greek for “nose”), because of the small nose-like projections between the antennal sockets.

### Genus Earinus

Wesmael, 1837

#### Key to Vietnamese species of the genus Earinus Wesmael

**Table:** 

1.	Malar space about 2.6 times as long as basal width of mandible; vein M+CU of hind wing subequal to vein 1-M; scutellar sulcus 0.7 times as long as dorsal part of scutellum (Fig. 153); propodeum with large areola and transverse carinae; head comparatively robust (Fig. 158); pterostigma robust and marginal cell narrow (Figs 151, 155)	Earinus brevistigmus sp. n.
–	Malar space twice as long as basal width of mandible; vein M+CU of hind wing longer, 1.2 times as long as vein 1-M; scutellar sulcus 0.2 times as long as dorsal part of scutellum (Fig. 144); propodeum without areola, but with two parallel longitudinal carinae (Fig. 144); head less robust (Fig. 149); pterostigma less robust and marginal cell wider (Figs 142, 145)	Earinus aurantius sp. n.

#### Earinus aurantius sp. n.

urn:lsid:zoobank.org:act:78C9CD4C-E320-4024-B7FE-F629C30492B8

Figs 142 –150 

##### Type material.

Holotype, ♀ (RMNH), “N.W. Vietnam: Tonkin, Hoang Lien N.R., 15 km W [of] Sa Pa, c. 1900 m, 15–21.x.1999, Malaise traps, C. v. Achterberg, RMNH’99”.

##### Diagnosis.

The new species is similar to Earinus burmensis Gupta & Bhat, 1974, from Myanmar, but differs by having the first tergite 1.3 times as long as its apical width (Earinus burmensis: twice); mesoscutum medio-apically and scutellum orange brown; hind tarsus black (burmensis: only apices of hind tarsal segments) and tibia with dark brown ring (burmensis: absent).

##### Description.

Holotype, ♀, length of body 6.6 mm, of fore wing 6.0 mm, of ovipositor sheath 6.0 mm.

###### Head.

Antennal segments 34; length of third segment 1.1 times fourth segment, length of third, fourth and penultimate segments 3.8, 3.4 and 1.7 times their width, respectively; maxillary palp as long as height of head; length of malar space twice as long as basal width of mandible; in dorsal view length of eye 4.2 times temple (Fig. 149); POL:OD:OOL = 7:3:6; face shiny, nearly smooth with very sparse minute punctures; between antennal sockets two carinae and a short groove (Fig. 149); frons, vertex and temple shiny and smooth (Fig. 149).

###### Mesosoma.

Length of mesosoma 1.6 times its height; subpronope large and deep; pronotum shiny and largely smooth; area near lateral carina of mesoscutum smooth anteriorly and crenulate posteriorly; notauli completely absent; mesoscutum shiny with sparse fine punctures and setae, slightly depressed medio-posteriorly; notauli completely absent; scutellar sulcus short, 0.2 times as long as scutellum and with 4 carinae; scutellum distinctly narrowed posteriorly, sparsely finely punctate; precoxal sulcus absent; mesopleuron shiny, smooth; metapleuron nearly smooth with sparse punctures; propodeum with two medial parallel carinae from base to apex of propodeum (Fig. 144).

###### Wings.

Fore wing: second submarginal cell pentagonal (Fig. 146); vein SR1 straight; r:3-SR: SR1 = 4:2:53; vein 1-SR+M completely sclerotized and pigmented. Hind wing: vein M+CU 1.2 times as long as vein 1-M.

###### Legs.

Length of hind femur, tibia and basitarsus 3.8, 8.2 and 9.5 times their width, respectively; hind femur (as remainder of legs) with short setae (Fig. 147); length of outer and inner spur of middle tibia 0.4 and 0.5 times middle basitarsus, respectively; outer apex of middle tibia with a row of 6 pegs and 2 pegs at apex; ventral side of middle basitarsus with sparse unusual thick setae; length of outer and inner spur of hind tibia 0.2 and 0.4 times hind basitarsus; tarsal claws without lobe; outer side of hind tibia with sparse unusual thick setae.

###### Metasoma.

First tergite widened apically, slightly depressed laterally, with short medial carina and strongly convergent dorsal carinae; length of first tergite 1.3 times its apical width (Fig. 145); second tergite 1.1 times longer than third tergite (Fig. 145); metasoma shiny and smooth but first tergite coriaceous basally; ovipositor sheath as long as fore wing.

###### Colour.

Black; palpi, mandible, tegula, legs, but subbasal ring and apex of hind tibia and hind tarsus dark brown; mesoscutum medio-apically and scutellum orange brown; wing membrane infuscate.

##### Distribution.

NW Vietnam: Lao Cai.

**Figure 142.**
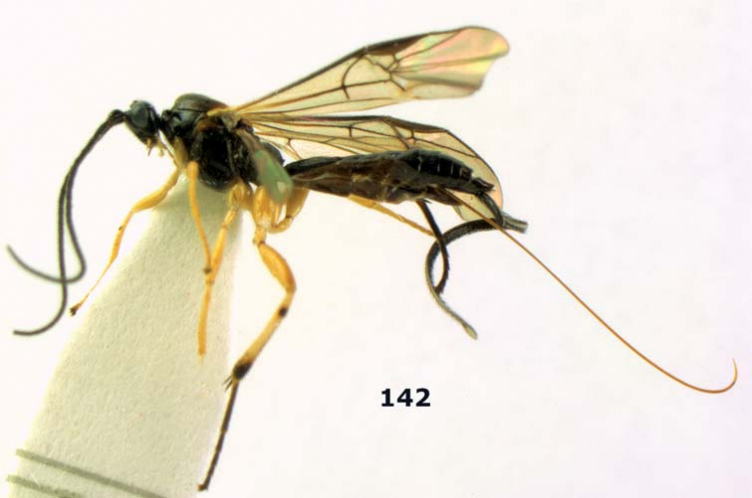
Earinus aurantius sp. n., female, holotype. Habitus lateral.

**Figures 143–150.**
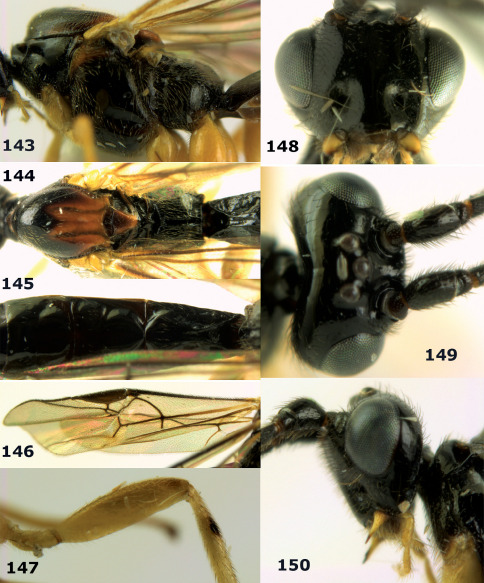
Earinus aurantius sp. n., female, holotype. **143** mesosoma lateral **144** mesosoma dorsal **145** first-third metasomal tergites dorsal **146** wings **147** hind femur lateral **148** head anterior **149** head dorsal **150** head lateral.

##### Biology.

Unknown.

##### Etymology.

From “aurantium” (Latin for “orange”), because of the orange brown scutellum.

#### Earinus brevistigmus sp. n.

urn:lsid:zoobank.org:act:868A3D2F-7D77-41F4-BE73-0BF4A014E5D5

Figs 151 –159 

##### Type material.

Holotype, ♀ (RMNH), “S. Vietnam: Lam Dong, Bidoup Nuiba N.P., n[ea]r Da Lat, Mal. traps 1–12, 1650–1700 m, 11–19.x.2005, C. v. Achterberg & R. de Vries, RMNH’05”.

##### Diagnosis.

The new species is similar to Earinus burmensis Gupta & Bhat, 1974, from Myanmar, but differs by having the malar space 2.6 times as long as basal width of mandible (Earinus burmensis:1.8 times); first metasomal tergite 1.2 times as long as its apical width (burmensis: twice); precoxal sulcus absent (burmensis:with adeep depression posteriorly)). The new species is also similar to Earinus bicolor Chou & Sharkey, 1989, from China, but differs by having the metasoma black (bicolor:yellowish red); POL 0.9 times as long as OOL (bicolor:0.7 times) and face distinctly punctate (bicolor:sparsely minutely punctate).

##### Description.

Holotype, ♀, length of body 3.7 mm, of fore wing 3.3 mm, of ovipositor sheath 0.9 mm.

###### Head.

Antennal segments 32, length of third segment 1.5 times fourth segment, length of third, fourth and penultimate segments 3.7, 2.5 and 1.5 times their width, respectively; maxillary palp as long as height of head; length of malar space 2.6 times basal width of mandible; in dorsal view length of eye 2.3 times temple (Fig. 158); POL:OD:OOL = 8:4:9; face punctate; frons flat, shiny and smooth, between antennal sockets with a deep groove (Fig. 158); vertex and temple shiny with very sparse minute punctures.

###### Mesosoma.

Length of mesosoma 1.5 times its height; subpronope medium-sized; pronotum shiny and smooth; area near lateral carina of mesoscutum crenulate; mesoscutum shiny with sparse fine punctures and setae, slightly depressed medio-posteriorly; notauli completely absent; scutellar sulcus 0.7 times as long as scutellum and with 2 carinae; scutellum slightly narrowed posteriorly, sparsely finely punctate; precoxal sulcus absent; mesopleuron shiny, smooth dorsally and with sparse fine punctures ventrally; metapleuron smooth; propodeum with long areola and costulae present, area inside areola with transverse carinae.

###### Wings.

Fore wing: second submarginal cell triangular (Fig. 155); vein SR1 straight; r:3-SR: SR1 = 2:1:50, r:2-SR:r-m = 2:13:12; vein 1-SR+M medially unsclerotized and only pigmented. Hind wing: vein M+CU subequal to vein 1-M.

###### Legs.

Length of hind femur, tibia and basitarsus 4.0, 5.5 and 10.0 times their width, respectively; hind femur (as remainder of legs) with short setae (Fig. 156); length of outer and inner spur of middle tibia 0.4 and 0.5 times middle basitarsus, respectively; outer apex of hind tibia with a cluster of 7 pegs; length of outer and inner spur of hind tibia 0.4 and 0.5 times hind basitarsus; tarsal claws without lobe.

###### Metasoma.

**Figure 151.**
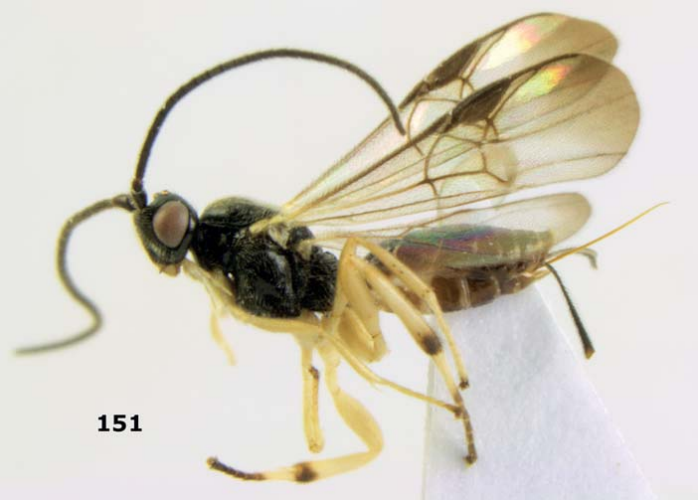
Earinus brevistigmus sp. n., female, holotype. Habitus lateral.

First tergite widened apically, slightly depressed laterally, with short dorsal carinae basally; length of first tergite 1.2 times its apical width (Fig. 154); second metasomal 1.2 times as long as third tergite (Fig. 154); metasoma shiny and smooth but basal half of first tergite faintly striate; ovipositor sheath 0.9 times as long as fore wing, widened apically (Fig. 151).

###### Colour.

Black; clypeus, palpi, galea, tegulae and legs ivory or pale yellowish, but tarsal claws, apex of hind tibia and second-fifth segments of hind tarsus dark brown; wing membrane infuscate.

##### Distribution.

S Vietnam: Lam Dong.

**Figures 152–159.**
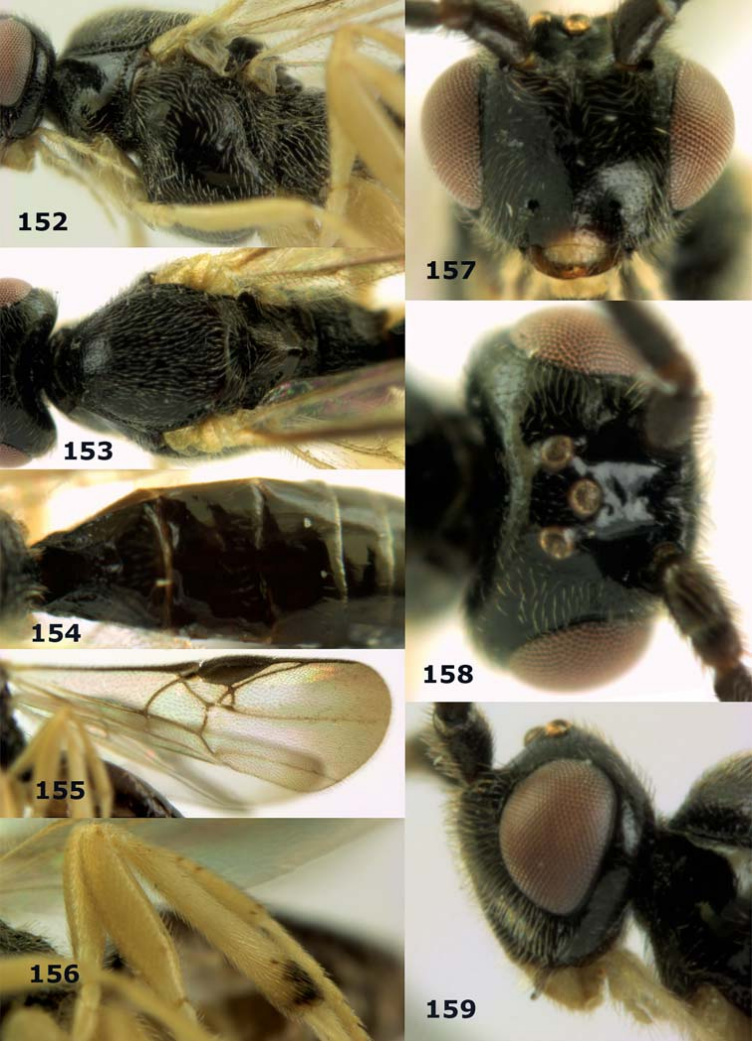
Earinus brevistigmus sp. n., female, holotype. **152** mesosoma lateral **153** mesosoma dorsal **154** first-third metasomal tergites dorsal **155** wings **156** hind femur and tibia lateral **157** head anterior **158** head dorsal **159** head lateral.

##### Biology.

Unknown.

##### Etymology.

From “brevis” (Latin for “short”), and “stigma” (Greek for “mark”), because of the short pterostigma.

### Genus Euagathis

Szépligeti, 1900

#### Key to Vietnamese species of the genus Euagathis Szépligeti

**Table:** 

1.	Scutellum strongly protruding (Fig. 193); propodeum distinctly depressed and without carinae medio-posteriorly, with large postero-dorsal lamelliform protuberance and laterally with long setae (Fig. 194); metapleuron coarsely rugose; area below precoxal sulcus coarsely punctate; wing of males dark brown, of females paler brown and with basal half of fore wing more or less with yellowish tinge; hind femur of females brown or black; stigmal spot absent or near so (Fig. 192); lateral border of scutellar sulcus more or less protruding	Euagathis ophippium (Cameron, 1899)
–	Scutellum flat to weakly convex (Figs 174, 184, 188); propodeum without distinct depression and with carinae medio-posteriorly (Figs 174, 184), but rugose in Euagathis dravida (Fig. 171), atmost with a short postero-dorsal flange and with moderately short to medium-sized setae laterally; metapleuron more or less punctate medially; area below precoxal sulcus less coarsely punctate or smooth; wings usually with distinct stigmal spot (Fig. 173) or with spot being part of a dark band and wings apically infuscate or dark brown (Fig. 163; but intermediate in Euagathis dravida: Fig. 169); hind femur of female entirely or partly yellowish brown (but black in Euagathis dravida); lateral border of scutellar sulcus not protruding	2
2.	Notauli coarsely crenulate or at least subposteriorly so; precoxal sulcus widely crenulate; propodeal spiracle comparatively large ; vein M+CU of hind wing comparatively long; [fore tarsus and hind femur robust (cf. Fig. 191); lateral ocelli small, POL about 1.7 times OOL]	Euagathis robusta van Achterberg & Chen, 2002
–	Notauli smooth or slightly crenulate subposteriorly (Figs 161, 164, 184); precoxal sulcus less crenulate; propodeal spiracle comparatively small; vein M+CU of hind wing rather short (Fig. 173)	3
3.	Lateral lobes of mesoscutum distinctly convex posteriorly and medially distinctly punctate (Fig. 184, but sometimes sparsely so); surroundings of vein cu-a of hind wing at least partly glabrous; metapleuron densely or finely punctate submedially; first metasomal tergite 1.7–2.1 times as long as apical width (Fig. 184); area below precoxal sulcus distinctly punctate	4
–	Lateral lobes of mesoscutum weakly convex or flattened posteriorly and lobes submedially largely smooth (fig. 174); surroundings of vein cu-a of hind wing setose; metapleuron sparsely punctate medially; first metasomal tergite 1.5–1.6 times as long as apical width (Figs 165, 168, 175), if about twice as long as apical width (Euagathis parallela: Fig. 197) then first tergite with sublateral depressions; area bellow precoxal sulcus smooth, punctate or sparsely punctate	6
4.	Head and mesosoma completely black (Fig. 169); stigmal spot of fore wing absent (Fig. 169); vein M+CU of hind wing about 0.3 times as long as 1-M	Euagathis dravida Bhat & Gupta, 1977
–	Head (except more or less dorsally) and antero-ventrally mesosoma yellowish-brown (Fig. 182); stigmal spot of fore wing medium-sized to large (Fig. 182); vein M+CU of hind wing about 0.5 times as long as 1-M	5
5.	Scutellum rounded anteriorly and gradually elevated and without carina (Fig. 161); pterostigma at least narrowly dark brown apically (Fig. 160); precoxal sulcus with short rugae and crenulae anteriorly (Fig. 160); antenna of female hardly tapering apically, 10th antennal segment from apex 0.9–1.1 times as long as wide and subapical segments submoniliform; lateral lobes of mesoscutum sparsely punctate, with distinct interspaces; lateral lobes without oblique rugae near medio-posterior area of mesoscutum (Fig. 161); fore wing of female without distinct apical infuscation (Fig. 160); dorsal third of metapleuron distinctly and densely punctate	Euagathis abbotti (Ashmead, 1900)
–	Scutellum angulate anteriorly and more or less carinate (Fig. 184); pterostigma yellow apically (Fig. 182); precoxal sulcus with some long rugae and crenulae anteriorly (Fig. 184); antenna of female distinctly tapering apically, 10th antennal segment from apex 1.3–1.4 times as long as wide and subapical segments normal; lateral lobes of mesoscutum usually densely punctate, at least partly without distinct interspaces; fore wing of female with distinct apical infuscation (Fig. 182), rarely reduced; lateral lobes frequently with oblique rugae near medio-posterior area of mesoscutum; dorsal third of metapleuron weakly and sparsely punctate	Euagathis forticarinata (Cameron, 1899)
6.	Second metasomal suture comparatively widely impressed, distinct (Figs 165, 197); first metasomal tergite of female longitudinally depressed sublaterally near middle of tergite (Figs 165, 197); costulae of propodeum largely absent (Fig. 164); laterally temples straight; second-fourth segments of middle tarsus of female comparatively slender (Fig. 195); vertex and frons yellowish	7
–	Second metasomal suture absent (Figs 168, 175, 187); first metasomal tergite of female without or with weak sublateral depressions (Figs 168, 175); costulae of propodeum present (Figs 167, 174); laterally temples slightly concave (figs); second-fourth segments of middle tarsus of female comparatively robust (Figs 166, 191); vertex and frons black, but yellowish in Euagathis maculipennis	8
7.	First metasomal tergite distinctly widened apically, 1.0–1.4 times as long as its apical width (Fig. 165); anterior half of precoxal sulcus distinctly crenulate; middle lobe of mesoscutum posteriorly distinctly differentiated from rest of mesoscutum (Fig. 164); second tergite largely yellowish brown or blackish (Fig. 165); apex of hind tibia less convex	Euagathis borneoensis Szépligeti, 1902
–	First tergite nearly parallel-sided, about twice as long as its apical width (Fig. 197); anterior half of precoxal sulcus smooth; middle lobe of mesoscutum posteriorly weakly differentiated from rest of mesoscutum; second tergite ivory laterally and medially dark brown (Fig. 197); apex of hind tibia more convex	Euagathis parallela van Achterberg & Chen, 2002
8.	Apical third of wings golden yellow (Figs 172, 173); scapus brownish-yellow; areola of propodeum comparatively wide (Fig. 174); head conspicuously setose (Figs 179–181)	Euagathis flavosoma sp. n.
–	Apical third of wings dark brown (Figs 166, 188); scapus black (but more or less brown in Euagathis maculipennis); areola of propodeum narrow (Fig. 167); head less setose	9
9.	Occipital flange comparatively narrow, less protruding than basal width of mandible; second suture of metasoma finely impressed (Fig. 187); malar space comparatively short (Fig. 186); marginal cell of fore wing subhyaline; vertex, frons and stemmaticum yellowish-brown	Euagathis maculipennis (Brullé, 1846)
–	Occipital flange comparatively wide, more protruding than basal width of mandible (Fig. 188; second suture of metasoma absent (Fig. 189); malar space long (Fig. 188); marginal cell of fore wing dark brown (Fig. 188); vertex, frons and stemmaticum often black	10
10.	Metapleuron coarsely and densely punctate; stemmaticum and vertex yellowish-brown; hind basitarsus yellowish-brown (Fig. 188); second-fourth segments of fore tarsus of ♀ robust and distinctly widened apically (Fig. 190)	Euagathis mayunae van Achterberg & Chen, 2002
–	Metapleuron finely and sparsely punctate; stemmaticum and vertex black; hind basitarsus dark brown or blackish (Fig. 166); second-fourth segments of fore tarsus of ♀ less robust and only slightly widened apically (Fig. 166)	Euagathis chinensis (Holmgren, 1868)

#### Euagathis abbotti

(Ashmead, 1900)

Figs 160–162 

##### Diagnosis.

A very variable species, especially in respect to its coloration. Melanistic males are similar to melanistic males of Euagathis forticarinata (Cameron), but the latter have the scutellum angulate anteriorly, the mesoscutum and the head dorsally never more or less darkened and the mesoscutum and mesopleuron distinctly sculptured. Variation in the series from South Vietnam: second submarginal cell of fore wing with or without a short ramellus; apical antennal segment 1.5–1.7 times as long as penultimate segment; vein M+CU of hind wing 0.5 times as long as 1-M; first tergite 1.8–2.0 times as long as its apical width; hind tibia dark brown or yellowish-brown apically.

##### Distribution.

S Vietnam: Dong Nai, Kon Tum. Outside Vietnam known from Brunei; Indonesia; (Java; Sumatra), Malaysia (Sabah) and Thailand.

**Figures 160–162.**
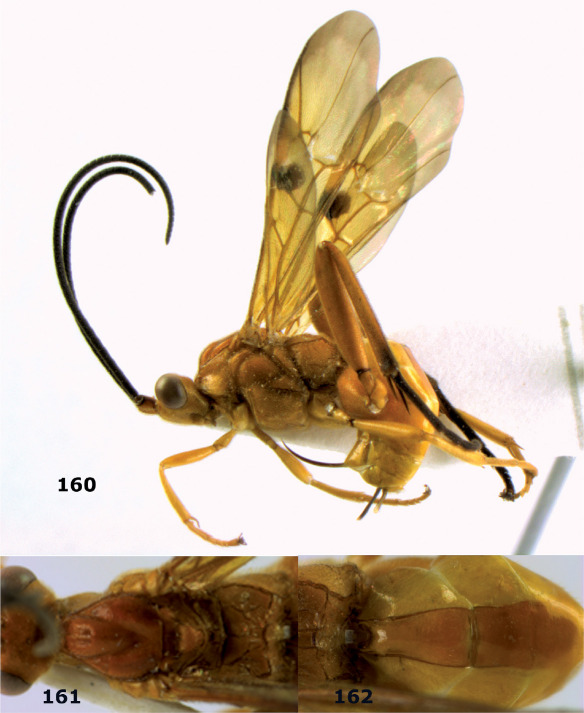
Euagathis abbotti (Ashmead), female, Cat Tien National Park. **160** habitus lateral **161** mesosoma dorsal **162** first-third metasomal tergites dorsal.

##### Notes.

As redefined in this paper the following new synonyms have to be added: Euagathis nigrithorax Bhat & Gupta, 1977, from India and from Indonesia (Sumatra): Euagathis variabilis Enderlein, 1920; Euagathis variabilis var. tibialis Enderlein, 1920; Euagathis variabilis var. melanopleura Enderlein, 1920; Euagathis variabilis var. sucarandana Enderlein, 1920.

#### Euagathis borneoensis

Szépligeti, 1902

Figs 163–165 

##### Distribution.

NE Vietnam: Ha Noi, Vinh Phuc. Outside Vietnam known from China (Fujian; Guangxi; Hunan; Jiangsu; Sichuan; Yunnan; Zhejiang); India and Indonesia (Java; Kalimantan; Sumatra).

**Figures 163–165.**
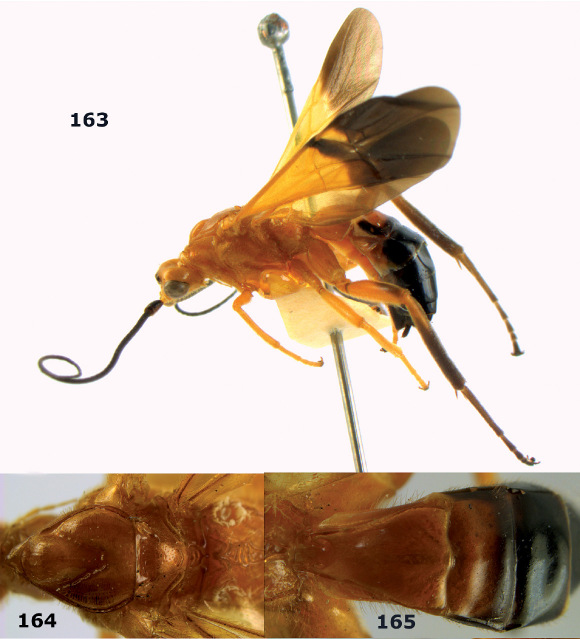
Euagathis borneoensis Szépligeti, female, Gia Lam. **163** habitus lateral **164** mesosoma dorsal **165** first-third metasomal tergites dorsal.

#### Euagathis chinensis

(Holmgren, 1868)

Figs 166–168 

##### Distribution.

NE Vietnam: Ha Noi, Ha Tay, Hoa Binh, Thai Nguyen, Vinh Phuc; CN Vietnam: Nghe An and C Vietnam: Thua Thien-Hué. Outside Vietnam known from China (Anhui; Fujian; Guangdong; Guangxi; Guizhou; Hainan Island; Hong Kong; Hunan; Jiangsu; Jiangxi; Qinghai; Sichuan; Taiwan; Yunnan; Zhejiang); India; Indonesia (Java; Sumatra); Japan; Laos; W. Malaysia; Myanmar; Nepal; Singapore; Sri Lanka and Thailand.

**Figures 166–168.**
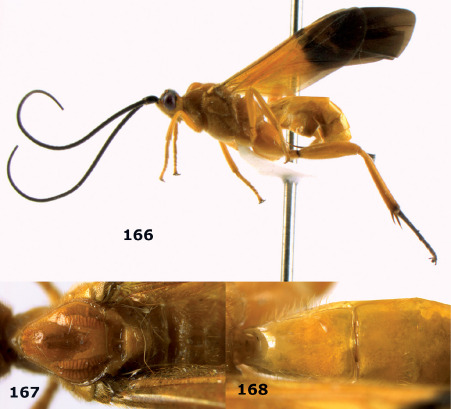
Euagathis chinensis (Holmgren), female, Dai Tu. **166** habitus lateral **167** mesosoma dorsal **168** first-third metasomal tergites dorsal.

#### Euagathis dravida

Bhat & Gupta, 1977

Figs 169–171 

##### Distribution.

NE Vietnam: Hoa Binh; CN Vietnam: Ha Tinh. Outside Vietnam known from India.

**Figures 169–171.**
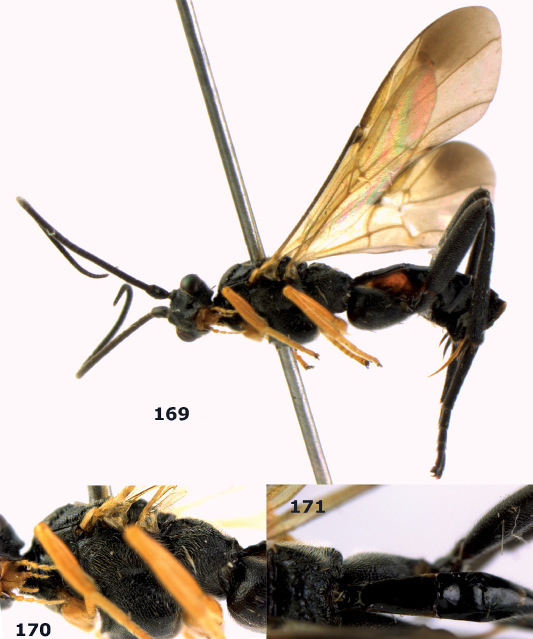
Euagathis dravida Bhat & Gupta, female, Thung Cuu. **169** habitus lateral **170** mesosoma lateral **171** first-third metasomal tergites dorsal.

#### Euagathis flavosoma sp. n.

urn:lsid:zoobank.org:act:112F48E5-484A-43B0-A5E0-8230E1ED4B85

Figs 172 –181 

##### Type material.

Holotype, ♀ (IEBR), Aga. 264, “S. Vietnam: Tay Ninh, Lo Go-Xa Mat N.P., forest, 7.xi.2003, K.D. Long”.

##### Diagnosis.

This species is close to Euagathis leptocera Cameron, 1907, from East Malaysia, but the new species differs by having the third segment of fore tarsus about as long as wide (Fig. 177; Euagathis leptocera: about 1.5 times longer than wide), a short ramellus (Euagathis leptocera: hardly developed); the metasoma reddish-yellow (leptocera: yellowish-brown) and the scutellum rugose-punctate (leptocera: coarsely punctate). The new species is also similar to Euagathis forticarinata (Cameron), but it differs by having vein M+CU about 0.4 times vein 1-M (Euagathis forticarinata: about 0.5 times); first tergite of female 1.3 times as long as apical width (forticarinata: 1.5–1.9 times) and second-fourth middle tarsal segments robust (forticarinata: moderately slender).

##### Description.

Holotype, ♀, length of body 10.1 mm, of fore wing 11.2 mm.

###### Head.

Antennal segments 59, length of third segment 1.2 times fourth segment, length of third, fourth and penultimate segments 2.1, 1.8 and 1.7 times their width, respectively; length of maxillary palp 1.3 times height of head; in dorsal view length of eye 1.3 times temple; temple concave laterally and distinctly narrowed (Fig. 180); POL:OD:OOL = 7:6:19; face and vertex smooth; malar space densely setose (Fig. 179); pair of crests between antennal sockets convergent, and higher than rim of antennal sockets.

###### Mesosoma.

Length of mesosoma 1.4 times its height; subpronope large and deep; side of pronotum shiny and smooth; area near lateral carina of mesoscutum smooth; side of mesoscutum smooth, middle lobe with sparse fine punctures anteriorly; medio-posteriorly flat, slightly impressed, shiny and smooth; notauli shallow and smooth (Fig. 174); scutellar sulcus long, 1.1 times as long as scutellum (17:15); scutellum rugose-punctate; subposterior crest strong and curved (Fig. 174); mesopleuron below precoxal sulcus with sparse distinct punctures, and above sulcus almost smooth; precoxal sulcus wide, shallow and coarsely crenulate (Fig. 178), 3 anterior crenulae connected to prepectal carina; metapleuron punctate anteriorly and rugose-punctate posteriorly; propodeum with strong carina basally, large areola and costulae present; propodeal spiracle elongate, subelliptical, 2.3 times as long as wide; distance between spiracle and lateral carina 1.3 times as long as width of spiracle.

###### Wings.

Fore wing: second submarginal cell pentagonal, distinctly narrowed anteriorly, with short ramellus (Fig. 173); r:3-SR:SR1 = 13:5:125; 2-SR:3-SR:r-m = 24:5:21. Hind wing: vein M+CU rather short, 0.4 times as long as 1-M (40:90); surroundings of cu-a sparsely short setose.

###### Legs.

Length of hind femur, tibia and basitarsus 4.0, 7.3 and 8.8 times their width, respectively; hind femur (as remainder of legs) with short and dense setosity (Fig. 176); outer side of apex of hind tibia with 3 pegs; length of outer and inner spurs of middle tibia 0.3, and 0.5 times their basitarsus, respectively; length of outer and inner spurs of hind tibia 0.3 and 0.6 times their basitarsus; fore and middle tarsi robust (Fig. 177); hind spurs distinctly widened basally; first-third hind tarsal segments with a carina-like row of special short strong setae ventrally.

###### Metasoma.

First tergite rather short, strongly widened apically (Fig. 175), smooth; length of first tergite 1.3 times its apical width, without sublateral depressions; second metasomal suture absent (Fig. 175); ovipositor sheath short, truncate apically, 0.6 times as long as inner hind tibial spur.

###### Colour.

Brownish-yellow; antenna yellowish-brown, but scapus brownish-yellow; fore wing yellow, with a dark brown stigmal spot (Fig. 173); veins 2-SR+M and basal third of 1-M dark brown; hind wing yellow, slightly infuscate apically.

##### Distribution.

S Vietnam: Tay Ninh.

**Figure 172.**
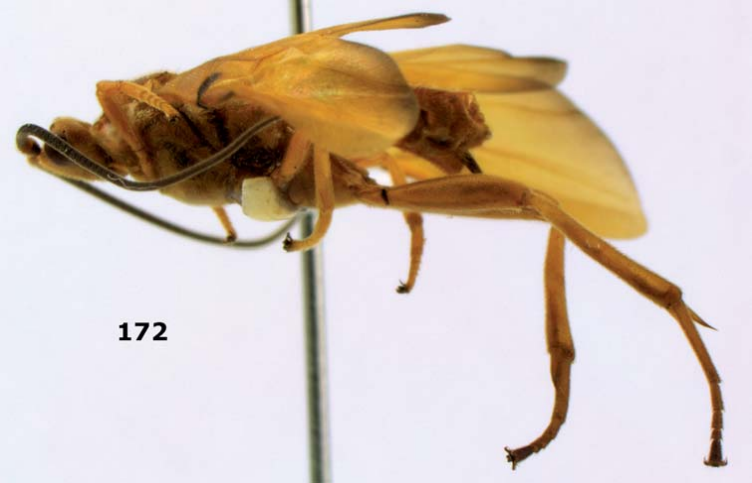
Euagathis flavosoma sp. n., female, holotype. Habitus lateral.

**Figures 173–181.**
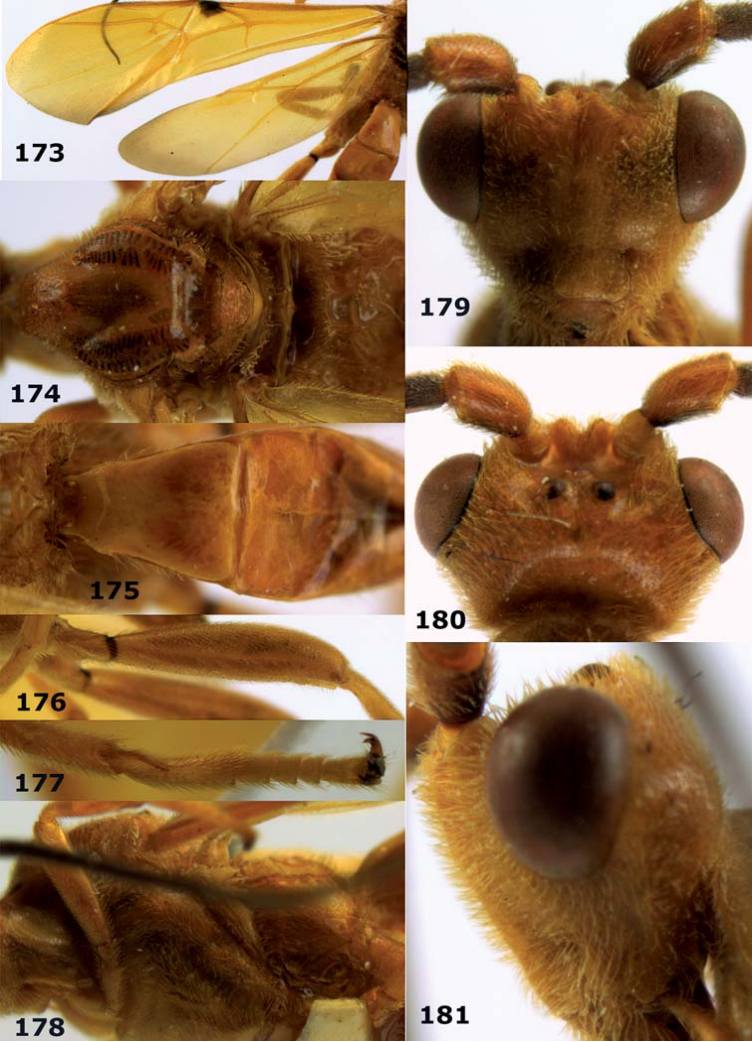
Euagathis flavosoma sp. n., female, holotype. **173** wings **174** mesosoma dorsal **175** first-third metasomal tergites dorsal **176** hind femur lateral **177** fore tarsus **178** mesosoma lateral **179** head anterior **180** head dorsal **181** head lateral.

##### Biology.

Unknown.

##### Etymology.

From “flavus” (Latin for “yellow”), and “soma” (Greek for “body”), because of the yellow body.

#### Euagathis forticarinata

(Cameron, 1899)

Figs 182–184 

##### Notes.

Here belong the following new synonyms: Euagathis jinshanensis Chen & Yang, 2006 (holotype is a melanistic male from Fujian (China) with darkened posterior part of mesosoma and legs and not a female as indicated) and Euagathis sharkeyi Chen & Yang, 2006 (holotype is female from Fujian (China) with distinctly convex and sculptured mesoscutal lobes and pale hind leg). Also belongs here part of the series of Euagathis penetrans sensu Chen & Yang, 2006, from China (not Zelodia penetrans (Smith, 1860) from Wallacea).

##### Distribution.

Vietnam in NW Vietnam: Son La, Yen Bai; NE Vietnam: Bac Kan, Ha Giang, Hanoi, Ha Tay, Hoa Binh, Ninh Binh, Thai Nguyen, Vinh Phuc; CN Vietnam: Ha Tinh, Nghe An; C Vietnam: Quang Nam, Quang Tri, Thua Thie-Hue and S Vietnam: Kien Giang. Outside Vietnam known from China (Guangdong; Guangxi; Guizhou; Hainan Island; Hong Kong; -Hubei; Jiangxi; Macau; Sichuan; Taiwan; Yunnan; Zhejiang); India; Indonesia (Java; Sulawesi; Sumatra; West Lesser Sundas); W. Malaysia; Nepal; Philippines (Luzon); Sri Lanka and Thailand.

**Figures 182–184.**
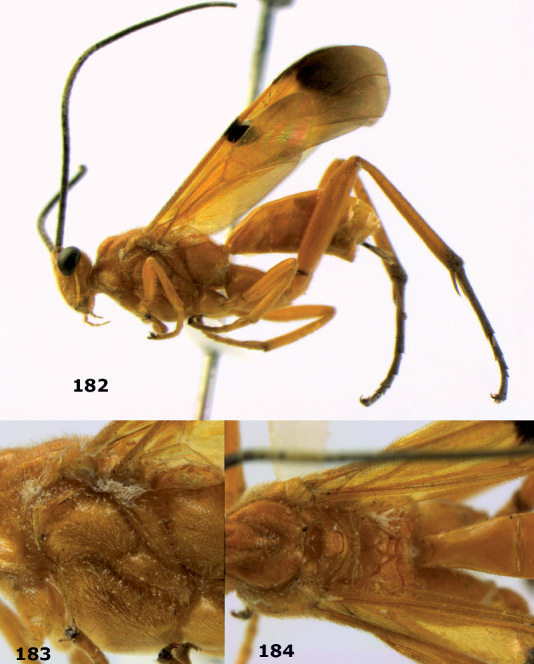
Euagathis forticarinata (Cameron), female, Cao Phong. **182** habitus lateral **183** mesosoma lateral **184** mesosoma and first tergite dorsal.

#### Euagathis maculipennis

(Brullé, 1846)

Figs 185–187 

##### Distribution.

CN Vietnam: Nghe An, Ha Tinh. Outside Vietnam known from India and China (Yunnan).

**Figures 185–187.**
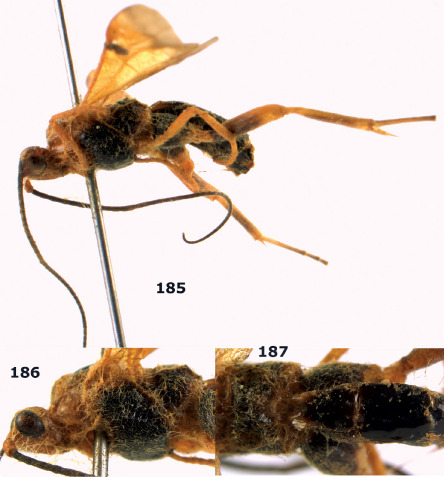
Euagathis maculipennis (Brullé), male, China. **185** habitus lateral **186** mesosoma lateral **187** propodeum and first tergite dorsal.

#### Euagathis mayunae

van Achterberg & Chen, 2002

Figs 188–191 

##### Distribution.

NE Vietnam: Hai Phong (Cat Ba Island). New record for Vietnam. Outside Vietnam known from China (Fujian).

**Figures 188–191.**
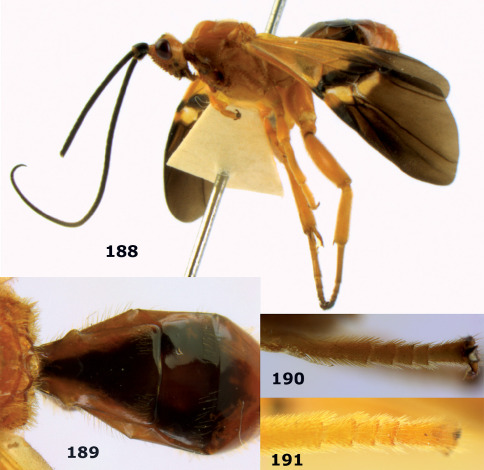
Euagathis mayunae van Achterberg & Chen, female, Cat Ba Island. **188** habitus lateral **189** first tergite dorsal **190** fore tarsus **191** middle tarsus.

#### Euagathis ophippium

(Cameron, 1899)

Figs 192–194 

##### Distribution.

NE Vietnam: Hoa Binh. Outside Vietnam known from China (Beijing; Fujian; Guangxi; Guizhou; Hunan; Jiangsu; Jilin; Shandong; Yunnan; Zhejiang); India; Japan; Korea; Nepal and Russia (Far East).

**Figures 192–194.**
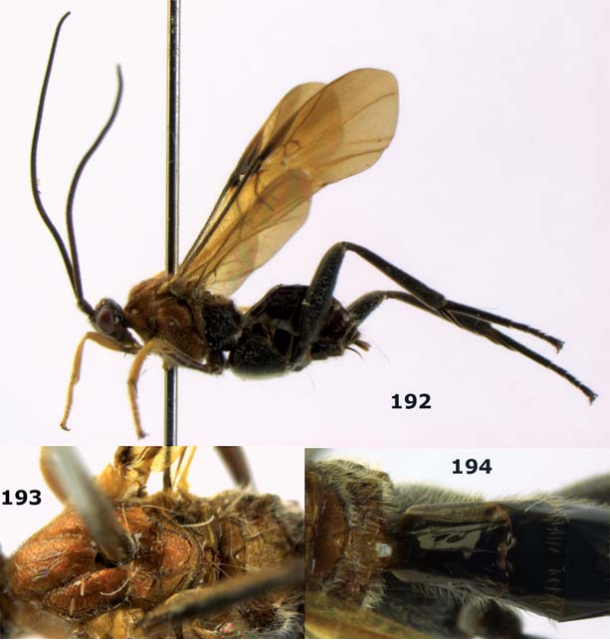
Euagathis ophippium (Cameron), female, Cuc Phuong National Park. **192** habitus lateral **193** mesosoma dorsal **194** first-third metasomal tergites dorsal.

#### Euagathis parallela

van Achterberg, 2002

Figs 195–197 

##### Distribution.

NW Vietnam: Lao Cai. Outside Vietnam known from China (Fujian).

**Figures 195–197.**
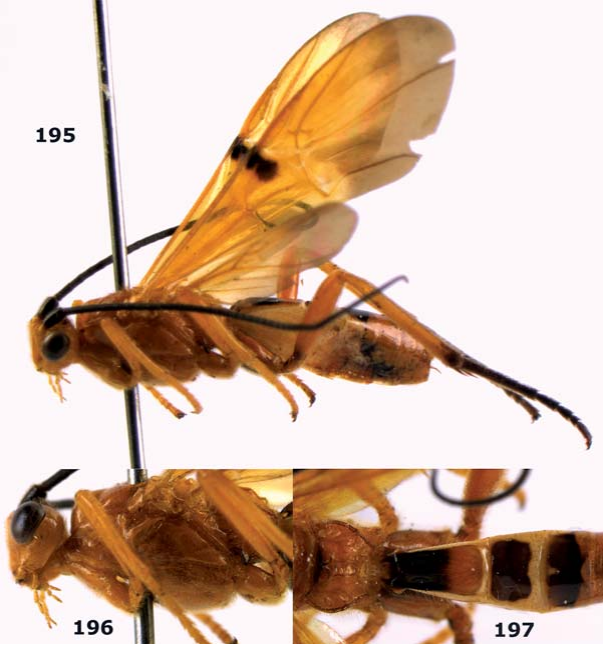
Euagathis parallela van Achterberg, female, holotype. **195** habitus lateral **196** head and mesosoma lateral **197** first-third metasomal tergites dorsal.

#### Euagathis robusta

van Achterberg & Chen, 2002

##### Distribution.

NE Vietnam: Hoa Binh. Outside Vietnam known from China (Fujian; Yunnan).

### Genus Gyragathis gen. n.

urn:lsid:zoobank.org:act:5BC4761E-BF89-4FEA-BC9C-F5948BCE94A3

#### Type species.

Gyragathis quyi sp. n.

#### Etymology.

From “gyros” (Greek for “circle”) and the generic name “Agathis”, because of the circular carina near the antennal sockets. Gender: feminine.

#### Diagnosis.

**Figure 198.**
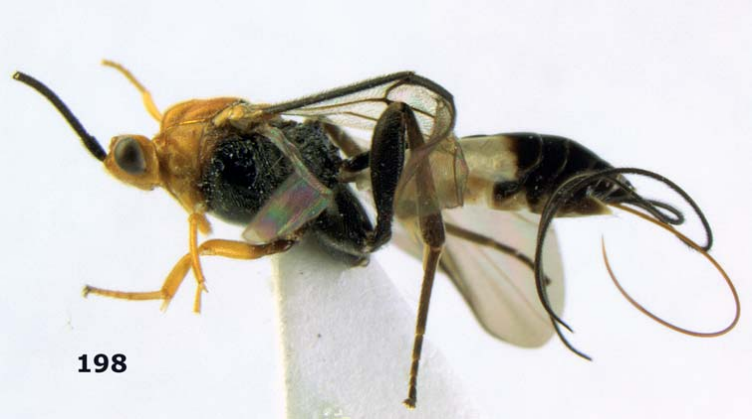
Gyragathis quyi gen. n. sp. n., female, holotype. Habitus lateral.

Apex of antenna without spine; antennal sockets (up to level of anterior ocellus) with a circular carina (Figs 206, 208); temples with a lateral tubercle (Fig. 207), in dorsal view parallel-sided and angulate (Fig. 206); area behind antennal sockets shallowly impressed (Fig. 206); area between antennal sockets with a trough (Fig. 208); pedicellus short (Fig. 198); clypeus flattened and with narrow straight ventral rim, but laterally wider and protruding (Fig. 205), malar flange protruding ventrally (Figs 205, 207); mouth-parts normal, galea not longer than wide, shorter than labial palp, and not visible in lateral view (Fig. 207); labrum slanted backwards, horizontal; mandible small; vertical axis of malar triangle about equal to horizontal axis, part of head below eyes directly narrowed ventrally (Fig. 205); malar suture absent; scutellum spaced punctate, with a subposterior crest and its medio-posterior depression transverse, rather narrow and largely smooth (Fig. 203); transverse metasternal carina non-lamelliform and below upper level of hind coxal cavities; veins of fore wing largely present, but vein 1-SR+M of fore wing absent (Fig. 202); vein 1-M of hind wing 0.6 times as long as vein M+CU; hind wing with 2 + 2 hamuli; fore and middle tarsal claws with a submedial lobe; hind trochantellus shorter than trochanter and trochanter flattened ventrally, without ventral carina; hind tibia without long black bristles (Fig. 198); outer face of middle tibia with row of pegs; ovipositor sheath distinctly longer than metasoma and 0.9–1.2 times fore wing.

#### Phylogenetic position.

Putative synapomorphies of the new genus Gyragathisare the circular carina of the frons, the tuberculate temple and the genal protuberance behind the mandible. It is otherwise similar to some atypical species of Therophilus Wesmael. Typical Therophilus has as synapomorphies the deeply concave frons, the narrowed marginal cell of the fore wing (occurs also in Camptothlipsis) and the widened second metasomal tergite.

#### Distribution.

Oriental (four species).

#### Biology.

Unknown.

#### Gyragathis quyi sp. n.

urn:lsid:zoobank.org:act:B3B86CDE-F9CF-4F03-A8A3-FCB294B7E791

Figs 198 –208 

##### Type material.

Holotype, ♀ (RMNH), “N. Vietnam: Ninh Binh, Cuc Phuong N.P., n[ea]r centre, c 225 m, 15–27.v.2000, Mai Phu Quy, RMNH’00”

##### Diagnosis.

The new species is similar to Gyragathis angulosa (Bhat & Gupta, 1977) comb. n., from the Philippines, but it has the notauli finely crenulate anteriorly and medially (smooth in Gyragathis angulosa), apical half of the wing membrane slightly infuscate (apical 0.7 dark brown and basally yellowish-hyaline), second tergite 1.1 times as long as its apical width and with a crenulate curved groove (about twice as long as wide and with a transverse groove), second metasomal suture smooth (crenulate), third tergite without a transverse groove and about 1.1 times as long as its basal width (groove present and about twice as long as wide). Gyragathis daanyuanensis (Chen & Yang, 2006) comb. n. from China (Fujian) has the head and mesoscutum black, the temple rugulose posteriorly, the ovipositor sheath about as long as the fore wing and the basal half of the hind tibia yellowish. Gyragathis parallelus (Chou & Sharkey, 1989) comb. n. from China (Taiwan) has the mesoscutum orange brown and the hind tibia completely dark brown, but the head is blackish, the first tergite is costate and the ovipositor sheath 0.9 times as long as the fore wing.

##### Description.

Holotype, ♀, length of body 8.4 mm, of fore wing 6.3 mm, of ovipositor sheath 7.7 mm.

###### Head.

Antennal segments 40; length of third segment 1.2 times fourth segment, length of third, fourth and penultimate segments 2.8, 3.0 and 2.0 times their width, respectively; length of apical segment twice as long as penultimate segment; maxillary palp 0.7 times height of head; malar space 2.7 times as long as basal width of mandible; in dorsal view length of eye 2.7 times temple; temples parallel-sided (Fig. 206); POL:OD:OOL = 6:4:11; face moderately densely punctate (Fig. 205), with distinct smooth interspaces; clypeus largely smooth, with some spaced fine punctures; frons flattened and smooth; vertex and temple shiny and mainly smooth.

###### Mesosoma.

**Figures 199–208.**
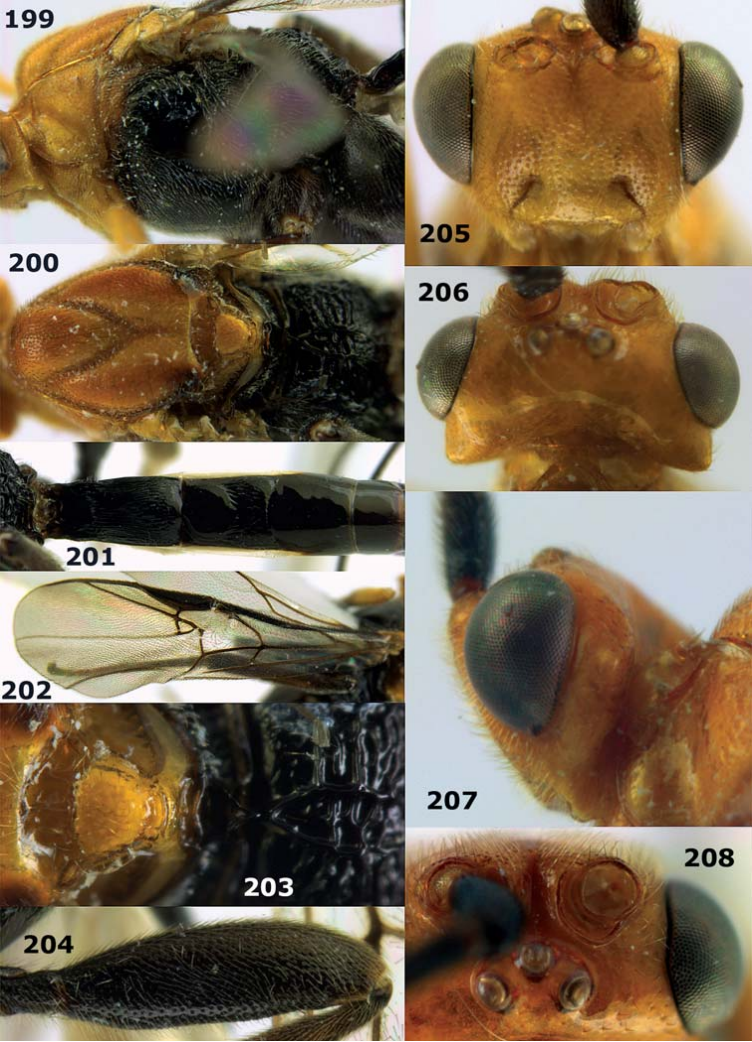
Gyragathis quyi gen. n. sp. n., male, holotype. **199** mesosoma lateral **200** mesosoma dorsal **201** first-third metasomal tergites dorsal **202** wings **203** scutellum and propodeum dorsal **204** hind femur lateral **205** head anterior **206** head dorsal **207** head lateral **208** detail of frons dorsal.

Length of mesosoma 1.5 times its height; subpronope large and shallow and epomial carina strongly developed; pronotum largely smooth with distinct punctures and strong setae dorsally; prepectal carina rather wide lamelliform; area near lateral carina of mesoscutum crenulate; middle lobe of mesoscutum densely finely punctate, but apically spaced punctate as lateral lobes; notauli deep and finely crenulate anteriorly, posteriorly smooth, united and forming a shallow groove (Fig. 200); scutellar sulcus 0.6 times as long as dorsal face of scutellum, with 3 carinae; scutellum somewhat convex, spaced punctate, its subposterior crest curved and distinct and its medio-posterior depression transverse, concave posteriorly, comparatively large and with a minute carina; precoxal sulcus narrowly crenulate and anteriorly absent (Fig. 199); mesopleuron below precoxal sulcus with moderately and spaced punctate; mesopleuron above precoxal sulcus shiny and smooth medially, coarsely punctate postero-ventrally; metapleuron rather densely coarsely punctate, but rugose ventrally; propodeum coarsely transversely rugose medially, anteriorly and posteriorly largely smooth except for narrow median area (Fig. 200); short median carina connected to elongate areola; propodeal spiracle medium-sized, 1.4 times as long as wide.

###### Wings.

Fore wing: second submarginal cell triangular, rather large and narrowly petiolate (Fig. 202); vein SR1 straight; r:3-SR+SR1 = 1:17; apical half of subbasal cell sparsely setose. Hind wing: vein M+CU 0.6 times as long as vein 1-M; no distinct vein 2-M; plical lobe large and glabrous, except for a few lateral setae.

###### Legs.

Length of hind femur, tibia and basitarsus 3.5, 6.5 and 10.5 times their width, respectively; hind femur (as remainder of legs) with short setae; length of outer and inner spur of middle tibia 0.45 and 0.55 times middle basitarsus, respectively; outer side of middle tibia with row of 5 pegs; length of outer and inner spur of hind tibia 0.4 and 0.6 times hind basitarsus, respectively; outer side of hind tibia with cluster of 10 pegs; outer side of hind coxa with sparse fine punctures; outer side of hind femur rugose-punctate.

###### Metasoma.

First tergite subparallel-sided, 1.9 times as long as its apical width (Fig. 201); dorsal carinae absent, finely and densely longitudinally striate, apically with sparse fine punctures and partly smooth; second tergite subparallel-sided, shiny, smooth but with a wide elongate punctate transverse depression, 1.1 times as long as its apical width and 1.3 times its basal width (Fig. 201); third tergite without a transverse groove and about 1.1 times as long as its basal width; ovipositor sheath 1.23 times as long as fore wing.

###### Colour.

Black or blackish-brown; head, palpi, pronotum, propleuron, mesoscutum (except dark brown patch latero-posteriorly), scutellum, tegula, mesopleuron antero-dorsally, fore and middle legs (but middle coxa, trochanter, trochantellus and base of femur brown) brownish-yellow to pale yellow (e.g., mesoscutum laterally and medio-posteriorly, respectively); apical half of metasoma dark brown, but hypopygium narrowly ivory apically and medially; middle and hind tibial spurs, first and second tergites narrowly dorsally, and ventral half of metasoma white; hind tibia (but basally blackish), hind tarsus, pterostigma and veins dark brown; wing membrane subhyaline, but apical half slightly infuscate.

##### Distribution.

N Vietnam: Ninh Binh.

##### Biology.

Unknown.

##### Etymology.

It is a pleasure to name this species after Prof. Dr Mai Phu Quy, who was an excellent counterpart during the RMNH-IEBR expeditions in Vietnam in the period 1999–2007.

### Genus Gyrochus

Enderlein, 1920

#### Gyrochus yunnanensis

Wang, 1984

Figs 209–213 

##### Distribution.

S Vietnam: Dak Lak. New record for Vietnam. Known outside Vietnam from China (Yunnan and Hainan Island).

**Figures 209–213.**
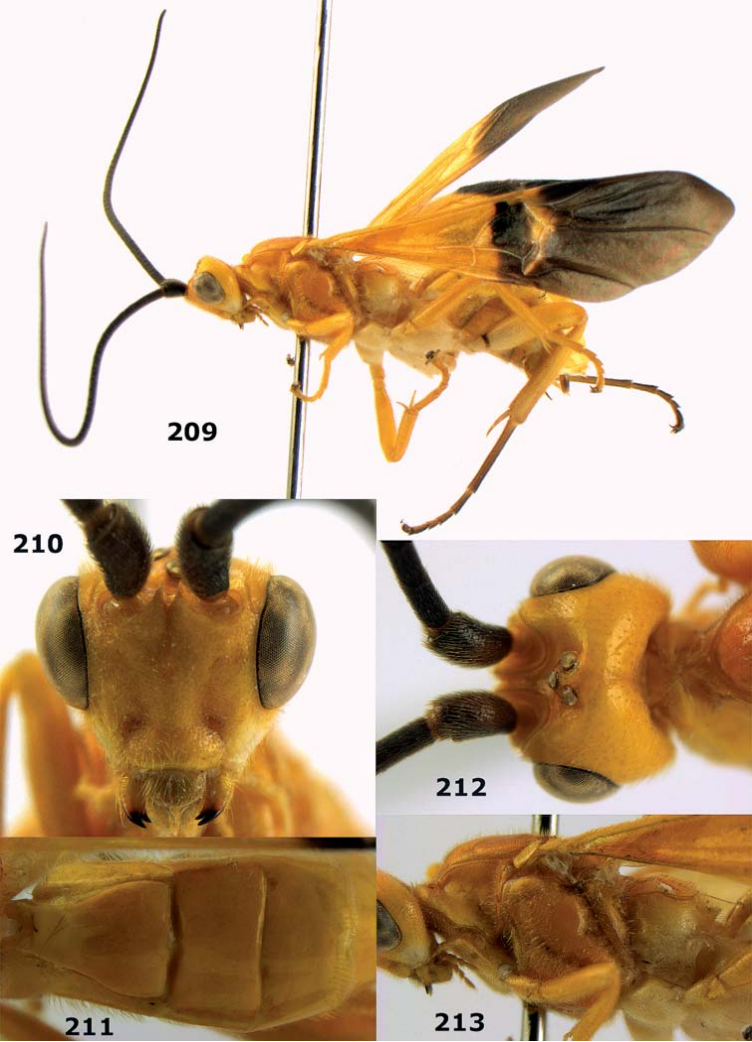
Gyrochus yunnanensis Wang, female, Chu Yang Sin National Park. **209** habitus lateral **210** head anterior **211** first-third metasomal tergites dorsal **212** head dorsal **213** mesosoma lateral.

##### Note.

The holotype has only the apical fifth of the fore wing dark brown and the pterostigma brown.

### Genus Lytopylus

Foerster, 1862

#### Note.

The only Vietnamese species has the hind leg (but the tibial spurs are white or ivory) and the head (but the labrum is white or pale brown) black, the basal half of third metasomal tergite coarsely striate and with a transverse crenulate groove (Fig. 216) and the first tergite distinctly widened apically (Figs 216, 219).

#### Lytopylus romani

(Shestakov, 1940) comb. n.

Figs 214–219 

##### Distribution.

**Figures 214–219.**
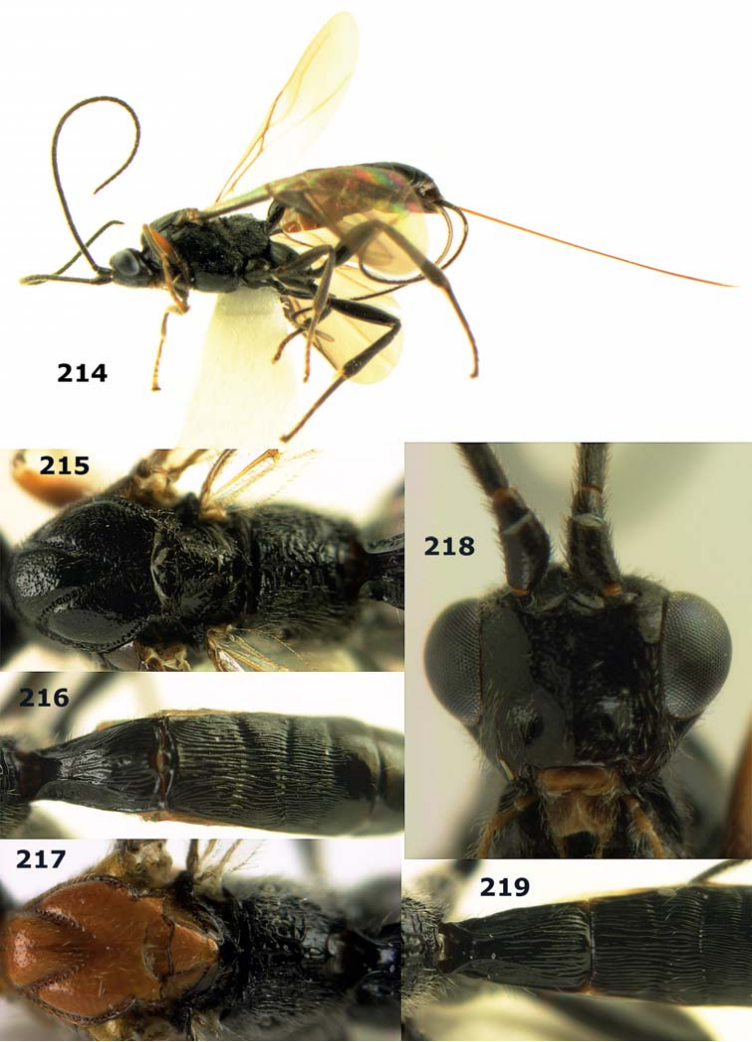
Lytopylus romani (Shestakov), female, Hoang Lien National Park, but 217 and 219 of pale form from Pa Co Hang Kia National Park. **214** habitus lateral **215**, **217** mesosoma dorsal **216**, **219** first-third metasomal tergites dorsal **218** head anterior.

NW Vietnam: Lao Cai, Hoa Binh. Wide spread outside Vietnam (from India to Japan) and a parasitoid of Tortricidae. The specimen from Hoa Binh belongs to Lytopylus romani f. ebulus (Nixon, 1950), because it has the pronotum, mesoscutum, scutellum and mesopleuron reddish or orange-brown (Fig. 217), which are black in the typical form (Figs 214, 215).

### Genus Therophilus

Wesmael, 1837

#### Note.

The majority of the Vietnamese Therophilus species previously were considered to belong to the genera Agathis Latreille and later to Bassus Fabricius. The genus Therophilus Wesmael is rather heterogeneous (e.g., Figs 224, 232, 250, 270, 282) and probably will be divided into several genera or subgenera in the future.

#### Key to Vietnamese species of the genus Therophilus Wesmael

**Table:** 

1.	Scutellum densely rugose, rugulose or rugulose-punctate, largely without distinct smooth interspaces; marginal cell of fore wing comparatively wide (Figs 297, 305); area below precoxal sulcus very densely punctate; basal area of second tergite moderately wide (Figs 298, 299, 304)	2
–	Scutellum punctate and with distinct interspaces or largely smooth; marginal cell of fore wing usually narrower (Figs 223, 233, 242) and area below precoxal sulcus moderately punctate; if densely punctate and marginal cell rather wide then basal area of second tergite strongly transverse (Fig. 282: Therophilus javanus)	3
2.	Lateral lobes of mesoscutum rugose-striate posteriorly; notauli fused posteriorly, forming deep groove near scutellar sulcus (Fig. 303); second metasomal tergite smooth or superficially finely striate (Fig. 304)	Therophilus marucae sp. n.
–	Lateral lobes of mesoscutum finely punctate posteriorly; notauli connected posteriorly, forming a curved crenulate groove, area near scutellar sulcus flat; second tergite smooth or anterior half more or less superficially obliquely striate (Figs 298, 299)	Therophilus marshi (Bhat & Gupta, 1977) comb. n.
3.	Frons deeply depressed and in front of anterior ocellus with a distinctly protruding lamella (Figs 264, 266); precoxal sulcus coarsely crenulate and area below it coarsely punctate and anteriorly reticulate; hind tibia black, except for a more or less developed white (N Vietnam) or pale brown (S Vietnam) basal band (Fig. 256); hind femur densely and coarsely rugose-striate; wing membrane dark brown (N Vietnam; Fig. 260)) to yellowish-brown (S Vietnam); [head black]	Therophilus depressiferus sp. n.
–	Frons at most moderately depressed and in front of anterior ocellus without a distinctly protruding lamella (Figs 227, 236, 254); precoxal sulcus finely crenulate and area below it and anteriorly finely punctate; hind tibia usuall y whitish except black apical third (Figs 229, 238, 277, 283, 285); hind femur at most densely punctate; wing membrane subhyaline or slightly infuscate	4
4.	Body completely pale yellowish or brownish-yellow, with base of first tergite and second tergite more or less ivory (Fig. 310); frons rather depressed (Fig. 307); length of ovipositor sheath 0.6–0.7 times fore wing; [malar space about 3.3 times as long as basal width of mandible; first metasomal tergite 1.3 times as long as its apical width; hind leg yellow]	Therophilus mellisoma sp. n.
–	Body at least partly black or dark brown (Figs 220, 229, 238); frons hardly or not depressed (Figs 227, 235, 354); length of ovipositor sheath variable	5
5.	First metasomal tergite 2.5–2.7 times as long as its apical width (Figs 270, 359); mesosoma (Figs 267, 268, 356) and second metasomal tergite elongate (Figs 270, 359); base of second tergite ivory	6
–	First tergite 1.0–1.7 times as long as its apical width (Figs 224, 232, 241); mesosoma (Fig. 229, 238, 276) and usually second tergite (Figs 250, 259, 278) comparatively short; colour of base of second tergite variable	7
6.	Head and mesosoma brownish-yellow (Fig. 267); base of first tergite narrowly ivory (Fig. 270); mesopleuron comparatively robust (Fig. 268); mesoscutum smooth, at most somewhat punctulate	Therophilus elongator sp. n.
–	Head, mesosoma and base of first tergite black (Figs 356, 359); mesopleuron comparatively slender (Fig. 357); mesoscutum finely punctate	Therophilus punctiscutum sp. n.
7.	Second metasomal tergite distinctly striate (Figs 368, 377, 386; rarely partly smooth anteriorly); hind femur weakly sculptured	8 Note. If head orange brown, vertex posteriorly very finely transversely aciculate, second submarginal cell of fore wing minute but with vein r-m of fore wing distinctly developed, frons with medial ridge, second tergite with smooth convex area surrounded by striae and notauli completely and distinctly crenulate, cf. Therophilus choui (Chen & Yang, 2006) comb. n. from China.
–	Second tergite smooth or largely so (Figs 232, 282, 350); sculpture of hind femur variable	14
8.	Mesoscutum protruding over pronotum (Fig. 366); middle mesoscutal lobe comparatively short and wide (Fig. 367); second metasomal tergite elongate and subparallel-sided, ivory (Fig. 368)	Therophilus robustus sp. n.
–	Mesoscutum not protruding anteriorly (Figs 339, 374); middle mesoscutal lobe more elongate and narrower (Figs 221, 376); second tergite transverse (Fig. 377) or narrowed anteriorly (Figs 323, 341, 386) and black (but ivory in Therophilus festivus; Fig. 278)	9
9.	Hind femur robust (Fig. 379); second metasomal tergite basally smooth, with a distinctly curved groove, and posteriorly striate (Fig. 377); [middle lobe of mesoscutum flat medially]	Therophilus rugosiferus sp. n.
–	Hind femur moderately slender (Figs 225, 327, 343); second tergite basally striate, as is the remainder of tergite, and often with a less developed groove (Figs 224, 323, 341)	10
10.	Mesoscutum rather matt and densely micro-sculptured (Fig. 340); hind femur comparatively slender (Fig. 343); first metasomal tergite robust and comparatively wide (Fig. 341); second submarginal cell of fore wing normal (Fig. 342); [second tergite coarsely striate, comparatively wide and with a curved groove]	Therophilus parasper sp. n.
–	Mesoscutum distinctly shiny and evenly finely punctate (Figs 221, 322); hind femur less slender (Figs 225, 327); first tergite less robust and narrower (Figs 224, 278, 323); [mesoscutum black and moderately slender; propodeum finely reticulate and often partly only coriaceous]	11 Note. If the mesoscutum is orange brown and rather robust (Fig. 238), the second tergite more slender (Fig. 241) and the propodeum coarsely reticulate (Fig. 240), cf. sculptured specimens of Therophilus contrastus sp. n.
11.	Second metasomal tergite finely striate, ivory anteriorly and tergite hardly narrowed anteriorly (Figs 224, 278); length of ovipositor sheath 0.9–1.3 times fore wing; second submarginal cell of fore wing medium-sized (Figs 223, 276); subbasally hind tibia white	12
–	Second tergite rather coarsely to finely costate, black and tergite narrowed anteriorly (Figs 323, 386); length of ovipositor sheath 0.6–0.7 times fore wing; second submarginal cell of fore wing smaller (Figs 320, 387); subbasally hind tibia black to brown	13
12.	Basal half of hind tibia entirely white (Figs 276, 277, 280); first metasomal tergite somewhat widened apically (Fig. 278)	Therophilus festivus (Muesebeck, 1953) comb. n.
–	Basal half of hind tibia largely dark brown, only with a subbasal white ring (Figs 220, 225); first tergite parallel-sided apically (Fig. 224)	Therophilus annuliferus sp. n.
13.	First metasomal tergite regularly and rather densely striate laterally (Fig. 386); scutellum angulate anteriorly and coarsely punctate (Fig. 385); subbasally hind tibia black; N Vietnam	Therophilus scutellatus sp. n.
–	First tergite sparsely, and rather irregularly striate laterally (Fig. 323); scutellum rounded anteriorly and sparsely punctate (Fig. 322); subbasally hind tibia dark brown; S Vietnam	Therophilus nigrolineatus sp. n. Note. Similar to Therophilus transcasperatus (Chen & Yang, 2006) comb. n. from China, but that species has the hind leg largely yellowish-brown, the hind tibia ivory (except for its apical darkened quarter), the second tergite with a distinct curved transverse impression, vein r-m of the fore wing very small, the hind femur rather slender, the antenna brown and the face largely, the pronotum ventrally and the posterior orbita pale brown or yellowish-brown. If the basal 0.4 of the hind tibia is pale yellowish (with only a faintly indicated darker subbasal band), cf. the male holotype of Therophilus fujianicus (Chen & Yang, 2006) comb. n. from China. [fore and middle legs and hind trochanter and trochantellus yellowish-brown; prepectal carina rather weakly developed; vein r-m of fore wing in only one wing reduced; surroundings of vein M+CU1 of fore wing sparsely setose; middle mesoscutal lobe rather convex, somewhat more convex than lateral lobes]. If similar but notauli distinctly crenulate, ovipositor sheath about as long as fore wing (not about twice as indicated in the original description) and propodeum coarsely vermiculate-rugose, cf. Therophilus tanycoleosus (Chen & Yang, 2006) comb. n. from China.
14.	Hind femur very coarsely and densely punctate ventrally (Fig. 281); area below precoxal sulcus very densely punctate; second metasomal tergite shorter than third tergite and with strongly transverse anterior part (Fig. 282); second submarginal cell of fore wing very small (Fig. 281); mesonotum orange-yellow	Therophilus javanus (Bhat & Gupta, 1977) comb. n.
–	Hind femur at most moderate punctate ventrally (Fig. 234); area below precoxal sulcus spaced punctate; second tergite at least somewhat longer than third tergite and with less transverse anterior part (Figs 232, 241, 288); shape of second submarginal cell of fore wing and colour of mesonotum variable	15
15.	Hind femur robust, rather swollen (Figs 234, 252, 290, 352); subbasal cell of fore wing evenly setose; length of ovipositor sheath 0.5–1.0 times fore wing; second metasomal tergite comparatively short and transverse (Figs 232, 250, 288, 350), entirely black or brownish-yellow	16
–	Hind femur moderately slender (Figs 243, 294, 334); subbasal cell of fore wing at least partly glabrous; length of ovipositor sheath 0.5–0.9 times fore wing; second tergite comparatively elongate (Figs 214, 296, 332), ivory anteriorly and dark brown or black posteriorly	19
16.	Length of ovipositor sheath about 0.5 times fore wing (Fig. 347); posterior half of frons flat, no triangular area in front of anterior ocellus (Fig. 354); tegulum yellowish-brown, contrasting with dark brown humeral plate (Fig. 349); hind trochanter yellowish-brown; [marginal cell of fore wing very narrow (Fig. 351); ocelli small]	Therophilus planifrons sp. n.
–	Length of ovipositor sheath 0.8–1.0 times fore wing (Figs 229, 247, 285); posterior half of frons partly depressed and with a triangular area in front of anterior ocellus (Figs 236, 254, 293); tegulum and humeral plate similarly coloured (Figs 231, 249); hind trochanter dark brown	17
17.	Head and mesosoma (except propodeum) brownish-yellow (Fig. 229); vein M+CU of hind wing 1.2–1.3 times as long as vein 1-M; second metasomal tergite rather square (Fig. 232); marginal cell of fore wing narrow parallel-sided (Fig. 233); anterior ocellus smaller than posterior ocelli (Fig. 236); scutellum without distinct subposterior transverse crenulate depression (Fig. 231)	Therophilus cattienensis sp. n. Note. If only apical 0.4 of metasoma is dark brown, vein M+CU of hind wing about as long as vein 1-M, hind femur very wide (at most 3 times as long as wide), notauli finely crenulate, second submarginal cell small, triangular and petiolate, marginal cell of fore wing very narrow, prepectal carina medium-sized, propodeum coarsely areolate and mesoscutum yellowish-brown (but laterally darkened), cf. Therophilus tongmuensis (Chen & Yang, 2006) comb. n. from China.
–	Head and mesosoma black (Figs 247, 285); vein M+CU of hind wing about as long as vein 1-M (Fig. 289); second tergite distinctly transverse (Figs 250, 288); anterior ocellus similar to posterior ocelli (Figs 254, 293); scutellum with a subposterior transverse crenulate depression (Figs 249, 287)	18
18.	Frons laterally rather coarsely (female) or finely (male) punctate; basal half of hind tibia largely whitish, with only a subbasal dark brown patch (Figs 285, 290); medial area of second tergite protruding medio-posteriorly (Fig. 288); propodeum coarsely reticulate-rugose and transverse carina distinctly developed (Fig. 287); [length of first tergite about 1.3 times as long as wide apically; propodeum posteriorly steep]	Therophilus levisoma sp. n.
–	Frons laterally sparsely punctulate; basal half of hind tibia partly dark brown (Figs 247, 252); medial area of second tergite hardly or not protruding medio-posteriorly (Fig. 250); propodeum rather finely reticulate and transverse carina weakly developed (Fig. 249)	Therophilus crenulisulcatus sp. n.
19.	Length of ovipositor sheath 0.5–0.6 times fore wing (Fig. 294); second metasomal tergite widened apically and shorter, without a transverse groove and completely smooth (Fig. 296); mesonotum black; apical half of subbasal cell of fore wing largely sparsely setose	Therophilus lienhoachihensis (Chou & Sharkey, 1989) comb. n.
–	Length of ovipositor sheath 0.9 times fore wing (Figs 238, 328); second tergite hardly widened apically and longer, with a shallow transverse groove and with more or less superficial striae (Figs 241, 332); colour of mesonotum variable; setosity of apical half of subbasal cell of fore wing variable	20
20.	Mesoscutum and scutellum orange brown (Fig. 238); notauli coarsely crenulate posteriorly (Fig. 240); scutellum distinctly rugulose medio-posteriorly (Fig. 240); first metasomal tergite without superficial granulation; apical half of subbasal cell of fore wing glabrous or nearly so	Therophilus contrastus sp. n.
–	Mesoscutum and scutellum black (Fig. 329); notauli narrowly crenulate posteriorly (Fig. 331); scutellum largely smooth medio-posteriorly (Fig. 331); first metasomal tergite with superficial granulation; apical half of subbasal cell of fore wing largely setose	Therophilus nuichuaensis sp. n.

#### Therophilus annuliferus sp. n.

urn:lsid:zoobank.org:act:15FAC037-95B4-41EB-862D-20A16AD939DF

Figs 220 –228 

##### Type material.

Holotype, ♀ (RMNH), “C. Vietnam: Thua Thien Hué, Phong Dién N.R., nr base-camp, 15 km W. Phong My, 50–60 m, 23.iii-6.iv.2001, Mal. traps 4 + 5, C. v. Achterberg & R. de Vries, RMNH”. Paratypes: 2 ♀ (RMNH, IEBR), “S. Vietnam: Dak Lak, Chu Yang Sin, Krong K’Mar, Mal. traps, 800–940 m, 2–10.vi.2007, C. van Achterberg & R. de Vries, RMNH’07”.

##### Diagnosis.

Closely related to Therophilus nigrolineatus sp. n., but Therophilus annuliferus has the ovipositor sheath about as long as the fore wing (Therophilus nigrolineatus: 0.6 times) and the sculpture of the second metasomal tergite only striate (nigrolineatus: finely costate).

##### Description.

Holotype, ♀, length of body 5.9 mm, of fore wing 4.6 mm, ovipositor sheath 4.9 mm.

###### Head.

Antenna incomplete, with 31 segments remaining, length of third segment 1.5 times fourth segment, length of third and fourth segments 4.0 and 2.8 times their width, respectively; length of maxillary palp 0.8 times height of head; malar space 1.1 times as long as basal width of mandible; in dorsal view length of eye 4.0 times temple; temple directly narrowed posteriorly (Fig. 227); ocelli in low triangle, POL:OD:OOL= 9:5:11; face shiny and distinctly rather finely punctate; clypeus largely smooth and weakly convex, only laterally punctulate; frons with weak medial ridge, smooth medially and densely finely punctate laterally; vertex and temple shiny and largely smooth, with sparse fine punctures.

###### Mesosoma.

Length of mesosoma 1.5 times its height; pronotum smooth with five carinae anteriorly, finely densely punctate dorso-posteriorly and posterior groove distinctly crenulate; area near lateral carina of mesoscutum crenulate; mesoscutum spaced finely punctate, but densely anteriorly, medio-posteriorly lobes slightly convex; notauli complete and narrowly crenulate; scutellar sulcus 0.6 times as long as dorsal face of scutellum, shallow and with 3 carinae; scutellum shiny and with sparse rather coarse punctures, subposterior crest obsolescent (Fig. 221); precoxal sulcus deep, strongly but narrowly crenulate and anteriorly absent (Fig. 222); mesopleuron below precoxal sulcus spaced finely punctate; remainder of mesopleuron shiny and largely smooth, but rather coarsely punctate below scrobe; metapleuron densely setose, spaced moderately punctate and ventrally rugose; propodeum coarsely reticulate, but smooth posteriorly (Fig. 221); propodeal spiracle rather large, 1.3 times as long as wide

###### Wings.

Fore wing: second submarginal cell small and petiolate (Fig. 223); vein SR1 straight; vein r short, r:3-SR+SR1 = 1:26; r-m about as long as petiolus (Fig. 223); apical half of subbasal cell sparsely setose. Hind wing: vein M+CU 0.8 times as long as vein 1-M.

###### Legs.

Length of hind femur, tibia and basitarsus 4.2, 7.7 and 12 times their width, respectively; hind femur densely and rather coarsely punctate but with narrow smooth interspaces and with short setae (Fig. 225); length of outer and inner spur of middle tibia 0.5 and 0.7 times middle basitarsus, respectively; outer side of middle tibia with row of 4 pegs and 2 pegs at apex; length of outer and inner spurs of hind tibia 0.3 and 0.5 times hind basitarsus, respectively; tarsal claws with large lobe.

###### Metasoma.

First tergite parallel-sided without distinct dorsal carinae, its length 2.3 times its apical width (Fig. 224); first and second tergites densely and moderately coarsely striate; second tergite somewhat narrowed anteriorly and with a curved transverse groove (Fig. 224); remainder of metasoma (including second suture) smooth; ovipositor sheath 1.06 times as long as fore wing.

###### Colour.

**Figure 220.**
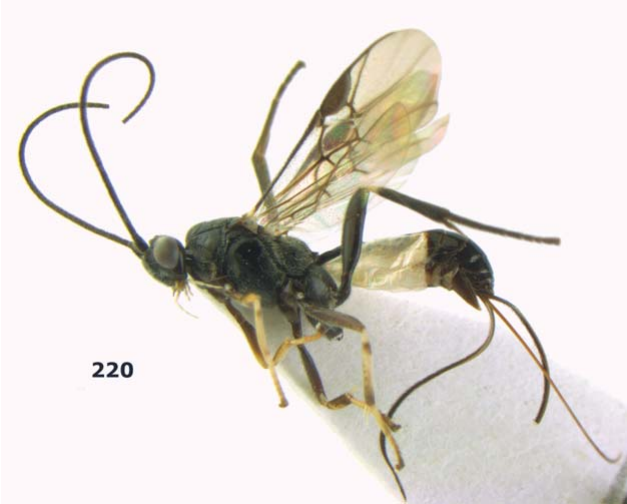
Therophilus annuliferus sp. n., female, holotype. Habitus lateral.

Black; antenna blackish-brown; mesoscutum slightly chestnut-brown posteriorly; palpi, mandible, second tergite laterally, third tergite antero-laterally and basal half of metasoma ventrally white or ivory; fore leg (but coxa, trochanter, trochantellus, femur ventrally and partly laterally and small patch of tibia brown), middle tibia dorsally (but subbasal patch and apical fifth brown), spurs and tarsus pale yellow; remainder of middle leg dark brown; hind leg black or blackish-brown and with a white basal tibial ring (Fig. 220); tegulae, apical half of metasoma ventrally, veins and pterostigma dark brown; wing membrane slightly infuscate.

###### Variation.

Antennal segments of female 35; vein M+CU of hind wing 0.8–0.9 times as long as vein 1-M; outer side of middle tibia with row of 4–5 pegs; length of body 5.2–5.9 mm, of fore wing 4.4–4.6 mm; length of first tergite 2.2–2.3 times as long as wide apically; length of ovipositor sheath 0.9–1.1 times fore wing.

##### Distribution.

S Vietnam: Dak Lak and C Vietnam: Thua Thien Hué.

**Figures 221–228.**
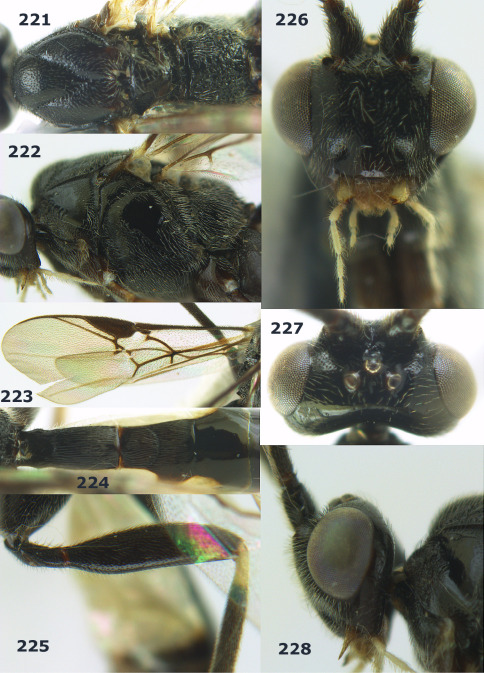
Therophilus annuliferus sp. n., female, holotype. **221** mesosoma dorsal **222** mesosoma lateral **223** wings **224** first-third metasomal tergites dorsal **225** hind femur lateral **226** head anterior **227** head dorsal **228** head lateral.

##### Biology.

Unknown.

##### Etymology.

From “annulus” (Latin for “ring”), and “fero” (Latin for “carry”), because of the white basal ring of the hind tibia.

#### Therophilus cattienensis sp. n.

urn:lsid:zoobank.org:act:C1A6A43F-2997-4075-B2D0-7C078F96D81C

Figs 229 –237 

##### Type material.

Holotype, ♀ (RMNH), “S. Vietnam: Dong Nai, Cat Tien N.P., Mal. traps, c. 100 m, 9.iv-13.v.2007, M.P. Quy, N.T. Manh & C. v. Achterberg, RMNH’07”. Paratype: 2 ♂ (IEBR, RMNH), “S. Vietnam: Dak Lak, Chu Yang Sin N.P., n[ea]r dam, 840–940 m, 2–10.vi.2007, Mal. traps, C. v. Achterberg & R. de Vries, RMNH’07”.

##### Diagnosis.

The new species is close to Therophilus lanyuensis (Chou & Sharkey, 1989) comb. n. from China, but differs by having the precoxal sulcus weakly crenulate and narrow (Therophilus lanyuensis: distinctly crenulate and wide), the metapleuron rugose-punctate (lanyuensis: reticulate ventrally), the mesopleuron below the precoxal sulcus smooth (lanyuensis: densely punctate) and about 28 antennal segments (lanyuensis: 38–40).

##### Description.

Holotype, ♀, length of body 3.3 mm, of fore wing 2.6 mm, of ovipositor sheath 2.4 mm.

###### Head.

Antennal segments 28, length of third segment 1.2 times fourth segment, length of third, fourth and penultimate segments 4.0, 3.3 and 2.3 times their width, respectively; length of apical antennal segment 1.4 times as long as penultimate segment; maxillary palp 0.8 times height of head; malar space 2.5 times as long as basal width of mandible; in dorsal view length of eye 5.7 times temple; ocelli in high triangle (Fig. 236), POL:OD:OOL= 6:4:9; with two carinae between antennal sockets; face with distinct punctures; frons with distinct punctures laterally; vertex and temple shiny with sparse fine punctures.

###### Mesosoma.

Length of mesosoma 1.5 times its height; pronotal trough rugose, dorsally rugose-punctate; area near lateral carina of mesoscutum crenulate; mesoscutum shiny, sparsely punctate and setose; notauli complete, wide and largely crenulate; scutellar sulcus 0.5 times as long as scutellum with 3 carinae; scutellum without subposterior crest (Fig. 231), sparsely but distinctly punctate; precoxal sulcus weakly crenulate and narrow; mesopleuron below precoxal sulcus smooth; mesopleuron above precoxal sulcus with sparse distinct punctures; metapleuron rugose-punctate; propodeum reticulate-rugose.

###### Wings.

Fore wing: second submarginal medium-sized and triangular (Fig. 233); marginal cell narrow (Fig. 233); vein SR1 straight; r:3-SR+SR1=2:37. Hind wing: vein M+CU 1.3 times as long as vein 1-M (22:17).

###### Legs.

Length of hind femur, tibia and basitarsus 2.6, 5.0 and 7.5 times their width, respectively; hind femur (as remainder of legs) with short setae; length of outer and inner spur of middle tibia 0.4 and 0.6 times middle basitarsus, respectively; outer side of middle tibia with 4 pegs; length of outer and inner spur of hind tibia 0.3 and 0.5 times hind basitarsus, respectively (figs); tarsal claws with lobe.

###### Metasoma.

Length of first tergite 1.3 times its apical width (Fig. 232); first tergite coarsely longitudinally striate (rather costate); second tergite 0.9 times as long as third tergite, smooth but partly superficially granulate and with smooth transverse groove (Fig. 232); remainder of metasoma smooth; ovipositor sheath 0.9 times as long as fore wing.

###### Colour.

Brownish-yellow; propodeum, apex of hind tibia and hind tarsus, first tergite basally and third-seventh tergites largely dark brown; pterostigma dark brown; wing membrane subhyaline.

###### Variation.

Antennal segments of female 28, of male 30; outer side of middle tibia with row of 1–4 pegs; length of hind femur 2.6–3.0 times as long as wide; second tergite pale yellow (♀) or largely dark brown (♂), smooth or partly superficially granulate; length of body 3.2–3.3 mm, of fore wing 4.4–4.6 mm; head dorsally, mesopleuron partly and metapleuron of males dark brown or blackish.

##### Distribution.

S Vietnam: Dak Lak, Dong Nai.

**Figure 229.**
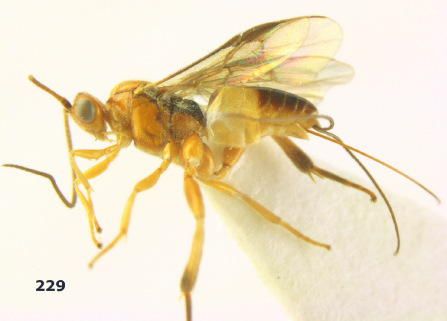
Therophilus cattienensis sp. n., female, holotype. Habitus lateral.

**Figures 230–237.**
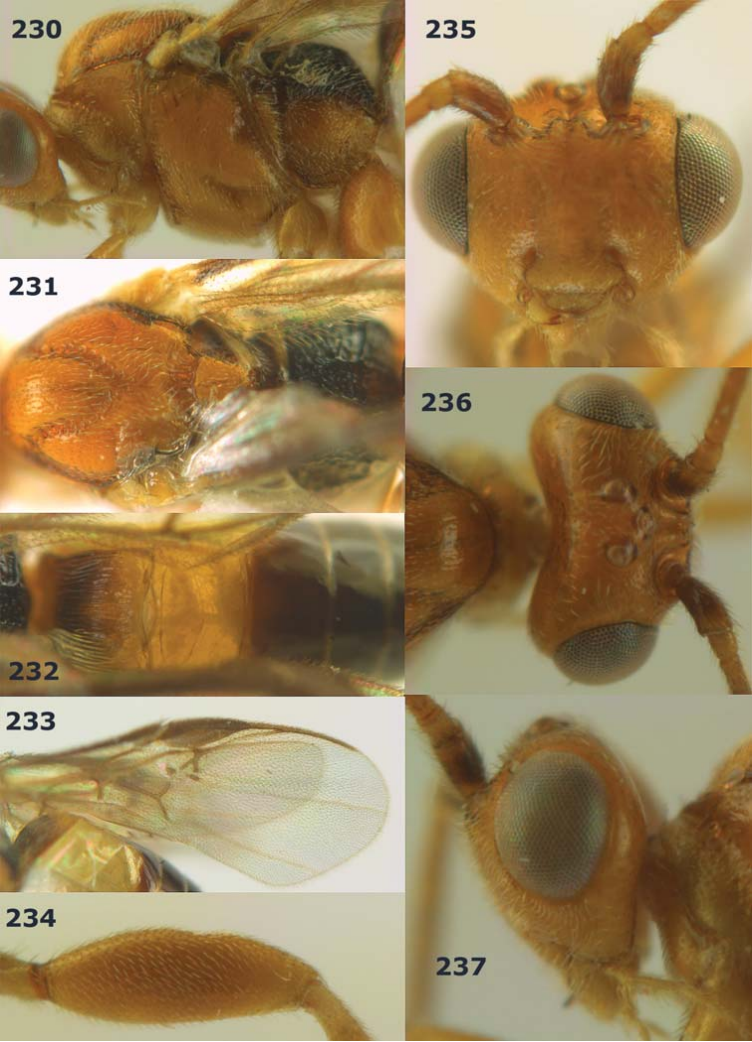
Therophilus cattienensis sp. n., female, holotype. **230** mesosoma lateral **231** mesosoma dorsal **232** first-third metasomal tergites dorsal **233** wings **234** hind femur lateral **235** head anterior **236** head dorsal **237** head lateral.

##### Biology.

Unknown.

##### Etymology.

Named after the type locality: Cat Tien National Park.

#### Therophilus contrastus sp. n.

urn:lsid:zoobank.org:act:D8721AA6-7169-45A9-B605-97DDFB9B8E58

Figs 238 –246 

##### Type material.

Holotype, ♀ (RMNH), “S. Vietnam: Dong Nai, Cat Tien N.P., c 100 m, 13–19.v.2007, Crocodile tr[ail], Mal. traps, C. v. Achterberg & R. de Vries, RMNH’07”. Paratypes (3 ♀): 1 ♀ (IEBR), id. but Ficus trail, 9–30.iv.2007; 1 ♀ (RMNH), “Museum Leiden, Vietnam (Dong Nai Prov.), Cat Tien N.P., Ben Cu trail, 14–20.v.2007, C. van Achterberg, R. de Vries & E. Gassó Miracle”, “mixed bamboo and wood [= deciduous] forest, by hand, 220 m, 11°26'54.8N; 107°26'30.9E; 1 ♀ (IEBR), Aga. 092, “N. Vietnam: Ha Noi, Gia Lam, orchard, MT, 20–30.iv.2001, K.D. Long”.

##### Diagnosis.

The new species is similar to Therophilus lienhoachihensis (Chou & Sharkey, 1989), but differs by having the notauli fused posteriorly, forming a large crenulate area (Therophilus lienhoachihensis:rugose); the scutellum with sparse fine punctures ( lienhoachihensis:rugose-punctate) and the mesonotum reddish yellow (lienhoachihensis:black). Bassus albifasciatus (Watanabe, 1934) is similar, but Therophilus contrastus differs by having the tarsal claws with a large lobe (Bassus albifasciatus: claws without a lobe); outer side of the middle tibia with a row of 4–5 pegs (albifasciatus: 4–10 pegs); the pronotum crenulate anteriorly (albifasciatus: with two carinae) and the head entirely black (albifasciatus: reddish-brown). The new species is also close to Therophilus festivus (Muesebeck, 1953), but differs by having the first tergite about 1.7 times as long as its apical width (Therophilus festivus: 1.8–1.9 times); pronotum and mesonotum reddish-yellow (festivus: black)and basal half of hind tibia without a dark brown basal ring (festivus: basal ring usually present).

##### Description.

Holotype, ♀, length of body 5.2 mm, of fore wing 3.6 mm, ovipositor sheath 3.0 mm.

###### Head.

Antennal segments 36, length of third segment 1.2 times fourth segment, length of third, fourth and penultimate segments 3.7, 3.1 and 2.0 times their width, respectively; length of apical antennal segment 1.3 times as long as penultimate segment; maxillary palp 2.2 times height of head; malar space 2.7 times as long as basal width of mandible; in dorsal view length of eye 3.5 times temple; temple directly narrowed posteriorly (Fig. 246); POL:OD:OOL= 9:6:11; face with densely punctate; frons rather dull and distinctly punctate laterally (Fig. 246); vertex and temple with sparse fine punctures.

###### Mesosoma.

Length of mesosoma 1.5 times its height; subpronope shallow; pronotal trough largely smooth, crenulate anteriorly, dorsally with sparse fine punctures; area near lateral carina of mesoscutum crenulate; mesoscutum with very sparse fine punctures; notauli complete and crenulate; scutellar sulcus 0.6 times as long as dorsal face of scutellum and with 3 carinae; scutellum with sparse punctures, subposterior crest short (Fig. 240); precoxal sulcus complete and largely crenulate (Fig. 239); mesopleuron with sparse distinct punctures; mesopleuron rugose-punctate anteriorly, reticulate-rugose posteriorly; propodeum largely reticulate-rugose; propodeal spiracle small, as long as wide.

###### Wings.

Fore wing: second submarginal cell small (Fig. 242); vein SR1 straight; r:3-SR+SR1 = 3:62. Hind wing: vein M+CU 0.8 times as long as vein 1-M.

###### Legs.

Length of hind femur, tibia and basitarsus 3.8, 6.1 and 9.2 times their width, respectively; hind femur (as remainder of legs) with short setae (Fig. 243); length of outer and inner spur of middle tibia 0.4 and 0.7 times middle basitarsus, respectively; outer side of middle tibia with a row of 3 pegs; length of outer and inner spur of hind tibia 0.3 and 0.5 times hind basitarsus, respectively; outer apex of hind tibia with a cluster of 7 pegs; tarsal claws with a distinct lobe.

###### Metasoma.

First tergite longitudinally striate without dorsal carinae; length of first tergite 1.7 times its apical width (Fig. 241); second tergite with weak transverse groove, slightly striate (Fig. 241); remainder of metasoma shiny and smooth; ovipositor sheath 0.8 times as long as fore wing.

###### Colour.

Black; mouthparts, fore and middle legs, hind trochantellus and basal half of hind tibia pale yellow; pronotum, mesonotum, mesopleuron above precoxal sulcus orange brown; second tergite basally, first-second tergites laterally ivory; pterostigma dark brown; wing membrane subhyaline.

###### Variation.

Antenna with 33–36 segments; first tergite 1.7–1.9 times as long as its apical width; second tergite weakly longitudinally striate or smooth; ovipositor sheath 0.8–0.9 times as long as fore wing; length of body 4.9–5.2 mm and of fore wing 3.6–3.8 mm.

##### Distribution.

NE Vietnam: Ha Noi and S Vietnam: Dong Nai.

**Figure 238.**
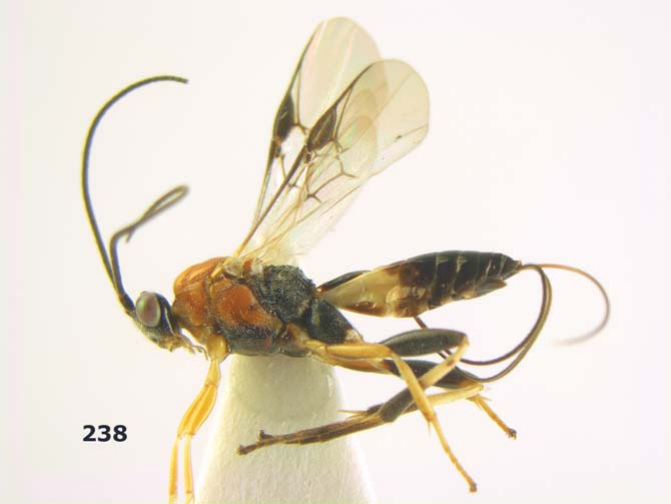
Therophilus contrastus sp. n., female, holotype. Habitus lateral.

**Figures 239–246.**
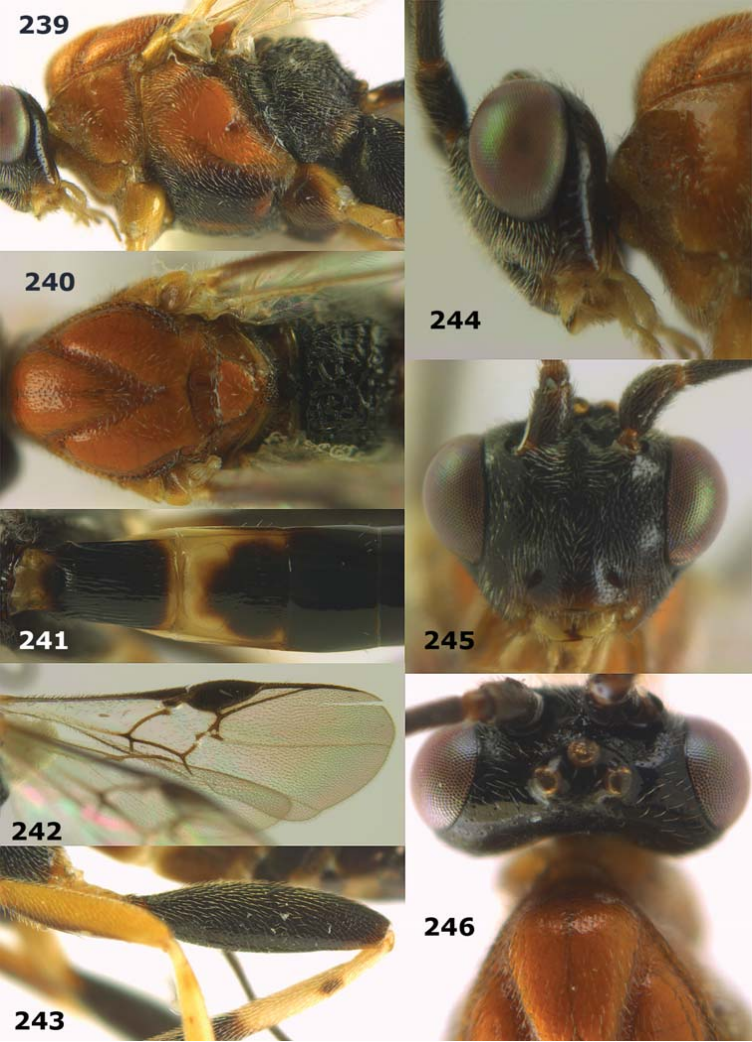
Therophilus contrastus sp. n., female, holotype. **239** mesosoma lateral **240** mesosoma dorsal **241** first-third metasomal tergites dorsal **242** wings **243** hind femur lateral **244** head lateral **245** head anterior **246** head dorsal.

##### Biology.

Unknown.

##### Etymology.

From “contra” (Latin for “opposite”), because of the contrasting orange and black body colour.

#### Therophilus crenulisulcatus sp. n.

urn:lsid:zoobank.org:act:69A1A1CF-E8EA-4BAB-8A5A-0B32E4559A6D

Figs 247 –255 

##### Type material.

Holotype, ♀ (RMNH) “NW Vietnam: Tonkin, Hoang Lien N.R., 15 km W Sa Pa, c 1900 m, 15–21.x.1999, Malaise traps, C. v. Achterberg, RMNH’99”.

##### Diagnosis.

Therophilus daanyuanensis (Chen & Yang, 2006) comb. n. from Fujian (China) is similar but Therophilus daanyuanensis has no medio-posterior groove of the mesoscutum, the tegulae, the mesopleuron and the metapleuron partly orange brown, the first tergite distinctly longitudinally striate, the second tergite striate and with a wide curved transverse depression, the fore coxa and trochanter and trochantellus brownish-yellow and the middle tibia completely ivory.

##### Description.

Holotype, ♀, length of body 3.3 mm, of fore wing 3.1 mm, ovipositor sheath 2.5 mm.

###### Head.

Antenna with 27 segments, length of third segment 1.2 times fourth segment, length of third, fourth and penultimate segments 3.5, 3.0 and 1.5 times their width, respectively; length of maxillary palp 0.7 times height of head; malar space 2.3 times as long as basal width of mandible; in dorsal view length of eye 2.1 times temple; temple gradually narrowed posteriorly (Fig. 254); POL:OD:OOL = 8:5:10; face shiny and punctulate, with a small narrow groove medio-dorsally; clypeus largely smooth and weakly convex, mainly dorsally punctulate; frons with a weak medial ridge (Fig. 254), moderately concave anteriorly, smooth medially and sparsely punctulate laterally and with an indistinct triangular area in front of anterior ocellus; vertex and temple shiny, largely smooth and sparsely punctulate.

###### Mesosoma.

Length of mesosoma 1.3 times its height; pronotum smooth without carinae anteriorly, rather sparsely punctate dorso-posteriorly and posterior groove finely crenulate; area near lateral carina of mesoscutum crenulate; mesoscutum largely setose and smooth, only somewhat spaced punctulate, medio-posteriorly lobes flattened; notauli complete and narrowly crenulate, but somewhat widened posteriorly; scutellar sulcus 0.5 times as long as dorsal face of scutellum, moderately deep and with 3 carinae; scutellum shiny and smooth, subposterior crest obsolescent but with a rather wide and coarsely crenulate groove in front of it (Fig. 249) and medio-posterior depression narrow and finely crenulate; precoxal sulcus narrow, rather deep, narrowly crenulate and anteriorly absent (Fig. 248); mesopleuron below precoxal sulcus smooth as remainder of mesopleuron, setose (including speculum); metapleuron densely setose, dorsally rather densely and moderately punctate and ventral half reticulate-rugose; propodeum spaced reticulate, with transverse carina and dorsal areola (Fig. 249); propodeal spiracle small, 1.5 times as long as wide.

###### Wings.

Fore wing: second submarginal cell small and petiolate (Fig. 251); vein SR1 bent towards pterostigma; r:3-SR+SR1 = 3:43; r-m about twice as long as petiolus (Fig. 251); apical half of subbasal cell densely setose. Hind wing: vein M+CU as long as vein 1-M; setose near vein cu-a.

###### Legs.

Length of hind femur, tibia and basitarsus 3.6, 6.4 and 9 times their width, respectively; hind femur largely smooth, spaced punctulate and with medium-sized setae (Fig. 252); length of outer and inner spur of middle tibia both 0.45 times middle basitarsus, respectively; outer side of middle tibia with a row of 3 minute pegs and 2 minute pegs apically; length of outer and inner spurs of hind tibia 0.4 and 0.5 times hind basitarsus, respectively; tarsal claws with large lobe.

###### Metasoma.

First tergite widened posteriorly, sparsely and weakly striate, with dorsal carinae weakly developed, its length 1.5 times its apical width (Fig. 250); second tergite smooth, somewhat narrowed anteriorly and with a curved transverse groove (Fig. 250); remainder of metasoma (including weakly impressed second suture) smooth; ovipositor sheath 0.81 times as long as fore wing.

###### Colour.

Black; palpi pale yellowish; middle and hind spurs white; antenna, legs (but fore tibia brown and fore femur partly yellowish-brown, hind and middle basitarsi basally, hind tibia with a narrow basal and a wide submedial band white or ivory), tegulae, metasoma (but baso-ventrally pale brown), veins and pterostigma dark brown; wing membrane slightly infuscate.

##### Distribution.

NW Vietnam: Lao Cai.

**Figure 247.**
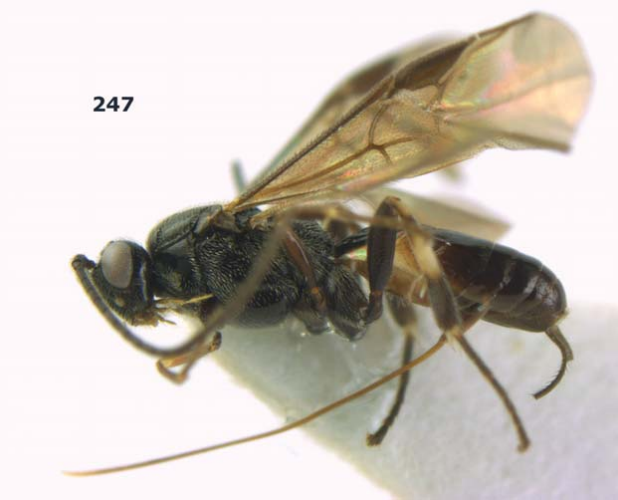
Therophilus crenulisulcatus sp. n., female, holotype. Habitus lateral.

**Figures 248–255.**
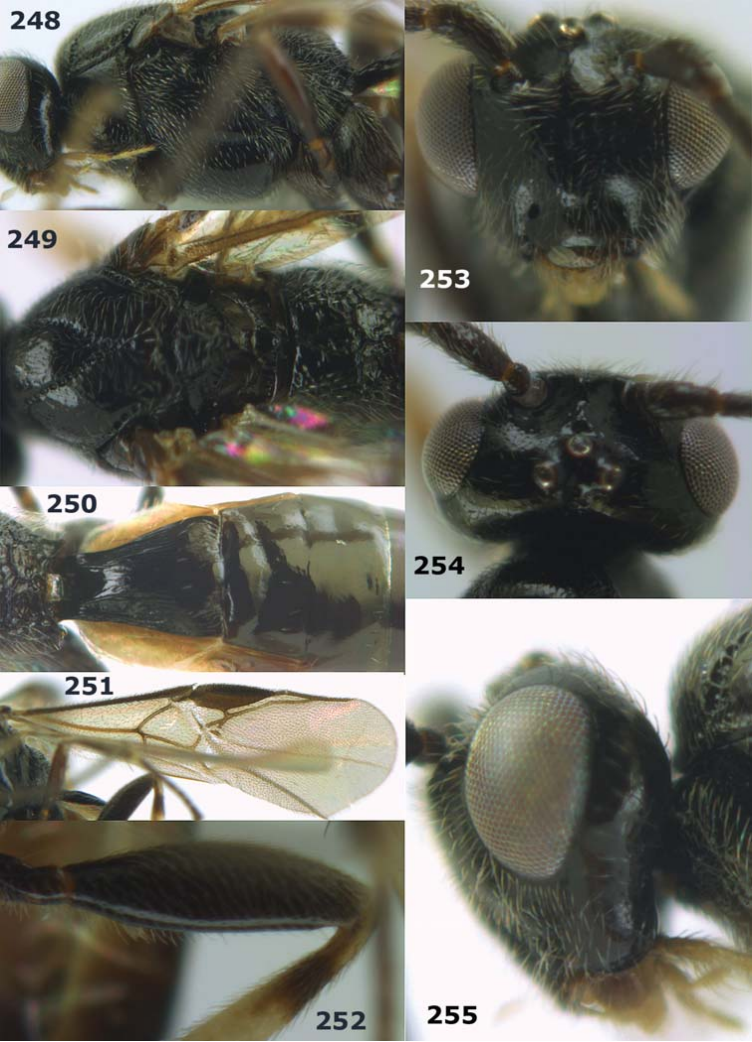
Therophilus crenulisulcatus sp. n., female, holotype. **248** mesosoma lateral **249** mesosoma dorsal **250** first-third metasomal tergites dorsal **251** wings **252** hind femur lateral **253** head anterior **254** head dorsal **255** head lateral.

##### Biology.

Unknown.

##### Etymology.

From “crenulatus” (Latin for “finely notched”), and “sulcus” (Latin for “groove”), because of the crenulate posterior groove of the pronotum.

#### Therophilus depressiferus sp. n.

urn:lsid:zoobank.org:act:7E491474-C571-4B5B-A1D6-5F2DF2A2D022

Figs 256 –266 

##### Type material.

Holotype, ♀ (RMNH), “N. Vietnam: Ninh Binh, Cuc Phuong N.P., n[ea]r centre, c 225 m, 29.vi-18.vii.2000, Mai Phu Quy, RMNH’01”. Paratypes (2 ♀): 1♀ (IEBR), same data as holotype; 1 ♀ (RMNH), “S. Vietnam: Dong Nai, Cat Tien N.P., Mal. traps, c 100 m, 9.iv.2007–13.v.2007, M.P. Quy, N.T. Manh & C. v. Achterberg, RMNH’07”.

##### Diagnosis.

The new species is similar to Therophilus rudimentarius (Enderlein, 1920) comb. n., but differs by having POL half as long as OOL (Therophilus rudimentarius:0.7 times); the pronotal trough crenulate medially, rugose-punctate ventrally and sparsely finely punctate dorsally (rudimentarius:entirely punctate); the mesosoma 1.8 times as long as high (rudimentarius:1.4 times); the first tergite 1.5 times as long wide apically (rudimentarius:1.6 times). The new species is similar to Bassus albifasciatus (Watanabe, 1934), but differs by having the tarsal claws with a distinct lobe (Bassus albifasciatus:simple); the first tergite 1.5 times as long as wide apically (albifasciatus:1.8 times) and the second tergite entirely black (albifasciatus: basal 0.6 reddish-brown to black).

##### Description.

Holotype, ♀, length of body 8.5 mm, of fore wing 7.4 mm, of ovipositor sheath 7.2 mm.

###### Head.

Antennal segments 45; length of third segment 1.3 times fourth segment, length of third, fourth and penultimate segments 2.8, 2.5 and 1.5 times their width, respectively; length of apical segment 2.3 times as long as penultimate segment; maxillary palp 0.8 times height of head; malar space 2.4 times as long as basal width of mandible; in dorsal view length of eye 4.8 times temple; ocelli in low triangle (Fig. 264), POL:OD:OOL= 7:6:13; face densely punctate (Fig. 263); frons smooth, laterally densely punctate and concave near antennal sockets (Fig. 264); vertex and temple shiny with sparse punctures.

###### Mesosoma.

Length of mesosoma 1.8 times its height; pronotum shiny with rugae ventrally, dorsally pronotum with dense fine punctures (Fig. 257); area near lateral carina of mesoscutum crenulate; lateral lobes of mesoscutum shiny, sparsely punctate, slightly flat posteriorly; middle lobe of mesoscutum with dense punctures medially, smooth laterally and apically; notauli deep and crenulate, united posteriorly forming short groove near scutellar sulcus; scutellar sulcus 0.7 times as long as dorsal face of scutellum and with 4 carinae; scutellum convex with sparse distinct punctures, subposterior crest transverse, interrupted medially (Fig. 258); precoxal sulcus wide, strongly crenulate (Fig. 257); mesopleuron below precoxal sulcus areolate-rugose anteriorly, rugose-punctate posteriorly; mesopleuron above precoxal sulcus largely smooth medially, distinctly punctate posteriorly; metapleuron reticulate-punctate dorsally, ventrally setose and largely rugose; propodeum largely areolate-rugose with 5 transverse carinae medially; propodeal spiracle medium-sized, 1.7 times as long as wide.

###### Wings.

Fore wing: second submarginal cell medium-sized triangular (Fig. 260); vein SR1 straight; r:3-SR+SR1=7:78. Hind wing: vein M+CU 0.8 times as long as vein 1-M.

###### Legs.

**Figure 256.**
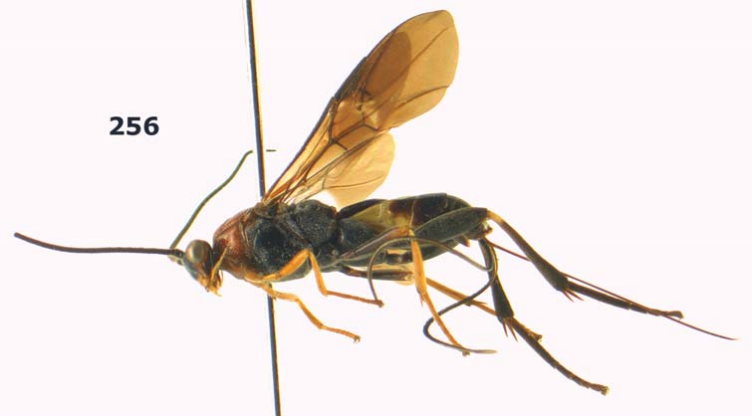
Therophilus depressiferus sp. n., female, holotype. Habitus lateral.

Length of hind femur, tibia and basitarsus 3.7, 6.7 and 8.0 times their width, respectively; hind femur (as remainder of legs) with short setae (Fig. 261); length of outer and inner spur of middle tibia 0.3 and 0.5 times middle basitarsus, respectively; outer side of middle tibia with 1 peg and a cluster of 5 pegs at apex; hind tibia distinctly narrowed basally and widened apically (Fig. 256); length of outer and inner spur of hind tibia 0.3 and 0.4 times hind basitarsus, respectively; outer side of hind tibia with a cluster of 12 pegs (figs); hind coxa distinctly punctate; hind femur reticulate-punctate; tarsal claws with a large lobe (Fig. 262).

###### Metasoma.

First tergite distinctly depressed laterally; length of first tergite 1.5 times its apical width (Fig. 259); dorsal carinae developed, intermingled with striae at apical third; first tergite longitudinally rugose-striate, coriaceous apically, area between striae granulate; second tergite with weak rugose transverse groove; basal area of second tergite with sparse setae and punctures (Fig. 259); ovipositor sheath 0.97 times as long as fore wing.

###### Colour.

Black; mouthparts, fore leg and middle tibia and tarsus yellow; medial round area of face, malar space and temple partly, mesoscutum, tegula reddish yellow; hind tibia (but basal pale yellow ring and yellow spurs) and tarsus brown; first-second metasomal tergites ivory ventrally; wing membrane dark brown, with pale band extending from vein 2-SR+M to vein 3-CU1.

###### Variation.

Length of body 8.2–9.3 mm, of fore wing 7.0–7.4 mm, vein M+CU 0.7–0.8 times vein 1-M, outer side of middle tibia with 2–12 pegs; second tergite without weak transverse groove; large reddish-yellow medial area of face may be fused with clypeus; entirely malar space and temple may be reddish-yellow; length of ovipositor sheath 1.0–1.1 times fore wing; wing membrane dark brown or yellowish-brown; basal ring of hind tibia white or pale brownish.

##### Distribution.

N Vietnam: Ninh Binh and S Vietnam: Dong Nai.

**Figures 257–266.**
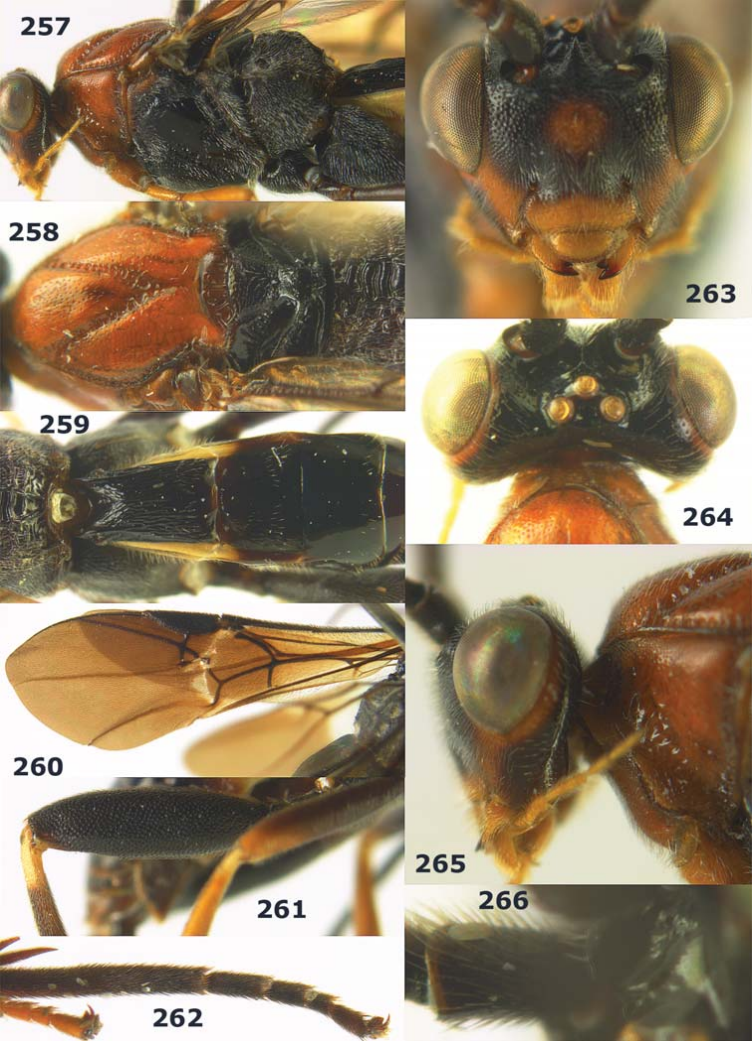
Therophilus depressiferus sp. n., female, holotype. **257** mesosoma lateral **258** mesosoma dorsal **259** first-third metasomal tergites dorsal **260** wings **261** hind femur lateral **262** hind tarsus lateral **263** head anterior **264** head dorsal **265** head lateral **266** frons dorso-lateral.

##### Biology.

Unknown.

##### Etymology.

From “depressus” (Latin for “pressed down”), and “fero” (Latin for “carry”), because of the depressed frons.

#### Therophilus elongator sp. n.

urn:lsid:zoobank.org:act:10E7D393-F094-4F44-8C90-B0A206129883

Figs 267 –275 

##### Type material.

Holotype, ♀ (RMNH), “C. Vietnam: Ha Tinh, Vu Quang N.P., 97 m, 18°19'43N; 105°26'29E, 23.ix.-5.x.2009, Mal[aise] trap 21, C. v. Achterberg & R. de Vries, RMNH’09”.

##### Diagnosis.

**Figure 267.**
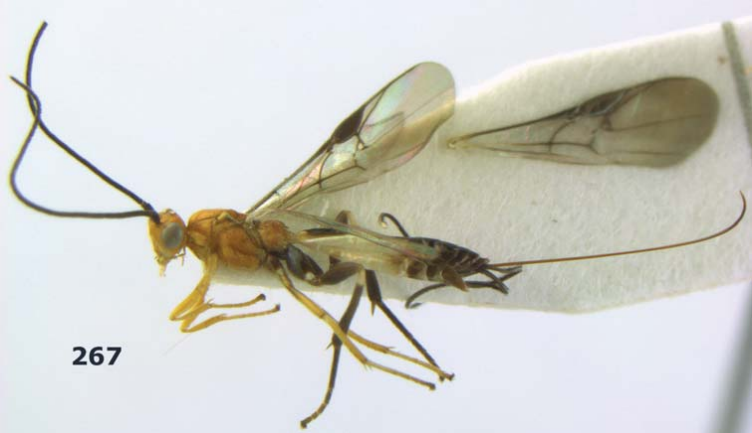
Therophilus elongator sp. n., female, holotype. Habitus lateral.

**Figures 268–275.**
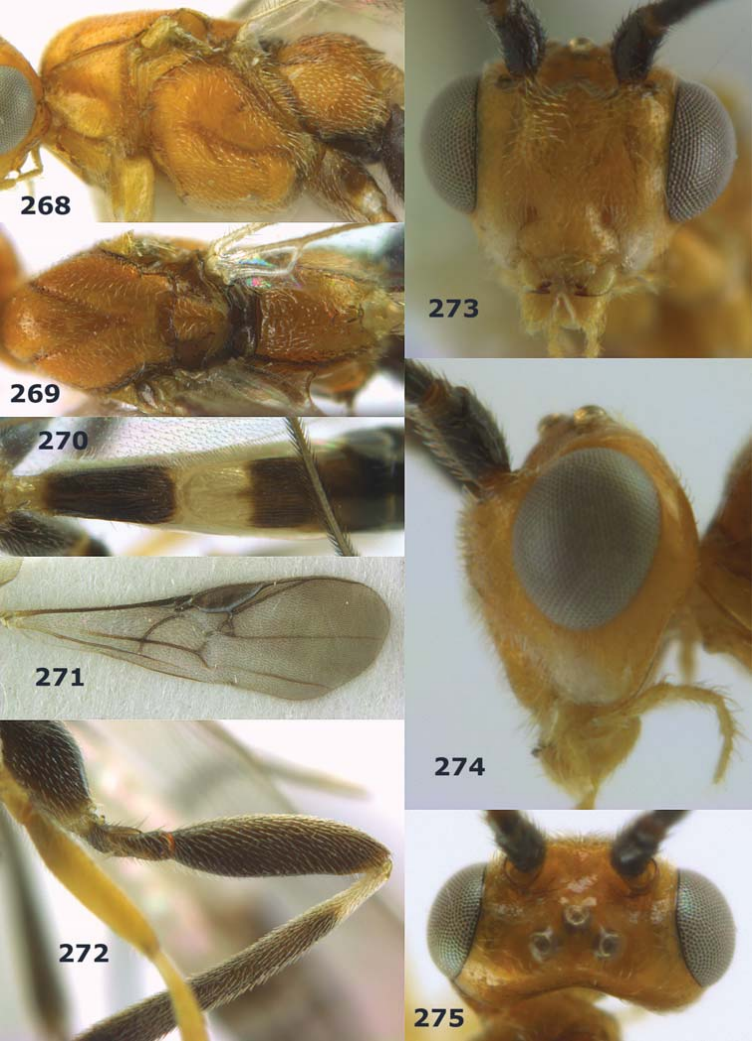
Therophilus elongator sp. n., female, holotype. **268** mesosoma lateral **269** mesosoma dorsal **270** first-third metasomal tergites dorsal **271** wings **272** hind femur lateral **273** head anterior **274** head lateral **275** head dorsal.

Close to Therophilus punctiscutum sp. n. and differs mainly by its pale colour (cf. Figs 267, 356) and more robust mesopleuron (cf. Figs 268, 357).

##### Description.

Holotype, ♀, length of body 4.6 mm, of fore wing 3.7 mm, ovipositor sheath 4.3 mm.

###### Head.

Antenna with 35 segments, length of third segment 1.3 times fourth segment, length of third, fourth and penultimate segments 3.8, 3.0 and 1.7 times their width, respectively; length of maxillary palp 0.7 times height of head; malar space 1.8 times as long as basal width of mandible; in dorsal view length of eye 4.0 times temple; temple directly narrowed posteriorly (Fig. 275); ocelli in low triangle, POL:OD:OOL= 8:5:11; face shiny, largely smooth and punctulate; clypeus largely smooth and moderately convex; frons with obsolete medial ridge, with wide flattened triangular area in front of anterior ocellus, weakly depressed behind antennal sockets, smooth but sparsely punctulate laterally; vertex and temple shiny and smooth, but temple with sparse punctures.

###### Mesosoma.

Length of mesosoma 1.7 times its height; pronotum largely smooth, but with three carinae anteriorly, with some punctures dorso-posteriorly and posterior groove finely crenulate; area near lateral carina of mesoscutum indistinctly crenulate; mesoscutum spaced punctulate, medio-posteriorly lobes flattened posteriorly; notauli complete and narrowly crenulate, ending distinctly in front of scutellar sulcus; scutellar sulcus half as long as dorsal face of scutellum, shallow, curved and with 3 short carinae; scutellum shiny and smooth (except for some punctures), subposterior crest absent (Fig. 269) and medio-posterior depression narrow, small and smooth; mesopleuron comparatively robust (Fig. 268); precoxal sulcus narrow, rather deep, narrowly crenulate and anteriorly absent (Fig. 268); mesopleuron below precoxal sulcus spaced punctulate; remainder of mesopleuron shiny and largely smooth, but distinctly punctate below scrobe; metapleuron moderately densely setose, dorsally rather coarsely punctate and ventrally rugose; dorsal face of propodeum elongate, moderately rugose, without median carina, and with a small incomplete areola posteriorly (Fig. 269); propodeal spiracle rather small and round.

###### Wings.

Fore wing: second submarginal cell medium-sized and petiolate, petiolus 0.7 times vein r-m and sides of cell curved (Fig. 271); vein SR1 straight; vein 1-R1 distinctly shorter than vein 2-R1; vein r very short, r:3-SR+SR1 = 1:52; apical half of subbasal cell moderately setose. Hind wing: vein M+CU 0.6 times as long as vein 1-M; surroundings of vein cu-a largely glabrous.

###### Legs.

Length of hind femur, tibia and basitarsus 3.8, 8.9 and 9 times their width, respectively; hind femur finely pimply or punctulate and with short setae (Fig. 272); length of outer and inner spur of middle tibia 0.3 and 0.4 times middle basitarsus, respectively; outer side of middle tibia with a row of 3 pegs and 2 pegs at apex; length of outer and inner spurs of hind tibia 0.2 and 0.4 times hind basitarsus, respectively; tarsal claws with large lobe.

###### Metasoma.

First tergite subparallel-sided, with distinct dorsal carinae in its basal half, its length 2.5 times its apical width and rather coarsely longitudinally striate (Fig. 270); second tergite elongate and anteriorly slightly narrowed (Fig. 270), densely and moderately coarsely striate and with a distinct transverse groove; remainder of metasoma (including shallow second suture) smooth; ovipositor sheath 1.18 times as long as fore wing.

###### Colour.

Yellowish-brown; antenna blackish-brown; tegulae, middle coxa, base of middle femur and hind spurs brown; malar space, base of first tergite, basal half of second tergite, ventral half of metasoma and basal ring of hind tibia white; remainder of metasoma and of hind leg dark brown; mandible ivory; palpi, fore leg and remainder of middle leg brownish-yellow; veins and pterostigma dark brown; wing membrane subhyaline but slightly infuscate apically.

##### Distribution.

C Vietnam: Ha Tinh.

##### Biology.

Unknown.

##### Etymology.

From “elongatus” (Latin for “prolonged”), because of the elongated first metasomal tergite.

#### Therophilus festivus

(Muesebeck, 1953) comb. n.

Figs 276–280 

##### Distribution.

NE Vietnam: Ha Giang, Hoa Binh, Vinh Phuc; NW Vietnam: Lao Cai; C Vietnam: Quang Tri and S Vietnam: Dong Nai. Outside Vietnam known from China (Jiangsu; Shandong; Shanghai; Taiwan; Zhejiang); India; Japan (including Okinawa); Korea; Nepal; Philippines (Mindoro); Russia (Far East). Introduced into U.S.A.

##### Biology.

Recorded as a parasitoid of Tortricidae, Pyralidae, Blastobasidae, Carposinidae, Noctuidae, Gelechiidae and Cossidae, but no reared material seen by us and such a wide host range seems most improbable.

**Figures 276–280.**
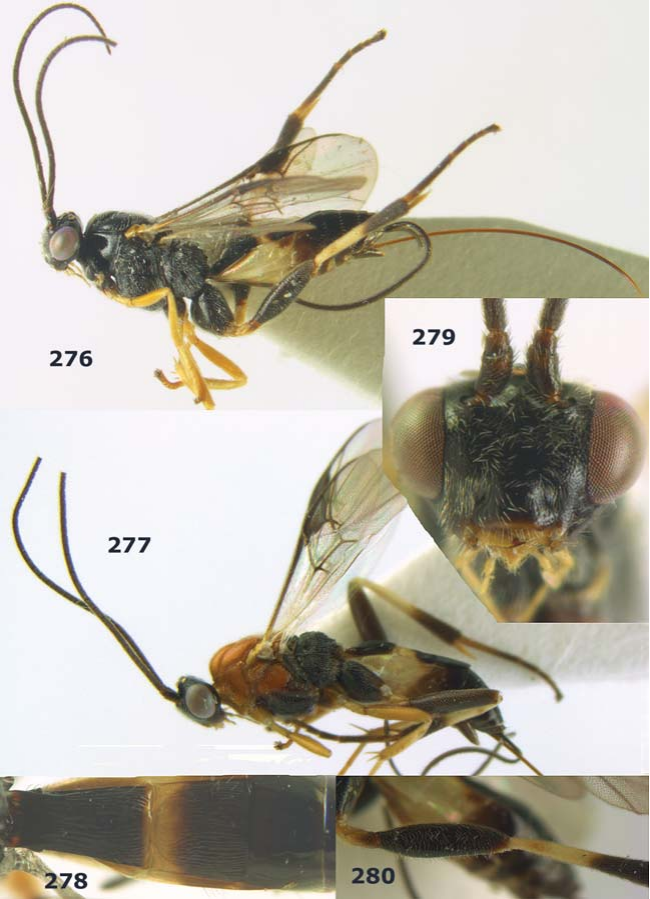
Therophilus festivus (Muesebeck), female, Cat Tien National Park, including pale form (277). **276**, **277** habitus lateral **278** first-third metasomal tergites dorsal **279** head anterior **280** hind femur lateral.

##### Note.

One female from Cat Tien N.P. (Ficus trail, 9–30.iv.2007) is a pale form with the pronotum, the mesopleuron dorsally and the mesoscutum orange brown (Fig. 277); normally the mesosoma is completely black (Fig. 276). The basal cell of the fore wing is densely or sparsely setose.

#### Therophilus javanus

(Bhat & Gupta, 1977) comb. n.

Figs 281–284 

##### Distribution.

N Vietnam: Bac Ninh, Ha Noi, C Vietnam: Quang Nam and S Vietnam: Lam Dong. Outside Vietnam known from Indonesia (Java) and Malaysia.

**Figures 281–284.**
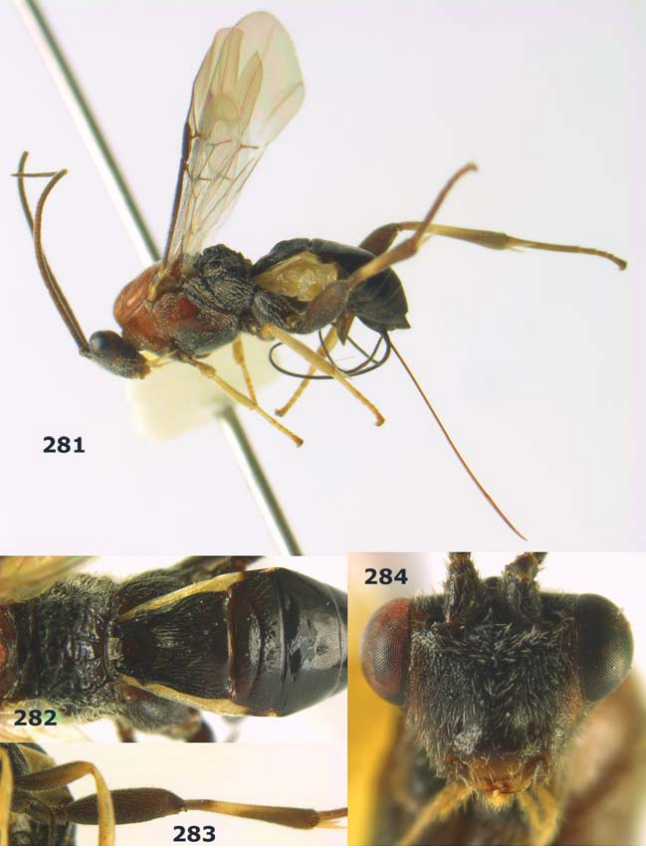
Therophilus javanus (Bhat & Gupta), female, Quang Nam. **281** habitus lateral **282** propodeum and first-third metasomal tergites dorsal **283** hind femur lateral **284** head anterior.

##### Biology.

Parasitoid of Pyralidae.

#### Therophilus levisoma sp. n.

urn:lsid:zoobank.org:act:6ED66F41-7206-46DA-A155-366BF0BEA25D

Figs 285 –293 

##### Type material.

Holotype, ♀ (RMNH) “NW Vietnam: Tonkin, Hoang Lien N.R., 15 km W Sa Pa, c 1900 m, 15–21.x.1999, Malaise traps, C. v. Achterberg, RMNH’99”. Paratypes: 2 ♀ + 2 ♂, (RMNH, IEBR), same data as holotype.

##### Diagnosis.

The new species is similar to Therophilus sungkangensis (Chou & Sharkey, 1989) comb. n., but differs by having the propodeum with a strong transverse carina dividing propodeum in anterior and posterior areas and its posterior half steep (Therophilus sungkangensis: without transverse carina and posteriorly convex); precoxal sulcus narrow, largely crenulate (sungkangensis: rather wide and anterior 0.4 absent); notauli strongly crenulate (sungkangensis: weakly crenulate); the new species is also similar to Therophilus rugosiferus sp. n., but differs by having the apical half of the second tergite smooth (Therophilus rugosiferus: apical half striate; vein M+CU of the hind wing as long as vein 1-M (rugosiferus: 0.8 times) and the outer and inner spurs of the middle tibia subequal (rugosiferus: inner spur 1.7 times longer than outer spur). The new species is similar to Therophilus daanyuanensis (Chen & Yang, 2006) comb. n. from Fujian (China), because of the coarsely crenulate notauli and the absence of a medio-posterior groove of the mesoscutum. However, Therophilus daanyuanensis has the frons largely smooth (only punctulate), the tegulae, the mesopleuron and the metapleuron partly orange brown, the marginal cell of the fore wing comparatively narrow basally, the first tergite distinctly longitudinally striate, the second tergite striate and with a wide curved transverse depression, the fore coxa and trochanter and trochantellus brownish-yellow and the middle tibia completely ivory. Bassus canaliculatus Chen & Yang, 2006, from China, is superficially similar, but has a distinct medio-posterior groove of mesoscutum and finely crenulate notauli, head and mesosoma completely black, pronotum ventrally and fore coxa blackish; simple tarsal claws and depression of second tergite distinctly sinuate. Therophilus tonghuaensis (Chen & Yang, 2006) comb. n. from China belongs also here and differs from Bassus canaliculatus by having the fore coxa pale yellowish, vein r-m of the fore wing short, resulting in a minute second submarginal cell, hind femur brownish and less robust and the transverse depression of the second tergite is nearly straight.

##### Description.

Holotype, ♀, length of body 4.1 mm, of fore wing 4.0 mm, ovipositor sheath 3.1 mm.

###### Head.

Antennal segments 32, length of third segment 1.3 times fourth segment, length of third, fourth and penultimate segments 5.0, 4.0 and 2.0 times their width, respectively; length of apical antennal segment 1.5 times as long as penultimate segment; maxillary palp 0.7 times height of head; malar space 2.8 times as long as basal width of mandible; in dorsal view length of eye 2.7 times temple; temple roundly narrowed posteriorly (Fig. 293); ocelli in high triangle, POL:OD:OOL= 8:4:10; face distinctly punctate laterally, rugose-punctate medially; frons densely punctate; vertex and temple shiny with very sparse minute punctures.

###### Mesosoma.

Length of mesosoma 1.5 times its height; pronotum largely smooth, ventrally punctate, dorsally with distinct punctures; area near lateral carina of mesoscutum crenulate; mesoscutum shiny, sparsely punctate and setose; middle lobe of mesoscutum shiny and punctate; notauli narrow anteriorly, widened posteriorly, largely crenulate (Fig. 287); scutellar sulcus about as long as dorsal part of scutellum and with 4 carinae; scutellum largely smooth with very sparse fine punctures, subposteriorly with a transverse elevation (Fig. 287); precoxal sulcus short, narrow and largely crenulate; mesopleuron shiny with very sparse fine punctures, area near precoxal sulcus smooth; metapleuron densely setose, rugose-punctate; propodeum irregularly rugose and divided in two areas by a transverse carina (Fig. 287), apical half rather steep; propodeal spiracle small, as long as wide.

###### Wings.

Fore wing: second submarginal cell small and petiolate (Fig. 289); vein SR1 straight; r:3-SR+SR1=3:63. Hind wing: vein M+CU as long as vein 1-M.

###### Legs.

Length of hind femur, tibia and basitarsus 3.3, 5.4 and 9.5 times their width, respectively; hind femur (as remainder of legs) with short setae; outer and inner spurs of middle tibia subequal and 0.5 times as long as middle basitarsus, respectively; outer side of middle tibia with 4 pegs; hind tibia compressed basally and distinctly widened apically; length of outer and inner spur of hind tibia 0.3 and 0.5 times hind basitarsus, respectively; outer apex of hind tibia with a cluster of 9 pegs; tarsal claws with lobe.

###### Metasoma.

First tergite slightly depressed laterally, 1.3 times as long as its apical width (Fig. 288), shiny with some longitudinal striae, smooth apically; second tergite with V-shape transverse groove (Fig. 288); remainder of metasoma shiny and smooth; ovipositor sheath 0.8 times as long as fore wing.

###### Colour.

**Figure 285.**
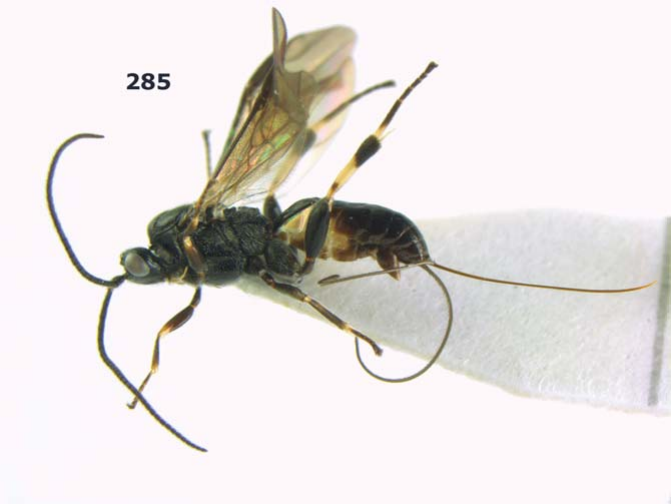
Therophilus levisoma sp. n., female, holotype. Habitus lateral.

Black; fore leg brown (but coxa, trochanter, trochantellus and largely femur dark brown); basal ring of middle tibia and one third of middle basitarsus, two thirds of hind tibia (except small patch (Figs 285, 290), one third of hind basitarsus and spurs ivory; pterostigma dark brown; wing membrane infuscate.

###### Variation.

Antennal segments of male 30 and of female 28–32; length of body of female 3.3–4.1 mm and of male 3.7 mm; length of fore wing of female 2.6–4.0 mm and of male 3.9 mm; face entirely punctate or partly rugose-punctate; apical antennal segment 1.2 times as long as penultimate segment; outer side of middle tibia with a row of 3 pegs; fore leg yellow or largely dark brown and brown; frons smooth to punctate laterally.

##### Distribution.

NW Vietnam: Lao Cai.

**Figures 286–293.**
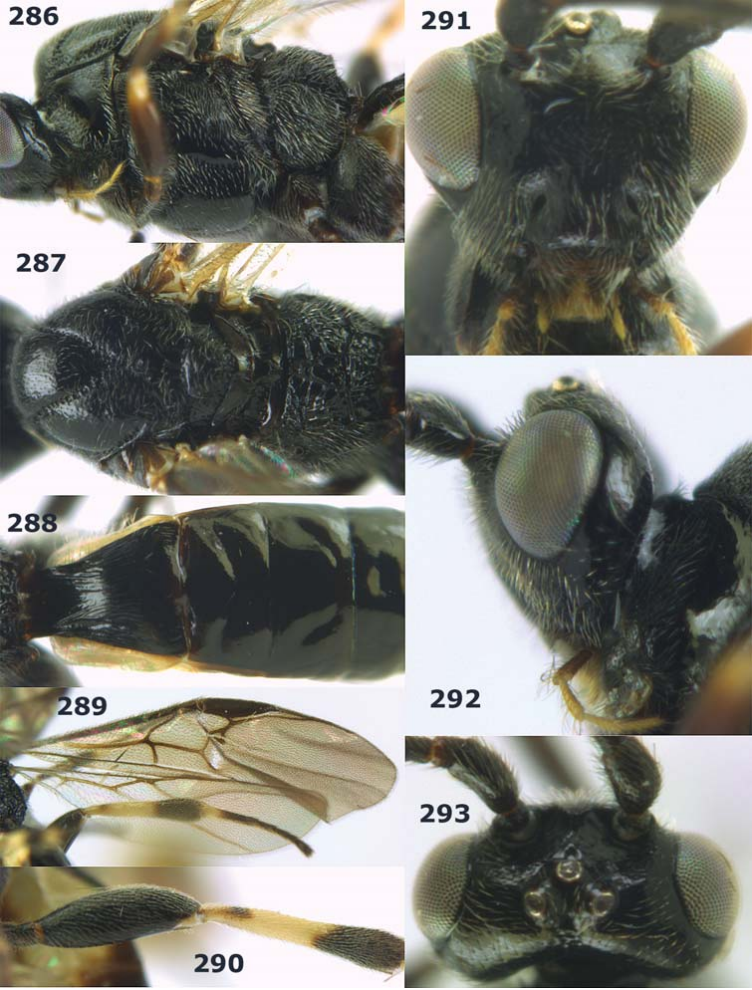
Therophilus levisoma sp. n., female, holotype. **286** mesosoma lateral **287** mesosoma dorsal **288** first-third metasomal tergites dorsal **289** wings **290** hind femur lateral **291** head anterior **292** head lateral **293** head dorsal.

##### Biology.

Unknown.

##### Etymology.

From “levis” (Latin for “smooth”), and “soma” (Greek for “body”), because of the smooth body.

#### Therophilus lienhuachihensis

(Chou & Sharkey, 1989) comb. n.

Figs 294–296 

##### Distribution.

NE Vietnam: Ha Giang, Vinh Phuc and NC Vietnam: Ha Tinh. Outside Vietnam known from China (Taiwan).

**Figures 294–296.**
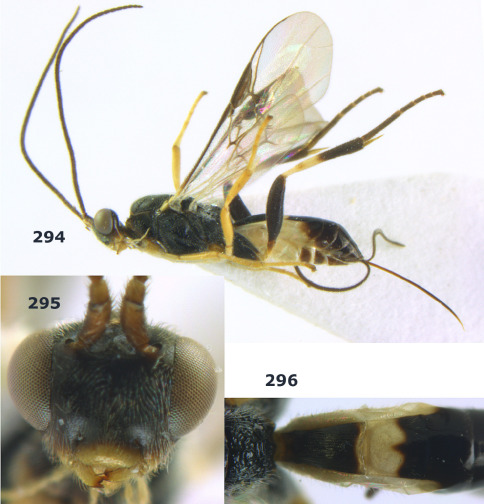
Therophilus lienhuachihensis (Chou & Sharkey), female, Thanh Son. **294** habitus lateral **295** head anterior **296** propodeum and first-third metasomal tergites dorsal.

#### Therophilus marshi

(Bhat & Gupta, 1977) comb. n.

Figs 297–300 

##### Distribution.

NE Vietnam: Ha Giang; NC Vietnam: Ha Tinh and S Vietnam: Dong Nai. Outside Vietnam known from East Malaysia (Sabah).

**Figures 297–300.**
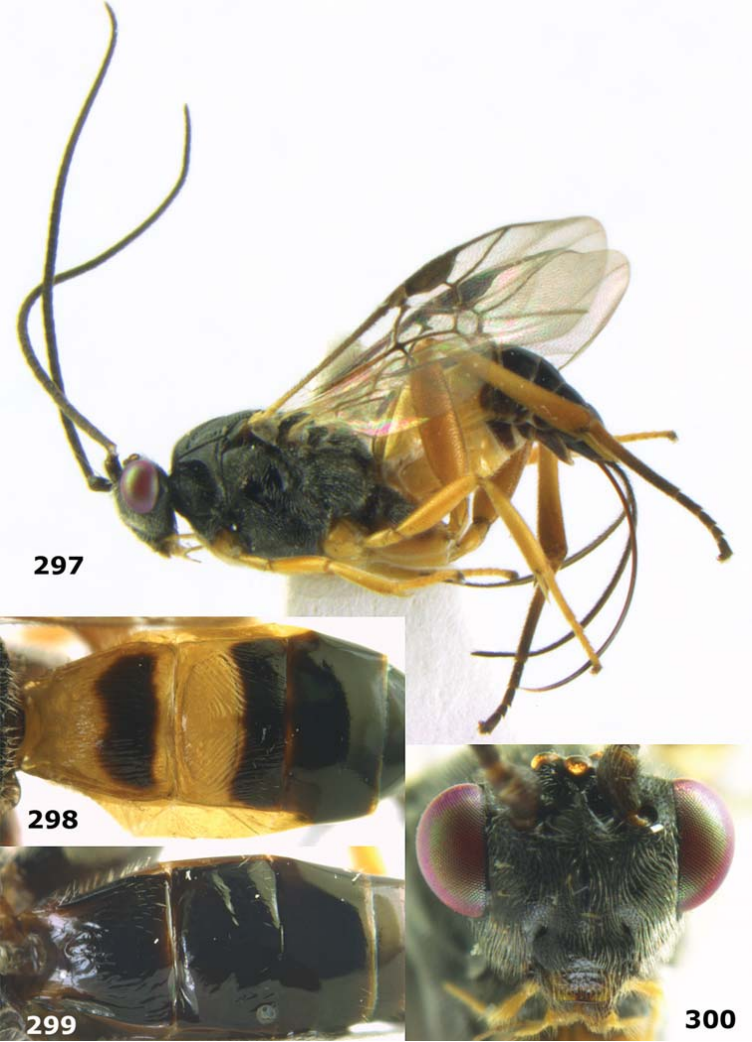
Therophilus marshi (Bhat & Gupta), female (but 299 of dark male), Cat Tien National Park. **297** habitus lateral **298**, **299** first-third metasomal tergites dorsal **300** head anterior.

##### Note.

The females have the humeral plate pale yellow and distinctly contrasting with the dark brown tegulum; males have the humeral plate partly dark brown and less contrasting.

#### Therophilus marucae sp. n.

urn:lsid:zoobank.org:act:077EC951-CD21-4C5D-872B-AF622F50E878

Figs 301 –309 

##### Type material.

Holotype, ♀ (RMNH), Aga. 309, “NE Vietnam: Ha Noi, Tu Liem, ex Maruca vitrata, [on black] bean, 27.v.2007, D.Th. Hoa”. Paratypes (2 ♀ + 8 ♂ + 1 unknown): 1 ♀ + 1 ♂ (IEBR), Aga.131 and Aga.132, id., but 10–20.v.2003, D.Th. Dung; 1 ♂, (IEBR), Aga. 334, id., but 20.vii.2007, K.D. Long; 1 ♀ + 1 ♂ (RMNH), Aga. 102, Aga. 107, “S. Vietnam: Lam Dong, Duc Trong, 1000 m, [on] black bean, 28.vii.2002, K.D. Long; 1 ♂ + 1 unknown (IEBR, RMNH), Aga. 003 (missing apex of metasoma; ex Maruca vitrata) and Aga. 019, “NE Vietnam: Bac Ninh, Tien Son, [on] soybean, 25.v.1995, K.D. Long”; 1 ♂ (IEBR), Aga. 011 and 1 ♂ (RMNH), Aga. 027, id., but Minh Dao, on black bean, 1.vi.1995, K.D. Long; 2 ♂ (IEBR, RMNH), Aga. 245 and Aga. 245a, “N. Vietnam: Ha Noi, Soc Son, beans + peanut, 26.v.2004, K.D. Long”.

##### Diagnosis.

The new species is similar to Therophilus asper (Chou & Sharkey, 1989) comb. n., but differs by having the malar space 3.4 times as long as basal width of the mandible (Therophilus asper: 2.7 times); the first tergite with distinct dorsal carinae (asper: dorsal carinae indistinct); the precoxal sulcus punctate anteriorly (asper: absent anteriorly); the mesoscutum rugose-striate apically (asper: rugose); the outer side of the middle tibia with 2 rows of 2 pegs at apex (asper: with 7 pegs). The new species is close to Therophilus luzonicus (Bhat & Gupta, 1977) comb. n., but differs by having mesoscutum densely rugose-punctate and rugose-striate apically (Therophilus luzonicus: sparsely punctate); the scutellar sulcus with 3 carinae (luzonicus: with 1 carina) and the scutellum rugose-punctate (luzonicus: sparsely punctate). The new species is also similar to Therophilus muesebecki (Bhat & Gupta, 1977) comb. n., but differs by having ; the transverse groove on the second tergite present (Therophilus muesebecki: absent); the scutellum dull and rugose-punctate (muesebecki: sparsely finely punctate) and the areola of the propodeum with 4 transverse carinae (Therophilus muesebecki: with 2 carinae).

##### Description.

Holotype, ♀, length of body 4.6 mm, of fore wing 3.6 mm, ovipositor sheath 3.1 mm.

###### Head.

Antennal segments 37, length of third segment 1.3 times fourth segment, length of third, fourth and penultimate segments 2.5, 2.0 and 1.0 times their width, respectively; length of apical antennal segment twice as long as penultimate segment; length of maxillary palp 0.6 times height of head; malar space 3.4 times as long as basal width of mandible; in dorsal view length of eye 3.0 times temple; temple roundly narrowed posteriorly (Fig. 308); POL:OD:OOL = 10:6:14; face with dense distinct punctures; frons, vertex and temple shiny and smooth.

###### Mesosoma.

Length of mesosoma 1.4 times its height; pronotum smooth with sparse fine punctures dorsally, crenulate posteriorly; area near lateral carina of mesoscutum crenulate; lateral and middle lobes of mesoscutum densely rugose-punctate, rugose-striate apically; notauli deep and crenulate, fused with scutellar sulcus posteriorly forming a groove; scutellar sulcus 0.6 times as long as dorsal face of scutellum and with 3 carinae; scutellum convex, slightly narrowed, rugose-punctate, subposterior crest sinuate (Fig. 303); precoxal sulcus wide, largely punctate anteriorly and crenulate posteriorly (Fig. 302); mesopleuron below precoxal sulcus with dense distinct punctures; mesopleuron above precoxal sulcus shiny and smooth medially, sparsely finely punctate anteriorly, densely moderately punctate posteriorly; metapleuron setose, reticulate-rugose dorsally, ventrally with strong rugae; propodeum with large areola and costulae developed, area of areola with 4 transverse carinae (Fig. 303); propodeal spiracle large, 1.5 times as long as wide.

###### Wings.

Fore wing: second submarginal cell small and petiolate (Fig. 305); vein SR1 straight; r:3-SR+SR1 = 5:18. Hind wing: vein M+CU slightly longer vein 1-M (17:16).

###### Legs.

Length of hind femur, tibia and basitarsus 3.4, 5.4 and 9.2 times their width, respectively; hind femur (as remainder of legs) with short setae and coarsely punctate (Fig. 306); length of outer and inner spur of middle tibia 0.4 and 0.7 times middle basitarsus, respectively; outer side of middle tibia with 2 rows of 2 pegs at apex; length of outer and inner spur of hind tibia 0.3 and 0.5 times hind basitarsus, respectively; hind tibia with cluster of 7 pegs; tarsal claws with lobe.

###### Metasoma.

Length of first tergite 1.1 times its apical width (Fig. 304), with a short carina medially, dorsal carinae developed, intermingled with striae apically; first tergite shiny, strongly depressed and smooth basally, moderately striate apically; second tergite with transverse groove, shiny and smooth basally, weakly striate apically (Fig. 304), ovipositor sheath 0.8 times as long as fore wing.

###### Colour.

Black; antenna light brown (but scapus dark brown); tegulae dark brown; fore and middle legs pale yellow; fore and middle coxae brown; hind tibia and tarsus dark yellow; pterostigma dark brown; wing membrane subhyaline.

###### Variation.

Antennal segments of both sexes 35–37; length of body of female 4.2–5.0 mm, and of male 4.3–4.6 mm, length of fore wing of female 3.6–4.2 mm, of male 3.7–4.2 mm, apical antennal segment 1.4–2.0 times as long as penultimate segment; vein M+CU of hind wing 0.9–1.1 times as long as vein 1-M; length of first tergite (both sexes) 1.0–1.1 times as long as its apical width; basal area of second tergite weakly rugose or smooth.

##### Distribution.

NE Vietnam: Bac Ninh, Ha Noi; CN Vietnam: Quang Binh and S Vietnam: Lam Dong.

**Figure 301.**
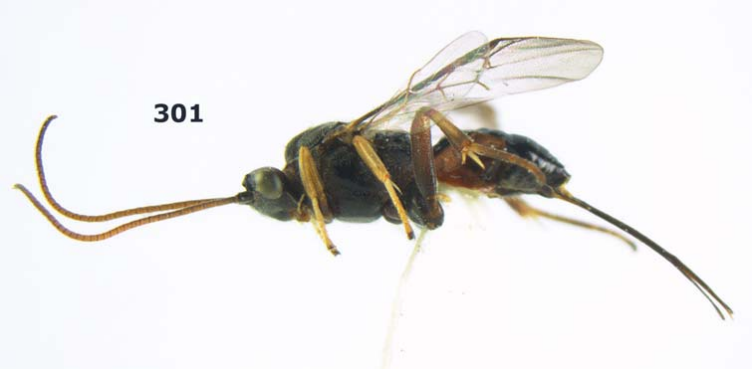
Therophilus marucae sp. n., female, holotype. Habitus lateral.

**Figures 302–309.**
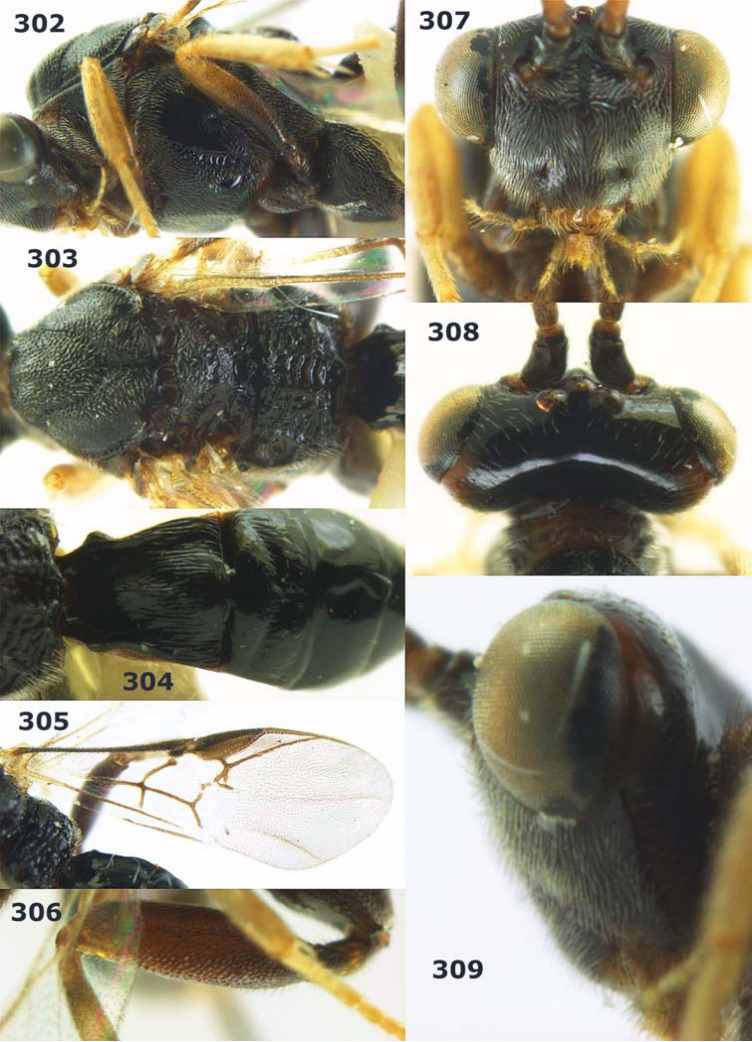
Therophilus marucae sp. n., female, holotype. **302** mesosoma lateral **303** mesosoma dorsal **304** first-third metasomal tergites dorsal **305** wings **306** hind femur lateral **307** head anterior **308** head dorsal **309** head lateral.

##### Biology.

According to the label data parasitoid of the pod bean borers Maruca vitrata (Fabricius) and Etiella zinckenella (Treitschke) (Pyralidae) on soybean (Glycine max (Linnaeus)) and black bean (Dumasia villosa DC.), but no host remains are preserved.

##### Etymology.

Named after the generic name of one of its hosts: Maruca.

#### Therophilus mellisoma sp. n.

urn:lsid:zoobank.org:act:B7153BF0-476E-4C91-9F05-FF25DCFE22BA

Figs 310 –319 

##### Type material.

Holotype, ♀ (RMNH), Aga. 152, “NE Vietnam: Hoa Binh, Yen Thuy, orchard, MT, 20–30.x.2002, K.D. Long”. Paratypes (2 ♀): 1 ♀ (IEBR), Aga. 153, same data as holotype; 1 ♀ (RMNH), Aga. 294, “CN Vietnam: Nghe An, Con Cuong, Pu Mat N.P., 13.iv.2006, P.Th. Nhi”.

##### Diagnosis.

The new species is similar to Therophilus similis (Bhat & Gupta, 1977) comb. n., but differs by having the malar space 3.3 times as long as basal width of the mandible (Therophilus similis: 2.3–2.6 times) and the mesoscutum rugose-punctate anteriorly, sparsely punctate posteriorly (similis: sparsely minutely punctate). The new species is also similar to Therophilus rudimentarius (Enderlein, 1920) comb. n., but differs by having the malar space 3.3 times as long as the basal width of the mandible (Therophilus rudimentarius: 2.2–2.6 times); the pronotum smooth (rudimentarius: minutely punctate and trough crenulate) and the first tergite 1.3 times as long as its apical width (rudimentarius: 1.6–1.7 times).

##### Description.

Holotype, ♀, length of body 4.1 mm, of fore wing 3.4 mm, of ovipositor sheath 1.9 mm.

###### Head.

Antennal segments 31, length of third segment 1.1 times fourth segment, length of third, fourth and penultimate segments 3.8, 3.4 and 1.5 times their width, respectively; apical antennal segment as long as penultimate segment; maxillary palp 0.6 times height of head; malar space 3.3 times as long as basal width of mandible; in dorsal view length of eye 4.0 times temple; ocelli in high triangle (Fig. 307), POL:OD:OOL = 8:4:11; face distinctly finely punctate medially, slightly rugose-punctate medially; frons smooth; vertex and temple smooth with very sparse minute punctate.

###### Mesosoma.

Length of mesosoma 1.5 times its height; pronotum smooth, crenulate anteriorly; area near lateral carina of mesoscutum sparsely crenulate; mesoscutum rugose-punctate anteriorly, sparsely punctate posteriorly; middle lobe of mesoscutum with distinct longitudinal elevation medially; notauli complete, sparsely crenulate; scutellar sulcus 0.5 times as long as dorsal face of scutellum and with 3 carinae; scutellum with sparse distinct punctures with rugose subposterior carina (Fig. 312); precoxal sulcus complete and crenulate (Fig. 311); mesopleuron below precoxal sulcus with distinct punctures; mesopleuron above precoxal sulcus smooth, near precoxal sulcus punctate; metapleuron distinctly punctate, rugose ventrally; propodeum largely reticulate-rugose; propodeal spiracle medium-sized, 1.3 times as long as wide.

###### Wings.

Fore wing: second submarginal cell obsolescent and with long petiolus (Figs 314, 319); vein SR1 curved, r:3-SR+SR1 = 3:47. Hind wing: vein M+CU 0.8 times as long as vein 1-M.

###### Legs.

Length of hind femur, tibia and basitarsus 3.3, 5.4 and 8.6 times their width, respectively; hind femur (as remainder of legs) with short setae (Fig. 315); length of outer and inner spur of middle tibia 0.3 and 0.5 times middle basitarsus, respectively; outer side of middle tibia with a row of 4 pegs and 3 pegs at apex; length of outer and inner spur of hind tibia 0.3 and 0.5 times hind basitarsus, respectively; tarsal claws with lobe.

###### Metasoma.

Length of first tergite 1.3 times its apical width (Fig. 313), its surface weakly striate, smooth apically; second tergite 0.5 times as long as its apical width and 0.8 times as long as third tergite medially (Fig. 313); remainder of metasoma smooth; ovipositor sheath 0.6 times as long as fore wing.

###### Colour.

Brownish-yellow; antenna, vein SC+C+R, pterostigma, 1-SR1, apex of hind tibia apically, hind tarsus and ovipositor sheath dark brown; wing membrane subhyaline.

###### Variation.

Antenna with 31 segments; length of body 3.6–4.1 mm, length of fore wing 3.3–3.4 mm; vein M+CU of hind wing 0.8–0.9 times as long as vein 1-M; length of first tergite 1.3–1.5 times its apical width.

##### Distribution.

NE Vietnam: Hoa Binh and CN Vietnam: Nghe An.

**Figure 310.**
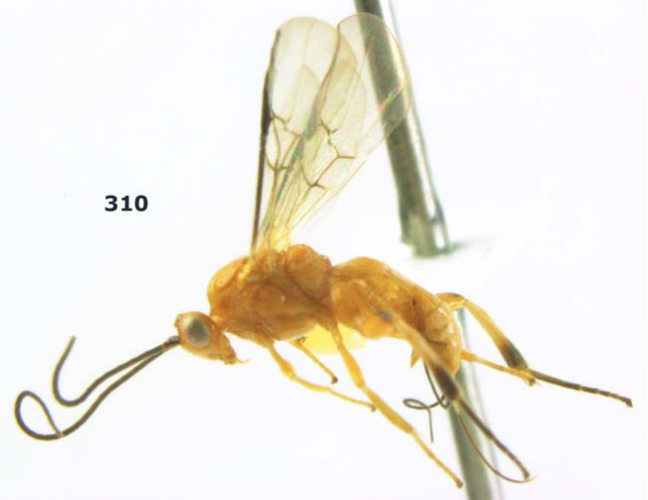
Therophilus mellisoma sp. n., female, holotype. Habitus lateral.

**Figures 311–319.**
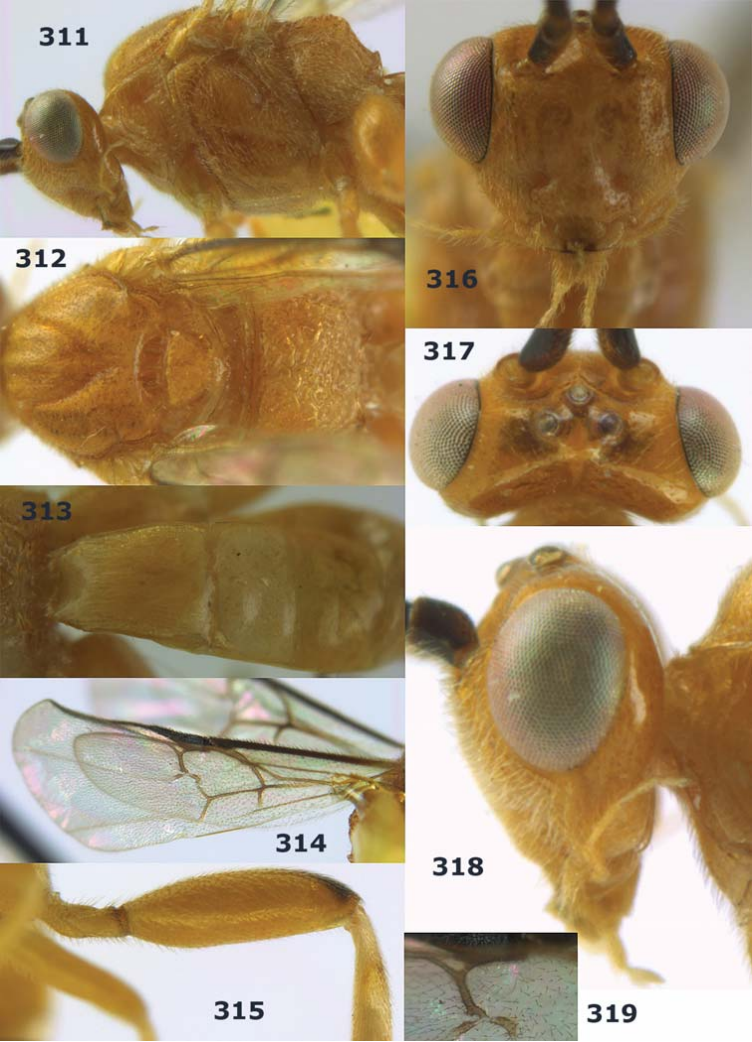
Therophilus mellisoma sp. n., female, holotype. **311** mesosoma lateral **312** mesosoma dorsal **313** first-third metasomal tergites dorsal **314** wings **315** hind femur lateral **316** head anterior **317** head dorsal **318** head lateral **319** detail second submarginal cell of fore wing.

##### Biology.

Unknown.

##### Etymology.

From “mellitus” (Latin for “pertaining to honey”), and “soma” (Greek for “body”), because of the honey coloured body.

#### Therophilus nigrolineatus sp. n.

urn:lsid:zoobank.org:act:66B5CD51-B754-4EDE-8FA9-016C6864EA30

Figs 320 –328 

##### Type material.

Holotype, ♀ (RMNH), “S. Vietnam: Dong Nai, Cat Tien N.P., Crocodile tr[ail], Mal. traps, c 100 m, 13–19.v.2007, C. van Achterberg & R. de Vries, RMNH’07”. Paratypes (15 ♀ + 4 ♂): 1 ♀ + 1 ♂ (RMNH, IEBR), same data as holotype; 7 ♀ (RMNH, IEBR), id., but 10–30.iv.2007, M.P. Quy & N.T. Manh; 1 ♀ (RMNH), id., but 9–26.iv.2007; 2 ♀ (RMNH, IEBR), id., but 9.iv-13.v.2007, M.P. Quy, N.T. Manh & C. v. Achterberg; 2 ♀ + 3 ♂, id., but bird trail, Malaise traps 30–35, 15–20.v.2007, C. van Achterberg & R. de Vries. 2 ♀ (RMNH, IEBR), id., but Dong trail, 9–30.iv.2007, M.P. Quy & N.T. Manh, RMNH’07”.

##### Diagnosis.

The new species is close to Therophilus annulus (Chou & Sharkey, 1989) comb. n., from China (Taiwan) but differs by having the pronotum smooth (Therophilus annulus: sparsely minutely punctate along margins and trough with 3 carinae), the propodeum with a large areola-like median area with irregular transverse carinae (annulus: rugose and with 2 median carinae), the ovipositor sheath 0.6 times as long as the fore wing (annulus: 1.3 times) and the first tergite 2.2 times as long as wide apically (annulus: 1.5–1.6 times).

##### Description.

Holotype, ♀, length of body 5.5 mm, of fore wing 5.3 mm, ovipositor sheath 3.1 mm.

###### Head.

Antennal segments 38, length of third segment 1.8 times fourth segment, length of third, fourth and penultimate segments 4.0, 2.2 and 1.3 times their width, respectively; apical antennal segment 1.5 times as long as penultimate segment; length of maxillary palp 0.8 times height of head; malar space 1.9 times as long as basal width of mandible; temple short, in dorsal view length of eye 3.0 times temple; temple roundly narrowed posteriorly (Fig. 325); ocelli in low triangle, POL:OD:OOL = 10:6:13; face shiny, distinctly and finely punctate; frons with medial ridge, smooth; vertex and temple shiny, largely smooth, but sparsely punctulate.

###### Mesosoma.

Length of mesosoma 1.4 times its height; pronotum smooth with two carinae anteriorly and fine punctures dorsally; area near lateral carina of mesoscutum crenulate; latelal and middle lobes of mesoscutum with close distinct punctures anteriorly, medio-posteriorly slightly convex and smooth; notauli complete crenulate, narrower anteriorly and wider posteriorly (Fig. 322); scutellar sulcus 0.7 times as long as dorsal part of scutellum and with 3 carinae; scutellum distinctly narrowed posteriorly, shiny with sparse fine punctures, subposterior crest short and curved (Fig. 322); precoxal sulcus deep, strongly crenulate (Fig. 321); mesopleuron below precoxal sulcus with dense distinct punctures; mesopleuron above precoxal sulcus shiny and largely smooth, sparsely finely punctate posteriorly; metapleuron densely setose, rugose-punctate; propodeum with large areola-like area with irregular transverse carinae (Fig. 322); propodeal spiracle rather large, 1.7 times as long as wide

###### Wings.

**Figure 320.**
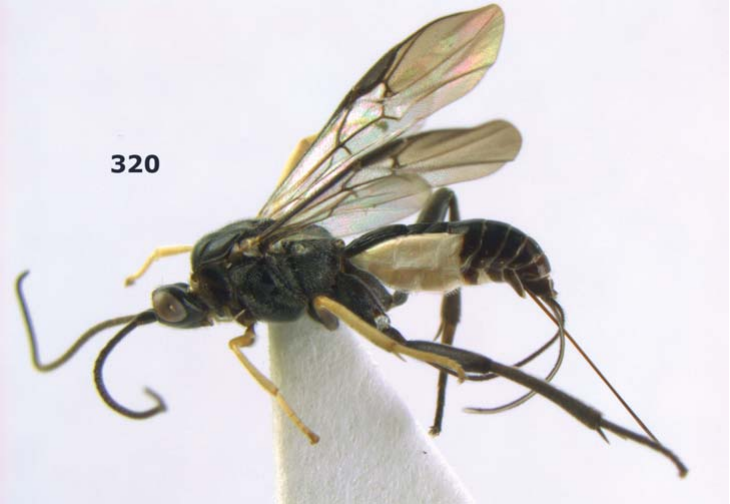
Therophilus nigrolineatus sp. n., female, holotype. Habitus lateral.

Fore wing: second submarginal cell small and petiolate (Fig. 320); vein SR1 sinuate; r:3-SR+SR1 = 1:33. Hind wing: vein M+CU 0.7 times as long as vein 1-M.

###### Legs.

Length of hind femur, tibia and basitarsus 3.6, 7.5 and 10.0 times their width, respectively; hind femur (as remainder of legs) with short setae (Fig. 320); length of outer and inner spur of middle tibia 0.5 and 0.7 times middle basitarsus, respectively; outer side of middle tibia with a row of 5 pegs and 2 pegs at apex; length of outer and inner spur of hind tibia 0.4 and 0.5 times hind basitarsus, respectively; tarsal claws with lobe.

###### Metasoma.

First tergite parallel-sided without dorsal carinae, its length 2.2 times its apical width (Fig. 323); first and second tergites coarsely striate; second tergite with transverse curved groove (Fig. 323); remainder of metasoma smooth; ovipositor sheath 0.6 times as long as fore wing.

###### Colour.

Black; antenna dark brown but paler apically; fore leg (but coxa brown), middle tibia and tarsus yellow; hind leg black or blackish-brown; wing membrane infuscate apically and subhyaline basally (Figs 320, 328); first and second tergites white laterally.

###### Variation.

Antennal segments of females 39–40, of male 36; vein M+CU 0.8 times as long as vein 1-M; scutellum rugose-punctate or sparsely punctate; outer side of middle tibia with row of 2–3 pegs; length of body 4.0–5.5 mm, of fore wing 3.9–5.3 mm.

##### Distribution.

S Vietnam: Dong Nai.

**Figures 321–328.**
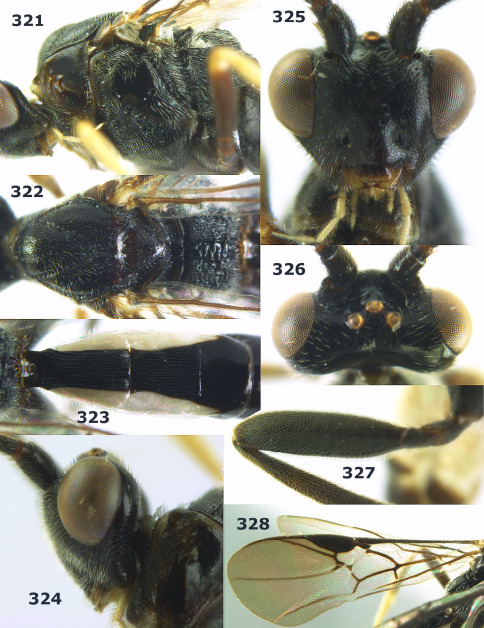
Therophilus nigrolineatus sp. n., female, holotype. **321** mesosoma lateral **322** mesosoma dorsal **323** first-third metasomal tergites dorsal **324** head lateral **325** head anterior **326** head dorsal **327** hind femur lateral **328** wings.

##### Biology.

Unknown.

##### Etymology.

From “nigro” (Latin for “black”), and “linea” (Latin for “line”), because of the black linear middle part of the first and second metasomal tergites.

#### Therophilus nuichuaensis sp. n.

urn:lsid:zoobank.org:act:62D30014-5267-43BA-9E4C-4D5DAF86B4FD

Figs 329 –337 

##### Type material.

Holotype, ♀ (RMNH), “Vietnam: Ninh Thuân, Núi Chúa N.P., dry south part, Mal. traps, 100–180 m, 22–29.v.2007, C. v. Achterberg & R. de Vries, RMNH’07”. Paratypes: 1 ♀ + 4 ♂ (IEBR, RMNH), same data.

##### Diagnosis.

Runs in the key by Chen & Yang (2006) to Therophilus tayulingensis (Chou & Sharkey, 1989) from China (Taiwan), but that species has only the apical fifth of the hind tibia dark brown, vein SR1 of the fore wing curved and resulting in a very narrow marginal cell anteriorly, vein cu-a of the fore wing oblique, the posterior ocelli comparatively large, larger than the anterior ocellus, the scutellar sulcus with three carinae, the first tergite costate and the second tergite distinctly transverse. It runs in Chou & Sharkey (1989) because of the nearly square second tergite to Therophilus festivus (Muesebeck, 1953), but that species has the scutellar sulcus with three carinae, the first tergite costate, the second tergite rather costate to nearly smooth, the median area of the second tergite weakly differentiated and the first tergite less robust, 1.6–1.9 times as long as wide apically.

##### Description.

Holotype, ♀, length of body 3.2 mm, of fore wing 2.5 mm, ovipositor sheath 2.2 mm.

###### Head.

Antenna incomplete, with 21 segments remaining, length of third segment 1.3 times fourth segment, length of third and fourth segments 3.0 and 2.4 times their width, respectively; length of maxillary palp 0.6 times height of head; malar space 2.4 times as long as basal width of mandible; in dorsal view length of eye 3.2 times temple; temple gradually narrowed posteriorly (Fig. 336); ocelli in moderately high triangle, POL:OD:OOL = 9:5:10; face moderately shiny and densely punctulate; clypeus largely smooth and weakly convex, punctulate; frons with sharp and distinct medial ridge, shallowly concave anteriorly, smooth and rather convex medially and moderately punctulate laterally; area in font of anterior ocellus short and with a short subparallel depression; vertex and temple shiny and largely smooth, with sparsely punctulate.

###### Mesosoma.

Length of mesosoma 1.3 times its height; pronotum with distinct convex area antero-ventrally, with seven carinae or rugae antero-medially, finely densely punctate dorso-posteriorly and posterior groove distinctly crenulate, remainder mainly smooth; area near lateral carina of mesoscutum crenulate; mesoscutum spaced punctulate, middle lobe with a pair of shallow longitudinal depressions, medio-posteriorly lobes slightly convex; notauli complete and narrowly crenulate; scutellar sulcus half as long as dorsal face of scutellum, moderately deep and with one carina; scutellum shiny, rather flattened and with sparse rather coarse punctures, subposterior crest obsolescent, but with a narrow crenulate transverse groove (Fig. 331), medio-posterior depression obsolescent; precoxal sulcus deep, moderately and narrowly crenulate, but anteriorly absent (Fig. 330); mesopleuron below precoxal sulcus spaced punctulate and superficially coriaceous; remainder of mesopleuron spaced finely punctate and partly superficially coriaceous; metapleuron densely setose, spaced moderately punctate and ventrally rugose; propodeum coarsely rugose-reticulate, but smooth posteriorly (Fig. 331); propodeal spiracle small and round.

###### Wings.

Fore wing: second submarginal cell small and petiolate (Fig. 333); vein SR1 straight; r:3-SR+SR1 = 1:26; r-m about slightly longer than petiolus (Fig. 333); apical half of subbasal cell moderately setose; 2-R1 about as long as 1-R1 (Fig. 333). Hind wing: vein M+CU as long as vein 1-M.

###### Legs.

Length of hind femur, tibia and basitarsus 3.6, 6.6 and 7.6 times their width, respectively; hind femur largely smooth, finely pimply and with short setae (Fig. 334); length of outer and inner spur of middle tibia 0.5 and 0.7 times middle basitarsus, respectively; outer side of middle tibia with a row of 3 pegs and 2 pegs at apex; length of outer and inner spurs of hind tibia 0.4 and 0.5 times hind basitarsus, respectively; tarsal claws with large lobe.

###### Metasoma.

First tergite mainly superficially granulate intermingled with rugulae, parallel-sided without distinct dorsal carinae, its length 1.4 times its apical width (Fig. 332); second and third tergites smooth, second tergite slightly narrowed anteriorly and with an obsolescent curved transverse groove (Fig. 332); remainder of metasoma (including second suture) smooth; ovipositor sheath 0.90 times as long as fore wing.

###### Colour.

Black; pedicellus brown and distinctly paler than scapus and flagellar segments mainly brown with dark medial band or largely dark brown; mandible, palpi, malar space ventrally, tegulae, basal 0.6 of hind tibia (but with an indistinct subbasal brown patch), largely hind trochanter and trochantellus, apex and base of first tergite narrowly, second tergite, third tergite laterally and ventral half of metasoma ivory or pale yellowish; pronotum antero-medially, fore leg (but coxa largely dark brown), middle leg (but coxa dark brown and tarsus brown), scutellum laterally, metanotum, remainder of metasoma, pterostigma and veins dark brown; wing membrane subhyaline.

###### Variation.

Antennal segments of female 30, of male 29–32; vein M+CU 0.7–1.1 times as long as vein 1-M; outer side of middle tibia with row of 3–6 pegs; apex of first tergite narrowly (♀) or widely (♂) ivory; posterior half of second tergite dark brown (♀) or ivory (♂);length of body 3.2–3.9 mm, of fore wing 2.5–3.3 mm.

##### Distribution.

S Vietnam: Ninh Thuân.

**Figure 329.**
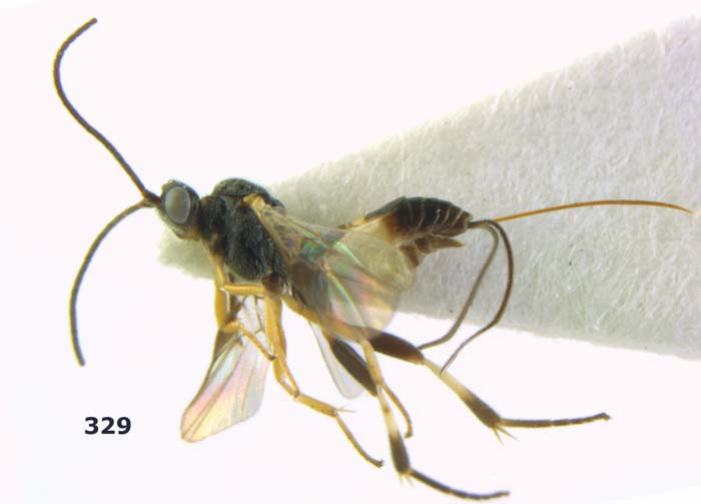
Therophilus nuichuaensis sp. n., female, holotype. Habitus lateral.

**Figures 330–337.**
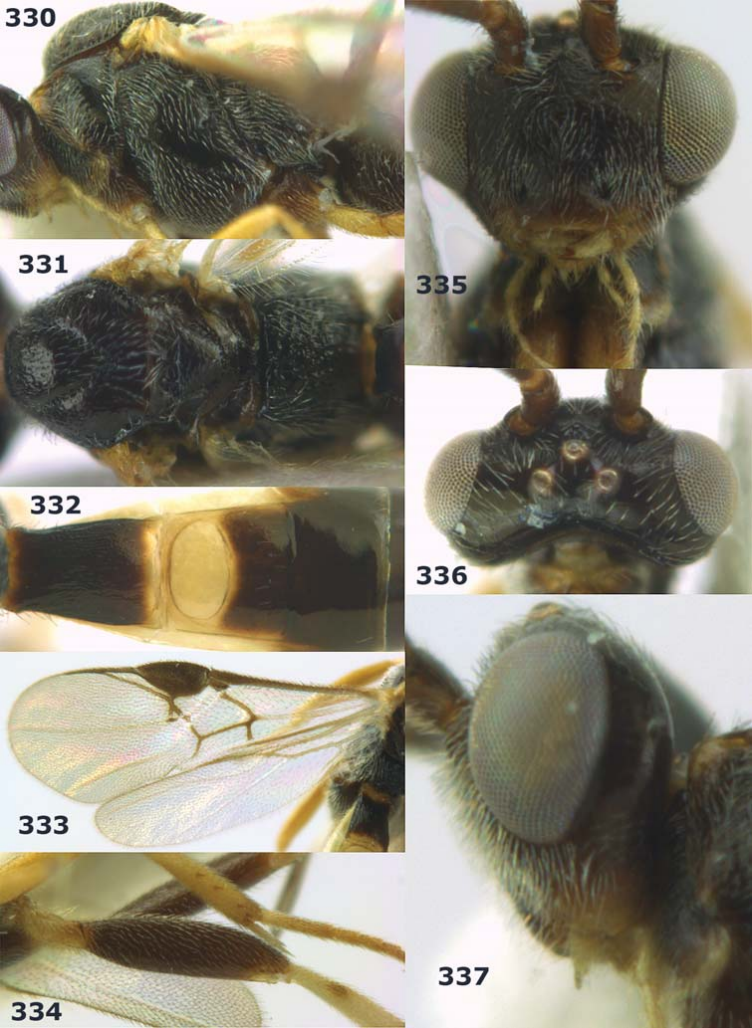
Therophilus nuichuaensis sp. n., female, holotype. **330** mesosoma lateral **331** mesosoma dorsal **332** first-third metasomal tergites dorsal **333** wings **334** hind femur lateral **335** head anterior **336** head dorsal **337** head lateral.

##### Biology.

Unknown.

##### Etymology.

Named after the type locality: Núi Chúa National Park.

#### Therophilus parasper sp. n.

urn:lsid:zoobank.org:act:E8CE7AB5-4FDB-4196-B216-BA959B8B2D6F

Figs 338 –346 

##### Type material.

Holotype, ♀ (RMNH), “N.W. Vietnam: Tonkin, Hoang Lien N.P., 10 km SW Sa Pa, c. 1550 m, 22–29.x.1999, Malaise traps, C. v. Achterberg, RMNH’99”.

##### Diagnosis.

The new species is similar to Therophilus asper (Chou & Sharkey, 1989) comb. n., but differs by having the hind femur 5 times as long as wide (Therophilus asper: 4 times); the propodeum with a pentagonal areola (asper: without distinct areola); the first tergite 1.7 times as long as its apical width (asper: 1.3 times ) ; the second tergite striate (asper: almost completely smooth) ; the outer side of the middle tibia with 3 pegs (asper: with 7 pegs) and hind tibia with a yellowish basal ring (asper: without pale basal ring).

##### Description.

Holotype, ♀, length of body 6.6 mm, of fore wing 6.3 mm, of ovipositor sheath 5.0 mm.

###### Head.

Antennal segments 37, length of third segment 1.2 times fourth segment, length of third, fourth and penultimate segments 3.1, 3.0 and 2.0 times their width, respectively; length of apical antennal segment 1.2 times as long as penultimate segment; maxillary palp 0.8 times height of head; malar space 2.5 times as long as basal width of mandible; in dorsal view length of eye 3.4 times temple; temples gradually narrowed (Fig. 345); POL:OD:OOL = 6:4:10; face distinctly punctate with short medial groove and rugose-punctate medially; frons coriaceous with fine faint rugae laterally; vertex and temple smooth with very sparse minute punctures.

###### Mesosoma.

Length of mesosoma 1.6 times its height; pronotum shiny smooth medially, crenulate anteriorly, pronotal trough rugose-punctate, dorsally with dense punctures; area near lateral carina of mesoscutum densely crenulate; mesoscutum rugose-punctate, slightly flat posterior with sparse punctures; notauli complete, crenulate; scutellar sulcus 0.6 times as long as dorsal part of scutellum and with one carinae; scutellum largely rugose-punctate with transverse subposterior crest (Fig. 340); precoxal sulcus crenulate, extending 0.9 of mesopleuron (Fig. 339); mesopleuron below precoxal sulcus with sparse fine punctures; mesopleuron above precoxal sulcus shiny with very sparse minute punctures; metapleuron densely setose, rugose-punctate; propodeum with pentagonal areola basally with transverse rugae medio-apically; propodeal spiracle small, as long as wide.

###### Wings.

Fore wing: second submarginal cell medium-sized (Fig. 342); vein SR1 distinctly curved; r:3-SR+SR1 = 3:73. Hind wing: vein M+CU 0.8 as long as vein 1-M (25:30).

###### Legs.

Length of hind femur, tibia and basitarsus 5.0, 8.1 and 10.7 times their width, respectively; hind femur (as remainder of legs) with short setae (Fig. 343); length of outer and inner spur of middle tibia 0.4 and 0.7 times middle basitarsus, respectively; outer side of middle tibia with 3 pegs; length of outer and inner spur of hind tibia 0.3 and 0.5 times hind basitarsus, respectively; tarsal claws with lobe.

###### Metasoma.

First tergite with short medial carina, slightly depressed laterally, its length 1.7 times its apical width (Fig. 341); first tergite sparsely striate medially, densely rugose-striate apically; second tergite with transverse groove, striate with medial striae convergent (Fig. 341); ovipositor sheath 0.8 times as long as fore wing.

###### Colour.

Black; palpi, fore legs, middle tibia and tarsus yellow; middle femur yellowish brown; hind tibia with a pale yellowish basal ring (Fig. 343); first-second tergites whitish ventrally; pterostigma dark brown; wing membrane infuscate apically and subhyaline basally (Fig. 342).

##### Distribution.

NW Vietnam: Lao Cai.

**Figure 338.**
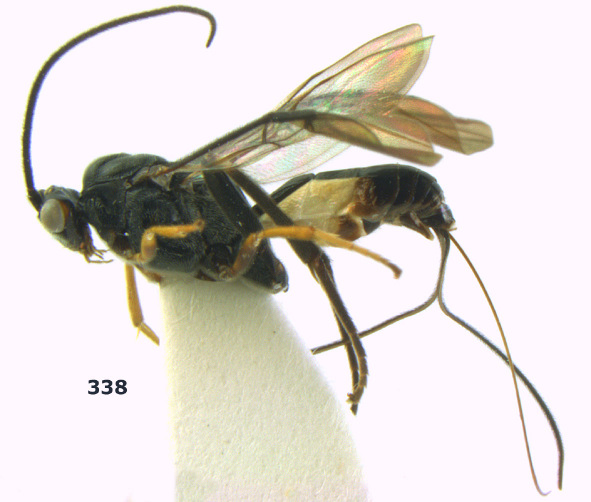
Therophilus parasper sp. n., female, holotype. Habitus lateral.

**Figures 339–346.**
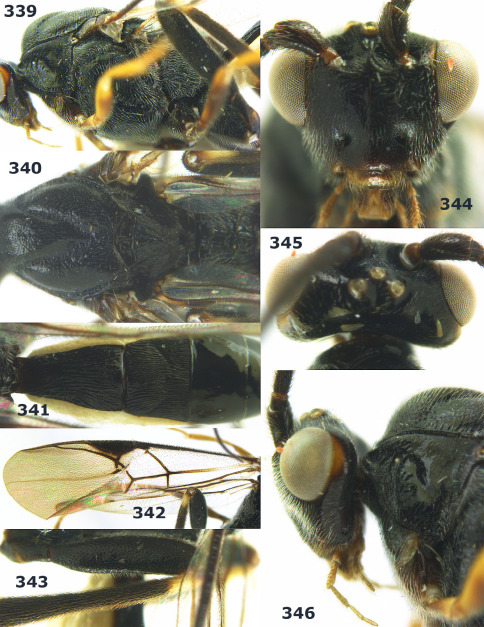
Therophilus parasper sp. n., female, holotype. **339** mesosoma lateral **340** mesosoma dorsal **341** first-third metasomal tergites dorsal **342** wings **343** hind femur lateral **344** head anterior **345** head dorsal **346** head lateral.

##### Biology.

Unknown.

##### Etymology.

From “para” (Greek for “near”), and the specific name “asper”, because of its similarity to that species.

#### Therophilus planifrons sp. n.

urn:lsid:zoobank.org:act:AAFD7DFA-8057-4755-9790-6BC6F6E0FFCF

Figs 347 –355 

##### Type material.

Holotype, ♀ (RMNH), “NW. Vietnam: Tonkin, Hoang Lien N.P., 15 km SW Sa Pa, c 1900 m, 15–21.x.1999, Malaise traps, C. v. Achterberg, RMNH’99”.

##### Diagnosis.

Differs from other known species by the combination of the flattened frons, the less transverse head, the comparatively short ovipositor sheath (about 0.5 times fore wing), the lack of a triangular area in front of the anterior ocellus and the tegulum paler than the humeral plate.

##### Description.

Holotype, ♀, length of body 3.4 mm, of fore wing 3.0 mm, ovipositor sheath 1.3 mm.

###### Head.

Antenna with 28 segments, length of third segment 1.2 times fourth segment, length of third, fourth and penultimate segments 3.7, 3.0 and 2.0 times their width, respectively; length of maxillary palp 0.7 times height of head; malar space 1.8 times as long as basal width of mandible; in dorsal view length of eye 4.0 times temple; temple roundly narrowed posteriorly (Fig. 354); ocelli in low triangle, POL:OD:OOL = 9:5:11; face shiny and distinctly punctulate; clypeus smooth medially, remainder finely punctate and rather weakly convex; frons flattened posteriorly, without a medial ridge or triangular area, but with a short groove medio-anteriorly, smooth medially and distinctly punctulate laterally; vertex and temple shiny and largely smooth, with sparse punctulation.

###### Mesosoma.

Length of mesosoma 1.7 times its height; pronotum smooth, with distinct epomial and deep subpronope anteriorly, finely sparsely punctate dorso-posteriorly and posterior groove indistinctly crenulate ventrally; area near lateral carina of mesoscutum crenulate; mesoscutum spaced punctulate but finely punctate near notauli, medio-posteriorly lobes flattened; notauli complete and narrowly crenulate anteriorly and becoming wider posteriorly, coalescent part rather widely crenulate; scutellar sulcus half as long as dorsal face of scutellum, shallow and with one short carina; scutellum shiny and with a few punctures, subposterior crest obsolescent but with distinctly crenulate groove and medio-posteriorly with semi-circular crenulate depression (Fig. 349); precoxal sulcus rather shallow, weakly and narrowly crenulate and anteriorly absent (Fig. 348); remainder of mesopleuron spaced punctulate; metapleuron rather densely setose, spaced finely punctate dorsally and antero-ventrally rugose; propodeum coarsely areolate dorsally and rather finely reticulate posteriorly, with a coarse transverse carina, a triangular areola and a short median carina dorsally, partly smooth anteriorly (Fig. 349); propodeal spiracle rather small, round.

###### Wings.

Fore wing: second submarginal cell medium-sized and petiolate (Fig. 351); vein SR1 distinctly bent towards pterostigma and close to it (Fig. 351); pterostigma wide and vein r short, r:3-SR+SR1 = 1:21; r-m slightly longer than petiolus (Fig. 351); apical half of subbasal cell densely setose. Hind wing: vein M+CU as long as vein 1-M.

###### Legs.

Length of hind femur, tibia and basitarsus 2.9, 6.1 and 7.4 times their width, respectively; hind femur shiny, largely smooth and with rather short setae (Fig. 352); length of outer and inner spur of middle tibia 0.35 and 0.45 times middle basitarsus, respectively; outer side of middle tibia with a row of 3 pegs and 2 pegs at apex; length of outer and inner spurs of hind tibia 0.35 and 0.50 times hind basitarsus, respectively; tarsal claws with large lobe.

###### Metasoma.

First tergite widened posteriorly, largely smooth, with a few striae laterally, its basal half with weak dorsal carinae, its length 1.4 times its apical width (Fig. 350); second and following tergites (including second suture) smooth; second tergite somewhat narrowed anteriorly and with a curved transverse groove (Fig. 350); ovipositor sheath 0.42 times as long as fore wing.

###### Colour.

Black; antenna, humeral plate, fore coxa basally, fore and middle tarsi largely, middle coxa largely, hind femur, apical third of hind tibia, hind tarsus (except whitish basal third of basitarsus), metasoma apically and ventrally (but antero-ventrally largely pale yellowish), ovipositor sheath, veins and pterostigma dark brown; palpi and mandible pale yellowish; remainder of fore and middle legs, hind trochanter and trochantellus, subbasal patch of hind tibia and tegulum brownish-yellow; spurs and remainder of hind tibia whitish; wing membrane slightly infuscate (Fig. 351).

##### Distribution.

NW Vietnam: Lao Cai.

**Figure 347.**
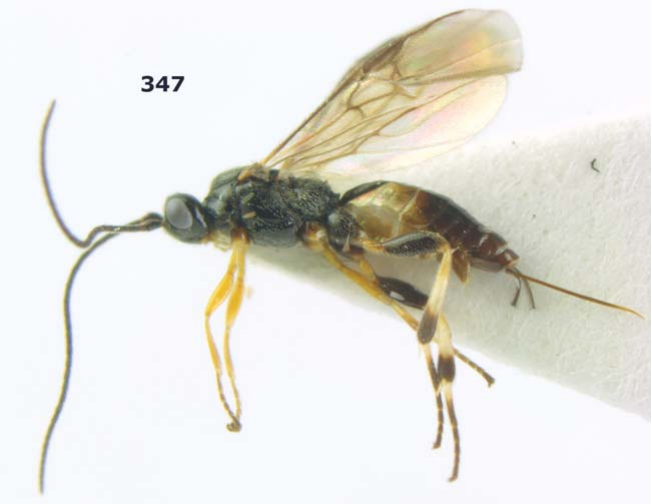
Therophilus planifrons sp. n., female, holotype. Habitus lateral.

**Figures 348–355.**
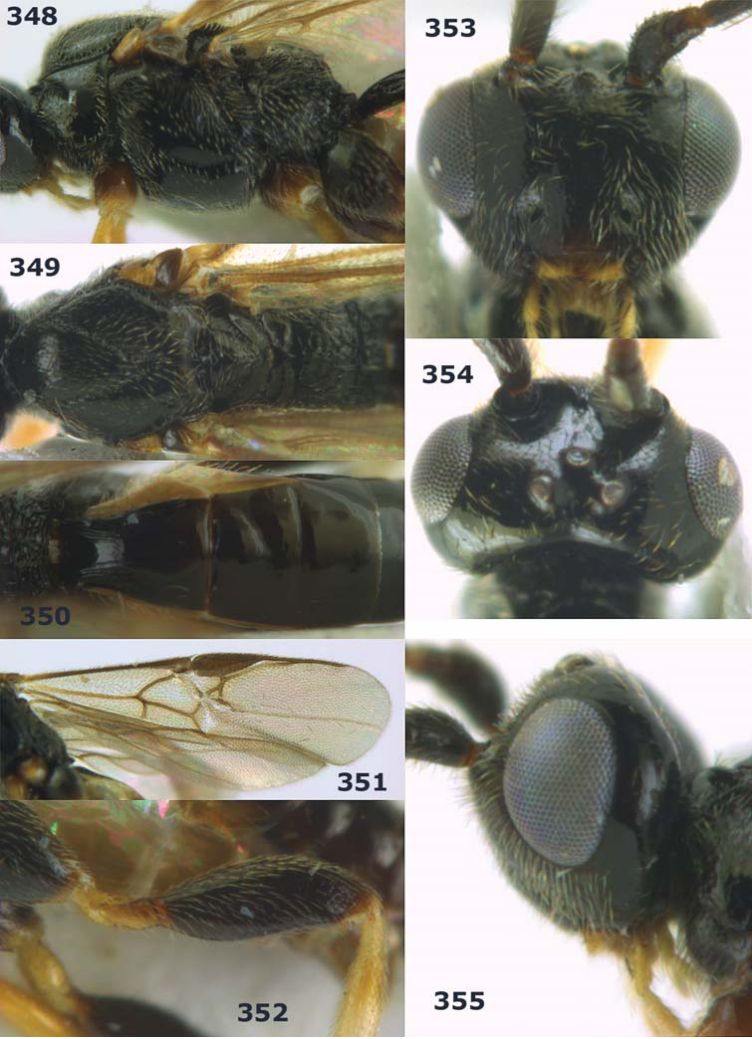
Therophilus planifrons sp. n., female, holotype. **348** mesosoma lateral **349** mesosoma dorsal **350** first-third metasomal tergites dorsal **351** wings **352** hind femur lateral **353** head anterior **354** head dorsal **355** head lateral.

##### Biology.

Unknown.

##### Etymology.

From “planus” (Latin for “flat”), and “frons” (Latin for “fore part”), because of the flat frons.

#### Therophilus punctiscutum sp. n.

urn:lsid:zoobank.org:act:E1471131-90B7-485B-815A-A089593A9FB0

Figs 356 –364 

##### Type material.

Holotype, ♀ (RMNH), “N. Vietnam: Hoa Binh, Pa Co Hang Kia N.R., 1321 m, 20°44'36N; 104°53'44E, 10–24.x.2009, Mal. tr. 8, C. v. Achterberg & R. de Vries, RMNH’09”.

##### Diagnosis.

Recognizable by its slender basal tergites of the metasoma and the slender mesosoma (including a slender mesopleuron), combined with a black head and mesosoma.

##### Description.

Holotype, ♀, length of body 4.9 mm, of fore wing 3.7 mm, ovipositor sheath 4.6 mm.

###### Head.

Antenna with 32 segments, length of third segment 1.4 times fourth segment, length of third, fourth and penultimate segments 4.2, 3.0 and 1.5 times their width, respectively; length of maxillary palp 0.7 times height of head; malar space 2.2 times as long as basal width of mandible; in dorsal view length of eye 3.8 times temple; temple gradually narrowed posteriorly (Fig. 363); ocelli in rather high triangle, POL:OD:OOL = 6:5:8; face rather shiny and distinctly rather densely and finely punctate; clypeus sparsely finely punctate and moderately convex; frons flat and finely punctate between antennal sockets and without a medial ridge, no triangular area posteriorly, but in front of anterior ocellus with a narrow groove, smooth medially and rather densely finely punctate laterally; vertex and temple shiny and largely smooth, with sparse punctulation.

###### Mesosoma.

Length of mesosoma 1.7 times its height; pronotum smooth with three weak carinae and double distinct epomial anteriorly, subpronope rather shallow, finely densely punctate and densely setose dorso-posteriorly and posterior groove narrowly crenulate; area near lateral carina of mesoscutum crenulate; mesoscutum moderately coarsely punctate, with distinct smooth interspaces, medio-posteriorly lobes slightly convex; notauli complete and narrowly crenulate; scutellar sulcus 0.4 times as long as dorsal face of scutellum, moderately deep and with one carina; scutellum flat, shiny and with sparse rather coarse punctures, subposterior crest weakly protruding and in front shallowly crenulate (Fig. 358), medio-posteriorly rather steep in front of protruding medial area of pronotum; precoxal sulcus narrow, rather deep, largely smooth and anteriorly absent (Fig. 357); mesopleuron below precoxal sulcus; remainder of mesopleuron shiny and spaced finely punctate, scrobe round and deep, isolated from pleural sulcus; metapleuron densely setose, rather dense but superficially punctate and ventrally rugose; propodeum rather coarsely densely vermiculate-reticulate (Fig. 358); propodeal spiracle medium-sized, 1.4 times as long as wide.

###### Wings.

Fore wing: second submarginal cell medium-sized and petiolate (Fig. 360); vein SR1 straight; r:3-SR+SR1 = 1:17; r-m about half as long as petiolus; apical half of subbasal cell largely sparsely setose. Hind wing: vein M+CU 0.8 times as long as vein 1-M (Fig. 360); vein 2-CU distinct.

###### Legs.

Length of hind femur, tibia and basitarsus 4.3, 8.2 and 9.2 times their width, respectively; hind femur spaced finely punctate and with short setae (Fig. 361); length of outer and inner spur of middle tibia 0.35 and 0.50 times middle basitarsus, respectively; outer side of middle tibia with a row of 3 pegs and 2 pegs at apex; length of outer and inner spurs of hind tibia 0.3 and 0.4 times hind basitarsus, respectively; tarsal claws with large obtuse lobe.

###### Metasoma.

First tergite parallel-sided, rather irregularly and densely longitudinally striate and 2.5 times as long as its apical width, with rather weak dorsal carinae in basal 0.6 of tergite (Fig. 359); second tergite irregularly longitudinally rugulose-striate, but basal 0.4 with nearly round convexity superficially micro-sculptured; second tergite somewhat narrowed anteriorly and with obsolete curved transverse groove (Fig. 359); remainder of metasoma (including second suture) smooth; ovipositor sheath 1.24 times as long as fore wing.

###### Colour.

Black; antenna, tegulae, fore and middle coxae, apical half of hind tibia and small subbasal patch, hind tarsus, metasoma apically and ventrally (but antero-ventrally largely ivory), ovipositor sheath, hind spurs, veins and pterostigma dark brown; malar space, palpi, mandible, fore trochanter, basal half of middle tibia (except brownish patch subbasally), middle spurs and middle basitarsus largely, remainder of hind tibia, basal third of second tergite and its lateral margin ivory or white; middle trochanter, base of middle femur, apical half of middle tibia and telotarsus brown; remainder of fore and middle legs brownish-yellow; and remainder of hind tibia whitish; wing membrane subhyaline.

##### Distribution.

NE Vietnam: Hoa Binh.

**Figure 356.**
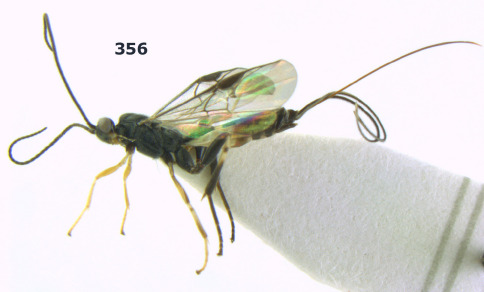
Therophilus punctiscutum sp. n., female, holotype. Habitus lateral.

**Figures 357–364.**
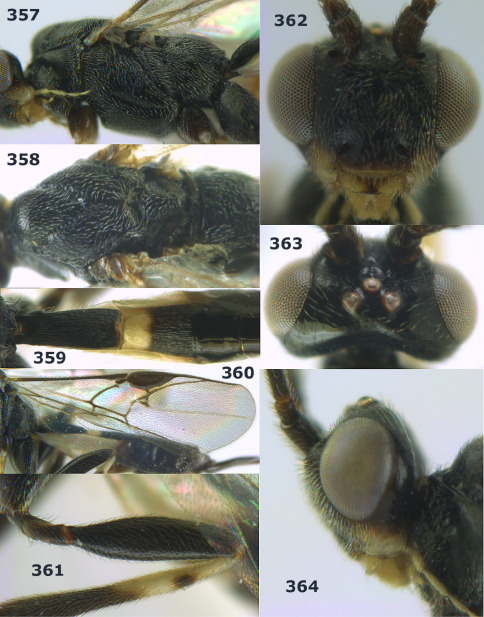
Therophilus punctiscutum sp. n., female, holotype. **357** mesosoma lateral **358** mesosoma dorsal **359** first-third metasomal tergites dorsal **360** wings **361** hind femur lateral **362** head anterior **363** head dorsal **364** head lateral.

##### Biology.

Unknown.

##### Etymology.

From “punctum” (Latin for “small hole, spot”), and “scutum” (Latin for “shield”), because of the punctate mesoscutum.

#### Therophilus robustus sp. n.

urn:lsid:zoobank.org:act:0BC020B8-F33D-4628-AA4C-223B735A76AB

Figs 365 –373 

##### Type material.

Holotype, ♂ (RMNH), “N. Vietnam: Ha Noi, Gia Lam, soya bean field, ex O[miodes] indicata, 24.iii.1995, Ha”, “Aga 004”.

##### Diagnosis.

Recognizable by the unique feature of the mesoscutum protruding over the pronotum anteriorly; in addition the middle lobe of the mesoscutum is comparatively short and wide.

##### Description.

Holotype, ♂, length of body 5.1 mm, of fore wing 4.2 mm.

###### Head.

Antenna with 35 segments, length of third segment 1.5 times fourth segment, length of third, fourth and penultimate segments 4.4, 3.0 and 1.5 times their width, respectively; length of maxillary palp 0.7 times height of head; malar space 1.6 times as long as basal width of mandible; in dorsal view length of eye 4.8 times temple; temple directly narrowed posteriorly (Fig. 373); ocelli in low triangle, POL:OD:OOL = 6:5:7; face flattened but medio-dorsally widely depressed, shiny and spaced finely punctate; clypeus largely smooth and weakly convex, only dorsally punctulate; frons with a short medial ridge, smooth medially and spaced coarsely punctate laterally, in front of anterior ocellus with a somewhat depressed large elongate triangular area and strongly depressed behind antennal sockets; vertex and temple shiny and largely smooth, with sparse punctures.

###### Mesosoma.

Length of mesosoma 1.3 times its height; pronotum smooth but coarsely crenulate antero-medially and posterior groove distinctly crenulate ventrally; area near lateral carina of mesoscutum finely crenulate; anteriorly mesoscutum porches over pronotum (Fig. 366); mesoscutum robust, very sparsely finely punctate, but rather densely on middle lobe, medio-posteriorly lobes convex and with deep crenulate groove in between; notauli complete and narrowly crenulate; scutellar sulcus 0.7 times as long as dorsal face of scutellum, rather deep and with 5 long carinae; scutellum shiny, weakly convex and with sparse punctulation, subposterior crest distinct and finely crenulate (Fig. 367), medio-posterior depression absent but steep posterior slope rugulose; precoxal sulcus rather deep, distinctly crenulate and anteriorly absent (Fig. 366); remainder of mesopleuron shiny and smooth; mesosternal sulcus largely smooth; metapleuron short setose, superficially punctate but ventrally rugose; propodeum very coarsely reticulate-rugose (Fig. 367); propodeal spiracle rather large (Fig. 367), 1.2 times as long as wide.

###### Wings.

Fore wing: second submarginal cell rather small and petiolate (Fig. 369); vein SR1 straight; r:3-SR+SR1 = 1:13; r-m about half as long as petiolus; apical half of subbasal cell nearly completely glabrous. Hind wing: vein M+CU as long as vein 1-M.

###### Legs.

Length of hind femur, tibia and basitarsus 3.5, 6.1 and 7 times their width, respectively; hind femur smooth (only slightly pimply) and with medium-sized setae (Fig. 370); length of outer and inner spur of middle tibia 0.5 and 0.7 times middle basitarsus, respectively; outer side of middle tibia with row of 3 pegs and 2 pegs at apex; length of outer and inner spurs of hind tibia 0.35 and 0.55 times hind basitarsus, respectively; tarsal claws with large lobe; hind coxa superficially granulate.

###### Metasoma.

First tergite somewhat widened apically, 1.4 times as long as its apical width, longitudinally costate, with dorsal carinae nearly complete but similar to surrounding sculpture (Fig. 368); second tergite rather costate, subparallel-sided, its basal 0.6 with a semicircular convexity and with a curved transverse groove (Fig. 368); remainder of metasoma (including second suture) smooth.

###### Colour.

Black; pronotum (but ventrally yellowish), mesoscutum, scutellum (but laterally dark brown) and mesopleuron antero-dorsally orange brown, scapus, pedicellus, clypeus ventrally, tegulum, fore and middle legs, propleural flange, hind trochanter and trochantellus, spurs, hind tarsus (but somewhat infuscate) and narrowly apex of metasoma brownish-yellow; humeral plate yellow; remainder of antenna brown to rather dark brown; malar space, metanotum and pterostigma dark brown; palpi, mandible, basal 0.6 of hind tibia (and without dark patch), apex of first tergite, second tergite, third tergite narrowly antero-medially and basal half of metasoma ventrally white or ivory; veins largely brown; wing membrane subhyaline.

##### Distribution.

N Vietnam: Ha Noi.

**Figure 365.**
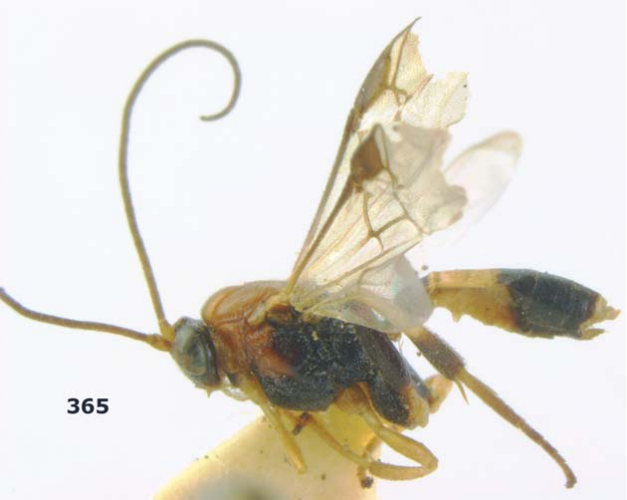
Therophilus robustus sp. n., male, holotype. Habitus lateral.

**Figures 366–373.**
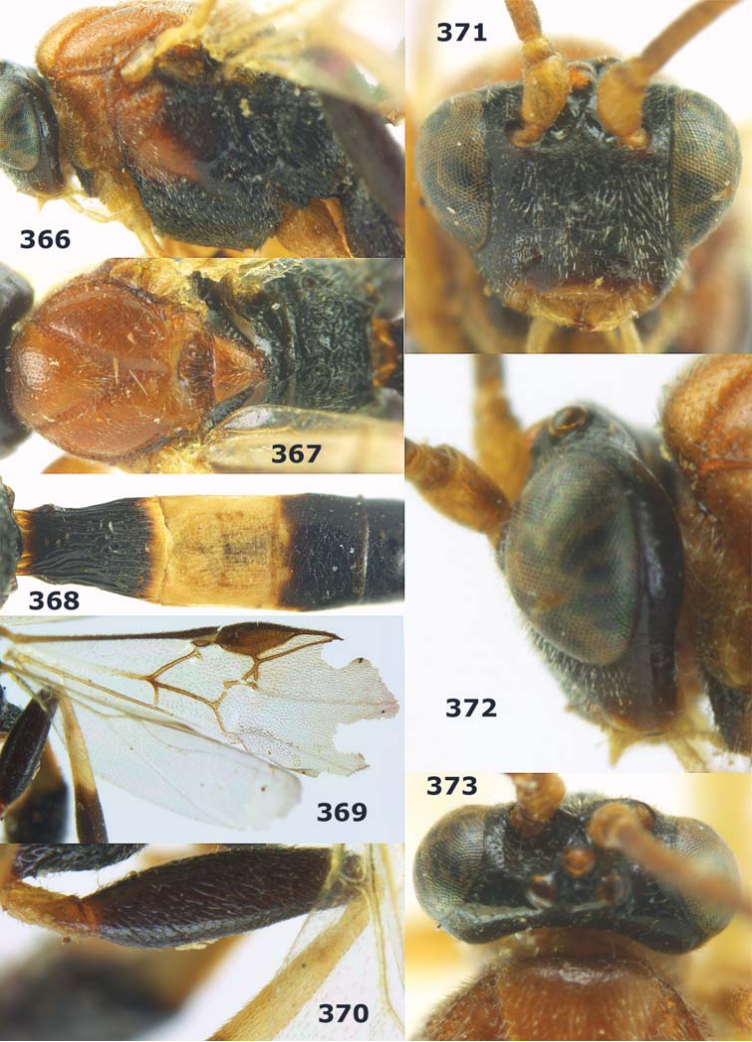
Therophilus robustus sp. n., male, holotype. **366** mesosoma lateral **367** mesosoma dorsal **368** first-third metasomal tergites dorsal **369** wings **370** hind femur lateral **371** head anterior **372** head lateral **373** head dorsal.

##### Biology.

The holotype was reared from Omiodes indicata (Fabricius, 1775) (Lepidoptera: Pyralidae: Pyraustinae) on soybean (Glycine max (Linnaeus)) according to the label data. It is the first record of a species of Agathidinae as parasitoid of this species.

##### Etymology.

From “robustus” (Latin for “strong”), because of the robust body.

#### Therophilus rugosiferus sp. n.

urn:lsid:zoobank.org:act:5379C380-F549-4271-9C35-9C3771989C82

Figs 374 –382 

##### Type material.

Holotype, ♀ (RMNH), “NW. Vietnam: Tonkin, Hoang Lien N.P., 10 km SW Sa Pa, c 1550 m, 22–29.x.1999, Malaise traps, C. v. Achterberg, RMNH’99”. Paratype: 1 ♂ + 1 ♀ (IEBR, RMNH), “NE Vietnam: Hoa Binh, Mai Chau, Pa Co N.P., 1100 m, 21.iv.2001, K.D. Long”.

##### Diagnosis.

Easily recognizable because of the large reticulate-rugose area on the mesoscutum subapically. The new species is similar to the Palaearctic Therophilus cingulipes (Nees, 1812) comb. n. (described as Bassus nantouensis Chou & Sharkey, 1989, from China and synonymized by Sharkey, 1996), but differs by having the pronotal trough rugose (Therophilus cingulipes: weakly crenulate), the notauli widened posteriorly (cingulipes: narrow posteriorly) and the propodeum with a transverse carina dividing the propodeum into two areas (cingulipes: without a distinct transverse carina).

##### Description.

Holotype, ♀, length of body 3.8 mm, of fore wing 3.2 mm.

###### Head.

Antenna broken, length of third segment 1.2 times fourth segment, length of third, fourth and penultimate segments 4.5, 3.5 times their width, respectively; maxillary palp 0.7 times height of head; malar space 3.6 times as long as basal width of mandible; in dorsal view, length of eye 7.0 times temple; temple roundly narrowed posteriorly; ocelli in high triangle (Fig. 381), POL:OD:OOL = 6:5:11; face densely setose with dense distinct punctures; frons with distinct fine punctures; vertex and temple shiny with sparse fine punctures; area near lateral ocelli densely punctate.

###### Mesosoma.

Length of mesosoma 1.5 times its height; pronotum setose and rugose-punctate ventrally and dorsally, smooth medially; area near lateral carina of mesoscutum crenulate; mesoscutum shiny almost coriaceous with very sparse minute punctures anteriorly, slightly flat and smooth posteriorly; notauli complete, crenulate and widened posteriorly, forming a large reticulate-rugose area (Fig. 376); scutellar sulcus 0.4 times as long as dorsal face of scutellum and with 6 carinae; scutellum convex, shiny with very sparse minute punctures, subposterior crest curved (Fig. 376); precoxal sulcus narrow, short and crenulate (Fig. 375); mesopleuron shiny with sparse fine punctures; metapleuron densely setose with distinct punctures anteriorly, rugose posteriorly; propodeum areolate-rugose, with a transverse carina dividing the propodeum into two areas (Fig. 376); propodeal spiracle small, as long as wide

###### Wings.

Fore wing: second submarginal cell small and petiolate (Fig. 378); vein SR1 curved; r:3-SR+SR1 = 3:42. Hind wing: vein M+CU 0.8 times as long as vein 1-M.

###### Legs.

Hind femur robust; length of hind femur, tibia and basitarsus 2.6, 5.0 and 9.5 times their width, respectively; hind femur (as remainder of legs) with short setae (Fig. 379); length of outer and inner spur of middle tibia 0.3 and 0.5 times middle basitarsus, respectively; outer side of middle tibia with 2 pegs and 2 pegs at apex; outer apex of hind tibia with a cluster of 13 pegs; length of outer and inner spur of hind tibia 0.3 and 0.4 times hind basitarsus, respectively; tarsal claws with lobe.

###### Metasoma.

Length of first metasomal tergite 1.4 times its apical width (Fig. 377); first tergite with dense longitudinal striae; second tergite with striate transverse groove, large basal area of second tergite smooth, apical half striate medially and smooth laterally (Fig. 377); remainder of metasoma shiny smooth; length of ovipositor 1.1 times fore wing, its sheath missing.

###### Colour.

Black; clypeus, mouthparts, fore legs (but reddish yellow trochantellus and basal half of tibia); middle legs light brown or infuscate (but yellow femur apically, tibia and hind basitarsus basally); hind spurs, basal two thirds of hind tibia and basal half of hind basitarsus yellowish; pterostigma dark brown; wing membrane slightly infuscate.

###### Variation.

Antenna with 33 segments (female); vein M+CU of hind wing 0.8–0.9 times as long as vein 1-M; POL:OD:OOL = 6:5:8 (male); outer side of middle tibia with 2–3 pegs and 2 pegs at apex; length of body 3.8–4.2 mm, of fore wing 3.0–3.5 mm.

##### Distribution.

NW Vietnam: Lao Cai and NE Vietnam: Hoa Binh.

**Figure 374.**
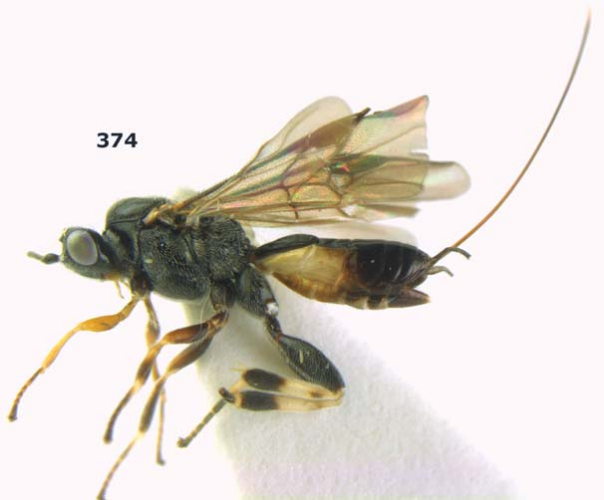
Therophilus rugosiferus sp. n., female, holotype. Habitus lateral.

**Figures 375–382.**
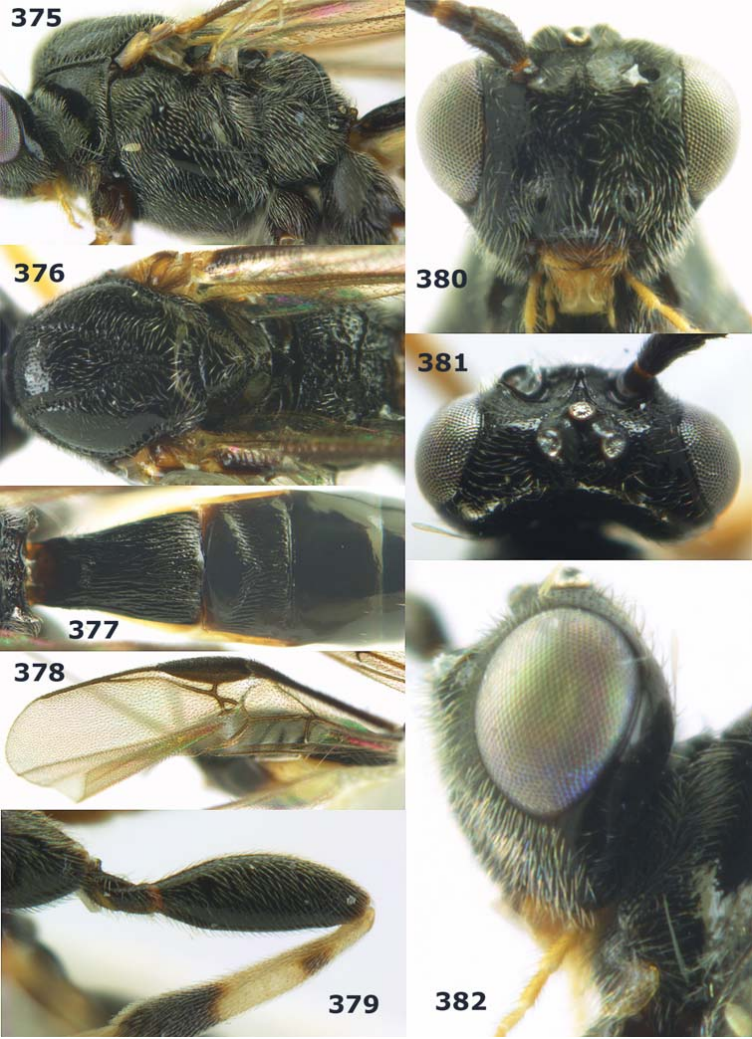
Therophilus rugosiferus sp. n., female, holotype. **375** mesosoma lateral **376** mesosoma dorsal **377** first-third metasomal tergites dorsal **378** fore wing **379** hind femur lateral **380** head anterior **381** head dorsal **382** head lateral.

##### Biology.

Unknown.

##### Etymology.

From “rugosus” (Latin for “wrinkled”), and “fero” (Latin for “carry”), because of the rugose part of the mesoscutum.

#### Therophilus scutellatus sp. n.

urn:lsid:zoobank.org:act:4FF9F64E-6E9D-436C-807B-F68E2E77B1B6

Figs 383 –391 

##### Type material.

Holotype, ♀ (RMNH), “N. Vietnam: Ninh Binh, Cuc Phuong N.P., n[ea]r centre, c. 225 m, 20.xii.1999–10.ii.2000, Mai Phu Quy, RMNH’00”.

##### Diagnosis.

Closely related to Therophilus nigrolineatus sp. n. from South Vietnam, from which it differs mainly by the shape and sculpture of the scutellum (angulate anteriorly and coarsely punctate; Therophilus nigrolineatus: rounded anteriorly and sparsely punctate) and the colour of the hind tibia (black subbasally; Therophilus nigrolineatus: dark brown subbasally).

##### Description.

Holotype, ♀, length of body 5.8 mm, of fore wing 5.5 mm, ovipositor sheath 4.0 mm.

###### Head.

Antenna with 38 segments, length of third segment 1.3 times fourth segment, length of third, fourth and penultimate segments 4.0, 3.2 and 1.5 times their width, respectively; length of maxillary palp 0.7 times height of head; malar space 1.4 times as long as basal width of mandible, rectangularly protruding backwards; in dorsal view length of eye 4.8 times temple; temple gradually narrowed posteriorly; ocelli in rather high triangle (Fig. 390), POL:OD:OOL = 5:5:8; face shiny and rather densely finely punctate; clypeus spaced finally punctate and weakly convex; frons with sharp medial ridge anteriorly and with medium-sized triangular depression in front of anterior ocellus, smooth medially and spaced punctate laterally, behind antennal sockets distinctly depressed; vertex and temple shiny and largely smooth, with sparse fine punctures.

###### Mesosoma.

Length of mesosoma 1.5 times its height; pronotum smooth, with long epomial carina and behind it some striae and a long sinuate oblique carina, with large and deep subpronope, finely densely punctate dorso-posteriorly and ventral half of posterior groove distinctly crenulate, remainder largely smooth and narrow; area near lateral carina of mesoscutum finely crenulate; mesoscutum densely and rather coarsely punctate, but sparsely medio-posteriorly, medio-posteriorly lobes slightly convex, with some weak striae near elongate depression at end of notauli; notauli complete and moderately crenulate anteriorly and more widely so posteriorly; scutellar sulcus 0.6 times as long as dorsal face of scutellum, shallow and with 1 long and 4 short carinae; scutellum angulate anteriorly and with several coarse punctures, shiny, subposterior crest obsolescent and finely crenulate in front of it (Fig. 385) and behind it a strongly shiny, flat, oblique and largely smooth area, without medio-posterior depression; area behind prepectal carina and pleural sulcus coarsely crenulate; precoxal sulcus rather deep, distinctly but narrowly crenulate and anteriorly absent (Fig. 384); mesopleuron below precoxal sulcus spaced finely punctate (ventrally becoming denser); remainder of mesopleuron shiny and largely smooth, but rather coarsely spaced punctate anteriorly and below scrobe; metapleuron densely setose, spaced coarsely punctate (interspaces about equal to diameter of punctures) and ventrally vermiculate-rugose; propodeum coarsely reticulate, with irregular median crest anteriorly (Fig. 385); propodeal spiracle rather large, nearly round.

###### Wings.

Fore wing: second submarginal cell rather small and petiolate, petiolus widened (Fig. 387); vein SR1 nearly straight; r:3-SR+SR1 = 1:17; r-m about 1.5 times as long as petiolus; apical half of subbasal cell glabrous but apical fifth sparsely setose. Hind wing: vein M+CU 0.8 times as long as vein 1-M.

###### Legs.

Length of hind femur, tibia and basitarsus 4.4, 7.8 and 11.4 times their width, respectively; hind femur densely and rather coarsely punctate but with narrow smooth interspaces and with rather short setae; length of outer and inner spur of middle tibia 0.4 and 0.7 times middle basitarsus, respectively; outer side of middle tibia with a row of 2 pegs and 1 peg at apex; length of outer and inner spurs of hind tibia 0.3 and 0.5 times hind basitarsus, respectively; tarsal claws with large obtuse lobe.

###### Metasoma.

First tergite longitudinally costate, distinctly narrowed behind spiracles without distinct dorsal carinae, its length 2.8 times its apical width (Fig. 386); second tergite distinctly narrowed anteriorly, antero-laterally distinctly depressed and remainder longitudinally costate (but somewhat curved anteriorly), without curved transverse groove (Fig. 386); remainder of metasoma (including second suture) smooth; ovipositor sheath 0.73 times as long as fore wing.

###### Colour.

Black; antenna blackish-brown; mesoscutum slightly chestnut-brown posteriorly; palpi, mandible apically (remainder rather dark brown), first and second tergites laterally, third tergite antero-laterally, middle spurs and basal half of metasoma ventrally white or ivory; fore leg (but coxa, trochanter, trochantellus, femur largely) and middle tarsus pale yellow; tegulae, middle trochanter, trochantellus and femur, apical half of metasoma ventrally, ovipositor sheath, veins and pterostigma dark brown; hind leg entirely black or blackish-brown, spurs brown; wing membrane rather infuscate (Fig. 387).

##### Distribution.

N Vietnam: Ninh Binh.

**Figure 383.**
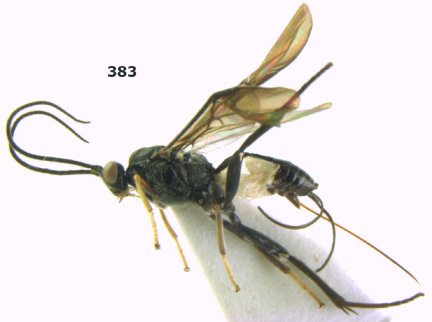
Therophilus scutellatus sp. n., female, holotype. Habitus lateral.

**Figures 384–391.**
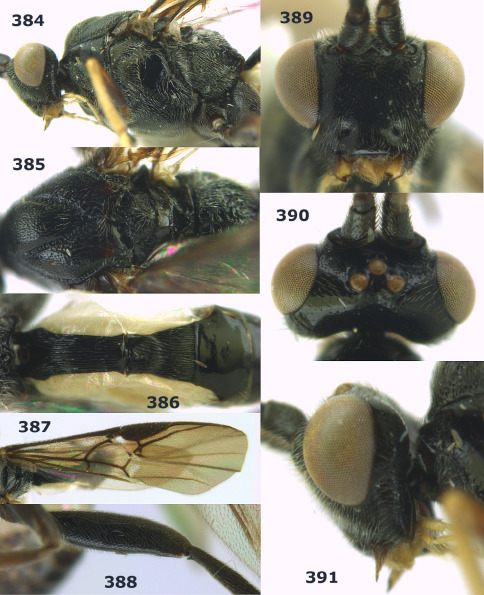
Therophilus scutellatus sp. n., female, holotype. **384** mesosoma lateral **385** mesosoma dorsal **386** first-third metasomal tergites dorsal **387** wings **388** hind femur lateral **389** head anterior **390** head dorsal **391** head lateral.

##### Biology.

Unknown.

##### Etymology.

From “scutellum” (Latin for “small shield”), because of the angulate and punctate scutellum.

### Genus Troticus

Brullé, 1846

#### Key to Vietnamese species of the genus Troticus Brullé

**Table:** 

1.	Fore wing with a large stigmal spot connected to a dark brown band below pterostigma (Figs 398, 404); apical half of wings dark brown; fore and middle tarsus robust (Fig. 398); first metasomal tergite about 0.8 times as long as its apical width (Fig. 403); second metasomal suture finely impressed (Fig. 403); ovipositor sheath truncate apically	Troticus giganteus sp. n.
–	Fore wing with a small isolated stigmal spot (Fig. 396); apical half of wings largely yellowish; fore and middle tarsus slender (Fig. 392); first tergite about 1.5 times as long as apical width (Fig. 397); second metasomal suture absent (Fig. 397); ovipositor sheath sharp apically	Troticus alloflavus sp. n.

#### Troticus alloflavus sp. n.

urn:lsid:zoobank.org:act:5F43E4F7-EF07-4C1D-A133-8DDC5736B0A8

Figs 392 –397 

##### Type material.

Holotype, ♀ (IEBR), Aga. 170, “S. Vietnam: Kien Giang, Phu Quoc N.P., 4.xii.2003, N.Th. Huong”.

##### Diagnosis.

The colour pattern of this species seems to be unique in the genera Troticus and Disophrys, having the wings completely yellowish and having only a small isolated stigmal spot.

##### Description.

Holotype, ♀, length of body 16.0 mm, of fore wing 16.0 mm.

###### Head.

Antennal segments 68, length of third segment subequal to fourth segment, length of third, fourth and penultimate segments 1.8, 2.0 and 1.2 times their width, respectively; apical antennal segment long conical, 2.6 times as long as penultimate segment; length of maxillary palp 0.7 times height of head; in dorsal view length of eye 1.3 times temple; temple gradually narrowed (Fig. 394); ocelli large, POL:OD:OOL = 10:9:24; face with dense distinct fine punctures; lateral carina of frons near antennal sockets (Fig. 394); pair of crests between antennal sockets slightly convergent; frons, vertex and temple smooth, sparsely setose.

###### Mesosoma.

Length of mesosoma 1.5 times its height; subpronope very deep and large; pronotum smooth laterally, crenulate anteriorly; area near lateral carina of mesoscutum smooth; side of mesoscutum shiny and smooth, medio-posteriorly slightly depressed, middle lobe convex; notauli wide, deep and coarsely crenulate; scutellar sulcus short with 3 carinae and 0.4 times as long as dorsal part of scutellum (Fig. 396); scutellum distinctly narrowed with sharp distinct punctures, subposterior crest strong and slightly curved; mesopleuron below precoxal sulcus finely punctate and above sulcus shiny, almost smooth; precoxal sulcus complete, deep and strongly crenulate, with one crenula connected to prepectal carina (Fig. 395); metapleuron finely punctate; propodeum with pentagonal areola and medial carina basally; spiracle very large, subelliptical, 2.4 times as long as wide (Fig. 396), distance between spiracle and lateral carina 0.5 times as long as width of spiracle.

###### Wings.

Fore wing: second submarginal cell large, pentagonal, narrow anteriorly, without ramellus (Fig. 393); r:3-SR:SR1= 6:10:75; 2-SR:3-SR:r-m = 12:10:14. Hind wing: M+CU rather long, 0.8 times as long as 1-M (42:50); surroundings of cu-a densely setose.

###### Legs.

Hind coxa rather long, hind femur robust, length of hind femur, tibia and basitarsus 4.0, 7.2 and 12.6 times their width, respectively; hind femur (as remainder of legs) with long and dense setosity; outer side of apex of hind tibia with two equal and distal each other pegs; length of outer and inner spurs of middle tibia 0.3 and 0.4 times middle basitarsus, respectively; length of outer and inner spurs of hind tibia 0.3 and 0.4 times hind basitarsus; fore and middle tarsi slender.

###### Metasoma.

First tergite slender, smooth, slightly widened apically (Fig. 397); length of first tergite 1.5 times its apical width, slightly depressed laterally; second metasomal suture absent (Fig. 397), ovipositor sheath short conical, 0.2 times as long as inner hind tibial spur.

###### Colour.

Yellow; antenna brown but scapus yellow, blackish ventrally; fore wing with isolated stigmal spot.

##### Distribution.

S Vietnam: Kien Giang.

**Figure 392.**
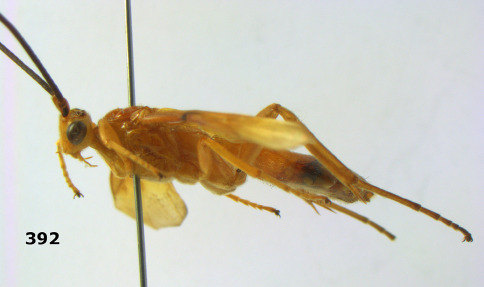
Troticus alloflavus sp. n., female, holotype. Habitus lateral.

**Figures 393–397.**
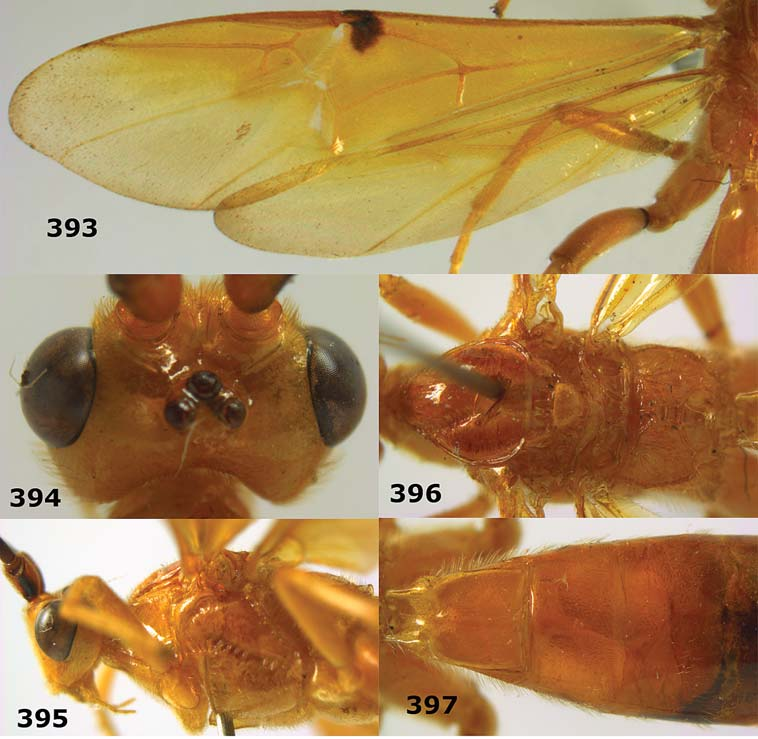
Troticus alloflavus sp. n., female, holotype. **393** wings **394** head dorsal **395** head and mesosoma lateral **396** mesosoma dorsal **397** first-third metasomal tergites dorsal.

##### Biology.

Unknown.

##### Etymology.

From “allo” (Greek for “other”), and “flavus” (Latin for “yellow”), because of the different yellow colour pattern.

#### Troticus giganteus sp. n.

urn:lsid:zoobank.org:act:73FC381C-CB07-4264-8FAD-00694A67FC32

Figs 398 –405 

##### Type material.

Holotype, ♀ (RMNH), Aga. 296, “Central North Vietnam: Nghe An, Pu Mat N.P., 17.iv.2006, P.Th. Nhi”.

##### Diagnosis.

Similar to Troticus latiabdominalis (Bhat, 1978) comb. n. from India. The latter has a short ramellus at the second submarginal cell of the fore wing, the vertex and the occiput brownish, the scutellar sulcus with 5 carinae, temple, face laterally and malar space yellowish-brown and the first metasomal tergite about as long as its apical width. Also rather similar to Euagathis gracilitarsis van Achterberg & Chen, 2002, from China, but differs by having the first tergite wider (its length 0.8 times its apical width; Euagathis gracilitarsis: 1.7 times its apical width), the second submarginal cell of the fore wing distinctly narrowed anteriorly (Euagathis gracilitarsis: slightly narrowed anteriorly), the temple largely black (Euagathis gracilitarsis: yellowish-brown), the metasoma yellowish-brown (Euagathis gracilitarsis: largely blackish) and the the pale patch below pterostigma large (Euagathis gracilitarsis: small).

##### Description.

Holotype, ♀, length of body 14.6 mm, of fore wing 13.5 mm.

###### Head.

Antennal segments 62, length of third segment 1.2 times fourth segment, length of third, fourth and penultimate segments 2.1, 2.0 and 1.3 times their width, respectively; apical antennal segment obtuse; scapus slender; length of maxillary palp 0.7 times height of head; in dorsal view length of eye 1.3 times temple; temple straight laterally and weakly narrowed (Fig. 399); ocelli small, POL:OD:OOL = 10:6:22; face finely punctate; lateral carina of frons almost connected to rim of antennal sockets (Fig. 399); frons, vertex and temple smooth, sparsely setose; pair of crests between antennal sockets slightly convergent.

###### Mesosoma.

Length of mesosoma 1.5 times its height; subpronope very deep and large; pronotum smooth laterally, crenulate anteriorly; area near lateral carina of mesoscutum smooth; side of mesoscutum shiny and smooth, medio-posteriorly slightly depressed, middle lobe convex; notauli wide, deep and coarsely crenulate; scutellum slightly narrowed posteriorly, rugose-punctate, subposterior crest strong and curved; scutellar sulcus with 3 carinae and 0.6 times as long as dorsal part of scutellum (Fig. 401); mesopleuron below precoxal sulcus finely punctate and above sulcus shiny, almost smooth; precoxal sulcus deep, extending two thirds of mesopleuron and strongly crenulate, with 6 carinae; metapleuron finely punctate; propodeum coarsely areolate with pentagonal areola and medial carina basally (Fig. 401); spiracle large, elliptical, 2.5 times as long as wide, distance between spiracle and lateral carina as long as width of spiracle.

###### Wings.

Fore wing: second submarginal cell large, pentagonal, narrow anteriorly, without ramellus (Fig. 404); r:3-SR:SR1= 3:7:61; 2-SR:3-SR:r-m = 10:7:11. Hind wing: M+CU rather long, 0.8 times as long as 1-M (32:40) (Fig. 405); surroundings of cu-a densely setose.

###### Legs.

**Figures 398.**
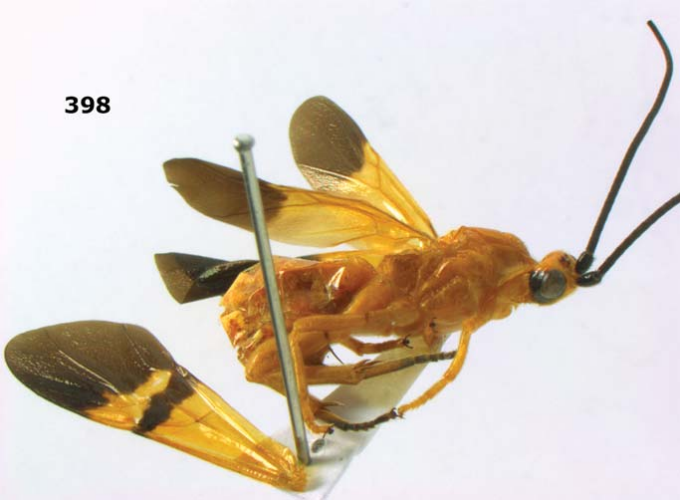
Troticus giganteus sp. n., female, holotype. Habitus lateral.

Hind coxa rather long, hind femur robust, length of hind femur, tibia and basitarsus 2.8, 5.6 and 6.2 times their width, respectively; hind femur (as remainder of legs) with long and dense setosity; outer side of apex of hind tibia with two distal each other pegs; length of outer and inner spurs of middle tibia 0.5 and 0.6 times middle basitarsus, respectively; length of outer and inner spurs of hind tibia 0.3 and 0.6 times hind basitarsus; fore and middle tarsi robust (Fig. 398).

###### Metasoma.

First tergite short, smooth, slightly widened apically (Fig. 403); length of first tergite 0.8 times its apical width, slightly depressed laterally; second metasomal suture finely impressed (Fig. 403), ovipositor sheath short, truncate apically, with long setae, 0.3 times as long as inner hind tibial spur; visible part of ovipositor 0.8 times as long as inner hind tibial spur.

###### Colour.

Yellowish-brown; antennae, malar space and temple largely black; basal half of wings and pterostigma yellow (Figs 398, 404, 405); large stigmal spot being part of large dark brown band below parastigma; apical third of wings and hind tarsus (except basitarsus) dark brown.

##### Distribution.

Central North Vietnam: Nghe An.

**Figures 399–405.**
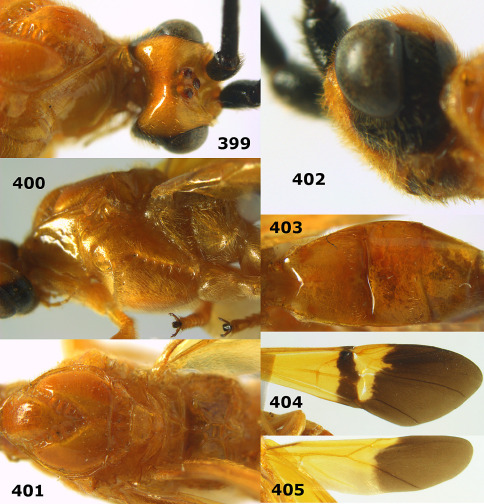
Troticus giganteus sp. n., female, holotype. **399** head dorsal **400** mesosoma lateral **401** mesosoma dorsal **402** head lateral **403** first-third metasomal tergites dorsal **404** fore wing **405** hind wing.

##### Biology.

Unknown.

##### Etymology.

From “gigantos” (Greek for “giant”), because is one of the largest known species of the genus.

### Genus Zelodia

van Achterberg gen. n.

urn:lsid:zoobank.org:act:BC1F3C94-29FB-49C3-8571-91581BFEE8F0

#### Type species.

Zelomorpha varipes van Achterberg & Maetô, 1990 [examined].

#### Etymology.

From “zelo” (first part of the genus name Zelomorpha) and “dia” (Latin for “between” because of its resemblance to the New World genus Zelomorpha Ashmead, 1900. Gender: feminine.

#### Diagnosis.

Apex of antenna with spine; area between antennal sockets with a pair of crests (Figs 415, 425), rarely with a trough; frons without a pair of carinae or crests laterally; malar space smooth, often comparatively long, usually hardly or not protruding posteriorly (Fig. 426); temples medium-sized (Fig. 425); notauli at least in anterior half of mesoscutum impressed and comparatively narrow (Fig. 421); vein M+CU of hind wing at most 0.8 times as long as vein 1-M; fore spur with a short, straight and setose apical spine (Figs 418, 436); fore tarsal claws bifurcate, its inner tooth usually as large as its outer tooth, but sometimes smaller; outer face of middle tibia only with apical pegs (Figs 427, 436); inner spur of middle tibia 0.8–1.1 times as long as middle basitarsus (Figs 409, 436); hind coxa without longitudinal carina or rugae; hind trochantellus usually with a distinct ventral carina on its outer edge or edge distinctly angulate; ventral surface of hind femur distinctly sculptured; ovipositor sheath short, about as long as apical height of metasoma, hardly or not protruding in dorsal view; apex of the ovipositor sheath rather blunt apically and with numerous ampulliform papillae.

#### Phylogenetic position.

Probably sister to the genus Euagathis Szépligeti according to Sharkey et al. (2009), but it belongs to the Coccygidium complex. It shares with the complex two synapomorphies: the apical spine of the antenna and the elongate inner spur of the middle tibia.Apomorphous character states of the new genus Zelodia within the Coccygidium complex are the comparatively narrow notauli and the densely sculptured ventral face of the hind femur. The genus Coccygidium de Saussure has the following synapomorphies: the fore spur with a long glabrous apical spine, the hind coxa with a longitudinal carina or rugae and the frons with more or less developed carinae or crests.

#### Notes.

Sharkey et al. (2006) included the Zelomorpha sulana group of Bhat and Gupta (1977) in Hypsostypos Baltazar, because of the rugose ventral surface of the hind femur and some other character-states. However, the type species of Hypsostypos has several autapomorphies and lacks the apical spine of the antenna typical for the Coccygidium complex to which this group belongs. Therefore, the Zelomorpha sulana group is excluded from Hypsostypos and Sharkey et al. (2009) intended to name this group Amputostypos. Unfortunately, they overlooked that the designated type species (Disophrys concolor Szépligeti, 1908) does not belong to this group. To the newly proposed genus Zelodia belong the following described species (all are new combinations):

Zelodia absoluta (Chen & Yang, 1998) China

Zelodia achterbergi (Chen & Yang, 2006) China

Zelodia albopilosella (Cameron, 1908) Sarawak

Zelodia chromoptera (Roman, 1913) Philippines (Mindanao)

Zelodia cordata (Bhat & Gupta, 1977) India, Burma, Singapore

Zelodia diluta (Turner, 1918) Australia (Queensland)

Zelodia dravida (Bhat & Gupta, 1977) India, China

Zelodia exornata (Turner, 1918) Australia (Queensland)

Zelodia longidorsata (Bhat & Gupta, 1977) India

Zelodia longiptera (Yang & Chen, 2006) China

Zelodia maculipes (Cameron, 1911) New Guinea (ZMA)

Zelodia nigra (Bhat & Gupta, 1977) Philippines (Mindoro), ? China

Zelodia nihonensis (Sharkey, 1996) Far East Russia, China, Korea, Japan.

Syn.: Coccygidium concolor sensu Chou & Sharkey (1989) and Sharkey et al. (2009).

Zelodia penetrans (Smith, 1860) Sulawesi, North Moluccas

Zelodia philippinensis (Bhat & Gupta, 1977) Philippines (Mindoro)

Zelodia quadrifossulata (Enderlein, 1920) Sundaland (Sumatra LT, Singapore), ? India, ? Sri Lanka, ? Nepal, ? Philippines (Leyte, Mindoro, Luzon, Negros)

Zelodia reticulosa (Yang & Chen, 2006) China

Zelodia ruida (Sharkey, 1996) Japan, Korea

Zelodia similis (Bhat & Gupta, 1977) Philippines (Mindoro, Palawan)

Syn.: Euagathis sulana Enderlein, 1920.

Zelodia varipes (van Achterberg & Maetô, 1990) Japan, China

#### Distribution.

East Palaearctic, Oriental, Australian and (rarely) in Afrotropical region.

#### Biology.

Unknown, but phylogenetically related genera contain parasitoids of Noctuidae.

#### Key to Vietnamese species of the genus Zelodia van Achterberg

**Table:** 

1.	Hind tibia and apical half of metasoma black (Figs 455, 406, 409, 418, 458); basal half of pterostigma dark brown or blackish	2
–	Hind tibia (except apically) and apical half of metasoma yellowish-brown (Figs 427, 436, 446); basal half of pterostigma yellow or largely so	6
2.	First metasomal tergite with coarsely rugose glymma and with distinct dorso-lateral carina above spiracle (Fig. 457); notum of second tergite 1.5–1.6 times as long as wide apically (Fig. 457); [first tergite black and 3–4 times as long as wide; hind coxa rugose dorsally]	Zelodia longidorsata (Bhat & Gupta, 1977)
–	First tergite with smooth glymma or glymma absent and without distinct dorso-lateral carina above spiracle (Figs 408, 409, 423); notum of second tergite about as long as wide apically (Figs 412, 423)	3
3.	First metasomal tergite distinctly widened apically and 1.7–1.9 times as long as wide apically (Figs 408, 462); length of malar space 2.0–2.2 times basal width of mandible; head comparatively long in anterior view (Figs 407, 465); propodeum and first tergite black	4
-	First tergite parallel-sided and 3–4 times as long as wide apically (Figs 412, 423); length of malar space 1.1–1.5 times basal width of mandible; head comparatively short in anterior view (Figs 414, 424); colour of propodeum and first tergite variable, often pale yellowish or ivory	5
4.	Hind coxa distinctly punctate-rugose (Fig. 408); first metasomal tergite black and with some punctures near apex of tergite sublaterally (Fig. 408); ocelli medium-sized	Zelodia absoluta (Chen & Yang, 1998)
–	Hind coxa mainly punctate (Fig. 458); first tergite pale yellowish or ivory and without punctures near apex of tergite sublaterally (Fig. 458); ocelli small (Fig. 466)	Zelodia microcellata sp. n.
5.	Propodeum, first and second tergites pale yellowish or ivory (Fig. 409); hind coxa rugulose dorsally and dark brown; head yellowish-brown; middle tarsal spurs similar in colour to pale yellowish middle tarsus	Zelodia albobasalis sp. n.
–	Propodeum, first and second tergites black (Fig. 418); hind coxa punctate dorsally and black; head black; middle tarsal spurs dark brown and contrasting with whitish middle tarsus	Zelodia anginota sp. n.
6.	Apical half of pterostigma yellow (Fig. 450); length of malar space 1.3–1.6 times basal width mandible (Fig. 453); OOL 1.0–1.2 times POL (Fig. 452)	Zelodia flavistigma sp. n.
–	Apical half of pterostigma dark brown (Figs 430, 440); length of malar space 2.4–3.0 times basal width mandible (Figs 434, 445); OOL 1.6–2.0 times POL (Figs 433, 444)	7
7.	Hind femur comparatively robust (Fig. 441); hind coxa and femur and more or less scapus and pedicellus yellow; hind femur weakly rugose or rugulose ventrally	Zelodia brevifemoralis sp. n.
–	Hind femur slender (Fig. 431); hind coxa, femur, scapus and pedicellus dark brown or largely so; hind femur coarsely rugose ventrally	Zelodia bicoloristigma sp. n.

#### Zelodia absoluta

(Chen & Yang, 1998) comb. n.

Figs 406–408

Coccygidium absolutus Chen & Yang, 1998 (in Yang and Chen 1998): 334–336; Chen and Yang 2006: 115–116, Fig. 41, plts 90–98.

##### Distribution.

NE Vietnam: Ha Giang, Hoa Binh, Ninh Binh; CN Vietnam: Ha Tinh; C Vietnam: Thua Thien-Hue. Outside Vietnam only known from China (Fujian). Vietnam is a new record.

**Figures 406–408.**
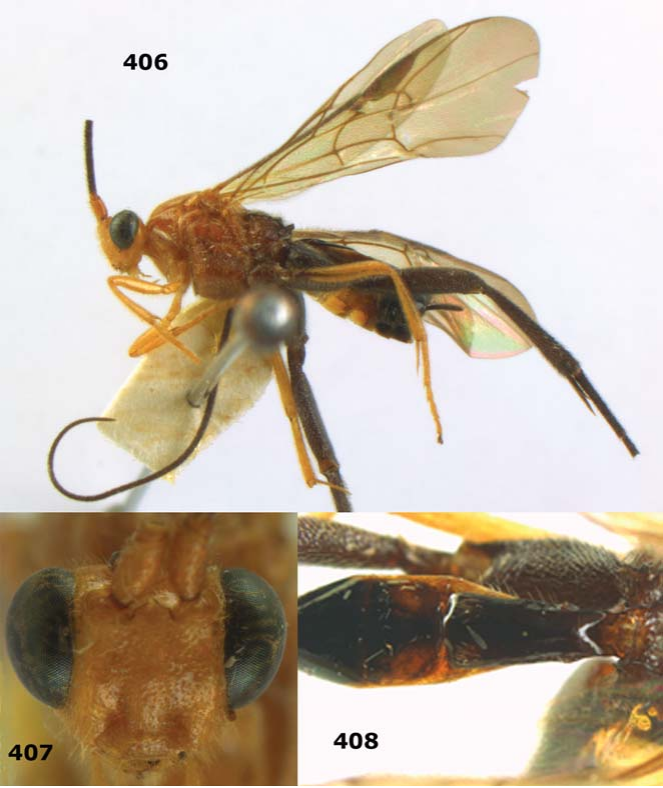
Zelodia absoluta (Chen & Yang), female, Ky Son. **406** habitus lateral **407** head anterior **408** first-third metasomal tergites dorsal.

##### Notes.

The holotype is a male, not a female as indicated in the original description. The sculpture of the notauli is variable: it varies from distinctly crenulate to nearly smooth. Also the shape of the first tergite is rather variable, as robust as depicted by Chen & Yang (2006) to fairly slender. This species resembles Zelodia quadrifossulata (Enderlein) from Sundaland (e.g., by the robust first metasomal tergite with some punctures latero-apically), but the latter differs by having the hind coxa largely smooth dorsally, the propodeum and the metanotum yellowish-brown, the scutellum moderately punctate and with distinct interspaces between the punctures, the mesopleuron only punctulate and the hind femur less slender. Very similar to Zelodia achterbergi (Chen & Yang), but that species differs mainly by the sparsely punctate mesopleuron (interspaces wider than diameter of punctures) and no distinct rugae near the prepectal carina. Also similar to Zelodia similis (Bhat & Gupta) from Philippines, but Zelodia similis has the hind leg and metasoma (blackish)-brown, OOL less than POL, face moderately punctate, length of malar space twice basal width of mandible, scutellum sparsely and moderately punctate, second tergite parallel-sided and comparatively slender.

#### Zelodia albobasalis sp. n.

urn:lsid:zoobank.org:act:35F40410-E562-4E7B-AB84-DC6604785443

Figs 409 –417 

##### Type material.

Holotype, ♀ (RMNH), “S. Vietnam: Dong Nai, Cat Tien N.P., Mal. traps c. 100 m, 9.iv-13.v.2007, Mai Phu Quy & Nguyen Thanh Manh, RMNH’07”.

##### Diagnosis.

The new species is similar to Zelodia similis (Bhat & Gupta, 1977) comb. n., from Philippines, but differs by having POL 0.9 times as long as OOL (Zelodia similis: 2.5 times); the malar space 1.3 times as long as basal width of the mandible (similis: twice) and the first tergite about 3 times as long as its apical width (similis: about 1.7 times). Alsosimilar to Zelodia quadrifossulata (Enderlein, 1920) comb. n., but differs by having the malar space comparatively short (Zelodia quadrifossulata: twice as long as basal width of mandible); the propodeal spiracle small (quadrifossulata: rather large) and the first tergite of female almost parallel-sided (quadrifossulata: distinctly widened apically).

Zelodia longiptera (Yang & Chen, 2006) comb. n. is similar, but that species has smaller ocelli (OOL 1.5 times POL), length of the malar space twice basal width of the mandible, the first tergite about twice as long as its apical width (not 3.2 times as indicated in the original description), the hind femur 4.3 times as long as wide, the scapus dark brown and the wings subhyaline.

##### Description.

Holotype, ♀, length of body 6.2 mm, of fore wing 5.7 mm.

###### Head.

Antennal segments 41; length of third segment 1.1 times as long as fourth segment; length of third, fourth and penultimate segments 2.3, 2.0 and 1.5 times their width, respectively; penultimate segment as long as apical segment without spine; antenna densely setose; length of maxillary palp 0.8 times as long as height of head; in dorsal view length of eye 3.0 times temple (Fig. 415); in lateral view 2.5 times as wide as width of eye (Fig. 410); ocelli large, POL:OD:OOL = 9:8:10; malar space 1.3 times as long as basal width of mandible, 0.3 times height of eye and 0.2 times as long as height of head in lateral view (Figs 410, 417); face distinctly punctate; frons shiny and smooth without lateral carinae; vertex shiny with sparse fine punctures; pair of crests between antennal sockets short, occipital flange medium-sized, its ventral margin convex (Fig. 410).

###### Mesosoma.

Length of mesosoma 1.3 times its height; subpronope small, deep; side of pronotum smooth, upper side with sparse fine punctures; area near lateral carina of mesoscutum smooth; mesoscutum shiny with sparse distinct punctures; notauli shallow areolate-punctate (Fig. 411); scutellar sulcus with three carinae and 0.8 times as long as dorsal part of scutellum; scutellum rugose-punctate; subposterior crest curved (Fig. 411); precoxal sulcus rather wide and strongly crenulate (Fig. 410); mesopleuron below and above precoxal sulcus with sparse and distinct fine punctures; upper side of metapleuron rugose-punctate, lower side with strong rugae; propodeum with long pentagonal areola, short basal carina (Fig. 411); propodeal spiracle small, round, twice as long as wide; distance between spiracle and lateral carina 1.2 times as long as width of spiracle.

###### Wings.

Fore wing: second submarginal cell narrow anteriorly, without ramellus (Fig. 416); r:3-SR:SR1 = 5:3:100; 2-SR:3-SR:r-m = 17:5:13. Hind wing: M+CU 0.6 times as long as 1-M (19:33); surroundings of cu-a sparsely setose.

###### Legs.

Length of hind femur, tibia and basitarsus 3.5, 3.9 and 4.5 times their width, respectively; outer side of hind coxa punctate; hind femur (as remainder of legs) with long and dense setosity (Fig. 413); outer side of apex of hind tibia with two pegs, and upper peg twice as long as lower peg; outer and inner spurs of middle tibia 0.5 and 0.8 times as long as middle basitarsus, respectively; length of outer and inner spurs of hind tibia 0.4 and 0.6 times hind basitarsus.

###### Metasoma.

First tergite rather long almost parallel-sided; length of first tergite 2.9 times its apical width (Fig. 412); length of second tergite subequal to its apical width; second metasomal suture developed; ovipositor sheath densely setose, 0.4 times as long as hind basitarsus.

###### Colour.

**Figure 409.**
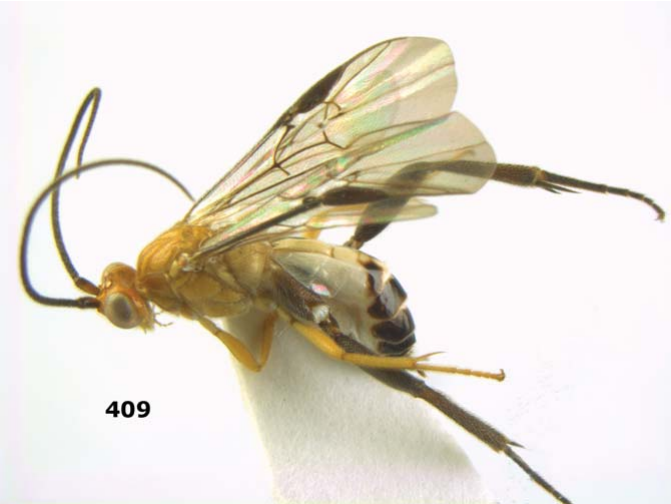
Zelodia albobasalis gen. n. sp. n., female, holotype. Habitus lateral.

Brownish-yellow; antenna (but scapus yellow basally), apex of vein C+SC+R and pterostigma dark brown; hind leg black or blackish brown; first-third metasomal segments white (but third tergite black dorso-laterally); remainder of metasomal segments black and metasoma ivory ventrally; wings slightly infuscate (Fig. 409).

##### Distribution.

S Vietnam: Dong Nai.

**Figures 410–417.**
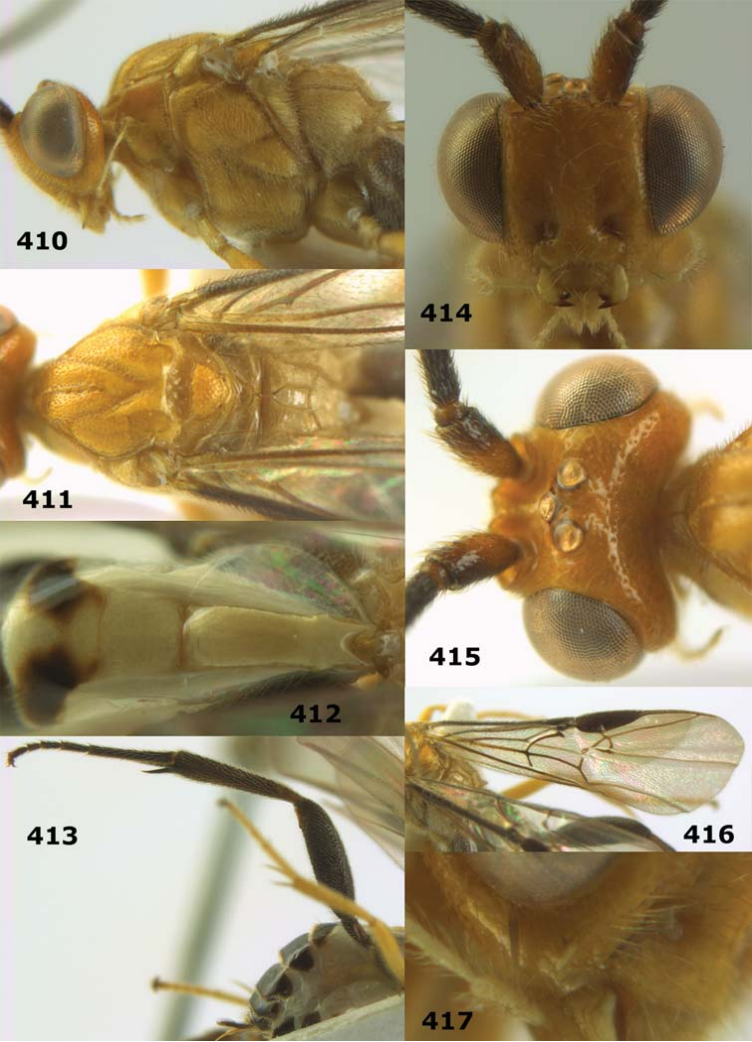
Zelodia albobasalis gen. n. sp. n., female, holotype. **410** mesosoma lateral **411** mesosoma dorsal **412** first-third metasomal tergites dorsal **413** hind leg lateral **414** head anterior **415** wings **416** head dorsal **417** lower part of head lateral.

##### Biology.

Unknown.

##### Etymology.

From “albus” (Latin for “white”) and “basis” (Latin for “foundation, base”), because of the white base of the metasoma.

#### Zelodia anginota sp. n.

urn:lsid:zoobank.org:act:D43D14EE-B5E8-4557-B0A2-96E4BCC65CDB

Figs 418 –426 

##### Type material.

Holotype, ♀ (RMNH), “S. Vietnam: Dak Lak, Chu Yang Sin N.P., n[ea]r dam, 744–900 m, 1–10.vi.2007, Mal[aise] trap, C. v. Achterberg & R. de Vries, RMNH’07”. Paratype: 1 ♀ (IEBR), Aga. 187, “NE Vietnam: Phu Tho, Xuan Son N.P., forest, 10.v.2005, P.Th. Nhi”.

##### Diagnosis.

The new species is close to Zelodia longidorsata (Bhat & Gupta, 1977), but differs by having the propodeal spiracle 3 times as long as wide (Zelodia longidorsata: twice); the first tergite without a dorso-lateral carina above the spiracle and the glymma smooth (longidorsata: dorso-lateral carina present above spiracle and glymma sculptured); the second tergite as long as wide apically (longidorsata: 1.5 times longer than wide) and the head and mesosoma black except for the reddish yellow mesonotum (longidorsata: head and mesosoma yellowish-brown).

##### Description.

Holotype, ♀, length of body 6.3 mm, of fore wing 6.4 mm.

###### Head.

Antennal segments 42, length of third segment 1.2 times fourth segment, length of third, fourth and penultimate segments 2.5, 2.1 and 1.7 times their width, respectively; penultimate segment 0.6 times as long as apical segment; antenna densely setose; scapus nearly cylindrical, 2.2 times as long as wide; length of maxillary palp 0.7 times height of head; malar space 1.5 times as long as basal width of mandible, 0.3 times height of eye and 0.2 times as long as height of head in lateral view (Fig. 426); in dorsal view eye twice as long as temple (Fig. 425); POL:OD:OOL = 9:8:13; face shiny, punctate, rugose-punctate medially; frons shiny, smooth and without lateral carinae; vertex shiny with sparse fine punctures; pair of crests between antennal sockets strong, convergent; occipital flange large, its ventral margin convex (Fig. 426).

###### Mesosoma.

Length of mesosoma 1.3 times its height; subpronope medium-sized, shallow; side of pronotum smooth and with sparse fine punctures posteriorly; area near lateral carina of mesoscutum smooth; mesoscutum with distinct punctures anteriorly, shiny and sparsely punctate posteriorly; middle lobe of mesoscutum slightly grooved; notauli narrow, rather deep and punctate, fused posteriorly, forming wide shallow groove (Fig. 421); scutellar sulcus rather long, with 3 carinae and 0.8 times as long as dorsal part of scutellum; scutellum convex, distinctly narrowed posteriorly, rugose-punctate; subposterior crest long and curved (Fig. 421); precoxal sulcus shallow, widely crenulate (Fig. 419); mesopleuron below sulcus largely punctate, and above sulcus with sparse fine punctures; metapleuron with long, dense setae, upper side areolate-rugose, lower side with strong rugae; propodeum setose basally with large areola and costulae, without a medial carina basally and with a weak anterior transverse carina (Fig. 421); propodeal spiracle large, elliptical, 3.0 times as long as wide; distance between spiracle and lateral carina as long as width of spiracle.

###### Wings.

Fore wing: second submarginal cell narrow anteriorly, without ramellus (Fig. 422); r:3-SR:SR1= 6:6:112; 2-SR:3-SR:r-m = 17:6:15. Hind wing: M+CU 0.5 times as long as 1-M (22:41); surroundings of cu-a setose.

###### Legs.

Length of hind femur, tibia and basitarsus 4.3, 6.5 and 11.3 times their width, respectively; outer side of hind coxa areolate-punctate; hind femur (as remainder of legs) with long and dense setosity (Fig. 420); outer side of apex of hind tibia with two pegs, and lower peg twice as long as upper peg; fore tarsus slender and with long setae; length of outer and inner spur of middle tibia 0.5 and 0.9 times middle basitarsus, respectively; length of outer and inner spur of hind tibia 0.4 and 0.7 times hind basitarsus.

###### Metasoma.

First tergite long, smooth, parallel-sided (Fig. 423); length of first tergite 3.6 times its apical width; second tergite rectangular and as long as wide apically, smooth with three transverse rows of setae apically; second metasomal suture absent (Fig. 423); ovipositor sheath 0.4 times as long as hind basitarsus.

###### Colour.

Black; mesonotum and mesopleuron reddish brown, but mesopleuron black posteriorly; fore and middle legs dark brown, but fore and middle tarsi whitish-yellow; first-third metasomal sternites white; pterostigma dark brown; wing membrane slightly infuscate, without stigmal spot.

###### Variation.

Length of body 6.3–7.3 mm; of fore wing 6.4–7.6 mm; antennal segments 42 (2); POL as long as OD or slightly larger; length of malar space 1.4–1.5 times basal width of mandible; first tergite slightly widened apically or parallel-sided, 3.1–3.6 times longer than wide apically.

##### Distribution.

NE Vietnam: Phu Tho and S Vietnam: Dak Lak.

**Figure 418.**
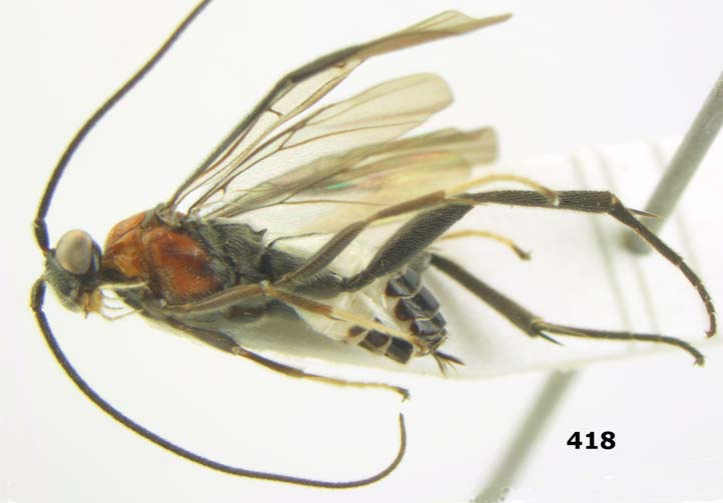
Zelodia anginota gen. n. sp. n., female, holotype. Habitus lateral.

**Figures 419–426.**
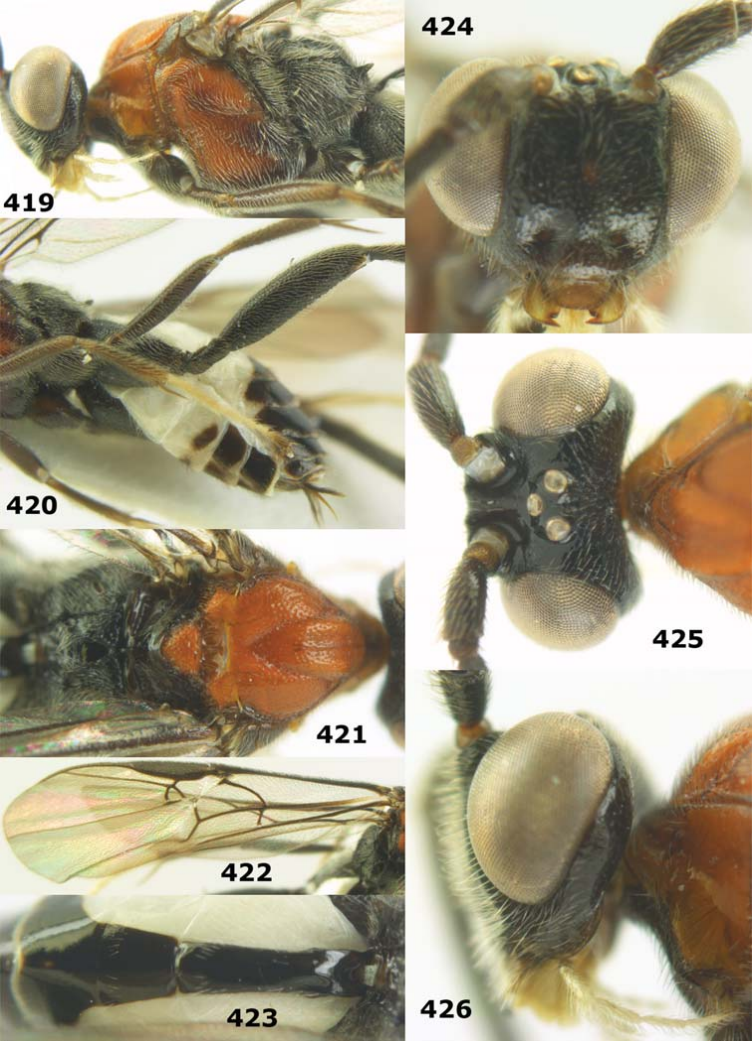
Zelodia anginota gen. n. sp. n., female, holotype. **419** mesosoma lateral **420** hind femur lateral **421** mesosoma dorsal **422** wings **423** first-third metasomal tergites dorsal **424** head anterior **425** head dorsal **426** head lateral.

##### Biology.

Unknown.

##### Etymology.

From “ango” (Latin for “pressed together”) and “notos” (Greek for “back”), because of the narrow first metasomal tergite.

#### Zelodia bicoloristigma sp. n.

urn:lsid:zoobank.org:act:4BD0A5CE-92A3-48F4-8A3A-51113196736A

Figs 427 –435 

##### Type material.

Holotype, ♀ (RMNH), “NW Vietnam: Tonkin, Hoang Lien N.P., 10 km SW Sa Pa, c. 1550 m, 23.x.1999, at light, C. v. Achterberg, RMNH’99”. Paratypes (4 ♀ + 6 ♂): 2 ♀ (RMNH), same data as holotype, but 22–29.x.1999, Malaise traps; 2 ♂ (RMNH), Aga. 297 & 288, “CN Vietnam: Nghe An, Con Cuong, Pu Mat N.P., 15 & 17.iv.2006, P.Th. Nhi”; 1 ♀ (IEBR), id., but Aga. 181, 22–25.vii.2004, Tr.X. Lam; 1 ♂ (IEBR), Aga 083, “Central North Vietnam (Truong Son range): Ha Tinh, Huong Son, Rao An, forest, 11.v.1998, K.D. Long”; 1 ♂ (IEBR), Aga. 186, “Northwest Vietnam: Lai Chau, Muong Lay, Hat Tre, forest fragment, 10.x.2004, K.D. Long”; 1 ♀ (IEBR), Aga. 192, “Northeast Vietnam: Phu Tho, Xuan Son N.P., forest, 5.v.2005, P.T. Nhi”; 1 ♀ + 1 ♂ (IEBR), Aga. 315 and 316, “Central Vietnam: Thua Thien-Hué, Bach Ma N.P., 600 m, 18 & 19.v.2007, K.D. Long”.

##### Diagnosis.

The new speciesis similar to Zelodia quadrifossulata (Enderlein), but the latter differs by having the pterostigma entirely brown, the hind leg dark brown and the metasoma (except for the more or less yellowish or brownish first and second tergites) black.

##### Description.

Holotype, ♀, length of body 7.6 mm, of fore wing 8.6 mm.

###### Head.

Antennal segments 43, densely bristly setose, length of third segment 1.1 times as long as fourth segment; length of third, fourth and penultimate segments 2.6, 2.3 and 1.8 times their width, respectively; penultimate segments half as long as apical segment; scapus long somewhat compressed, nearly twice as long as wide; length of maxillary palp 0.6 times as long as height of head; in dorsal view length of eye 2.3 times as long as temple (Fig. 433); temple punctulate and setose, in lateral view 0.6 times width of eye (Fig. 428); POL:OD:OOL = 6:5:10; malar space twice as long as basal width of mandible, 0.4 times as long as eye height and 0.3 times as long as height of head in lateral view (Fig. 434); face distinctly punctate, interspaces somewhat larger than punctures; frons shiny, smooth (except for some punctures) and without lateral carinae; vertex shiny, sparsely finely punctate; pair of crests between antennal sockets rather weakly developed, convergent; occipital flange large, its ventral margin round (Fig. 428).

###### Mesosoma.

Length of mesosoma 1.4 times its height; subpronope large, moderately deep; side of pronotum smooth, but dorsally upper side sparse finely punctate and crenulate postero-ventrally; area near lateral carina of mesoscutum slightly crenulate; middle lobe of mesoscutum distinctly punctate; lateral lobe of mesoscutum less densely and finer punctate than middle lobe except near notauli; notauli narrow, deep and nearly smooth (Fig. 429); scutellar sulcus subequal to dorsal part of scutellum and with transverse subposterior crest; scutellum rather coarsely punctate (Fig. 429); prepectal carina strong, rather lamelliform and crenulate behind it; precoxal sulcus moderately narrow and rather deep but absent anteriorly, moderately crenulate (Fig. 428); mesopleuron sparsely finely punctate; mesosternal sulcus narrow and finely crenulate; upper half of metapleuron coarsely punctate, lower side with strong vermiculate rugose; propodeum shiny and coarsely areolate and short medial carina basally (Fig. 429); propodeal spiracle elliptical, 2.5 times as long as wide; distance between spiracle and lateral carina equal to width of spiracle.

###### Wings.

Fore wing: second submarginal cell narrowed anteriorly and r-m nearly straight (Fig. 430); r:3-SR:SR1 = 10:5:171; 2-SR:3-SR:r-m = 25:5:22. Hind wing: M+CU 0.67 times as long as 1-M; surroundings of cu-a sparsely seto

###### Legs.

Hind coxa dorsally punctate and pimply; length of hind femur, tibia and basitarsus 4.5, 6.7 and 8.2 times their width, respectively; outer side of hind coxa punctate-pimply; hind femur and tibia with rather long setae ventrally (Fig. 431); outer side of apex of hind tibia with two pegs; outer and inner spurs of middle tibia 0.5 and 1.0 times as long as middle basitarsus, respectively; length of outer and inner spurs of hind tibia 0.4 and 0.7 times hind basitarsus.

###### Metasoma.

First tergite rather slender and smooth; length of first tergite 2.1 times its apical width (Fig. 435); length of second tergite 0.8 times apical width; second metasomal suture obsolescent (Fig. 435); ovipositor sheath densely setose apically elliptical, 0.5 times as long as hind basitarsus and 0.11 times fore wing.

###### Colour.

Yellowish-brown, but mesoscutum largely somewhat darkened and first and second tergites pale yellowish; hind coxa, trochantellus, trochantellus and largely femur, hind tibial spurs, ovipositor sheath, scapus and pedicellus largely dark brown; remainder of antenna, apex of hind tibia and tarsus blackish; wings subhyaline but near parastigma and vein r infuscate; parastigma, apical part of vein C+SC+R, apical half of pterostigma and veins of apical half of fore wing largely dark brown.

###### Variation.

Antennal segments 43–44; face moderately coarsely to finely spaced punctate; length of first tergite 1.7–2.1 times as long as its apical width; first and second tergites ivory or pale yellowish; mesosternum and third tergite more or less darkened. Males have basal third of pterostigma yellow and remainder dark brown.

##### Distribution.

NW Vietnam: Lao Cai, Lai Chau; NE Vietnam: Phu Tho; CN Vietnam: Nghe An, Ha Tinh and C Vietnam: Thua Thien-Hué.

**Figure 427.**
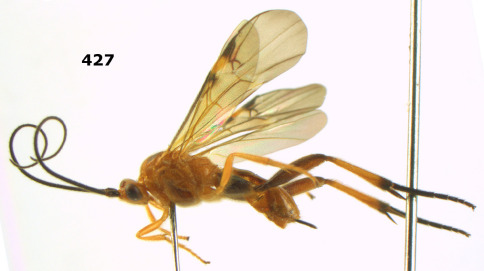
Zelodia bicoloristigma gen. n. sp. n., female, holotype. Habitus lateral.

**Figures 428–435.**
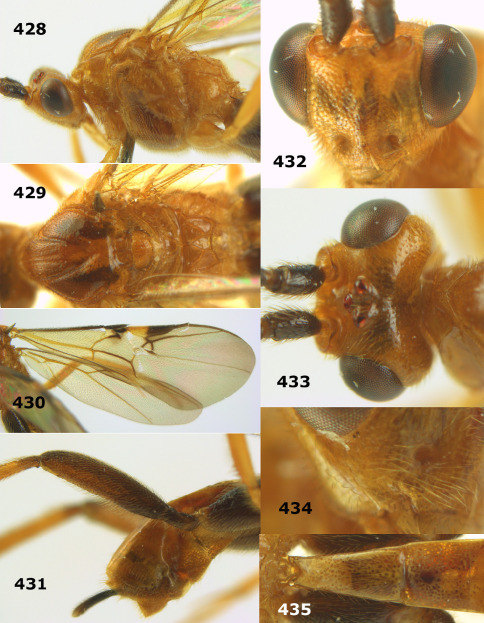
Zelodia bicoloristigma gen. n. sp. n., female, holotype. **428** head and mesosoma lateral **429** mesosoma dorsal **430** wings **431** hind femur lateral **432** head anterior **433** head dorsal **434** lower part of head lateral **435** first-third metasomal tergites dorsal.

##### Biology.

Unknown.

##### Etymology.

From “bi” (Latin for “two”), “coloris” (Latin for “hue, tint”) and “stigma” (Greek for “mark”), because of the two colours of the pterostigma.

#### Zelodia brevifemoralis sp. n.

urn:lsid:zoobank.org:act:AC1D7A26-3C5A-4E51-A57F-EC93EEBD10C4

Figs 436 –445 

##### Type material.

Holotype, ♀ (RMNH), “S. Vietnam: Kon Tum, Chu Mom Ray N.P., Mal. traps, 700–900 m, 26.ix.2006, Mai Phu Quy & Nguyen Thanh Manh, RMNH’07”. Paratypes (1 ♀ + 6 ♂): 1 ♀ (IEBR), Aga. 176, “Central North Vietnam: Ha Tinh, Huong Son, forest 8.v.2004, Tr.X. Lam; 1 ♂ (IEBR), Aga. 218, “N.W Vietnam: Yen Bai, Luc Yen, Phuc Loi, forest, 7.x.2003, K.D. Long”; 1 ♂ (RMNH), Aga. 352, “NE Vietnam: Ha Giang, Vi Xuyen, Minh Tan, bushes, 29.x.2006, K.D. Long”; 1 ♂ (IEBR), Aga. 253b, “NE Vietnam: Bac Kan, Ba Be N.P., forest, 17.vii.2004, K.D. Long”; 2 ♂ (RMNH, IEBR), “C. Vietnam: Ha Tinh, Vu Quang N.P., 139 m, 18°17'41N; 105°25'34E, 24.ix.-5.x.2009, Exp[erimental] trap 15, C. v. Achterberg & R. de Vries, RMNH’09"; 1 ♂ (RMNH), id., but Malaise trap 10, 53 m, 18°21'02N; 105°26'34E, 22.ix.-6.x.2009.

##### Diagnosis.

The new speciesis similar to Zelodia flavistigma sp. n., but differs by having the malar space about 3 times as long as basal width of mandible (Zelodia flavistigma: 1.3–1.6 times); vein r of fore wing shorter than vein 3-SR (flavistigma: longer than vein 3-SR) and face with distinct fine punctation (flavistigma: face medially rugose-punctate).

##### Description.

Holotype, ♀, length of body 7.0 mm, of fore wing 6.7 mm.

###### Head.

Antennal segments 47, length of third segment 1.2 times as long as fourth segment; length of third, fourth and penultimate segments 2.2, 1.8 and 1.7 times their width, respectively; penultimate segment as long as apical segment without spine; antenna densely setose; scapus nearly cylindrical, twice as long as wide; maxillary palp as long as height of head; in dorsal view length of eye 3.0 times as long as temple (Fig. 444); in lateral view 0.7 times width of eye (Fig. 445); POL:OD:OOL = 3:2:5; malar space 3.1 times as long as basal width of mandible, 0.5 times as long as eye height and 0.3 times as long as height of head in lateral view (Figs 442, 445); face distinctly punctate; frons shiny smooth without lateral carinae; vertex shiny, almost smooth; pair of crests between antennal sockets short, convergent; occipital flange rather large, its ventral margin convex (Fig. 437).

###### Mesosoma.

Length of mesosoma 1.5 times its height; subpronope small, deep; side of pronotum smooth, upper side with sparse fine punctures; area near lateral carina of mesoscutum sparsely crenulate; middle lobe of mesoscutum punctate; lateral lobe of mesoscutum almost smooth; notauli wide, shallow and sparsely crenulate (Fig. 438); scutellar sulcus subequal to dorsal part of scutellum and with one carina; scutellum rugose-punctate; subposterior crest curved (Fig. 438); precoxal sulcus wide and shallow, sparsely crenulate (Fig. 437); mesopleuron below and above precoxal sulcus with sparse and distinct fine punctures; upper side of metapleuron rugose-punctate, lower side with strong rugae; propodeum shiny with areola and short medial carina basally (Fig. 438); propodeal spiracle round, 2.2 times as long as wide; distance between spiracle and lateral carina equal to width of spiracle.

###### Wings.

Fore wing: second submarginal cell narrowed anteriorly (Fig. 440); vein r very short, r:3-SR:SR1= 3:4:110; 2-SR:3-SR:r-m = 16: 3:15. Hind wing: M+CU 0.6 times as long as 1-M; surroundings of cu-a sparsely setose.

###### Legs.

Length of hind femur, tibia and basitarsus 3.4, 5.3 and 12.0 times their width, respectively; outer side of hind coxa punctate; hind femur (as remainder of legs) with long and dense setosity (Fig. 441); outer side of apex of hind tibia with two pegs, and upper peg twice as long as lower peg; length of outer and inner spurs of middle tibia 0.6 times and subequal to middle basitarsus, respectively; length of outer and inner spurs of hind tibia 0.5 and 0.7 times hind basitarsus.

###### Metasoma.

First tergite rather long; length of first tergite twice its apical width (Fig. 439); length of second tergite 0.8 times its apical width; second metasomal suture fine; ovipositor sheath densely setose, 0.4 times as long as hind basitarsus.

###### Colour.

Yellow; antenna (except yellow scapus) dark brown; wings yellow subhyaline; parastigma, apex of vein C+SC+R, apical half of pterostigma and vein 1-R1 dark brown.

###### Variation.

Male: antennal segments 43–45; length of first tergite 1.8 times as long as its apical width; first-third segments of hind tarsus dark brown.

##### Distribution.

NE Vietnam: Ha Giang, NW Vietnam: Yen Bai, CN Vietnam: Ha Tinh and S Vietnam: Kon Tum.

**Figure 436.**
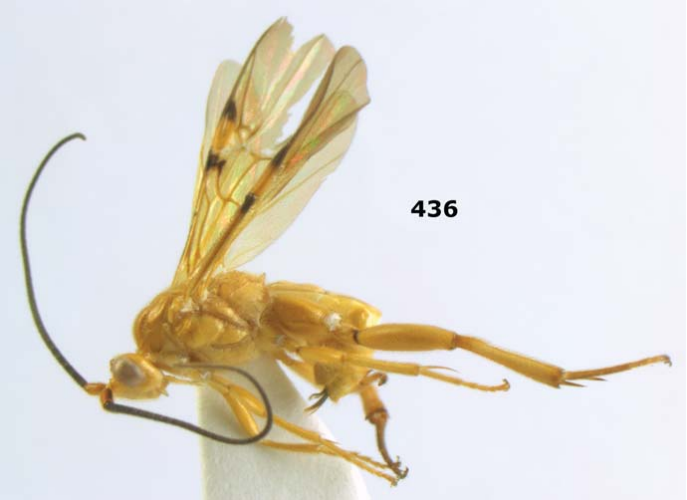
Zelodia brevifemoralis gen. n. sp. n., female, holotype. Habitus lateral.

**Figures 437–445.**
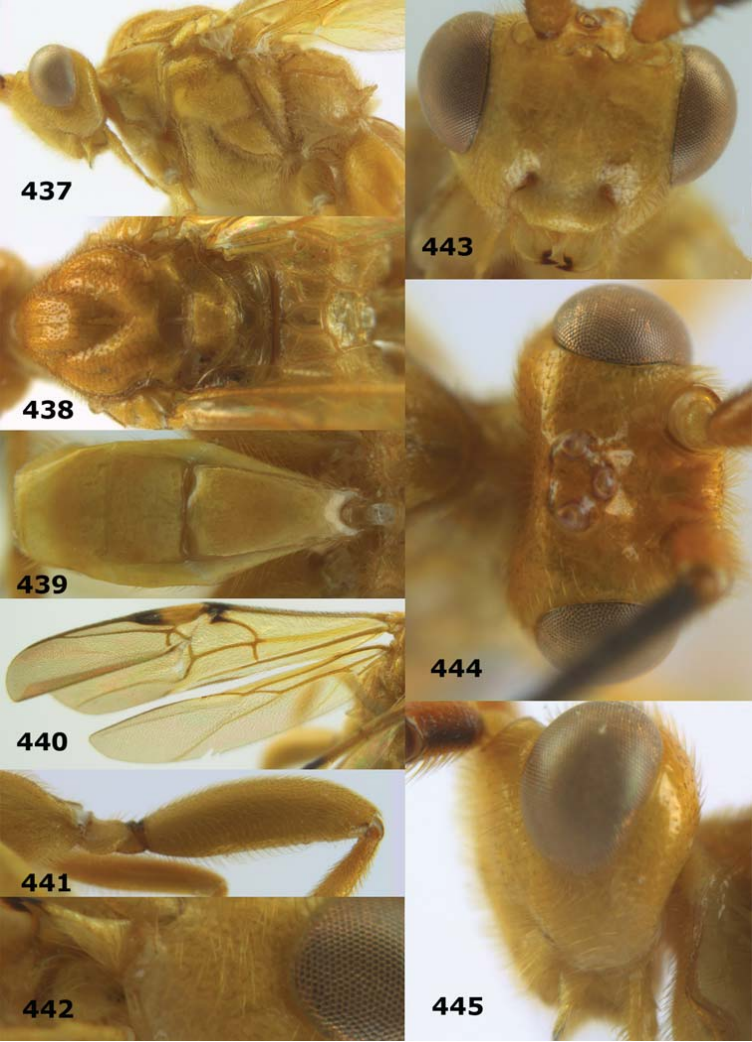
Zelodia brevifemoralis gen. n. sp. n., female, holotype. **437** head and mesosoma lateral **438** mesosoma dorsal **439** first-third metasomal tergites dorsal **440** wings **441** hind femur lateral **442** malar space lateral **443** head anterior **444** head dorsal **445** head lateral.

##### Biology.

Unknown.

##### Etymology.

From “brevis” (Latin for “short”) and “femur” (Latin for “foundation, base”), because of the short hind femur.

#### Zelodia flavistigma sp. n.

urn:lsid:zoobank.org:act:E5C1A5CD-5D01-4865-9A09-32CA74000225

Figs 446 –454 

##### Type material.

Holotype, ♀ (RMNH), Aga. 251a, “S. Vietnam: Dak Lak, Cu M’Gar, Ea Pok [forest], 10.vi.2005, K. Long”. Paratypes (9 ♀ + 14 ♂): 2 ♀ + 6 ♂ (IEBR, RMNH), same data as holotype; 4 ♀ + 8 ♂, (RMNH, IEBR), “S. Vietnam: Ban Me Thuat, park, 9.vi.2005, K.D. Long”; 2 ♀ (RMNH, IEBR), Aga. 250a and Aga. 251b, “S. Vietnam: Pleicu, Dac Do, 800 m, bushes, 8.vi.2005, K.D. Long”; 1 ♀ (IEBR), Aga. 223, ”S. Vietnam: Tay Ninh, Lo Go-Xa Mat N.P., 4.x.2003, K.D. Long”.

##### Diagnosis.

The new species resembles Zelodia dravida (Bhat & Gupta), but the latter differs by having the head, hind leg and metasoma black, the scutellum sparsely punctate, the second submarginal cell of fore wing triangular, the wing membrane subhyaline and the pterostigma entirely brown.

##### Description.

Holotype, ♀, length of body 7.2 mm, of fore wing 7.4 mm.

###### Head.

Antennal segments 43, length of third segment 1.1 times fourth segment, length of third, fourth and penultimate segments 2.5, 2.3 and 2.0 times their width, respectively; penultimate segment as long as apical segment; antenna densely setose; scapus nearly cylindrical, twice as long as wide; length of maxillary palp 0.9 times height of head; malar space 1.3 times as long as basal width of mandible; in dorsal view length of eye 3.4 times as long as temple (Figs 453, 454); lateral ocelli large, POL:OD:OOL = 5:4:5; face punctate, but rugose-punctate medially; frons shiny, smooth, without lateral carinae; vertex sparsely finely punctate; pair of crests between antennal sockets strong, convergent; occipital flange rather large, its ventral margin convex (Fig. 447).

###### Mesosoma.

Length of mesosoma 1.4 times its height; subpronope medium, shallow; side of pronotum smooth with sparse fine punctures posteriorly; area near lateral carina of mesoscutum smooth; mesoscutum with distinct punctures; middle lobe of mesoscutum slightly grooved; notauli narrow, rather deep and crenulate, fused posteriorly, forming a wide shallow groove (Fig. 448); scutellar sulcus 0.8 times as long as dorsal part of scutellum; scutellum rugose-punctate; subposterior crest curved (Fig. 448); precoxal sulcus wide and shallow, largely crenulate (Fig. 447); mesopleuron below as above precoxal sulcus with distinct fine punctures; upper side of metapleuron rugose-punctate, lower side with strong rugosity; propodeum shiny with areola, without basal medial carina (Fig. 448); propodeal spiracle elongate, 2.5 times as long as wide; distance between spiracle and lateral carina 1.4 times as long as width of spiracle.

###### Wings.

Fore wing: second submarginal cell narrowed anteriorly and r-m angled (Fig. 450); r:3-SR:SR1 = 5:3:72; 2-SR:3-SR:r-m = 13:3:12. Hind wing: M+CU 0.7 times as long as 1-M (30:45); surroundings of cu-a sparsely setose.

###### Legs.

Length of hind femur, tibia and basitarsus 3.3, 5.3 and 7.5 times their width, respectively; outer side of hind coxa punctate; hind femur (as remainder of legs) with long and dense setosity (Fig. 451); outer side of apex of hind tibia with two equal pegs; length of outer and inner spurs of middle tibia 0.5 and 0.9 times middle basitarsus, respectively; length of outer and inner spurs of hind tibia 0.3 and 0.4 times hind basitarsus.

###### Metasoma.

First tergite twice as long as its apical width (Fig. 449); second tergite 0.7 times as long as wide apically, second suture developed (Fig. 449); ovipositor sheath 0.3 times as long as hind basitarsus.

###### Colour.

Yellow; antenna (except yellow scapus) dark brown; membrane of fore wing yellowish and wing without stigmal spot; parastigma and apex of vein C+SC+R of fore wing dark brown; hind tarsus light brown.

###### Variation.

Antennal segments 44–45; apical antennal segment 1.2–1.3 times as long as penultimate segment; length of malar space 1.3–1.6 times basal width of mandible; length of first tergite 1.7–2.0 times as long as its apical width; second tergite 0.7–0.8 times as long as wide apically; hind tarsus blackish brown to light brown.

##### Distribution.

NE Vietnam: Bac Kan and S Vietnam: Ban Me Thuat, Dak Lak, Pleicu, Tay Ninh.

**Figure 446.**
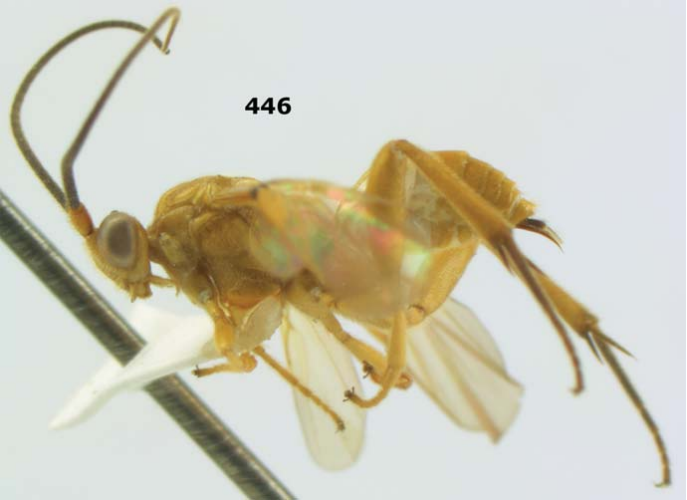
Zelodia flavistigma gen. n. sp. n., female, holotype. Habitus lateral.

**Figures 447–454.**
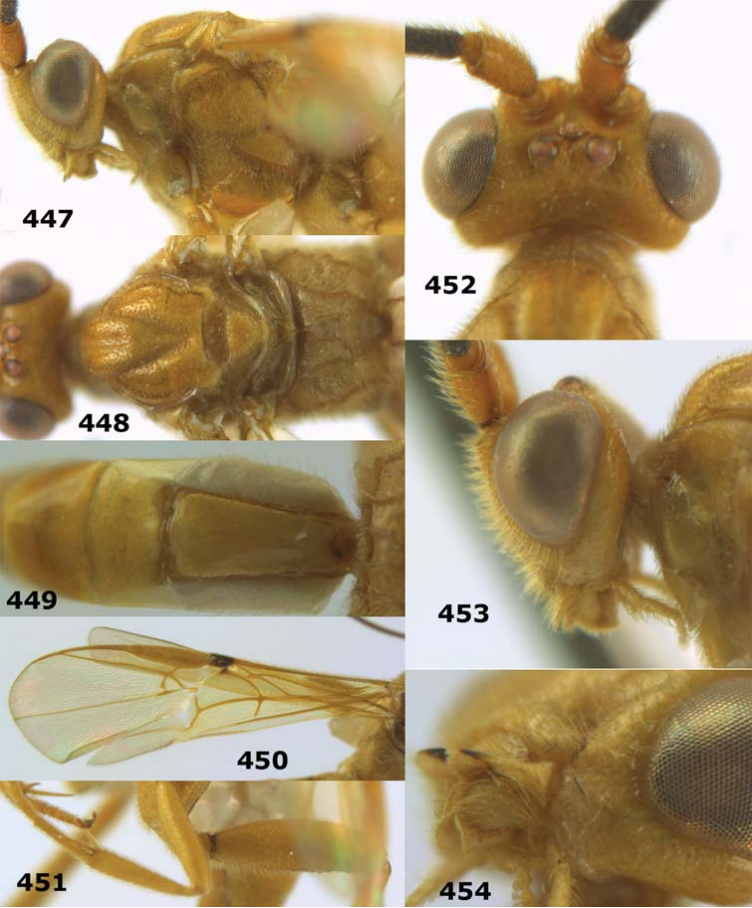
Zelodia flavistigma gen. n. sp. n., female, holotype. **447** head and mesosoma lateral **448** mesosoma dorsal **449** first-third metasomal tergites dorsal **450** wings **451** hind femur lateral **452** head dorsal **453** head lateral **454** malar space lateral.

##### Biology.

Unknown.

##### Etymology.

From “flavus” (Latin for “yellow”), and “stigma” (Greek for “mark”), because of the entirely yellow pterostigma.

#### Zelodia longidorsata

(Bhat & Gupta, 1977) comb. n.

Figs 455–457

Zelomorpha longidorsata Bhat and Gupta 1977: 252–254, Fig. 34f, 35c, d.

##### Distribution.

Central North Vietnam: Nghe An. Outside Vietnam known from India, China and Thailand. Thailand (RMNH) and Vietnam are new records.

**Figures 455–457.**
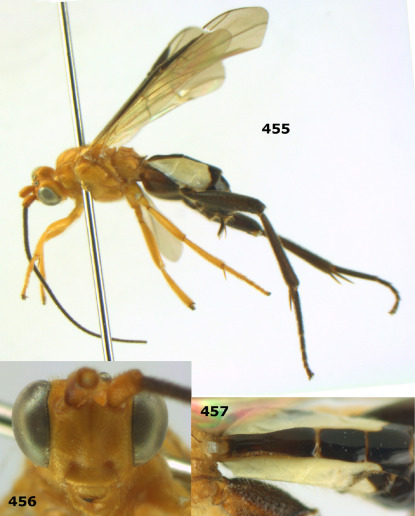
Zelodia longidorsata (Bhat & Gupta), female, Thailand. **455** habitus lateral **456** head anterior **457** first-third metasomal tergites dorsal.

#### Zelodia microcellata sp. n.

urn:lsid:zoobank.org:act:947D442E-B543-492E-8AC4-3E105B9A43AC

Figs 458 –467

Coccygidium varipes Chen and Yang 2006: 128–130, Fig. 50, plts 120–130.

##### Type material.

Holotype, ♂ (RMNH), “C. Vietnam: Thua Thien Hué, Phong Dién N.R., n[ea]r base-camp, 50–100 m, 25.iii.2001, C. van Achterberg, RMNH’01”.

##### Diagnosis.

The new species is close to the Palaearctic Zelodia varipes (van Achterberg & Maetô). The latter species differs by having the clypeus dorsally at lower level of eyes, hind femur finely sculptured, the pleural sulcus with shorter and more crenulae and rather narrow, the submedial cell of the fore wing largely glabrous and the mesoscutum dark brown.

The new species is similar to Zelodia quadrifossulata (Enderlein), but that species has the mesosoma completely yellowish-brown, OOL 1.6–2.0 times as long as POL and second tergite more or less yellowish or brown.

##### Description.

Holotype, ♂, length of body 6.0 mm, of fore wing 5.7 mm.

###### Head.

Antennal segments 39, densely bristly setose, setose length of third segment 1.2 times fourth segment, length of third, fourth and penultimate segments 3.0, 2.4 and 1.5 times their width, respectively; penultimate segment half as long as apical segment (including spine); scapus cylindrical, 2.1 times as long as wide; length of maxillary palp 0.6 times height of head; length of malar space twice basal with of mandible and 0.4 times height of head (Figs 464, 467); in dorsal view eye twice as long as temple (Fig. 466), in lateral view temple 0.55 times width of eye (Fig. 467), temple smooth, except for some fine punctures; ocelli comparatively small (Fig. 466), POL:OD:OOL = 6:5:9; face shiny and rather sparsely moderately punctate, no rugae medially; clypeus dorsally distinctly above lower level of eyes (Fig. 465); frons shiny, smooth, hardly depressed medially and without lateral carinae; vertex shiny and very sparsely finely punctate; pair of crests between antennal sockets weak, convergent; occipital flange large, its ventral margin convex (Fig. 464).

###### Mesosoma.

Length of mesosoma 1.3 times its height; subpronope medium-sized, rather shallow; side of pronotum smooth, but with some punctures dorsally and crenulate posteriorly; area near lateral carina of mesoscutum distinctly crenulate; mesoscutum distinctly rather densely punctate and shiny, but sparsely punctate posteriorly; middle lobe of mesoscutum slightly grooved; notauli rather narrow, rather deep and distinctly crenulate (Fig. 460); scutellar sulcus with 3 carinae and 0.8 times as long as dorsal face of scutellum; scutellum weakly convex and coarsely punctate; subposterior crest strong and curved (Fig. 460); precoxal sulcus distinct, nearly complete, anteriorly with some coarse sublongitudinal rugae, remainder moderately crenulate (Fig. 459); mesopleuron largely rather coarsely punctate with interspaces wider than punctures; pleural sulcus wide and coarsely crenulate, with 7, mostly coarse crenulae; metapleuron with long silvery setae, upper densely and coarsely punctate, lower half coarsely vermiculate rugose; propodeum coarsely areolate, without medial carina basally and with weak anterior transverse carina (Fig. 460); propodeal spiracle large, elliptical, 2.5 times as long as wide; distance between spiracle and lateral carina about equal to width of spiracle.

###### Wings.

Fore wing: second submarginal cell narrow anteriorly, without ramellus (Fig. 461); r:3-SR:SR1= 5:4:102; 2-SR:3-SR:r-m = 17:4:16; submedial cell sparsely but distinctly setose. Hind wing: M+CU 0.6 times as long as 1-M; surroundings of cu-a glabrous except for a few setae.

###### Legs.

Hind coxa rather pimply dorsally, basally punctate but largely smooth; length of hind femur, tibia and basitarsus 3.9, 6.7 and 7.6 times their width, respectively; hind femur coarsely punctate, ventrally coarsely rugose and with rather short setae (Fig. 463); outer side of apex of hind tibia with two pegs; fore tarsus slender and with long setae (as in other species); outer and inner spur of middle tibia 0.55 and 0.95 times middle basitarsus, respectively; outer and inner spur of hind tibia 0.4 and 0.7 times hind basitarsus.

###### Metasoma.

First tergite rather slender, smooth, slightly widened apically (Fig. 463); length of first tergite 1.9 times its apical width; second tergite rectangular and 0.8 times as long as wide apically, smooth with one transverse row of setae subapically; second metasomal suture obsolescent (Fig. 463).

###### Colour.

Dark brown (including antenna, pterostigma and veins); wing membrane slightly infuscate and without a distinct stigmal spot; head, pronotum and mesoscutum yellowish-brown; first tergite ivory; fore and middle legs (middle coxa, trochanter, trochantellus and base of middle femur dark brown and telotarsi slightly infuscate) brownish-yellow.

##### Distribution.

C Vietnam: Thua Thieu Hué. Further known from China (Fujian).

**Figure 458.**
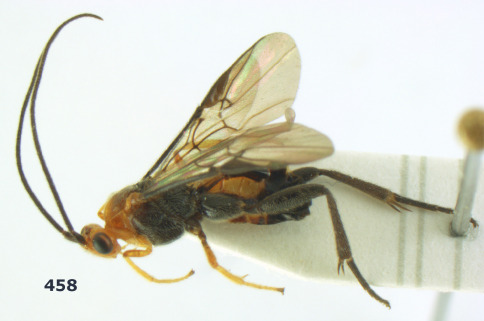
Zelodia microcellata gen. n. sp. n., male, holotype. Habitus lateral.

**Figures 459–467.**
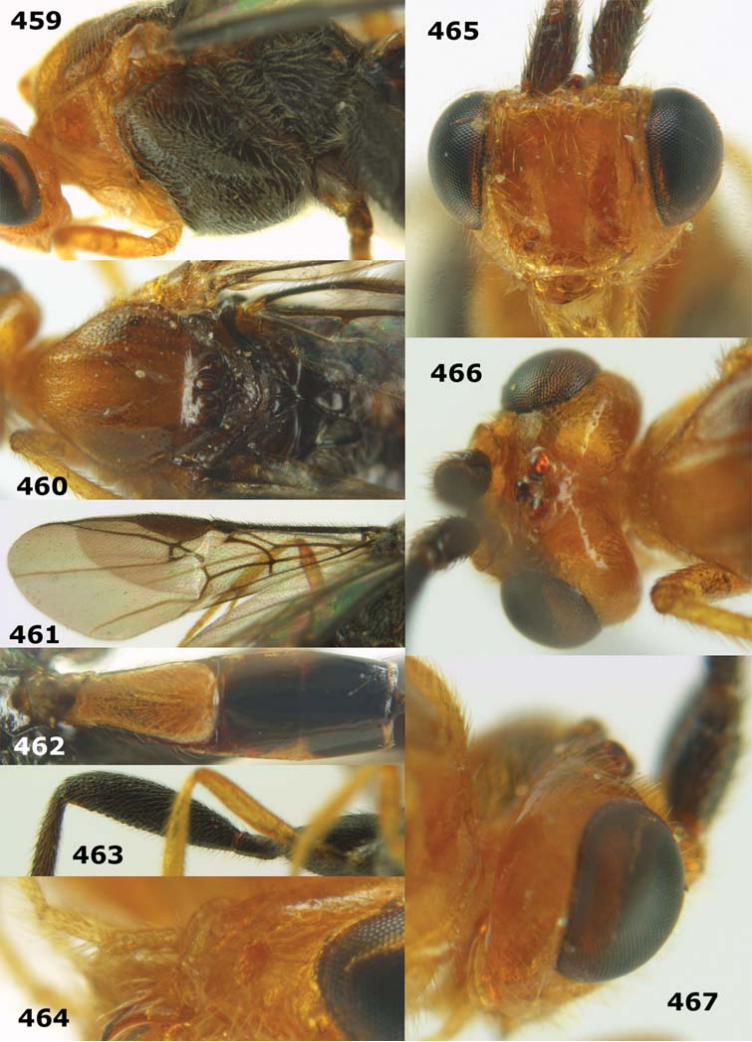
Zelodia microcellata gen. n. sp. n., male, holotype. **459** mesosoma lateral **460** mesosoma dorsal **461** wings **462** first-third metasomal tergites dorsal **463** hind femur lateral **464** malar space lateral **465** head anterior **466** head dorsal **467** head dorso-lateral.

##### Biology.

Unknown.

##### Etymology.

From “micro” (Greek for “small”), and “ocellus” (Latin for “small eye”), because of the small ocelli.

## Supplementary Material

XML Treatment for Agathis

XML Treatment for Bassus

XML Treatment for Biroia

XML Treatment for Braunsia

XML Treatment for Camptothlipsis

XML Treatment for Coccygidium

XML Treatment for Cremnops

XML Treatment for Disophrys

XML Treatment for Earinus

XML Treatment for Euagathis

XML Treatment for Gyragathis

XML Treatment for Gyrochus

XML Treatment for Lytopylus

XML Treatment for Therophilus

XML Treatment for Troticus

XML Treatment for Zelodia

## References

[B1] van Achterberg C (1974) The braconid types of Szépligeti in the Leiden Museum. Entomologische Berichten Amsterdam 34:79-80

[B2] van Achterberg C (1988) Revision of the subfamily Blacinae Foerster (Hymenoptera, Braconidae). Zoologische Verhandelingen Leiden 249:1-324

[B3] van Achterberg C (1990) Illustrated key to the subfamilies of the Holarctic Braconidae (Hymenoptera: Ichneumonoidea). Zoologische Mededelingen Leiden 64:1-20

[B4] van Achterberg C (1993) Illustrated key to the subfamilies of the Braconidae (Hymenoptera: Ichneumonoidea). Zoologische Verhandelingen Leiden 283:1-189

[B5] van Achterberg C (1997) Braconidae. An illustrated key to all subfamilies. ETI World Biodiversity Database CD-ROM Series Amsterdam.

[B6] van Achterberg C Chen XX (2002) Revision of the Euagathis species (Hymenoptera: Braconidae: Agathidinae) from China and northern Vietnam. Zoologische Mededelingen Leiden 76:309-346

[B7] Bhat S Gupta VK (1977) The subfamily Agathidinae (Hymenoptera, Braconidae). Ichneumonologia Orientalis 6. Oriental Insects Monograph 6:1-353

[B8] Chen JH Yang JQ (2006) Hymenoptera, Braconidae (IV), Agathidinae. Fauna Sinica Insecta 46:1-301

[B9] Chou LY Sharkey MJ (1989) The Braconidae (Hymenoptera) of Taiwan. 1. Agathidinae. Journal of Taiwan Museum 42 (1):147-223

[B10] Enderlein G (1920) Zur Kenntnis aussereuropäischer Braconiden. Archiv für Naturgeschichte (A)84: 51–224

[B11] Sharkey MJ (1996) The Agathidinae (Hymenoptera: Braconidae) of Japan. Bulletin of the National Institute of Agro-Environmental Sciences 13:1-100

[B12] Sharkey MJ Laurenne NM Sharanowski B Quicke DLJ Murray D (2006) Revision of the Agathidinae (Hymenoptera: Braconidae) with comparisons of static and dynamic alignments. Cladistics 22 (6):546-567 10.1111/j.1096-0031.2006.00121.x 34892897

[B13] Sharkey MJ Yu DS van Noort S Seltmann K Penev L (2009) Revision of the Oriental genera of Agathidinae (Hymenoptera, Braconidae) with an emphasis on Thailand including interactive keys to genera published in three different formats. ZooKeys 21:19-54

[B14] Simbolotti G van Achterberg C (1992) Revision of the West Palaearctic species of the genus Bassus Fabricius (Hymenoptera: Braconidae). Zoologische Verhandelingen Leiden 281:1-80

[B15] Simbolotti G van Achterberg C (1995) Revision of the Euagathis species (Hymenoptera: Braconidae) from Sunda region. Zoologische Verhandelingen Leiden 293 (1994):1-62

[B16] Stevens NB Austin AD Jennings JT (2010) Synopsis of Australian agathidine wasps (Hymenoptera: Braconidae: Agathidinae). Zootaxa 2480:1-26

[B17] Yang JQ Chen JH (1998) [One new species and one new record of the genus Coccygidium Saussure from China (Hymenoptera: Braconidae)]. Journal of Fujian Agricultural University 27(3): 332–336 [in Chinese with English summary].

[B18] Yu DS van Achterberg K Horstmann K (2007) Biological and taxonomical information: Ichneumonoidea 2006. Taxapad Interactive Catalogue, Lexington

